# A Dual Catalytic Approach for the Halogen-Bonding-Mediated Reductive Cleavage of α-Bromodifluoroesters and Amides

**DOI:** 10.1021/acs.joc.4c02413

**Published:** 2024-12-19

**Authors:** Tarannum Tasnim, Negin Shafiei, Katelyn J. Laminack, Bailey S. Robertson, Nash E. Nevels, Christopher J. Fennell, Spencer P. Pitre

**Affiliations:** Department of Chemistry, Oklahoma State University, 107 Physical Sciences, Stillwater, Oklahoma 74078, United States

## Abstract

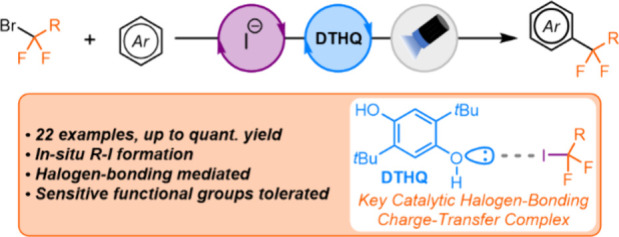

While charge-transfer complexes involving halogen-bonding interactions have emerged as an alternative strategy for the photogeneration of carbon radicals, examples using (fluoro)alkyl bromides are limited. This report describes a dual catalytic approach for radical generation from α-bromodifluoroesters and amides under visible-light irradiation. Mechanistic studies suggest that the reaction proceeds through *in situ* bromide displacement using a catalytic iodide salt, generating a C–I bond that can be engaged by our halogen-bonding photocatalysis platform.

The development of many mild, catalytic methods for the generation of carbon-centered radicals makes them an invaluable tool in chemical synthesis, particularly for the construction of C–C bonds.^1^ However, the requirement for prior synthetic steps to activate substrates as radical precursors detracts from the potential utility of the elegant radical reactions that have been developed.^2^ In this regard, alkyl halides are an ideal solution, as structurally diverse alkyl halides are readily available from commercial suppliers, and these substrates do not require prior synthetic steps to participate in radical reactions. The traditional method of generating carbon radicals from alkyl halides was the use of tributyltin hydride;^3^ however, the chemical community has moved on from these reactions owing to the toxicity of tin. The more contemporary approaches involve the reductive cleavage of the C–X bond of the alkyl halide using a sufficiently reducing source of electrochemical potential, like an excited state photocatalyst (Scheme 1A).^4^ However, as these approaches rely on generating sufficient electrochemical potential to render the single-electron transfer (SET) to the alkyl halide exergonic, groups that could otherwise be reduced at these redox potentials cannot be included in the reaction substrates, thereby diminishing the functional group tolerance of these reactions. Another more recent strategy involves halogen atom abstraction via a photocatalytically generated silyl, α-aminoalkyl, or aryl radical species.^5^ While these reactions enable carbon radical generation from a broad scope of alkyl halides, these methods generally require stoichiometric amounts of the halogen atom-transfer agent.

**Scheme 1:**
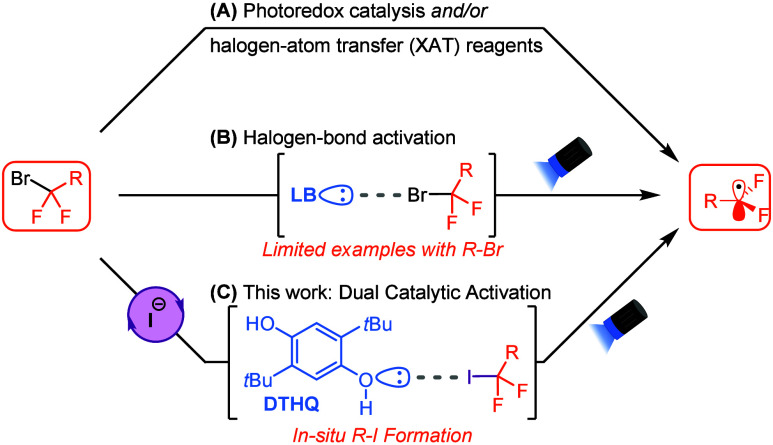
Modern Approaches to Carbon Radical Formation from Alkyl Bromides

Charge-transfer complexes (CTCs) involving halogen-bonding interactions between a Lewis base and a C–X bond have emerged as an alternative strategy for the photogeneration of carbon radicals from alkyl halides.^6^ A halogen-bonding interaction can be described as consisting of a partial n → σ* charge transfer from the nonbonding orbital of the Lewis base (halogen-bond acceptor) and the σ* orbital of the C–X bond (halogen-bond donor).^7^ Previous reports have demonstrated that stoichiometric Lewis bases, especially aliphatic amines, can efficiently form CTCs with alkyl iodides through halogen-bonding interactions, enabling a range of visible-light-mediated radical transformations.^6c,8^ More recently, efforts to employ catalytic Lewis bases, such as amines,^9^ pyridines,^10^ phosphines,^11^ and phenols,^12^ have been reported. However, methods for the activation of alkyl bromides using a halogen-bonding strategy are limited,^10,13^ as they are weaker halogen-bond donors compared to alkyl iodides (Scheme 1B).^14^ Therefore, a general strategy for expanding this reactivity to C–Br bonds would be highly beneficial, as alkyl bromides are inherently more bench-stable and more widely available compared to their alkyl iodide counterparts.

Herein, we report a dual catalytic approach for the generation of carbon radicals from α-bromodifluoroesters and amides under visible-light irradiation. Leveraging our prior experience in developing halogen-bonding photocatalyzed reactions,^12a^ our strategy involves *in situ* bromide displacement using a catalytic Finkelstein-type reaction with an iodide salt to generate a stronger C–I halogen-bond donor, facilitating formation of the key visible-light-absorbing CTC with our halogen-bond acceptor catalyst, 2,5-di-*tert*-butylhydroquinone [DTHQ (Scheme 1C)]. Our approach has enabled the radical coupling of a series of α-bromodifluoroesters and amides with a variety of electron-rich (hetero)aromatics. Insights into the underlying mechanism of the reaction are also presented.

We chose to begin our investigation of the dual catalytic approach for carbon radical generation from C–Br bonds with the *gem*-difluoroalkylation of 1,3,5-trimethoxybenzene (**1**) using ethyl bromodifluoroacetate (**2**) photocatalyzed by DTHQ under 427 nm irradiation as the model system (Table 1; see the Supporting Information for full optimization). In a preliminary set of experiments, it was determined that 20 mol % tetra-*N*-butylammonium iodide (Bu_4_NI) in DMSO at 55 °C gave the optimal yield of *gem*-difluoroalkylated product **3** (entry 1). The absence of Bu_4_NI resulted in a significant decrease in the yield of **3** (entry 2), consistent with our prior results that indicated that fluoroalkyl bromides do not react efficiently with our DTHQ halogen-bonding photocatalyst.^12a^ A small amount of conversion was observed in the absence of the DTHQ (entry 3), likely owing to background reactivity from an observed CTC between I^–^ and **2** (see the Supporting Information for further details).^15^ Highlighting the importance of the electron-rich hydroxyl groups on DTHQ for catalytic activity,^12a^ substituting DTHQ for 1,4-di-*tert*-butyl-2,5-dimethoxybenzene resulted in the same yield observed in the absence of DTHQ (entry 4). Further control experiments also indicated that the base, degassed conditions, and LED irradiation were all necessary for optimal reactivity (entries 5–7, respectively). Conducting the reaction at room temperature resulted in a significant decrease in reaction efficiency (entry 8). Finally, addition of TEMPO to the reaction mixture resulted in complete suppression of the formation of **3**, and the TEMPO–CF_2_CO_2_Et adduct was observed in 39% yield, providing support for a radical mechanism (see the Supporting Information).

**Table 1:**
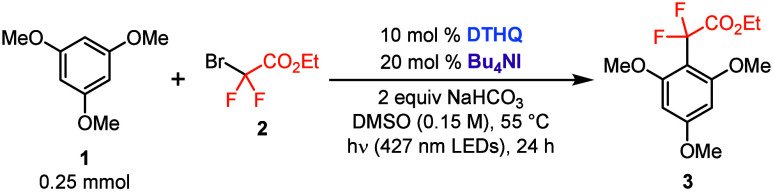
Optimized Conditions and Control Reactions

entry	modifications from the standard conditions	yield of **3** (%)
1	none	87
2	no Bu_4_NI	31
3	no DTHQ	11
4	1,4-di-*tert*-butyl-2,5-dimethoxybenzene instead of DTHQ	10
5	no NaHCO_3_	26
6	under air	22
7	no light	trace
8	room temperature	29

With the optimized conditions identified, we examined the scope of the dual-catalyzed *gem*-difluoroalkylation of electron-rich (hetero)arenes (Scheme 2). Using **2** as the radical precursor, several electron-rich aromatics were *gem*-difluoroalkylated in moderate to good yields (**3**–**6**). Electron-rich heteroarenes, such as 2,6-dimethoxypyridine (**7**), benzofurans (**8**), benzothiaphenes (**9**), and pyrroles (**10**) were also well tolerated. Notably, the reaction with *N*-phenylpyrrole (**10**) could be performed on a 1 mmol scale with no loss of reactivity (see the Supporting Information). Medicinally relevant heteroarenes such as caffeine (**11**), Boc-Trp-OH (**12**), melatonin (**13**), coumarin (**14**), and uracil (**15** and **16**) were all compatible substrates for our *gem*-difluoroalkylation reaction. Next, the scope of α-bromodifluoroesters and amides was evaluated using *N*-phenylpyrrole as the coupling partner. α-Bromodifluoroesters derived from adamantanol and l-menthol provided *gem*-difluoroalkylated products **17** and **18**, respectively, in good yields. A series of α-bromodifluoroamides also reacted smoothly under our reaction conditions (**19**–**22**). Aryl difluoroamides are often found in biologically active compounds, such as FKBP12 inhibitors,^16^ highlighting the potential utility of this method to provide facile access to these scaffolds. Finally, perfluorobutyl bromide^17^ (**23**) and an α-bromodifluorobenzoxazole derivative (**24**) were also competent radical precursors using this approach. In every case, removing the Bu_4_NI resulted in decreased yields for the *gem*-difluoroalkylated products (see Scheme 2).

**Scheme 2:**
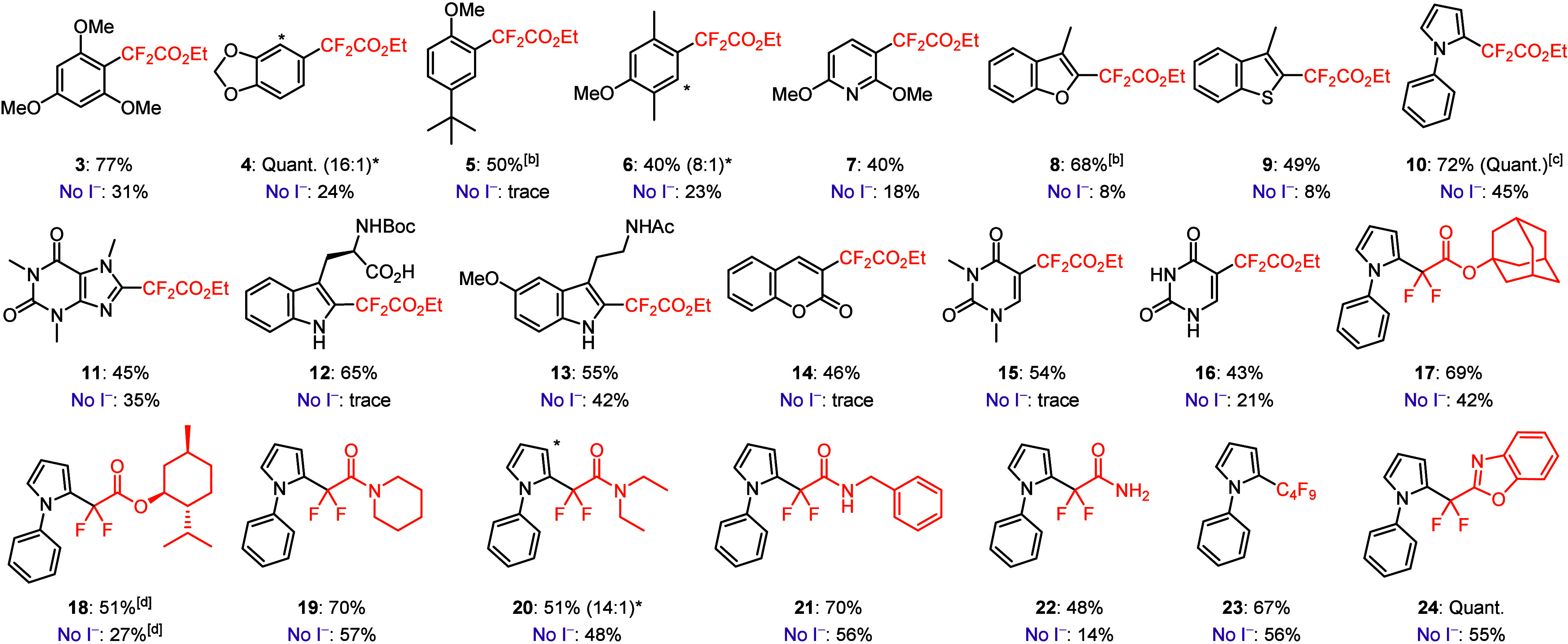
Reaction Scope For the standard conditions, (hetero)arene (0.5 mmol), R-Br (2.5 equiv), DTHQ (10 mol %), Bu_4_NI (20 mol %), and NaHCO_3_ (2 equiv) in DMSO (0.15 M) were irradiated under argon with two Kessil PR-160L 427 nm LEDs for 24 h at 55 °C. Yields are reported as isolated yields of the purified products. The control reactions in the absence of Bu_4_NI (No I^–^) were performed on a 0.15 mmol scale, and the yields were determined by ^19^F NMR using C_6_F_6_ as an external standard. With 40 mol % Bu_4_NI. Reaction performed on a 1 mmol scale. (Hetero)arene (2.5 equiv) and R-Br (0.5 mmol).

As our approach for radical generation from these α-halodifluoroesters and amides proceeds through direct activation of the C–X bond through a halogen-bonding interaction,^12a^ we hypothesized that our method may tolerate sensitive functional groups that would otherwise not be amenable to a highly reducing environment. To this end, we performed a small robustness screen by adding substrates with functional groups prone to single-electron reduction (see the Supporting Information for details).^18^ Our observations using the *gem*-difluoroalkylation of *N*-phenylpyrrole as the model reaction are summarized in Scheme 3. In general, aldehydes, trifluoroacetic anhydrides, and acid chlorides were all well tolerated under our reaction conditions, having either a negligible or a modest impact on the overall yield of **10**. It is noteworthy that trifluoroacetic anhydride, the additive tested with the lowest reduction potential (*E*_p/2_ = −0.20 V vs SCE),^19^ was completely recovered after the reaction, indicating no competitive reduction of the additive had occurred. In all cases, >70% of the additive was recovered after the reaction. These results highlight the benefit of our halogen-bonding approach, providing support for the direct activation and reduction of C–X bonds even in the presence of other easily reducible functional groups. Finally, nitro groups and sulfonyl chlorides were found to be incompatible under our reaction conditions, significantly impacting the yield of **10**.

**Scheme 3:**
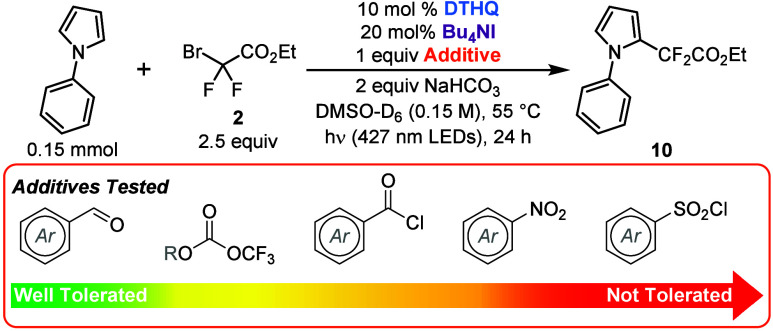
Sensitive Functional Group Screen

Next, we performed a series of mechanistic studies to understand the role of Bu_4_NI in our reaction. Using our initial model system, we sought to determine the effect of Bu_4_NI on the initial rate of the reaction (Scheme 4A). As anticipated, the rate of the reaction is significantly enhanced when starting with ethyl iododifluoroacetate (**25**) versus **2**, as **25** is expected to be a significantly stronger halogen-bond donor compared to **2**.^14^ In support of this hypothesis, the association constants (*K*_a_) for the [DTHQ:**25**] and [DTHQ:**2**] complexes were found to be 0.43 and 0.013 M^–1^, respectively (see the Supporting Information). Furthermore, the [DTHQ:**25**] complex absorbs more efficiently above 400 nm compared to the [DTHQ:**2**] complex (see the Supporting Information), highlighting that *in situ* formation of **25** is likely required for efficient reactivity under 427 nm LED irradiation. In this vein, the initial rate was observed to increase almost proportionally for the reaction with **2** upon addition of increasing concentrations of Bu_4_NI (see Scheme 4A). These data support the involvement of Bu_4_NI in the rate-determining step of the reaction.

To provide support for our hypothesis that our reaction is proceeding via the *in situ* formation of *gem*-difluoroalkyl iodides, we performed a series of computational studies. Optimization and frequency density functional theory calculations of all possible combinations of reactants, products, and transition state complexes were performed using Gaussian16.^20^ These were performed in a DMSO polarized continuum solvent using the B3LYP functional with dispersion corrections and the Weigend and Ahlrichs basis set projected to the continuous basis set limit.^21^ Comparisons of free energy estimations (Scheme 4B) show that **25** halogen-bonded to either a free iodide or bromide is thermodynamically preferable relative to **2** by 1.5 or 1.6 kcal/mol, respectively. The activation energy for the Finkelstein reaction between the *gem*-difluoroalkyl bromide and free I^–^ is 23.4 kcal/mol. This barrier is lower than that calculated for other systems known to undergo the Finkelstein reaction (see the Supporting Information), supporting the feasibility of this step in our system. In good agreement, we were also able to experimentally observe the *in situ* formation of **25** in control reactions between **2** and Bu_4_NI under the standard conditions (see the Supporting Information).^22^

**Scheme 4:**
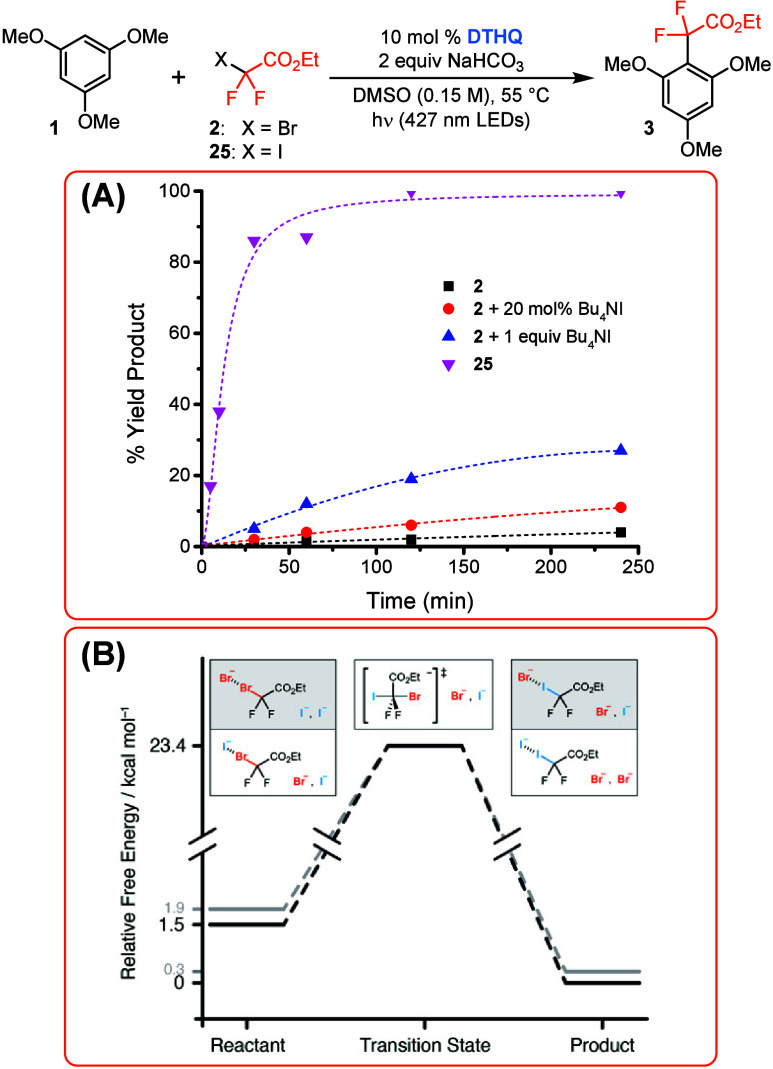
(A) Effect of Bu_4_NI on the Initial Reaction Rate and (B) Free Energy Diagram for the Proposed Finkelstein Displacement Step

The proposed mechanism for this transformation is outlined in Scheme 5. Initially, the α-bromodifluoroester or amide undergoes *in situ* bromide displacement by I^–^, generating CTC ***A***. CTC ***A*** can be intercepted by DTHQ to generate photoactive CTC ***B***, which upon excitation produces the difluoroalkyl radical, DTHQ^•+^, and regenerates I^–^. The difluoroalkyl radical subsequently adds to the electron-rich (hetero)arene to give intermediate **I**, which undergoes a HAT reaction with the DTHQ phenoxy radical (**II**) to yield the *gem*-difluoroalkylated (hetero)arene (**III**) and DTHQ, completing the catalyst turnover step. Given our mechanistic data, we have also identified two minor pathways that lead to product formation. The first involves the inefficient formation of the weak halogen-bonding complex between DTHQ and the α-bromodifluoroester or amide (CTC ***C***), which upon excitation also leads to product formation (see Table 1, entry 2). A second potential minor pathway involves the direct excitation of CTC ***A*** (or any of the variants shown in Scheme 4B), resulting in intracomplex single-electron transfer to eventually furnish difluoroalkyl radicals. This is supported by the observed inefficient product formation in the absence of the DTHQ catalyst (see entry 3 of Table 1).

**Scheme 5:**
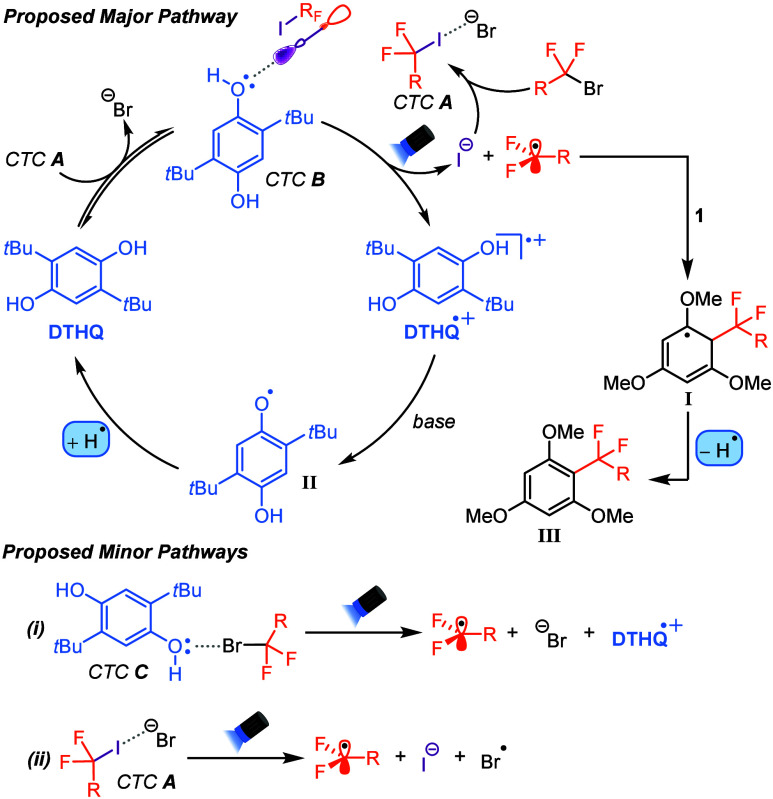
Proposed Mechanism for the Dual-Catalyzed Radical *gem*-Difluoroalkylation Reactions

In summary, we have developed a dual catalytic approach for the generation of carbon radicals from α-bromodifluoroesters and amides under visible-light irradiation. The reaction is proposed to proceed via an *in situ* bromide displacement mediated by catalytic I^–^, generating a *gem*-difluoroalkyl iodide that can be engaged by our DTHQ halogen-bonding photocatalyst. Given the greater abundance and increased stability of alkyl bromides compared to those of their iodide counterparts, we anticipate that this strategy can serve as a general solution for expanding halogen-bonding-mediated photochemical radical generation to less reactive alkyl bromide substrates, greatly increasing the scope of these transformations.

## Experimental Section

### General

All reactions were conducted in oven-dried glassware under an atmosphere of argon, unless otherwise stated. All solvents and reagents were purchased from commercial suppliers (Fisher Scientific, TCI America, Sigma-Aldrich, Oakwood Chemicals, Ambeed, and Combi-Blocks Inc.) and used as received unless otherwise noted. All photochemistry experiments were performed using two Kessil PR-160L 427 nm LEDs (40 W output at 100% intensity) placed 2.5 cm from the reaction vessels equipped with an overhead fan to maintain the temperature at ∼55 °C. Thin-layer chromatography (TLC) was conducted with silica gel 60 F254 precoated plates (250 μm) and visualized by exposure to ultraviolet (UV) light (254 nm) or potassium permanganate (KMnO_4_) staining. Flash column chromatography was performed using a Biotage Isolera Four instrument equipped with Sorbtech Purity flash column cartridges (60 Å porosity, 40–75 μm). ^1^H nuclear magnetic resonance (NMR) spectra were recorded at 400 MHz and are reported relative to deuterated solvent signals. Data for ^1^H NMR spectra are reported as follows: chemical shift (δ in parts per million), multiplicity, coupling constant (hertz), and integration. ^13^C NMR spectra were recorded at 101 or 201 MHz. Data for ^13^C NMR spectra are reported in terms of chemical shift. ^19^F NMR spectra were recorded at 376 MHz. Data for ^19^F NMR spectra are reported as follows: chemical shift, multiplicity, coupling constant (hertz), and integration. IR spectra were recorded on a Shimadzu IRAffinity-1S FT-IR spectrophotometer equipped with a QATR 10 single reflectance ATR accessory and are reported in terms of the frequency of absorption (inverse centimeters). High-resolution mass spectra were recorded with a quadrupole Orbitrap hybrid mass spectrometer at Oklahoma State University. Absorption spectra were recorded on a Shimadzu UV-2600 UV–visible spectrophotometer.

### Synthesis of α-Bromodifluoroesters

To a stirred solution of 2-bromo-2,2-difluoroacetic acid (1.75 g, 10 mmol, 1 equiv) and oxalyl chloride (1.7 mL, 20 mmol, 2 equiv) in 20 mL of dry CH_2_Cl_2_ was added 8 drops of DMF at room temperature under an argon atmosphere. After 2 h, the reaction mixture was cooled to 0 °C, and a solution of the corresponding alcohol (10.5 mmol, 1.05 equiv) and NEt_3_ (1.53 mL, 11 mmol, 1.1 equiv) in 20 mL of dry CH_2_Cl_2_ was added dropwise over 20 min. The reaction mixture was allowed to warm to room temperature and stirred for 24 h or until TLC analysis indicated the reaction had reached completion. The reaction mixture was then transferred to a separatory funnel and washed with 25 mL of H_2_O. The aqueous phase was then extracted with 25 mL of CH_2_Cl_2_, and the combined organic phases were washed with 25 mL of saturated NaHCO_3(aq)_, dried with MgSO_4_, and concentrated. The crude mixture was purified by flash column chromatography to afford the corresponding α-bromodifluoroesters. Alternatively, the same procedure can be employed starting directly from bromodifluoroacetyl chloride (940 μL, 10 mmol). All α-bromodifluoroesters synthesized were known compounds, and the spectral data agreed with those previously reported.^23^

### Synthesis of α-Bromodifluoroamides

A neat mixture of bromodifluoroacetate (1.28 mL, 10 mmol, 1 equiv) and the corresponding amine (10 mmol, 1 equiv) was stirred at room temperature for 24 h or until TLC analysis indicated the reaction had reached completion. The neat reaction mixture was directly purified by flash column chromatography to afford the corresponding α-bromodifluoroamides. All α-bromodifluoroamides synthesized were known compounds, and the spectral data agreed with those previously reported.^24^

### General Procedure for Radical Fluoroalkylation Reactions

An oven-dried two-dram vial equipped with a magnetic stir bar was charged with a (hetero)arene (0.5 mmol, 1 equiv), DTHQ (11.5 mg, 0.05 mmol, 10 mol %), Bu_4_NI (37 mg, 0.10 mmol, 20 mol %), and NaHCO_3_ (84.0 mg, 1.0 mmol, 2 equiv). DMSO was added (3.3 mL, 0.15 M), and the reaction mixture was degassed by sparging with argon for 5–6 min. To this was added fluoroalkyl bromide (1.25 mmol, 2.5 equiv) under argon. The reaction mixture was then sonicated and irradiated with two Kessil PR-160L 427 nm LED lamps for 24 h at ∼55 °C. The reaction mixture was transferred into a separatory funnel, diluted with 20 mL of DCM, and washed with 20 mL of 10 mM Na_2_S_2_O_3(aq)_. The aqueous phase was extracted with 20 mL of DCM. The combined organic phases were dried with MgSO_4_ and concentrated. The crude reaction mixture was purified by flash column chromatography using a Biotage Isolera Four instrument. Yields are reported as isolated yields of the purified products.

### Characterization Data

#### Ethyl 2,2-Difluoro-2-(2,4,6-trimethoxyphenyl)acetate (**3**)^25^

The title compound was prepared according to the general procedure from **1** (84 mg, 0.5 mmol, 1 equiv), **2** (162 μL, 1.25 mmol, 2.5 equiv), DTHQ (11.5 mg, 0.05 mmol, 10 mol %), Bu_4_NI (37 mg, 0.10 mmol, 20 mol %), and NaHCO_3_ (84 mg, 1.0 mmol, 2 equiv) in 3.3 mL of dry DMSO. The reaction mixture was irradiated with two Kessil 427 nm LEDs for 24 h at 55 °C and purified by flash column chromatography (0% → 60% EtOAc in Hex) to give the title compound as a white solid in 77% yield (113 mg): ^1^H NMR (400 MHz, CDCl_3_) δ 6.12 (s, 2H), 4.32 (q, *J* = 7.1 Hz, 2H), 3.82 (s, 3H), 3.79 (s, 6H), 1.33 (t, *J* = 7.1 Hz, 3H); ^13^C{^1^H} NMR (201 MHz, CDCl_3_) δ 164.8 (t, *J* = 33.3 Hz), 163.3, 160.2–160.1 (m), 113.4 (t, *J* = 247.7 Hz), 102.8 (t, *J* = 24.2 Hz), 91.4, 62.3, 56.2, 55.4, 14.0; ^19^F NMR (376 MHz, CDCl_3_) δ −96.23 (s, 2F); *R_f_* = 0.45 (2:1 Hex/EtOAc).

#### Ethyl 2-(Benzo[*d*][1,3]dioxol-5-yl)-2,2-difluoroacetate (**4**)^26^

The title compound was prepared according to the general procedure from 1,2-methylene dioxybenzene (58 μL, 0.5 mmol, 1 equiv), **2** (162 μL, 1.25 mmol, 2.5 equiv), DTHQ (11.5 mg, 0.05 mmol, 10 mol %), Bu_4_NI (37 mg, 0.10 mmol, 20 mol %), and NaHCO_3_ (84 mg, 1.0 mmol, 2 equiv) in 3.3 mL of dry DMSO. The reaction mixture was irradiated with two Kessil 427 nm LEDs for 24 h at 55 °C and purified by flash column chromatography (0% → 10% EtOAc in Hex) to give a 16:1 regioisomeric mixture of the title compound as a colorless oil in quantitative yield (124 mg): ^1^H NMR (400 MHz, CDCl_3_) δ 7.13–7.09 (m, 1H), 7.07–7.04 (m, 1H), 6.85 (d, *J* = 8.2 Hz, 1H), 6.02 (s, 1H), 4.30 (q, *J* = 7.1 Hz, 2H), 1.31 (t, *J* = 7.1 Hz, 3H); ^13^C{^1^H} NMR (201 MHz, CDCl_3_) δ 164.2 (t, *J* = 35.9 Hz), 149.8, 147.9, 126.5 (t, *J* = 26.0 Hz), 121.8, 119.9 (t, *J* = 6.8 Hz), 114.5, 111.9, 108.3, 106.1 (t, *J* = 6.3 Hz), 101.7, 63.1, 13.9; ^19^F NMR (376 MHz, CDCl_3_) δ −101.83 (s, 2F); *R_f_* = 0.51 (4:1 Hex/EtOAc).

#### Ethyl 2-[5-(*tert*-Butyl)-2-methoxyphenyl]-2,2-difluoroacetate (**5**)

The title compound was prepared according to the general procedure from 1-(*tert*-butyl)-4-methoxybenzene (88 μL, 0.5 mmol), **2** (162 μL, 1.25 mmol, 2.5 equiv), DTHQ (11.5 mg, 0.05 mmol, 10 mol %), Bu_4_NI (74 mg, 0.20 mmol, 40 mol %), and NaHCO_3_ (84 mg, 1.0 mmol, 2 equiv) in 3.3 mL of dry DMSO. The reaction mixture was irradiated with two Kessil 427 nm LEDs for 24 h at 55 °C and purified by flash column chromatography (0% → 20% EtOAc in Hex) to give the title compound as a colorless oil in 50% yield (71 mg): ^1^H NMR (400 MHz, CDCl_3_) δ 7.65 (s, 1H), 7.46 (d, *J* = 8.8 Hz, 1H), 6.87 (d, *J* = 8.6 Hz, 1H), 4.34 (q, *J* = 7.1 Hz, 2H), 3.79 (s, 3H), 1.35–1.28 (m, 12H); ^13^C{^1^H} NMR (201 MHz, CDCl_3_) δ 164.3 (t, *J* = 33.8 Hz), 154.4 (t, *J* = 5.0 Hz), 143.5, 129.1, 123.1 (t, *J* = 7.2 Hz), 121.1 (t, *J* = 23.4 Hz), 112.5 (t, *J* = 248.1 Hz), 111.0, 62.6, 55.7, 34.3, 31.4, 13.9; ^19^F NMR (376 MHz, CDCl_3_) δ −102.02 (s, 2F); *R_f_* = 0.57 (4:1 Hex/EtOAc); HRMS (ESI) *m*/*z* [M + H]^+^ calcd for C_15_H_20_F_2_O_3_H 287.1459, found 287.1456; IR (neat, cm^–1^) 2964, 2907, 2872, 1774, 1616, 1505, 1464, 1444, 1365, 1303, 1273, 1256, 1229, 1181, 1158, 1100, 1072, 1032, 901, 819, 766, 693.

#### Ethyl 2,2-Difluoro-2-(4-methoxy-2,5-dimethylphenyl)acetate (**6**)

The title compound was prepared according to the general procedure from 2-methoxy-1,4-dimethylbenzene (71 μL, 0.5 mmol), **2** (162 μL, 1.25 mmol, 2.5 equiv), DTHQ (11.5 mg, 0.05 mmol, 10 mol %), Bu_4_NI (37 mg, 0.10 mmol, 20 mol %), and NaHCO_3_ (84 mg, 1.0 mmol, 2 equiv) in 3.3 mL of dry DMSO. The reaction mixture was irradiated with two Kessil 427 nm LEDs for 24 h at 55 °C and purified by flash column chromatography (0% → 10% EtOAc in Hex) to give an 8:1 regioisomeric mixture of the title compound as a colorless oil in 40% yield (51 mg): ^1^H NMR (400 MHz, CDCl_3_) δ 7.31 (s, 1H), 6.64 (s, 1H), 4.31 (q, *J* = 7.1 Hz, 2H), 3.84 (s, 3H), 2.39 (s, 2H), 2.20 (s, 3H), 1.31 (t, *J* = 7.2 Hz, 3H); ^13^C{^1^H} NMR (201 MHz, CDCl_3_) δ 164.6 (t, *J* = 36.0 Hz), 159.1, 158.0, 136.4, 135.5, 128.4 (t, *J* = 8.5 Hz), 124.0, 122.5 (t, *J* = 23.9 Hz), 118.3 (t, *J* = 9.1 Hz), 115.8–113.4 (m), 113.1, 62.9, 55.3, 19.7, 15.7, 13.9; ^19^F NMR (376 MHz, CDCl_3_) δ −99.71 (s, 2F); *R_f_* = 0.66 (4:1 Hex/EtOAc); HRMS (ESI) *m*/*z* [M + H]^+^ calcd for C_13_H_16_F_2_O_3_H 259.1146, found 259.1139; IR (neat, cm^–1^) 2939, 2855, 1761, 1617, 1579, 1512, 1465, 1322, 1285, 1262, 1134, 1084, 1040, 948, 894, 850, 761.

#### Ethyl 2-(2,6-Dimethoxypyridin-3-yl)-2,2-difluoroacetate (**7**)^11a^

The title compound was prepared according to the general procedure from 2,6-dimethoxypyridine (66 μL, 0.5 mmol), **2** (162 μL, 1.25 mmol, 2.5 equiv), DTHQ (11.5 mg, 0.05 mmol, 10 mol %), Bu_4_NI (37 mg, 0.10 mmol, 20 mol %), and NaHCO_3_ (84 mg, 1.0 mmol, 2 equiv) in 3.3 mL of dry DMSO. The reaction mixture was irradiated with two Kessil 427 nm LEDs for 24 h at 55 °C and purified by flash column chromatography (0% → 20% EtOAc in Hex) to give the title compound as a colorless oil in 40% yield (52 mg): ^1^H NMR (400 MHz, CDCl_3_) δ 7.80 (d, *J* = 8.3 Hz, 1H), 6.38 (d, *J* = 8.2 Hz, 1H), 4.33 (q, *J* = 7.1 Hz, 2H), 3.94 (s, 3H), 3.93 (s, 3H), 1.31 (t, *J* = 7.1 Hz, 3H); ^13^C{^1^H} NMR (201 MHz, CDCl_3_) δ 165.2, 164.2 (t, *J* = 34.5 Hz), 160.1 (t, *J* = 5.2 Hz), 138.7 (t, *J* = 6.2 Hz), 112.6 (t, *J* = 247.9 Hz), 107.6 (t, *J* = 26.3 Hz), 101.7, 63.2, 54.2, 54.0, 14.3; ^19^F NMR (376 MHz, CDCl_3_) δ −101.68 (s, 2F); *R_f_* = 0.6 (4:1 Hex/EtOAc).

#### Ethyl 2,2-Difluoro-2-(3-methylbenzofuran-2-yl)acetate (**8**)^27^

The title compound was prepared according to the general procedure from 3-methylbenzofuran (64 μL, 0.5 mmol), **2** (162 μL, 1.25 mmol, 2.5 equiv), DTHQ (11.5 mg, 0.05 mmol, 10 mol %), Bu_4_NI (74 mg, 0.20 mmol, 40 mol %), and NaHCO_3_ (84 mg, 1.0 mmol, 2 equiv) in 3.3 mL of dry DMSO. The reaction mixture was irradiated with two Kessil 427 nm LEDs for 24 h at 55 °C and purified by flash column chromatography (0% → 10% EtOAc in Hex) to give the title compound as a colorless oil in 68% yield (85 mg): ^1^H NMR (400 MHz, CDCl_3_) δ 7.59 (d, *J* = 7.7 Hz, 1H), 7.49 (d, *J* = 8.3 Hz, 1H), 7.39 (t, *J* = 7.7 Hz, 1H), 7.31 (t, *J* = 7.5 Hz, 1H), 4.39 (q, *J* = 7.1 Hz, 2H), 2.42 (s, 3H), 1.36 (t, *J* = 7.1 Hz, 3H); ^13^C{^1^H} NMR (201 MHz, CDCl_3_) δ 162.5 (t, *J* = 34.1 Hz), 154.2, 141.0 (t, *J* = 32.3 Hz), 128.9, 126.3, 123.1, 120.4, 118.1, 111.6, 110.4 (t, *J* = 249.6 Hz), 63.6, 13.9, 7.7; ^19^F NMR (376 MHz, CDCl_3_) δ −103.42 (s, 2F); *R_f_* = 0.65 (4:1 Hex/EtOAc).

#### Ethyl 2,2-Difluoro-2-(3-methylbenzo[*b*]thiophen-2-yl)acetate (**9**)^28^

The title compound was prepared according to the general procedure from 3-methyl-benzothiophene (66 μL, 0.5 mmol), **2** (162 μL, 1.25 mmol, 2.5 equiv), DTHQ (11.5 mg, 0.05 mmol, 10 mol %), Bu_4_NI (37 mg, 0.10 mmol, 20 mol %), and NaHCO_3_ (84 mg, 1.0 mmol, 2 equiv) in 3.3 mL of dry DMSO. The reaction mixture was irradiated with two Kessil 427 nm LEDs for 24 h at 55 °C and purified by flash column chromatography (0% → 10% EtOAc in Hex) to give the title compound as a colorless oil in 49% yield (62 mg): ^1^H NMR (400 MHz, CDCl_3_) δ 7.88–7.81 (m, 1H), 7.80–7.73 (m, 1H), 7.47–7.40 (m, 2H), 4.34 (q, *J* = 7.1 Hz, 2H), 2.53 (t, *J* = 2.1 Hz, 3H), 1.33 (t, *J* = 7.1 Hz, 3H); ^13^C{^1^H} NMR (201 MHz, CDCl_3_) δ 163.2 (t, *J* = 35.6 Hz), 140.1, 138.9, 128.2 (t, *J* = 28.4 Hz), 126.0, 124.6, 122.7, 122.5, 112.6 (t, *J* = 252.2 Hz), 63.5, 13.9, 12.1; ^19^F NMR (376 MHz, CDCl_3_) δ −94.38 (s, 2F); *R_f_* = 0.7 (4:1 Hex/EtOAc).

#### Ethyl 2,2-Difluoro-2-(1-phenyl-1*H*-pyrrol-2-yl)acetate (**10**)^29^

The title compound was prepared according to the general procedure from 1-phenylpyrrole (72 mg, 0.5 mmol, 1 equiv), **2** (162 μL, 1.25 mmol, 2.5 equiv), DTHQ (11.5 mg, 0.05 mmol, 10 mol %), Bu_4_NI (37 mg, 0.10 mmol, 20 mol %), and NaHCO_3_ (84 mg, 1.0 mmol, 2 equiv) in 3.3 mL of dry DMSO. The reaction mixture was irradiated with two Kessil 427 nm LEDs for 24 h at 55 °C and purified by flash column chromatography (0% → 8% EtOAc in Hex) to give the title compound as a colorless oil in 72% yield (95 mg): ^1^H NMR (400 MHz, CDCl_3_) δ 7.46–7.41 (m, 3H), 7.39–7.37 (m, 2H), 6.88–6.87 (m, 1H), 6.65–6.64 (m, 1H), 6.29 (t, *J* = 3.25 Hz, 1H), 4.14 (q, *J* = 7.2 Hz, 2H), 1.22 (t, *J* = 7.2 Hz, 3H); ^13^C{^1^H} NMR (201 MHz, CDCl_3_) δ 163.1 (t, *J* = 34.1 Hz), 139.3, 128.8, 128.4, 127.3, 126.9, 124.3 (t, *J* = 29.9 Hz), 113.0 (t, *J* = 5.0 Hz), 110.7 (t, *J* = 246.0 Hz), 108.4, 63.1, 13.8; ^19^F NMR (376 MHz, CDCl_3_) δ −92.22 (s, 2F); *R_f_* = 0.55 (4:1 Hex/EtOAc).

#### Ethyl 2,2-Difluoro-2-(1,3,7-trimethyl-2,6-dioxo-2,3,6,7-tetrahydro-1*H*-purin-8-yl)acetate (**11**)^26^

The title compound was prepared according to the general procedure from caffeine (97 mg, 0.5 mmol), **2** (162 μL, 1.25 mmol, 2.5 equiv), DTHQ (11.5 mg, 0.05 mmol, 10 mol %), Bu_4_NI (37 mg, 0.10 mmol, 20 mol %), and NaHCO_3_ (84 mg, 1.0 mmol, 2 equiv) in 3.3 mL of dry DMSO. The reaction mixture was irradiated with two Kessil 427 nm LEDs for 24 h at 55 °C and purified by flash column chromatography (0% → 35% EtOAc in Hex) to give the title compound as a white solid in 45% yield (71 mg): ^1^H NMR (400 MHz, CDCl_3_) δ 4.47 (q, *J* = 7.2 Hz, 2H), 4.18 (t, *J* = 1.5 Hz, 3H), 3.54 (s, 3H), 3.41 (s, 3H), 1.41 (t, *J* = 7.1 Hz, 1H); ^13^C{^1^H} NMR (201 MHz, CDCl_3_) δ 161.0 (t, *J* = 31.1 Hz), 155.5, 151.4, 146.7, 141.4 (t, *J* = 30.4 Hz), 109.6, 109.2 (t, *J* = 251.1 Hz), 64.1, 33.4–33.3 (m), 29.8, 28.1, 13.9; ^19^F NMR (376 MHz, CDCl_3_) δ −103.27 (s, 2F); *R_f_* = 0.68 (1:1 Hex/EtOAc).

#### (*R*)-2-[(*tert*-Butoxycarbonyl)amino]-3-[2-(2-ethoxy-1,1-difluoro-2-oxoethyl)-1*H*-indol-3-yl]propanoic Acid (**12**)

The title compound was prepared according to the general procedure from Boc-Trp-OH (152 mg, 0.5 mmol), **2** (162 μL, 1.25 mmol, 2.5 equiv), DTHQ (11.5 mg, 0.05 mmol, 10 mol %), Bu_4_NI (37 mg, 0.10 mmol, 20 mol %), and NaHCO_3_ (84 mg, 1.0 mmol, 2 equiv) in 3.3 mL of dry DMSO. The reaction mixture was irradiated with two Kessil 427 nm LEDs for 24 h at 55 °C and purified by flash column chromatography (0% → 10% EtOAc in Hex) to give the title compound as a white solid in 65% yield (138 mg): ^1^H NMR (800 MHz, DMSO-*d*_6_) δ 11.45 (s, 1H), 7.70 (d, *J* = 8.1 Hz, 1H), 7.41 (d, *J* = 8.2 Hz, 1H), 7.20 (t, *J* = 7.6 Hz, 1H), 7.06 (t, *J* = 7.5 Hz, 1H), 6.60 (bs, 1H), 4.34 (q, *J* = 7.1 Hz, 2H), 4.21–4.17 (m, 1H), 3.47 (q, *J* = 7.0 Hz, 1H), 3.31 (dd, *J* = 14.6, 6.1 Hz, 1H), 3.14–3.06 (m, 1H), 1.30–1.13 (m, 12H); ^13^C{^1^H} NMR (201 MHz, DMSO-*d*_6_) δ 173.6, 163.1 (t, *J* = 35.6 Hz), 155.5, 136.5, 128.3, 124.7 (t, *J* = 29.3 Hz), 123.8, 120.5, 119.9, 113.2, 112.5, 112.2, 78.5, 64.1, 56.5, 55.4, 28.5, 26.6, 14.1; ^19^F NMR (753 MHz, DMSO-*d*_6_) δ −97.20 to −99.26 (m, 2F); *R_f_* = 0.9 (4:1 Hex/EtOAc); HRMS (ESI) *m*/*z* calcd for C_20_H_24_F_2_N_2_O_6_H 427.1680, found 427.1680; IR (neat, cm^–1^) 3355, 2982, 2930, 1750, 1695, 1506, 1456, 1394, 1368, 1301, 1244, 1155, 1094, 1059, 1022, 909, 853, 741.

#### Ethyl 2-[3-(2-Acetamidoethyl)-5-methoxy-1*H*-indol-2-yl]-2,2-difluoroacetate (**13**)^30^

The title compound was prepared according to the general procedure from melatonin (116 mg, 0.5 mmol), **2** (162 μL, 1.25 mmol, 2.5 equiv), DTHQ (11.5 mg, 0.05 mmol, 10 mol %), Bu_4_NI (37 mg, 0.10 mmol, 20 mol %), and NaHCO_3_ (84 mg, 1.0 mmol, 2 equiv) in 3.3 mL of dry DMSO. The reaction mixture was irradiated with two Kessil 427 nm LEDs for 24 h at 55 °C and purified by flash column chromatography (0% → 20% EtOAc in Hex) to give the title compound as a colorless oil in 55% yield (89 mg): ^1^H NMR (400 MHz, CDCl_3_) δ 8.33 (bs, 1H), 7.29 (d, *J* = 9.0 Hz, 1H), 7.09 (d, *J* = 2.4 Hz, 1H), 6.97 (dd, *J* = 8.9, 2.4 Hz, 1H), 5.69 (bs, 1H), 4.35 (q, *J* = 7.2 Hz, 2H), 3.87 (s, 3H), 3.55 (q, *J* = 6.5 Hz, 2H), 3.08 (t, *J* = 6.7 Hz, 2H), 1.94 (s, 3H), 1.35 (t, *J* = 7.2 Hz, 3H); ^13^C{^1^H} NMR (201 MHz, CDCl_3_) δ 170.3, 163.5 (t, *J* = 35.9 Hz), 154.8, 130.8, 128.4, 124.5, 115.8, 114.9–114.8 (m), 112.6, 111.3, 100.6, 63.8, 55.9, 40.2, 29.7, 23.9, 23.3, 13.9; ^19^F NMR (376 MHz, CDCl_3_) δ −100.42; *R_f_* = 0.4 (4:1 Hex/EtOAc).

#### Ethyl 2,2-Difluoro-2-(2-oxo-2*H*-chromen-3-yl)acetate (**14**)^31^

The title compound was prepared according to the general procedure from coumarin (73 mg, 0.5 mmol), **2** (162 μL, 1.25 mmol, 2.5 equiv), DTHQ (11.5 mg, 0.05 mmol, 10 mol %), Bu_4_NI (37 mg, 0.10 mmol, 20 mol %), and NaHCO_3_ (84 mg, 1.0 mmol, 2 equiv) in 3.3 mL of dry DMSO. The reaction mixture was irradiated with two Kessil 427 nm LEDs for 24 h at 55 °C and purified by flash column chromatography (0% → 15% EtOAc in Hex) to give the title compound as a colorless oil in 46% yield (62 mg): ^1^H NMR (400 MHz, CDCl_3_) δ 8.16 (s, 1H), 7.68–7.60 (m, 2H), 7.42–7.43 (m, 2H), 4.39 (q, *J* = 7.1 Hz, 2H), 1.35 (t, *J* = 7.1 Hz, 3H); ^13^C{^1^H} NMR (201 MHz, CDCl_3_) δ 162.3 (t, *J* = 32.7 Hz), 158.0 (t, *J* = 4.4 Hz), 154.2, 142.0 (t, *J* = 6.8 Hz), 133.7, 129.3, 125.2, 121.1 (t, *J* = 25.6 Hz), 117.5, 117.0, 110.5 (t, *J* = 250.9 Hz), 63.6, 13.8; ^19^F NMR (376 MHz, CDCl_3_) δ −106.14; *R*_*f*_ = 0.39 (7:1 Hex/EtOAc).

#### Ethyl 2-(1,3-Dimethyl-2,4-dioxo-1,2,3,4-tetrahydropyrimidin-5-yl)-2,2-difluoroacetate (**15**)^26^

The title compound was prepared according to the general procedure from 1,3-dimethyluracil (70 mg, 0.5 mmol, 1 equiv), **2** (162 μL, 1.25 mmol, 2.5 equiv), DTHQ (11.5 mg, 0.05 mmol, 10 mol %), Bu_4_NI (37 mg, 0.10 mmol, 20 mol %), and NaHCO_3_ (84 mg, 1.0 mmol, 2 equiv) in 3.3 mL of dry DMSO. The reaction mixture was irradiated with two Kessil 427 nm LEDs for 24 h at 55 °C and purified by flash column chromatography (0% → 50% EtOAc in Hex) to give the title compound as a colorless oil in 54% yield (70 mg): ^1^H NMR (400 MHz, CDCl_3_) δ 7.65 (s, 1H), 4.35 (q, *J* = 7.2 Hz, 2H), 3.48 (s, 3H), 3.31 (s, 3H), 1.34 (t, *J* = 7.1 Hz, 3H); ^13^C{^1^H} NMR (201 MHz, CDCl_3_) δ 162.7 (t, *J* = 33.0 Hz), 160.3 (t, *J* = 4.1 Hz), 151.1, 142.6 (t, *J* = 8.1 Hz), 111.1 (t, *J* = 249.7 Hz), 107.1 (t, *J* = 25.2 Hz), 63.4, 37.7, 27.8, 13.8; ^19^F NMR (376 MHz, CDCl_3_) δ −103.67 (s, 2F); *R*_*f*_ = 0.39 (2:1 Hex/EtOAc).

#### Ethyl 2-(2,4-Dioxo-1,2,3,4-tetrahydropyrimidin-5-yl)-2,2-difluoroacetate (**16**)^31^

The title compound was prepared according to the general procedure from uracil (56 mg, 0.5 mmol), **2** (162 μL, 1.25 mmol, 2.5 equiv), DTHQ (11.5 mg, 0.05 mmol, 10 mol %), Bu_4_NI (37 mg, 0.10 mmol, 20 mol %), and NaHCO_3_ (84 mg, 1.0 mmol, 2 equiv) in 3.3 mL of dry DMSO. The reaction mixture was irradiated with two Kessil 427 nm LEDs for 24 h at 55 °C and purified by flash column chromatography (0% → 60% EtOAc in Hex) to give the title compound as a white solid in 43% yield (50 mg): ^1^H NMR (400 MHz, acetone-*d*_6_) δ 10.36 (bs, 2H), 7.98 (s, 1H), 4.32 (q, *J* = 7.1 Hz, 2H), 1.29 (t, *J* = 7.1 Hz, 3H); ^13^C{^1^H} NMR (201 MHz, acetone-*d*_6_) δ 162.3 (t, *J* = 33.6 Hz), 161.1 (t, *J* = 4.4 Hz), 150.1, 141.6 (t, *J* = 8.0 Hz), 111.6 (t, *J* = 246.5 Hz), 106.8 (t, *J* = 25.0 Hz), 62.7, 13.2; ^19^F NMR (376 MHz, acetone-*d*_6_) δ −104.67 (s, 2F); *R*_*f*_ = 0.23 (1:1 Hex/EtOAc).

#### Adamantan-1-yl 2,2-Difluoro-2-(1-phenyl-1*H*-pyrrol-2-yl)acetate (**17**)

The title compound was prepared according to the general procedure from 1-phenylpyrrole (72 mg, 0.5 mmol), adamantan-1-yl 2-bromo-2,2-difluoroacetate^23^ (387 mg, 1.25 mmol, 2.5 equiv), DTHQ (11.5 mg, 0.05 mmol, 10 mol %), Bu_4_NI (37 mg, 0.10 mmol, 20 mol %), and NaHCO_3_ (84 mg, 1.0 mmol, 2 equiv) in 3.3 mL of dry DMSO. The reaction mixture was irradiated with two Kessil 427 nm LEDs for 24 h at 55 °C and purified by flash column chromatography (0% → 7% EtOAc in Hex) to give the title compound as a colorless oil in 69% yield (128 mg): ^1^H NMR (400 MHz, CDCl_3_) δ 7.48–7.30 (m, 5H), 6.86- 6.85 (m, 1H), 6.63–6.61 (m, 1H), 6.26 (t, *J* = 3.3 Hz, 1H), 2.25–2.14 (m, 3H), 2.04 (d, *J* = 3.0 Hz, 6H), 1.64 (t, *J* = 3.0 Hz, 6H); ^13^C{^1^H} NMR (201 MHz, CDCl_3_) δ 161.5 (t, *J* = 33.0 Hz), 139.7, 128.8, 128.1, 127.1, 126.8, 124.5 (t, *J* = 29.3 Hz), 113.1 (t, *J* = 5.4 Hz), 110.5 (t, *J* = 246.5 Hz), 108.3, 84.7, 40.7, 35.8, 30.8; ^19^F NMR (376 MHz, CDCl_3_) δ −91.92 (s, 2F), −96.78; *R*_*f*_ = 0.81 (4:1 Hex/EtOAc); HRMS (ESI) *m*/*z* calcd for C_22_H_23_F_2_NO_2_H 372.1775, found 372.1776; IR (neat, cm^–1^) 2908, 2853, 1755, 1595, 1542, 1497, 1456, 1425, 1355, 1319, 1281, 1256, 1197, 1112, 1095, 1069, 1034, 962, 934, 875, 837, 804, 769, 730, 696, 602, 550, 509.

#### (1*S*,2*R*,5*S*)-2-Isopropyl-5-methylcyclohexyl 2,2-Difluoro-2-(1-phenyl-1*H*-pyrrol-2-yl)acetate (**18**)

The title compound was prepared according to the general procedure from 1-phenylpyrrole (179 mg, 1.25 mmol, 2.5 equiv), (1*S*,2*R*,5*S*)-2-isopropyl-5-methylcyclohexyl 2-bromo-2,2-difluoroacetate^23^ (111 μL, 0.5 mmol), DTHQ (11.5 mg, 0.05 mmol, 10 mol %), Bu_4_NI (37 mg, 0.10 mmol, 20 mol %), and NaHCO_3_ (84 mg, 1.0 mmol, 2 equiv) in 3.3 mL of dry DMSO. The reaction mixture was irradiated with two Kessil 427 nm LEDs for 24 h at 55 °C and purified by flash column chromatography (0% → 4% EtOAc in Hex) to give the title compound as a colorless oil in 51% yield (97 mg): ^1^H NMR (400 MHz, CDCl_3_) δ 7.48–7.31 (m, 5H), 6.88–6.86 (m, 1H), 6.60–6.57 (m, 1H), 6.27 (t, *J* = 3.3 Hz, 1H), 4.73 (td, *J* = 10.9, 4.4 Hz, 1H), 1.89–1.84 (m, 1H), 1.78–1.74 (m, 1H), 1.72–1.61 (m, 2H), 1.48–1.38 (m, 2H), 1.11–0.91 (m, 2H), 0.91 (d, *J* = 6.5 Hz, 3H), 0.87–0.81 (m, 1H), 0.84 (d, *J* = 7.0 Hz, 3H), 0.71 (d, *J* = 7.0 Hz, 3H); ^13^C{^1^H} NMR (201 MHz, CDCl_3_) δ 162.8 (t, *J* = 33.7 Hz), 139.6 129.7, 128.8, 128.2, 127.4, 126.9, 124.1 (t, *J* = 29.0 Hz), 120.9, 120.5, 113.2 (t, *J* = 5.2 Hz), 110.9 (t, *J* = 246.5), 108.4, 77.8, 46.6, 40.0, 34.0, 31.3, 25.9, 23.2, 21.9, 20.6, 16.1; ^19^F NMR (376 MHz, CDCl_3_) δ −91.74 (s, 2F); *R*_*f*_ = 0.81 (4:1 Hex/EtOAc); HRMS (ESI) *m*/*z* [M + H]^+^ calcd for C_22_H_27_F_2_NO_2_H 376.2088, found 376.2083; IR (neat, cm^–1^) 2955, 2927, 2872, 1753, 1598, 1499, 1456, 1295, 1258, 1199, 1112, 1091, 1069, 1035, 1022, 950, 910, 765, 727, 693.

#### 2,2-Difluoro-2-(1-phenyl-1*H*-pyrrol-2-yl)-1-(piperidin-1-yl)ethan-1-one (**19**)

The title compound was prepared according to the general procedure from 1-phenylpyrrole (72 mg, 0.5 mmol), 2-bromo-2,2-difluoro-1-(piperidin-1-yl)ethan-1-one^24a^ (195 μL, 1.25 mmol, 2.5 equiv), DTHQ (11.5 mg, 0.05 mmol, 10 mol %), Bu_4_NI (37 mg, 0.10 mmol, 20 mol %), and NaHCO_3_ (84 mg, 1.0 mmol, 2 equiv) in 3.3 mL of dry DMSO. The reaction mixture was irradiated with two Kessil 427 nm LEDs for 24 h at 55 °C and purified by flash column chromatography (0% → 20% EtOAc in Hex) to give the title compound as a white solid in 70% yield (107 mg): ^1^H NMR (400 MHz, CDCl_3_) δ 7.45–7.37 (m, 5H), 6.88–6.85 (m, 1H), 6.56–6.52 (m, 1H), 6.25 (t, *J* = 3.30 Hz, 1H), 3.49 (t, *J* = 5.4 Hz, 2H), 3.33 (t, *J* = 5.6 Hz, 2H), 1.69–1.45 (m, 4H), 1.46–1.36 (m, 2H); ^13^C{^1^H} NMR (201 MHz, CDCl_3_) δ 160.9 (t, *J* = 28.8 Hz), 139.5, 128.8, 128.3, 127.2, 126.8, 125.1 (t, *J* = 28.1 Hz), 112.9 (t, *J* = 5.4 Hz), 112.2 (t, *J* = 243.1 Hz), 108.4, 47.4–47.4 (m), 44.3, 25.9, 25.4, 24.3; ^19^F NMR (376 MHz, CDCl_3_) δ −85.56 (s, 2F); *R*_*f*_ = 0.40 (4:1 Hex/EtOAc); HRMS (ESI) *m*/*z* [M + H]^+^ calcd for C_17_H_18_F_2_N_2_OH 305.1465, found 305.1461; IR (neat, cm^–1^) 2938, 2858, 1659, 1597, 1542, 1498, 1459, 1450, 1442, 1419, 1350, 1322, 1308, 1262, 1202, 1149, 1105, 1093, 1059, 1025, 1002, 938, 856, 765, 697, 607.

#### *N,N*-Diethyl-2,2-difluoro-2-(1-phenyl-1*H*-pyrrol-2-yl)acetamide (**20**)

The title compound was prepared according to the general procedure from 1-phenylpyrrole (72 mg, 0.5 mmol), 2-bromo-*N,N*-diethyl-2,2-acetamide^24b^ (184 μL, 1.25 mmol, 2.5 equiv), DTHQ (11.5 mg, 0.05 mmol, 10 mol %), Bu_4_NI (37 mg, 0.10 mmol, 20 mol %), and NaHCO_3_ (84 mg, 1.0 mmol, 2 equiv) in 3.3 mL of dry DMSO. The reaction mixture was irradiated with two Kessil 427 nm LEDs for 24 h at 55 °C and purified by flash column chromatography (0% → 20% EtOAc in Hex) to give a 14:1 regioisomeric mixture of the title compound as a colorless oil in 51% yield (75 mg): ^1^H NMR (400 MHz, CDCl_3_) δ 7.42–7.39 (m, 5H), 6.87–6.85 (m, 1H), 6.51–6.49 (m, 1H), 6.25 (t, *J* = 3.30 Hz, 1H), 3.37–3.21 (m, 1H), 1.09 (t, *J* = 7.1 Hz, 1H), 1.02 (t, *J* = 7.0 Hz, 1H); ^13^C{^1^H} NMR (201 MHz, CDCl_3_) δ 161.8 (t, *J* = 29.0 Hz), 139.5, 128.7, 128.2, 127.2, 126.9, 125.2 (t, *J* = 28.4 Hz), 112.7 (t, *J* = 5.4 Hz), 112.3 (t, *J* = 244.0 Hz), 108.4, 42.6 (t, *J* = 3.3 Hz), 41.4, 30.2, 29.3, 13.7, 12.0; ^19^F NMR (376 MHz, CDCl_3_) δ −86.57 (s, 2F); *R*_*f*_ = 0.40 (4:1 Hex/EtOAc); HRMS (ESI) *m*/*z* calcd for C_16_H_18_F_2_N_2_OH 293.1465, found 293.1461; IR (neat, cm^–1^) 2975, 2933, 1668, 1598, 1541, 1499, 1464, 1425, 1382, 1321, 1283, 1257, 1203, 1170, 1103, 1087, 1059, 1037, 928, 850, 767, 732, 680.

#### *N*-Benzyl-2,2-difluoro-2-(1-phenyl-1*H*-pyrrol-2-yl)acetamide (**21**)

The title compound was prepared according to the general procedure from 1-phenylpyrrole (72 mg, 0.5 mmol), *N*-benzyl-2,bromo-2,2-difluoroacetamide^24c^ (330 mg, 1.25 mmol, 2.5 equiv), DTHQ (11.5 mg, 0.05 mmol, 10 mol %), Bu_4_NI (37 mg, 0.10 mmol, 20 mol %), and NaHCO_3_ (84 mg, 1.0 mmol, 2 equiv) in 3.3 mL of dry DMSO. The reaction mixture was irradiated with two Kessil 427 nm LEDs for 24 h at 55 °C and purified by flash column chromatography (0% → 25% EtOAc in Hex) to give the title compound as a white solid in 70% yield (114 mg): ^1^H NMR (400 MHz, CDCl_3_) δ 7.43–7.34 (m, 5H), 7.36–7.28 (m, 3H), 7.24–7.15 (m, 2H), 6.86–6.84 (m, 1H), 6.69–6.67 (m, 1H), 6.35 (s, 1H), 6.27 (t, *J* = 3.25 Hz, 1H), 4.35 (d, *J* = 5.7 Hz, 1H); ^13^C{^1^H} NMR (201 MHz, CDCl_3_) δ 162.9 (t, *J* = 30.0 Hz), 139.5, 136.5, 128.82, 128.75, 127.9, 127.5, 127.2, 126.1, 124.2 (t, *J* = 29.3 Hz), 113.7 (t, *J* = 5.2 Hz), 112.4 (t, *J* = 247.4 Hz), 108.4, 43.7; ^19^F NMR (376 MHz, CDCl_3_) −92.50 (s, 2F); *R*_*f*_ = 0.37 (4:1 Hex/EtOAc); HRMS (ESI) *m*/*z* [M + H]^+^ calcd for C_19_H_16_F_2_N_2_OH 327.1309, found 327.1306; IR (neat, cm^–1^) 3224, 3090, 2933, 1706, 1683, 1564, 1538, 1497, 1456, 1424, 1351, 1303, 1263, 1226, 1200, 1127, 1062, 1045, 967, 926, 912, 811, 756, 696, 602, 543.

#### 2,2-Difluoro-2-(1-phenyl-1*H*-pyrrol-2-yl)acetamide (**22**)

The title compound was prepared according to the general procedure from 1-phenylpyrrole (72 mg, 0.5 mmol), 2-bromo-2,2-difluoroacetamide (217 mg, 1.25 mmol, 2.5 equiv), DTHQ (11.5 mg, 0.05 mmol, 10 mol %), Bu_4_NI (37 mg, 0.10 mmol, 20 mol %), and NaHCO_3_ (84 mg, 1.0 mmol, 2 equiv) in 3.3 mL of dry DMSO. The reaction mixture was irradiated with two Kessil 427 nm LEDs for 24 h at 55 °C and purified by flash column chromatography (0% → 60% EtOAc in Hex) to give the title compound as a colorless oil in 48% yield (56 mg): ^1^H NMR (400 MHz, CDCl_3_) δ 7.44–7.37 (m, 5H), 6.88–6.87 (m, 1H), 6.71–6.69 (m, 1H), 6.27 (t, *J* = 3.3 Hz, 1H), 6.08–5.92 (m, 2H); ^13^C{^1^H} NMR (201 MHz, CDCl_3_) δ 165.3 (t, *J* = 30.8 Hz), 139.4, 128.8, 128.5, 127.7, 127.2, 123.9 (t, *J* = 28.9 Hz), 121.0, 113.7 (t, *J* = 5.3 Hz), 112.1 (t, *J* = 247.3 Hz), 108.4; ^19^F NMR (376 MHz, CDCl_3_) δ −92.48 (s, 2F); *R*_*f*_ = 0.25 (4:1 Hex/EtOAc); HRMS (ESI) *m*/*z* calcd for C_12_H_10_F_2_N_2_OH 237.0839, found 237.0839; IR (neat, cm^–1^) 3450, 3310, 3192, 1688, 1613, 1596, 1540, 1500, 1459, 1412, 1356, 1319, 1309, 1261, 1204, 1174, 1108, 1090, 1071.

#### 2-(Perfluorobutyl)-1-phenyl-1*H*-pyrrole (**23**)^32^

The title compound was prepared according to the general procedure from 1-phenylpyrrole (72 mg, 0.5 mmol), perfluorobutyl bromide (194 μL, 1.25 mmol, 2.5 equiv), DTHQ (11.5 mg, 0.05 mmol, 10 mol %), Bu_4_NI (37 mg, 0.10 mmol, 20 mol %), and NaHCO_3_ (84 mg, 1.0 mmol, 2 equiv) in 3.3 mL of dry DMSO. The reaction mixture was irradiated with two Kessil 427 nm LEDs for 24 h at 55 °C and purified by flash column chromatography (0% → 5% EtOAc in Hex) to give the title compound as a colorless oil in 67% yield (124 mg): ^1^H NMR (400 MHz, CDCl_3_) δ 7.44–7.36 (m, 3H), 7.31–7.21 (m, 1H), 7.10 (t, *J* = 2.2 Hz, 2H), 6.36 (t, *J* = 2.2 Hz, 2H); ^13^C{^1^H} NMR (201 MHz, CDCl_3_) δ 140.9, 129.6, 125.6, 120.6, 119.4, 118.2–112.9 (m), 110.4, 110.1–106.6 (m); ^19^F NMR (376 MHz, CDCl_3_) δ −81.07 (t, *J* = 10.1 Hz, 3F), −101.12 (t, *J* = 13.5 Hz, 2F), −121.25 to −121.62 (m, 2F), −125.85 (m, 2F); *R*_*f*_ = 0.89 (4:1 Hex/EtOAc).

#### 2-[Difluoro(1-phenyl-1*H*-pyrrol-2-yl)methyl]benzo[*d*]oxazole (**24**)

The title compound was prepared according to the general procedure from 1-phenylpyrrole (72 mg, 0.5 mmol), 2-(bromodifluoromethyl)-1,3-benzoxazole (253 μL, 1.25 mmol, 2.5 equiv), DTHQ (11.5 mg, 0.05 mmol, 10 mol %), Bu_4_NI (37 mg, 0.10 mmol, 20 mol %), and NaHCO_3_ (84 mg, 1.0 mmol, 2 equiv) in 3.3 mL of dry DMSO. The reaction mixture was irradiated with two Kessil 427 nm LEDs for 24 h at 55 °C and purified by flash column chromatography (0% → 15% EtOAc in Hex) to give the title compound as a white solid in quantitative yield (170 mg): ^1^H NMR (400 MHz, CDCl_3_) δ 7.75–7.72 (m, 1H), 7.53–7.51 (m, 1H), 7.45–7.36 (m, 2H), 7.30–7.23 (m, 5H), 6.92–6.91 (m, 1H), 6.70–6.68 (m, 1H), 6.33 (t, *J* = 3.25 Hz, 1H); ^13^C{^1^H} NMR (201 MHz, CDCl_3_) δ 157.7 (t, *J* = 35.6 Hz), 150.4, 140.0, 139.1, 128.6, 128.3, 127.6, 127.0, 126.7, 125.1, 121.3, 113.3 (t, *J* = 4.6 Hz), 111.4 (t, *J* = 238.1 Hz), 111.2, 108.4; ^19^F NMR (376 MHz, CDCl_3_) δ −85.32 (s, 2F); *R*_*f*_ = 0.40 (4:1 Hex/EtOAc); HRMS (ESI) *m*/*z* calcd for C_18_H_12_F_2_N_2_OH 311.0999, found 311.0996; IR (neat, cm^–1^) 3106, 3068, 1618, 1595, 1541, 1499, 1450, 1427, 1362, 1347, 1320, 1297, 1260, 1240, 1204, 1174, 1101, 1056, 1029, 1017, 948, 906, 876, 774, 734, 698, 545.

## Data Availability

The data underlying this study are available in the published article and its Supporting Information.

## Supporting Information Available

Experimental procedures, reaction optimization, control reactions, UV–visible studies, computational studies, and NMR spectra (PDF)
