# Supplementary material for: A Dual Catalytic Approach for the Halogen-Bonding-Mediated Reductive Cleavage of α-Bromodifluoroesters and Amides
Source: J Org Chem. 2024 Dec 19;90(1):863–71. doi: 10.1021/acs.joc.4c02413 (PMC11731275; doi:10.1021/acs.joc.4c02413)

Supporting Information

A Dual Catalytic Approach for the Halogen-Bonding-Mediated Reductive Cleavage of α-Bromodifluoroesters and Amides

Tarannum Tasnim, Negin Shafiei, Katelyn J. Laminack, Bailey S. Robertson, Nash E. Nevels, Christopher J. Fennell and Spencer P. Pitre*

Department of Chemistry, Oklahoma State University, 107 Physical Sciences, Stillwater, OK 74078, United States

Email: spencer.p.pitre@okstate.edu

Table of Contents

A. Photochemistry Set-Up S2
B. Full Reaction Optimization S3
C. Procedure for 1 mmol Scale Radical Perfluoroalkylation Reaction S5
D. Procedure for Control Reaction with TEMPO S6
E. Control Reactions without $Bu_4N^+I^-$ S8
F. Low Yielding Scope Examples S14
G. UV-Vis Studies S15
H. Initial Rate Experiments S17
I. Sensitive Functional Group Screen S18
J. Finkelstein Displacement Studies S27
K. Computational Studies S30
K.1. *Thermodynamic Stability of Difluoroalkyl Complexes* S30
K.2. *Exploration of alternative mechanisms to Finkelstein reaction* S33
K.3. *Absolute activation energies for Finkelstein reaction conversions as a function of substrate* S34
K.4. *Structural scan of reagents susceptible to Finkelstein S_N2 conversion from bromide to iodide form* S35
L. References S69
M. NMR Spectra S70

A. Photochemistry Setup

All photochemistry experiments were performed using two Kessil PR-160L 427nm LEDs (40 W output at 100% intensity) placed 2.5 cm from the reaction vessels equipped with an overhead fan to maintain the temperature at approximately 55 °C.

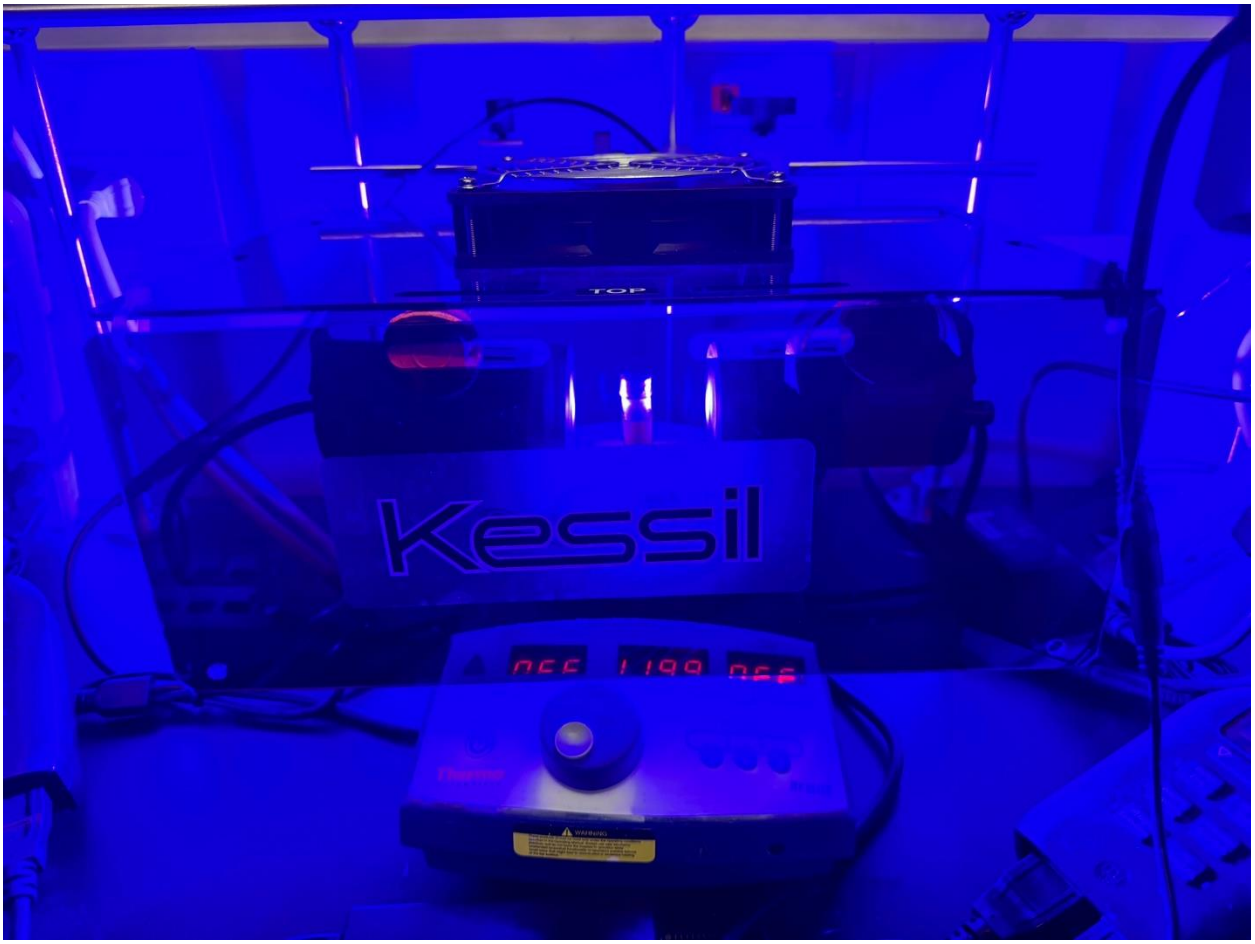

Figure S1. Picture of the photochemistry set-up employed in this work.

B. Full Reaction Optimization

Table S1. Optimization and control reactions for the *gem*-difluoroalkylation of 1,3,5-trimethoxybenzene (**1**).

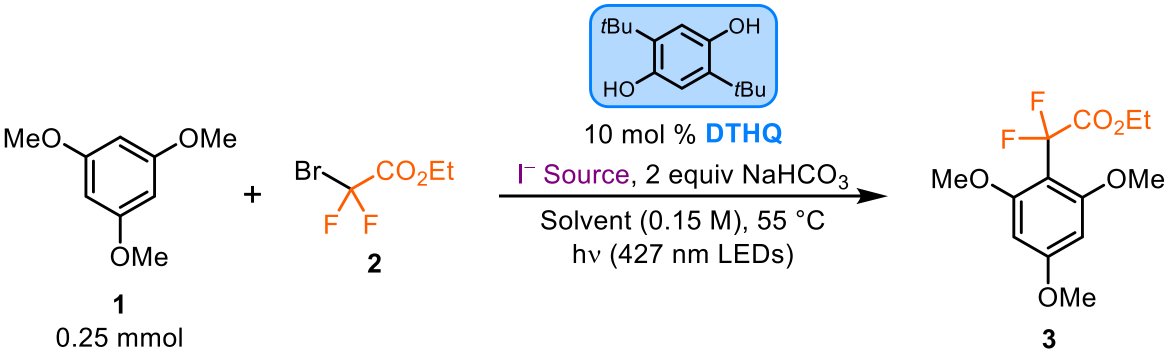

I^- (mol%)	DTHQ (mol%)	equiv H_2O	equiv 2	Solvent (Conc.)	Time	Yield 3[a]
NaI (5)	10	None	3	11:1 MeCN:MeOH (0.15 M)	30 h	Trace
NaI (10)	10	None	3	11:1 MeCN:MeOH (0.15 M)	30 h	Trace
NaI (20)	10	None	3	11:1 MeCN:MeOH (0.15 M)	30 h	Trace
NaI (100)	10	None	3	11:1 MeCN:MeOH (0.15 M)	30 h	24%
NaI (100)	10	None	3	11:1 Acetone: H_2O (0.15 M)	30 h	24%
$Bu_4N^+I^-$ (100)	10	None	3	11:1 Acetone: H_2O (0.15 M)	30 h	48%
$Bu_4N^+I^-$ (100)	10	None	3	Acetone (0.15 M)	30 h	91%
$Bu_4N^+I^-$ (100)	10	None	3	Acetonitrile (0.15 M)	30 h	18%
$Bu_4N^+I^-$ (100)	10	None	3	Methanol (0.15 M)	30 h	None
$Bu_4N^+I^-$ (100)	10	None	3	THF (0.15 M)	30 h	65%
$Bu_4N^+I^-$ (100)	10	None	3	DMF (0.15 M)	30 h	73%
$Bu_4N^+I^-$ (100)	10	None	3	DMSO (0.15 M)	30 h	Quant.
$Bu_4N^+I^-$ (75)	10	None	3	Acetone (0.15 M)	30 h	7%
$Bu_4N^+I^-$ (50)	10	None	3	Acetone (0.15 M)	30 h	66%
$Bu_4N^+I^-$ (25)	10	None	3	Acetone (0.15 M)	30 h	51%
$Bu_4N^+I^-$ (10)	10	None	3	Acetone (0.15 M)	30 h	77%
$Bu_4N^+I^-$ (5)	10	None	3	Acetone (0.15 M)	30 h	74%
$Bu_4N^+I^-$ (10)	10	None	3	Acetone (0.15 M)	6 h	Trace
$Bu_4N^+I^-$ (10)	10	None	3	Acetone (0.15 M)	12 h	35%
$Bu_4N^+I^-$ (10)	10	None	3	Acetone (0.15 M)	18 h	24%
$Bu_4N^+I^-$ (10)	10	None	3	Acetone (0.15 M)	24 h	59%
$Bu_4N^+I^-$ (20)	10	None	3	Dry Acetone (0.15 M)	30 h	20%
$Bu_4N^+I^-$ (10)	10	None	3	Dry Acetone (0.15 M)	30 h	Trace
$Bu_4N^+I^-$ (10)	10	1	3	Dry Acetone (0.15 M)	30 h	9%
$Bu_4N^+I^-$ (10)	10	5	3	Dry Acetone (0.15 M)	30 h	30%
$Bu_4N^+I^-$ (10)	10	10	2	Dry Acetone (0.15 M)	30 h	32%
$Bu_4N^+I^-$ (10)	10	20	1	Dry Acetone (0.15 M)	30 h	9%
$Bu_4N^+I^-$ (10)	10	50	3	Dry Acetone (0.15 M)	30 h	6%
$Bu_4N^+I^-$ (10)	10	100	3	Dry Acetone (0.15 M)	30 h	Trace
$Bu_4N^+I^-$ (100)	10	None	3	Dry Acetone (0.15 M)	30 h	62%
$Bu_4N^+I^-$ (100)	10	1	3	Dry Acetone (0.15 M)	30 h	72%
$Bu_4N^+I^-$ (100)	10	5	3	Dry Acetone (0.15 M)	30 h	72%
$Bu_4N^+I^-$ (100)	10	6	3	Dry Acetone (0.15 M)	30 h	55%
$Bu_4N^+I^-$ (100)	10	8	3	Dry Acetone (0.15 M)	30 h	54%
$Bu_4N^+I^-$ (100)	10	10	3	Dry Acetone (0.15 M)	30 h	66%

$Bu_4N^+I^-$ (100)	10	20	3	Dry Acetone (0.15 M)	30 h	51%
$Bu_4N^+I^-$ (100)	10	50	3	Dry Acetone (0.15 M)	30 h	16%
$Bu_4N^+I^-$ (100)	10	100	3	Dry Acetone (0.15 M)	30 h	8%
$Bu_4N^+I^-$ (100)	10	None	3	DMSO (0.15 M)	30 h	Quant.
$Bu_4N^+I^-$ (75)	10	None	3	DMSO (0.15 M)	30 h	Quant.
$Bu_4N^+I^-$ (60)	10	None	3	DMSO (0.15 M)	30 h	91%
$Bu_4N^+I^-$ (50)	10	None	3	DMSO (0.15 M)	30 h	Quant.
$Bu_4N^+I^-$ (40)	10	None	3	DMSO (0.15 M)	30 h	90%
$Bu_4N^+I^-$ (30)	10	None	3	DMSO (0.15 M)	30 h	97%
$Bu_4N^+I^-$ (20)	10	None	3	DMSO (0.15 M)	30 h	79%
$Bu_4N^+I^-$ (10)	10	None	3	DMSO (0.15 M)	30 h	6%
$Bu_4N^+I^-$ (20)	10	None	2.5	DMSO (0.15 M)	30 h	72%
$Bu_4N^+I^-$ (20)	10	None	2	DMSO (0.15 M)	30 h	60%
$Bu_4N^+I^-$ (20)	10	None	1.5	DMSO (0.15 M)	30 h	49%
$Bu_4N^+I^-$ (20)	10	None	1	DMSO (0.15 M)	30 h	26%
$Bu_4N^+I^-$ (20)	10	None	2.5	DMSO (0.15 M)	6 h	54%
$Bu_4N^+I^-$ (20)	10	None	2.5	DMSO (0.15 M)	12 h	58%
$Bu_4N^+I^-$ (20)	10	None	2.5	DMSO (0.15 M)	18 h	60%
$Bu_4N^+I^-$ (20)	10	None	2.5	DMSO (0.15 M)	24 h	87%
None	10	None	2.5	DMSO (0.15 M)	24 h	31%
$Bu_4N^+I^-$ (20)	None	None	2.5	DMSO (0.15 M)	24 h	11%
$Bu_4N^+I^-$ (20)	None	None	2.5	DMSO (0.15 M)	24 h	10%[b]
$Bu_4N^+I^-$ (20)	10	None	2.5	DMSO (0.15 M)	24 h	26%[c]
$Bu_4N^+I^-$ (20)	10	None	2.5	DMSO (0.15 M)	24 h	22%[d]
$Bu_4N^+I^-$ (20)	10	None	2.5	DMSO (0.15 M)	24 h	Trace[e]
$Bu_4N^+I^-$ (20)	10	None	2.5	DMSO (0.15 M)	24 h	10%[f]
$Bu_4N^+I^-$ (20)	10	None	2.5	DMSO (0.15 M)	24 h	29%[g]

[a]Yields were determined by ^{19}F NMR using hexafluorobenzene as an external standard. [b]1,4-di-*tert*-butyl-2,5-dimethoxybenzene instead of DTHQ. [c]Reaction carried out without base. [d]No degassing with Ar. [e]No light. [f]1,4-di-*tert*-butyl-2,5-dimethoxybenzene instead of DTHQ. [g]At room temperature.

C. Procedure for 1 mmol Scale Reaction

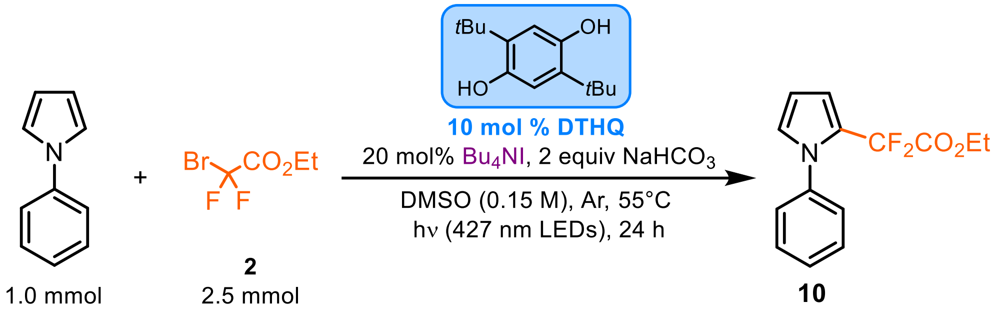

An oven-dried 25 mL flask equipped with a magnetic stir bar was charged with a 1-phenylpyrrole (143 mg, 1.0 mmol, 1 equiv), ethyl bromodifluoroacetate (327 µL, 32.5 mmol, 2.5 equiv), 2,5-di-*tert*-butylhydroquinone (22.2 mg, 0.1 mmol, 10 mol%), tetra-*N*-butyl ammonium iodide (74 mg, 0.20 mmol, 20 mol%) and $NaHCO_3$ (168 mg, 2.0 mmol, 2 equiv). DMSO was added (6.7 mL, 0.15 M), and the reaction mixture was degassed by sparging with argon for 5-6 minutes. The reaction mixture was then sonicated and irradiated with two Kessil 427 nm LED lamps for 24 h at approximately 55 °C. The reaction mixture was transferred into a separatory funnel and diluted with 20 mL of DCM and washed with 20 mL of 10 mM $Na_2S_2O_3$ (aq). The aqueous phase was extracted with 20 mL of DCM. The combined organic phases were dried with $MgSO_4$ and concentrated. Purified by flash column chromatography (0 →5% EtOAc in Hex) to give the desired product as a colorless oil in quantitative yield (136 mg). Spectral data matched that previously reported.

D. Procedure for Control Reaction with TEMPO

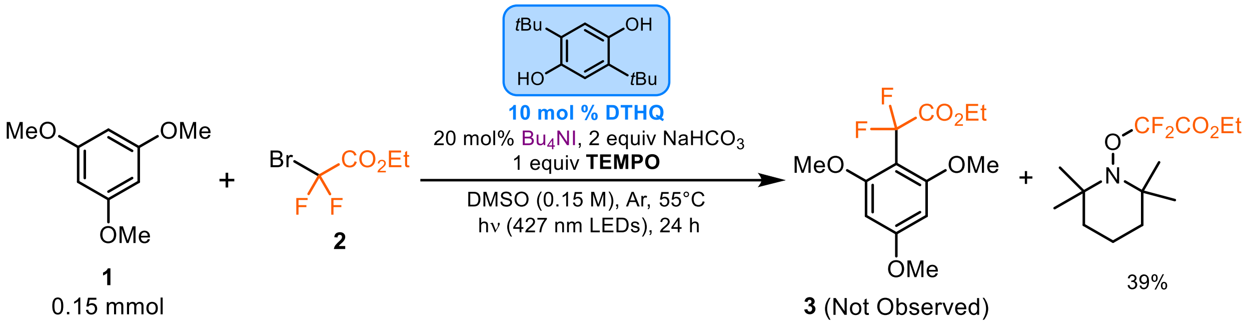

An oven-dried two-dram borosilicate glass vial equipped with a magnetic stir bar was charged with 1,3,5-trimethoxybenzene (25 mg, 0.15 mmol, 1 equiv), ethyl bromodifluoroacetate (49 µL, 0.375 mmol, 2.5 equiv), 2,5-di-*tert*-butylhydroquinone (7.0 mg, 0.03 mmol, 10 mol%) tetra-*N*-butyl ammonium iodide (13 mg, 0.030 mmol, 20 mol%), TEMPO (24 mg, 0.15 mmol, 1 equiv.) and $NaHCO_3$ (168 mg, 2.0 mmol, 2 equiv). DMSO was added (6.7 mL, 0.15 M), and the reaction mixture was degassed by sparging with argon for 5-6 minutes. The reaction mixture was then sonicated and irradiated with two Kessil 427 nm LED lamps for 24 h at approximately 55 °C. The reaction mixture was transferred into a separatory funnel and diluted with 15 mL of DCM and washed with 15 mL of 10 mM $Na_2S_2O_3$ (aq). The aqueous phase was extracted with 15 mL of DCM. The combined organic phases were dried with $MgSO_4$ and concentrated. Yield was calculated from ^{19}F NMR using hexafluorobenzene (16.0 mg) as an external standard (see Figure S2). The calculation showed no desired product (**3**) formation after 24 h. The yield for the TEMPO adduct was calculated as 39%.

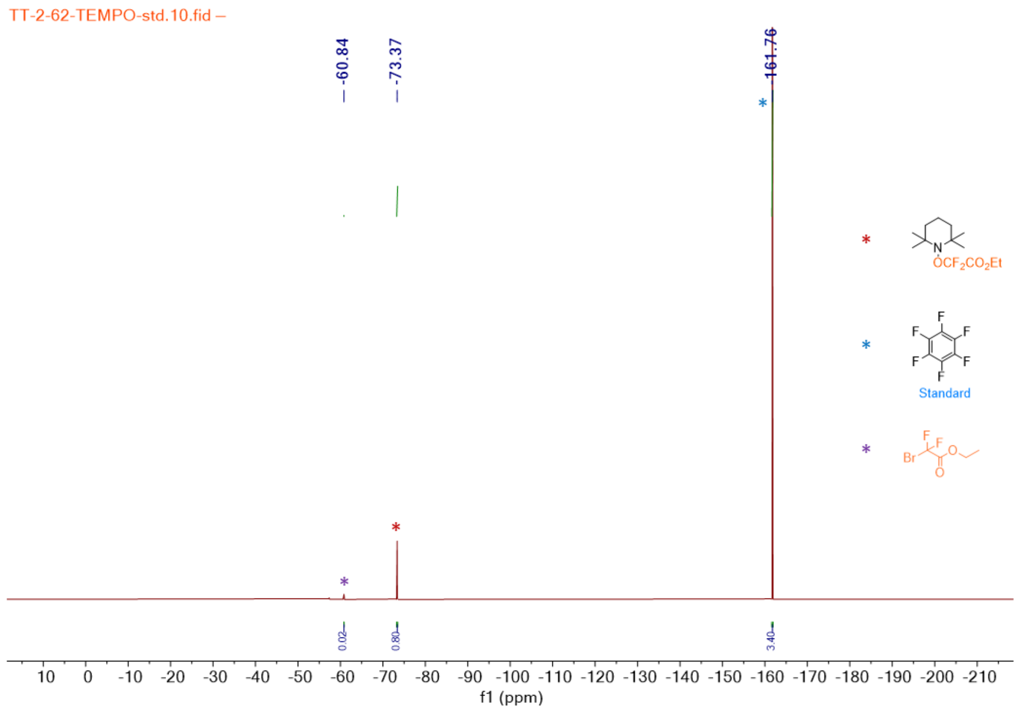

Figure S2. ^{19}F NMR spectrum of the crude material for the control reaction with TEMPO.

E. Control Reactions without $Bu_4N^+I^-$

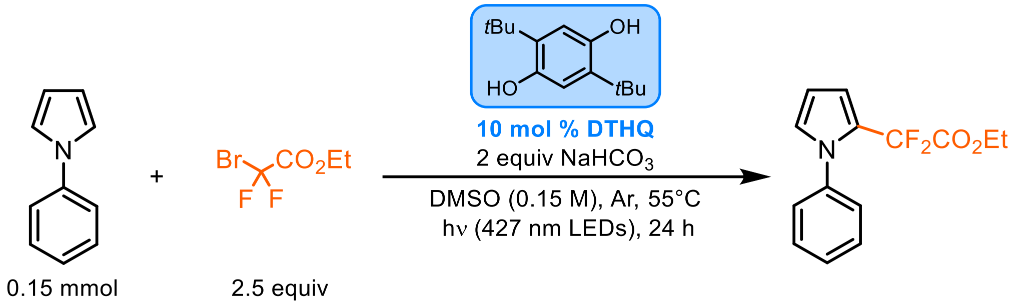

General Procedure: An oven-dried one-dram borosilicate glass vial equipped with a magnetic stir bar was charged with a (hetero)-arene (0.15 mmol, 1 equiv), 2,5-di-*tert*-butylhydroquinone (3.5 mg, 0.015 mmol, 10 mol %) and $NaHCO_3$ (25.0 mg, 0.30 mmol, 2 equiv). DMSO was added (1 mL, 0.15 M), and the reaction mixture was degassed by sparging with argon for 5-6 minutes. To this, perfluoroalkyl bromide (0.375 mmol, 2.5 equiv) was added under Argon. The reaction mixture was then sonicated and irradiated with two Kessil 427nm LED lamps for 24 h at approximately 55 °C. The reaction mixture was transferred into a separatory funnel, diluted with 15 mL of DCM and washed with 15 mL of 10 mM $Na_2S_2O_3$ (aq). The aqueous phase was extracted with 15 mL of DCM. The combined organic phases were dried with $MgSO_4$ and concentrated. Yields were calculated using ^{19}F NMR using hexafluorobenzene as an external standard.

Ethyl 2,2-difluoro-2-(2,4,6-trimethoxyphenyl)acetate (3): Control reaction was carried out according to the general procedure with 1,3,5-trimethoxybenzene (25 mg, 0.15 mmol, 1 equiv), ethyl bromodifluoroacetate (49 µL, 0.375 mmol, 2.5 equiv), 2,5-di-*tert*-butylhydroquinone (3.5 mg, 0.015 mmol, 10 mol%) and $NaHCO_3$ (25.0 mg, 0.30 mmol, 2 equiv) in 1 mL of dry DMSO. The reaction was irradiated with two Kessil 427 nm LEDs for 24 h at 55 °C to give the desired product **3** in 31% by ^{19}F NMR yield using C_6F_6 as an external standard.

Ethyl 2-(benzo[*d*][1,3]dioxol-5-yl)-2,2-difluoroacetate (4): Control reaction was carried out according to the general procedure with 1,2-methylene dixoybenzene (18 µL, 0.15 mmol, 1 equiv), ethyl bromodifluoroacetate (49 µL, 0.375 mmol, 2.5 equiv), 2,5-di-*tert*-butylhydroquinone (3.5 mg, 0.015 mmol, 10 mol%) and $NaHCO_3$ (25.0 mg, 0.30 mmol, 2 equiv) in 1 mL of dry DMSO.

The reaction was irradiated with two Kessil 427 nm LEDs for 24 h at 55 °C to give the desired product **4** in 24% by ^{19}F NMR yield using C_6F_6 as an external standard.

Ethyl 2-(5-(*tert*-butyl)-2-methoxyphenyl)-2,2 difluoroacetate (5): Control reaction was carried out according to the general procedure with 1-(*tert*-butyl)-4-methoxybenzene (27 µL, 0.15 mmol, 1 equiv), ethyl bromodifluoroacetate (49 µL, 0.375 mmol, 2.5 equiv), 2,5-di-*tert*-butylhydroquinone (3.5 mg, 0.015 mmol, 10 mol%) and $NaHCO_3$ (25.0 mg, 0.30 mmol, 2 equiv) in 1 mL of dry DMSO. The reaction was irradiated with two Kessil 427 nm LEDs for 24 h at 55 °C and no desired product **5** was detected from ^{19}F NMR.

Ethyl 2,2-difluoro-2-(4-methoxy-2,5-dimethylphenyl)acetate (6): Control reaction was carried out according to the general procedure with 2-methoxy-1,4-dimethylbenzene (21 µL, 0.15 mmol, 1 equiv), ethyl bromodifluoroacetate (49 µL, 0.375 mmol, 2.5 equiv), 2,5-di-*tert*-butylhydroquinone (3.5 mg, 0.015 mmol, 10 mol%) and $NaHCO_3$ (25.0 mg, 0.30 mmol, 2 equiv) in 1 mL of dry DMSO. The reaction was irradiated with two Kessil 427 nm LEDs for 24 h at 55 °C to give the desired product **6** in 23% by ^{19}F NMR using C_6F_6 as an external standard.

Ethyl 2-(2,6-dimethoxypyridin-3-yl)-2,2-difluoroacetate (7): Control reaction was carried out according to the general procedure with 2,6-dimethyloxypyridine (22 µL, 0.15 mmol, 1 equiv), ethyl bromodifluoroacetate (49 µL, 0.375 mmol, 2.5 equiv), 2,5-di-*tert*-butylhydroquinone (3.5 mg, 0.015 mmol, 10 mol%) and $NaHCO_3$ (25.0 mg, 0.30 mmol, 2 equiv) in 1 mL of dry DMSO. The reaction was irradiated with two Kessil 427 nm LEDs for 24 h at 55 °C to give the desired product **7** in 18% by ^{19}F NMR using C_6F_6 as an external standard.

Ethyl 2,2-difluoro-2-(3-methylbenzofuran-2-yl)acetate (8): Control reaction was carried out according to the general procedure with 3-methylbenzofuran (19 µL, 0.15 mmol, 1 equiv), ethyl bromodifluoroacetate (49 µL, 0.375 mmol, 2.5 equiv), 2,5-di-*tert*-butylhydroquinone (3.5 mg, 0.015 mmol, 10 mol%) and $NaHCO_3$ (25.0 mg, 0.30 mmol, 2 equiv) in 1 mL of dry DMSO. The reaction was irradiated with two Kessil 427 nm LEDs for 24 h at 55 °C to give the desired product **8** in 8% by ^{19}F NMR using C_6F_6 as an external standard

Ethyl 2,2-difluoro-2-(3-methylbenzo[*b*]thiophen-2-yl)acetate (9): Control reaction was carried out according to the general procedure with 3-methylbenzothiophene (22 µL, 0.15 mmol, 1 equiv), ethyl bromodifluoroacetate (49 µL, 0.375 mmol, 2.5 equiv), 2,5-di-*tert*-butylhydroquinone (3.5 mg, 0.015 mmol, 10 mol%) and $NaHCO_3$ (25.0 mg, 0.30 mmol, 2 equiv) in 1 mL of dry DMSO. The reaction was irradiated with two Kessil 427 nm LEDs for 24 h at 55 °C to give the desired product **9** in 8% by ^{19}F NMR using C_6F_6 as an external standard.

Ethyl 2,2-difluoro-2-(1-phenyl-1*H*-pyrrol-2-yl)acetate (10): Control reaction was carried out according to the general procedure with 1-phenylpyrrole (22 mg, 0.15 mmol, 1 equiv), ethyl bromodifluoroacetate (49 µL, 0.375 mmol, 2.5 equiv), 2,5-di-*tert*-butylhydroquinone (3.5 mg, 0.015 mmol, 10 mol%) and $NaHCO_3$ (25.0 mg, 0.30 mmol, 2 equiv) in 1 mL of dry DMSO. The reaction was irradiated with two Kessil 427 nm LEDs for 24 h at 55 °C to give the desired product **10** in 45% by ^{19}F NMR yield using C_6F_6 as an external standard.

Ethyl 2,2-difluoro-2-(1,3,7-trimethyl-2,6-dioxo-2,3,6,7-tetrahydro-1*H*-purin-8-yl)acetate (11): Control reaction was carried out according to the general procedure with caffeine (29 mg, 0.15 mmol, 1 equiv), ethyl bromodifluoroacetate (49 µL, 0.375 mmol, 2.5 equiv), 2,5-di-*tert*-butylhydroquinone (3.5 mg, 0.015 mmol, 10 mol%) and $NaHCO_3$ (25.0 mg, 0.30 mmol, 2 equiv) in 1 mL of dry DMSO. The reaction was irradiated with two Kessil 427 nm LEDs for 24 h at 55 °C to give the desired product **11** in 35% by ^{19}F NMR yield using C_6F_6 as an external standard.

(*R*)-2-((*tert*-butoxycarbonyl)amino)-3-(2-(2-ethoxy-1,1-difluoro-2-oxoethyl)-1*H*-indol-3-yl)propanoic acid (12): Control reaction was carried out according to the general procedure with Boc-Trp-OH (46 mg, 0.15 mmol, 1 equiv), ethyl bromodifluoroacetate (49 µL, 0.375 mmol, 2.5 equiv), 2,5-di-*tert*-butylhydroquinone (3.5 mg, 0.015 mmol, 10 mol%) and $NaHCO_3$ (25.0 mg, 0.30 mmol, 2 equiv) in 1 mL of dry DMSO. The reaction was irradiated with two Kessil 427 nm LEDs for 24 h at 55 °C to give the desired product **12** in trace amounts by ^{19}F NMR using C_6F_6 as an external standard.

Ethyl 2-(3-(2-acetamidoethyl)-5-methoxy-1*H*-indol-2-yl)-2,2-difluoroacetate (13): Control reaction was carried out according to the general procedure with melatonin (35 mg, 0.15 mmol, 1

equiv), ethyl bromodifluoroacetate (49 µL, 0.375 mmol, 2.5 equiv), 2,5-di-*tert*-butylhydroquinone (3.5 mg, 0.015 mmol, 10 mol%) and $NaHCO_3$ (25.0 mg, 0.30 mmol, 2 equiv) in 1 mL of dry DMSO. The reaction was irradiated with two Kessil 427 nm LEDs for 24 h at 55 °C to give the desired product **13** in 42% by ^{19}F NMR using C_6F_6 as an external standard.

Ethyl 2,2-difluoro-2-(2-oxo-2*H*-chromen-3-yl)acetate (14): Control reaction was carried out according to the general procedure with coumarin (22 mg, 0.15 mmol, 1 equiv), ethyl bromodifluoroacetate (49 µL, 0.375 mmol, 2.5 equiv), 2,5-di-*tert*-butylhydroquinone (3.5 mg, 0.015 mmol, 10 mol%) and $NaHCO_3$ (25.0 mg, 0.30 mmol, 2 equiv) in 1 mL of dry DMSO. The reaction was irradiated with two Kessil 427 nm LEDs for 24 h at 55 °C to give the desired product **14** in trace amounts by ^{19}F NMR using C_6F_6 as an external standard.

Ethyl 2-(1,3-dimethyl-2,4-dioxo-1,2,3,4-tetrahydropyrimidin-5-yl)-2,2-difluoroacetate (15): Control reaction was carried out according to the general procedure with 1,3-dimethyluracil (20 mg, 0.15 mmol, 1 equiv), ethyl bromodifluoroacetate (49 µL, 0.375 mmol, 2.5 equiv), 2,5-di-*tert*-butylhydroquinone (3.5 mg, 0.015 mmol, 10 mol%) and $NaHCO_3$ (25.0 mg, 0.30 mmol, 2 equiv) in 1 mL of dry DMSO. The reaction was irradiated with two Kessil 427 nm LEDs for 24 h at 55 °C to give the desired product **15** in trace amounts ^{19}F NMR yield using C_6F_6 as an external standard.

Ethyl 2-(2,4-dioxo-1,2,3,4-tetrahydropyrimidin-5-yl)-2,2-difluoroacetate (16): Control reaction was carried out according to the general procedure with uracil (17 mg, 0.15 mmol, 1 equiv), ethyl bromodifluoroacetate (49 µL, 0.375 mmol, 2.5 equiv), 2,5-di-*tert*-butylhydroquinone (3.5 mg, 0.015 mmol, 10 mol%) and $NaHCO_3$ (25.0 mg, 0.30 mmol, 2 equiv) in 1 mL of dry DMSO. The reaction was irradiated with two Kessil 427 nm LEDs for 24 h at 55 °C to give the desired product **16** in 21% by ^{19}F NMR using C_6F_6 as an external standard.

Adamantan-1-yl 2,2-difluoro-2-(1-phenyl-1H-pyrrol-2-yl)acetate (17): Control reaction was carried out according to the general procedure with 1-phenylpyrrole (22 mg, 0.15 mmol, 1 equiv), adamantan-1-yl 2-bromo-2,2-difluoroacetate (116 mg, 0.375 mmol, 2.5 equiv), 2,5-di-*tert*-butylhydroquinone (3.5 mg, 0.015 mmol, 10 mol%) and $NaHCO_3$ (25.0 mg, 0.30 mmol, 2 equiv)

in 1 mL of dry DMSO. The reaction was irradiated with two Kessil 427 nm LEDs for 24 h at 55 °C to give the desired product **17** in 42% by ^{19}F NMR using C_6F_6 as an external standard.

(1*S*,2*R*,5*S*)-2-isopropyl-5-methylcyclohexyl 2,2-difluoro-2-(1-phenyl-1*H*-pyrrol-2-yl)acetate (18): Control reaction was carried out according to the general procedure with 1-phenylpyrrole (63 mg, 0.375 mmol, 2.5 equiv), (1*S*,2*R*,5*S*)-2-isopropyl-5-methylcyclohexyl 2-bromo-2,2-difluoroacetate (33 µL, 0.15 mmol, 1 equiv), 2,5-di-*tert*-butylhydroquinone (3.5 mg, 0.015 mmol, 10 mol%) and $NaHCO_3$ (25.0 mg, 0.30 mmol, 2 equiv) in 1 mL of dry DMSO. The reaction was irradiated with two Kessil 427 nm LEDs for 24 h at 55 °C to give the desired product **18** in 27% by ^{19}F NMR using C_6F_6 as an external standard.

2,2-Difluoro-2-(1-phenyl-1*H*-pyrrol-2-yl)-1-(piperidin-1-yl)ethan-1-one (19): Control reaction was carried out according to the general procedure with 1-phenylpyrrole (22 mg, 0.15 mmol, 1 equiv), 2-bromo-2,2-difluoro-1-(piperidin-1-yl)ethan-1-one (58 µL, 0.375 mmol, 2.5 equiv), 2,5-di-*tert*-butylhydroquinone (3.5 mg, 0.015 mmol, 10 mol%) and $NaHCO_3$ (25.0 mg, 0.30 mmol, 2 equiv) in 1 mL of dry DMSO. The reaction was irradiated with two Kessil 427 nm LEDs for 24 h at 55 °C to give the desired product **19** in 57% by ^{19}F NMR using C_6F_6 as an external standard.

***N*,*N*-diethyl-2,2-difluoro-2-(1-phenyl-1*H*-pyrrol-2-yl)acetamide (20)**: Control reaction was carried out according to the general procedure with 1-phenylpyrrole (22 mg, 0.15 mmol, 1 equiv), 2-bromo-*N*,*N*-diethyl-2,2-acetamide (184 µL, 0.375 mmol, 2.5 equiv), 2,5-di-*tert*-butylhydroquinone (3.5 mg, 0.015 mmol, 10 mol%) and $NaHCO_3$ (25.0 mg, 0.30 mmol, 2 equiv) in 1 mL of dry DMSO. The reaction was irradiated with two Kessil 427 nm LEDs for 24 h at 55 °C to give the desired product **20** in 48% by ^{19}F NMR using C_6F_6 as an external standard.

***N*-benzyl-2,2-difluoro-2-(1-phenyl-1*H*-pyrrol-2-yl)acetamide (21)**: Control reaction was carried out according to the general procedure with 1-phenylpyrrole (22 mg, 0.15 mmol, 1 equiv), *N*-benzyl-2-bromo-2,2-difluoroacetamide (99 mg, 0.375 mmol, 2.5 equiv), 2,5-di-*tert*-butylhydroquinone (3.5 mg, 0.015 mmol, 10 mol%) and $NaHCO_3$ (25.0 mg, 0.30 mmol, 2 equiv) in 1 mL of dry DMSO. The reaction was irradiated with two Kessil 427 nm LEDs for 24 h at 55 °C to give the desired product **21** in 56% by ^{19}F NMR using C_6F_6 as an external standard.

2,2-Difluoro-2-(1-phenyl-1*H*-pyrrol-2-yl)acetamide (22): Control reaction was carried out according to the general procedure with 1-phenylpyrrole (22 mg, 0.15 mmol, 1 equiv), 2-bromo-2,2-difluoroacetamide (66 mg, 0.375 mmol, 2.5 equiv), 2,5-di-*tert*-butylhydroquinone (3.5 mg, 0.015 mmol, 10 mol%) and $NaHCO_3$ (25.0 mg, 0.30 mmol, 2 equiv) in 1 mL of dry DMSO. The reaction was irradiated with two Kessil 427 nm LEDs for 24 h at 55 °C to give the desired product **22** in 14% by ^{19}F NMR using C_6F_6 as an external standard.

2-(Perfluorobutyl)-1-phenyl-1*H*-pyrrole (23): Control reaction was carried out according to the general procedure with 1-phenylpyrrole (22 mg, 0.15 mmol, 1 equiv), perfluorobutyl bromide (58 µL, 0.375 mmol, 2.5 equiv), 2,5-di-*tert*-butylhydroquinone (3.5 mg, 0.015 mmol, 10 mol%) and $NaHCO_3$ (25.0 mg, 0.30 mmol, 2 equiv) in 1 mL of dry DMSO. The reaction was irradiated with two Kessil 427 nm LEDs for 24 h at 55 °C to give the desired product **23** in 56% by ^{19}F NMR using C_6F_6 as an external standard.

2-(Difluoro(1-phenyl-1*H*-pyrrol-2-yl)methyl)benzo[*d*]oxazole (24): Control reaction was carried out according to the general procedure with 1-phenylpyrrole (22 mg, 0.15 mmol, 1 equiv), 2-(bromodifluoromethyl)-1,3-benzoxazole (53 µL, 0.375 mmol, 2.5 equiv), 2,5-di-*tert*-butylhydroquinone (3.5 mg, 0.015 mmol, 10 mol%) and $NaHCO_3$ (25.0 mg, 0.30 mmol, 2 equiv) in 1 mL of dry DMSO. The reaction was irradiated with two Kessil 427 nm LEDs for 24 h at 55 °C to give the desired product **24** in 55% by ^{19}F NMR using C_6F_6 as an external standard.

F. Low Yielding Scope Examples.

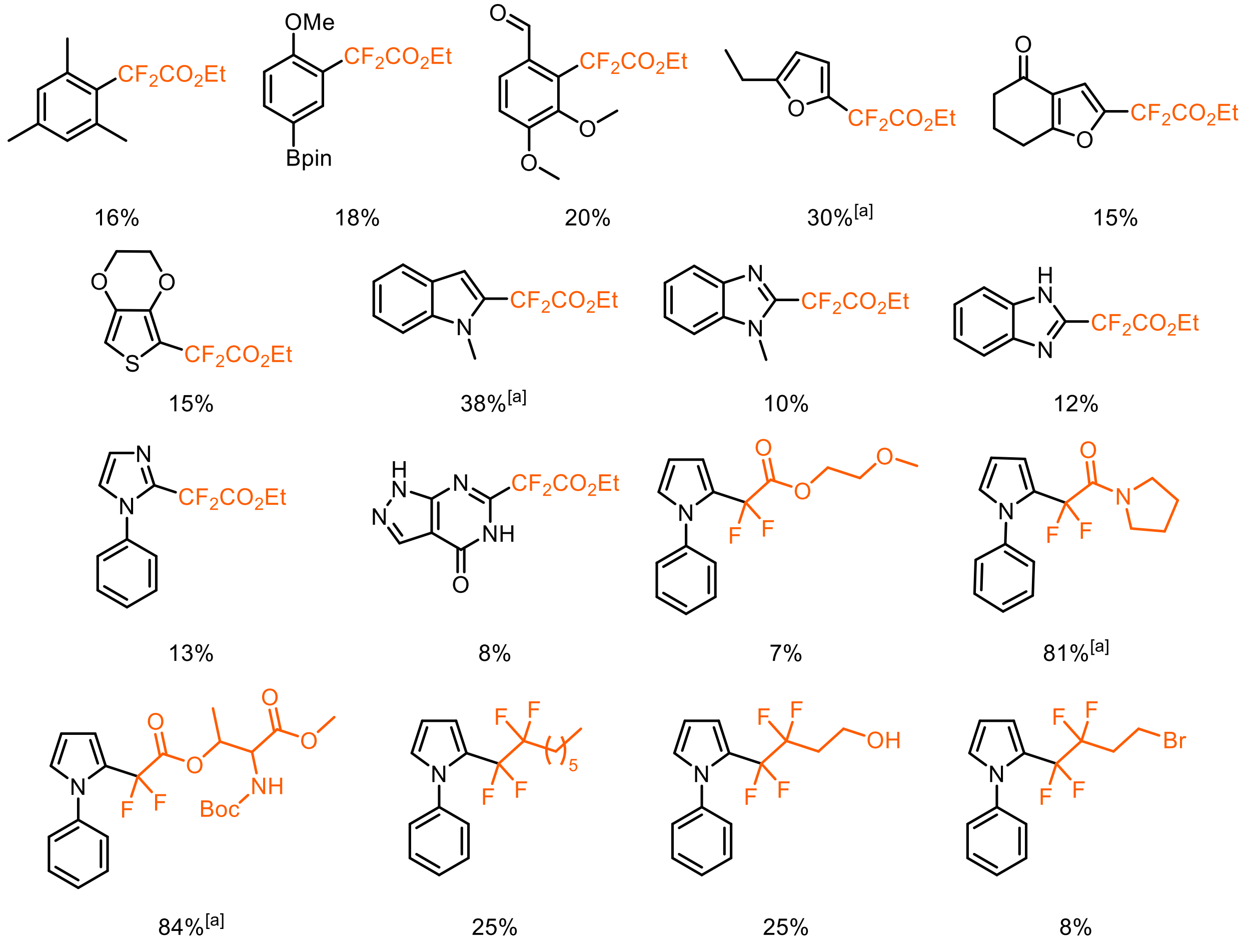

Figure S3. Low yielding scope examples for the *gem*-difluoroalkylation of electron-rich (hetero)arenes. Yields are reported as ^{19}F NMR yields using hexafluorobenzene as an external standard. [a] Could not be isolated in adequate purity for characterization.

G. UV-Vis Studies

Ethyl difluoroiodoacetate (ICF_2CO_2Et) was purchased as a dark purple liquid from Oakwood Chemical, washed with sat. aq. Na_2SO_4 (2 x 5mL), sat. aq. $NaHCO_3$ (5mL), sat. aq. NaCl (5 mL), dried over $MgSO_4$ and filtered through a short plug of neutral alumina to give a mostly decolorized (pale yellow) liquid which was used for the analysis.

To determine whether a CTC between the DTHQ catalyst and α-halodifluoroester (or amide) was responsible for the observed reactivity under visible light irradiation, we turned to UV-Vis studies (Figure S4). For these experiments, a series of freshly prepared solutions in DMSO were added to a quartz cuvette and the absorbance from 300-450 nm was measured. It was observed that DTHQ does not absorb in the visible region on its own, and while addition of $BrCF_2CO_2Et$ did result in a small redshift, the absorption of this CTC in the visible region was minimal. In contrast, the addition ICF_2CO_2Et to a solution of DTHQ does result in a redshift into the visible region. Therefore, this provides evidence that an *in-situ* bromide displacement by I^- must occur to generate a CTC that is capable of absorbing 427 nm irradiation.

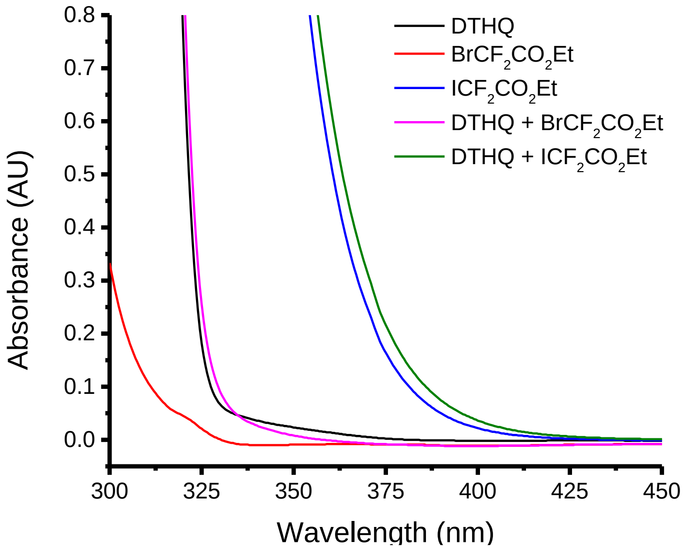

Figure S4. Absorption spectra of **(I)** DTHQ (0.015M), **(II)** $BrCF_2CO_2Et$ (0.15M), **(III)** ICF_2CO_2Et (0.15M), **(IV)** $BrCF_2CO_2Et$ + DTHQ, and **(V)** ICF_2CO_2Et + DTHQ.

To determine the association constants (K_a) for the DTHQ:ICF_2CO_2Et and DTHQ:$BrCF_2CO_2Et$ halogen-bonding complexes, we performed a Benesi–Hildebrand analysis.[1] In a quartz cuvette, the absorbance from 300-600 nm of a freshly prepared solution of DTHQ (15

mM) in DMSO was measured. To the solution of DTHQ was then added 10 equiv of XCF_2CO_2Et. The absorbance of this solution was then measured, and the procedure was repeated to give a range of 10 to 60 equivalents of XCF_2CO_2Et compared to the concentration of DTHQ (see Figures S5 and S6).

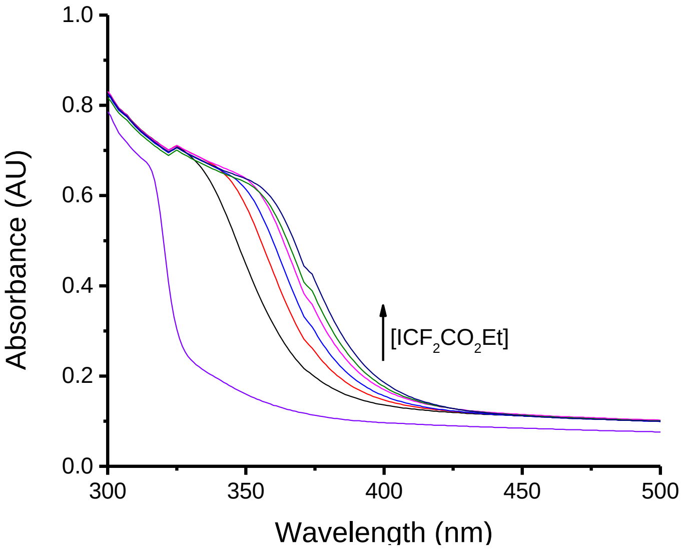

Figure S5. Absorption spectra of DTHQ (15 mM) with increasing concentrations of ICF_2CO_2Et (10-60 equiv) in DMSO.

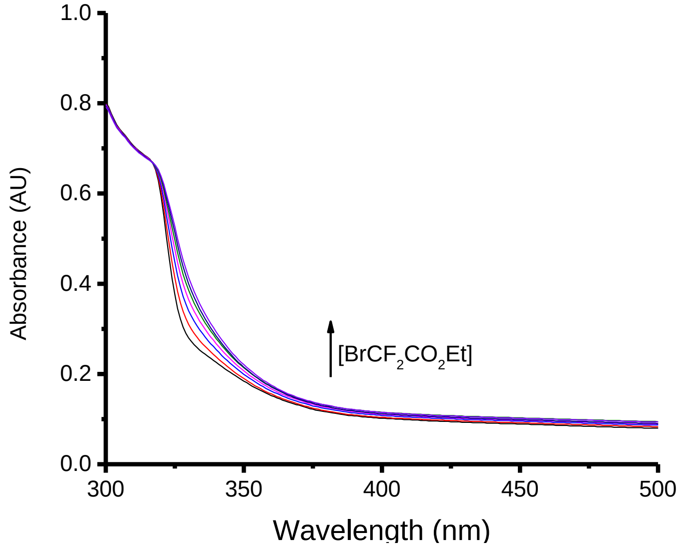

Figure S6. Absorption spectra of DTHQ (15 mM) with increasing concentrations of $BrCF_2CO_2Et$ (10-60 equiv) in DMSO.

To perform the Benesi–Hildebrand analysis, we then plotted the reciprocal of the change of absorbance of the DTHQ:ICF_2CO_2Et complex at 410 nm versus the reciprocal of the concentration

of ICF_2CO_2Et. For the DTHQ:$BrCF_2CO_2Et$ complex, we plotted the reciprocal of the change in absorbance of the complex at 390 nm versus the reciprocal of the concentration of $BrCF_2CO_2Et$. The corresponding plots can be found below in Figures S7 and S8. The linear relationship observed indicates that the stoichiometry of both halogen-bonding complexes is 1:1.

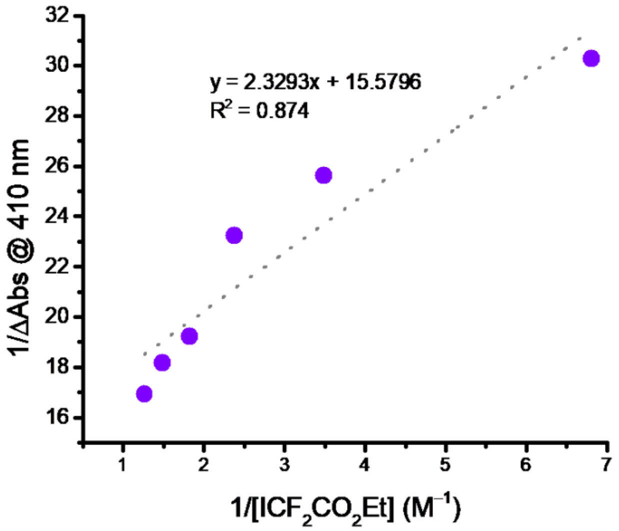

Figure S7. Benesi–Hildebrand plot for the determination of the association constant (K_a) between DTHQ and ICF_2CO_2Et. The change in absorbance at 410 nm was monitored as increasing concentrations of ICF_2CO_2Et (1-60 equiv) was added to a solution of DTHQ (15 mM) in DMSO.

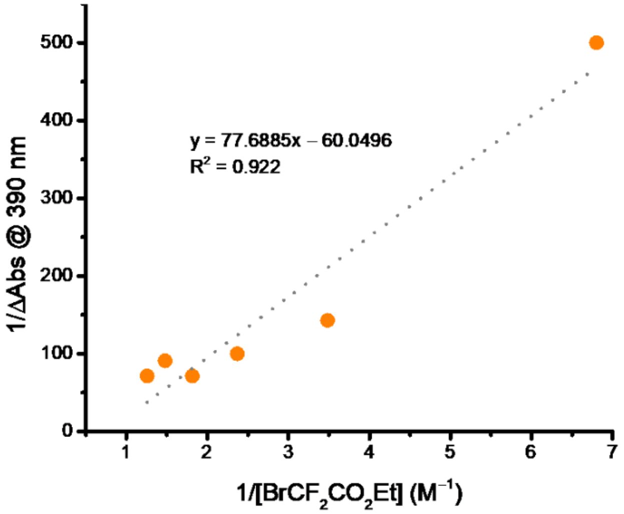

Figure S8. Benesi–Hildebrand plot for the determination of the association constant (K_a) between DTHQ and $BrCF_2CO_2Et$. The change in absorbance at 410 nm was monitored as increasing concentrations of $BrCF_2CO_2Et$ (1-60 equiv) was added to a solution of DTHQ (15 mM) in DMSO.

The linear relationship was then used to solve the Benesi–Hildebrand equation (eq S1), giving a K_a of 0.43 M^{-1} and 0.013 M^{-1} for the DTHQ:ICF_2CO_2Et and DTHQ:$BrCF_2CO_2Et$ halogen-bonding complexes in DMSO, respectively. As expected, ICF_2CO_2Et is a significantly stronger halogen-bond donor compared to $BrCF_2CO_2Et$, resulting in a larger observed K_a value.

$$\frac{1}{\Delta A_{440\,nm}} = \frac{1}{A_{440\,nm}} \times \frac{1}{K_a}\frac{1}{[C_4F_9I]} + \frac{1}{A_{440\,nm}} \quad (S1)$$

To help elucidate the role of Bu_4NI in our reaction, we performed additional UV-vis studies (Figure S9). Upon addition of Bu_4NI to a solution of $BrCF_2CO_2Et$ in DMSO, a significant redshift of the absorption band was observed, indicating the formation of a charge-transfer complex. This charge-transfer complex provides an explanation for the inefficient product formation observed in the absence of DTHQ in our reactions. Addition of DTHQ to this solution did not result in a change to the absorption spectrum, indicating the formation of a ternary charge-transfer complex is unlikely.

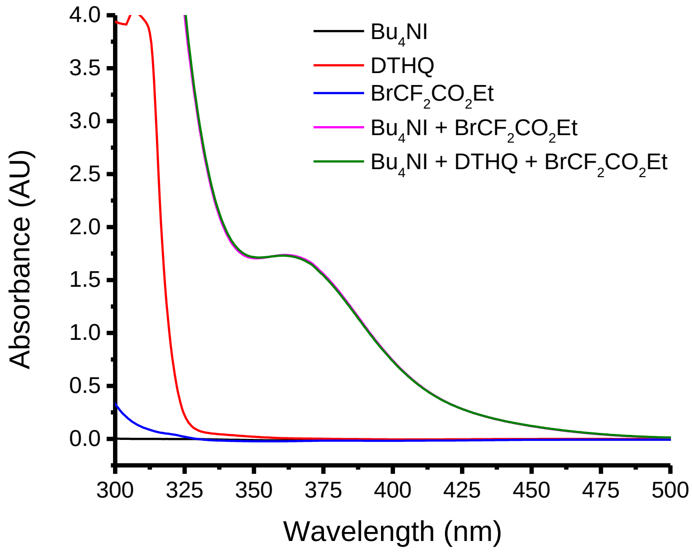

Figure S9. Absorption spectra of **(I)** Bu_4NI (0.030M), **(II)** DTHQ (0.015M), **(III)** $BrCF_2CO_2Et$ (0.375M), **(IV)** Bu_4NI + $BrCF_2CO_2Et$, and **(V)** $BrCF_2CO_2Et$ + DTHQ.

H. Initial Rate Experiments

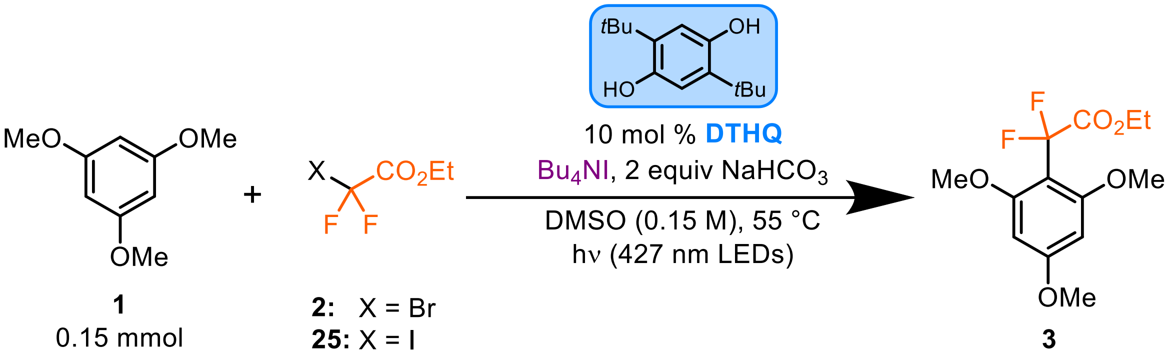

Following the general procedure, a series of initial rate experiment were carried out with 1,3,5-trimethoxybenzene (25 mg, 0.15 mmol, 1 equiv), XCF_2CO_2Et (X= Br; 49 μL, X= I; 55 μL, 0.375 mmol, 2.5 equiv), 2,5-di-*tert*-butylhydroquinone (7.0 mg, 0.03 mmol, 10 mol%), tetra-*N*-butyl ammonium iodide (0 equiv, 20 mol% or 1 equiv), and $NaHCO_3$ (168 mg, 2.0 mmol, 2 equiv) in 1 mL DMSO under Ar for 5 minutes, 10 minutes, 30 minutes, 1 hour, 2 h and 4 h at approximately 55 °C. Yields were determined by ^{19}F NMR using hexafluorobenzene as an external standard. The results are summarized in Figure S10.

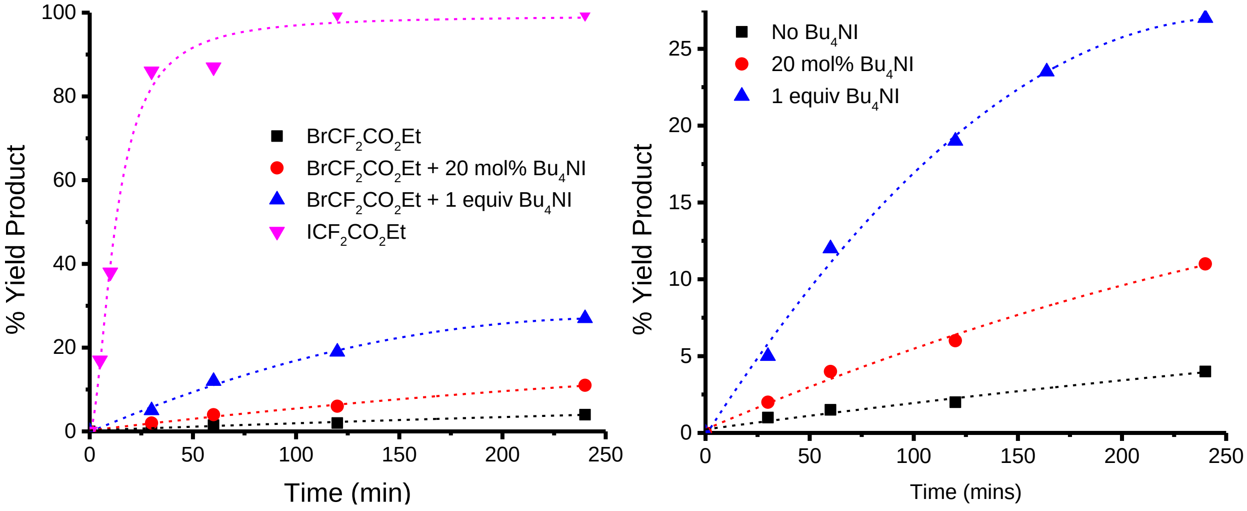

Figure S10. Effect of Bu_4NI on the initial reaction rate. The plot on the right contains experiments using $BrCF_2CO_2Et$ only.

I. Sensitive Functional Group Screen

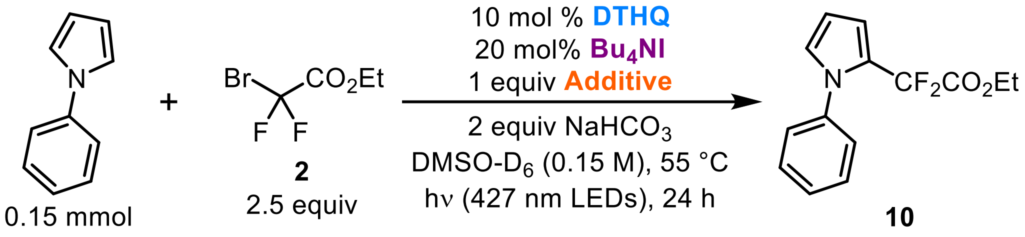

General Procedure: An oven-dried one-dram borosilicate glass vial equipped with a magnetic stir bar was charged with 1-phenylpyrrole (25 mg, 0.15 mmol, 1 equiv), 2,5-di-*tert*-butylhydroquinone (7.0 mg, 0.03 mmol, 10 mol%) tetra-*N*-butyl ammonium iodide (13 mg, 0.030 mmol, 20 mol%), $NaHCO_3$ (25.0 mg, 0.30 mmol, 2 equiv), additive (0.15 mmol, 1 equiv) in DMSO-D_6 (1 mL, 0.15 M), and degassed by sparging with argon for 5-6 minutes. To this, ethyl bromodifluoroacetate (49 µL, 0.375 mmol, 2.5 equiv) was added under Argon. The reaction mixture was then sonicated and irradiated with two Kessil 427nm LED lamps for 24 h at approximately 55 °C. Hexafluorobenzene was then added as an internal standard, and the mixture was then transferred to an NMR tube. Yields were determined by ^{19}F NMR.

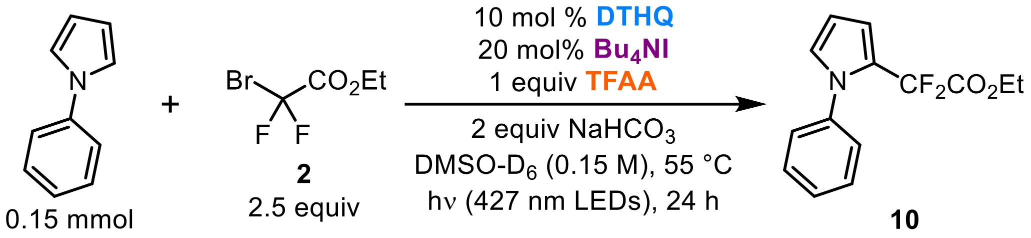

An oven-dried one-dram borosilicate glass vial equipped with a magnetic stir bar was charged with 1-phenylpyrrole (25 mg, 0.15 mmol, 1 equiv), 2,5-di-*tert*-butylhydroquinone (7.0 mg, 0.03 mmol, 10 mol%) tetra-*N*-butyl ammonium iodide (13 mg, 0.030 mmol, 20 mol%), $NaHCO_3$ (25.0 mg, 0.30 mmol, 2 equiv), trifluoroacetic anhydride (21 µL, 0.15 mmol, 1 equiv) in DMSO-D_6 (1 mL, 0.15 M), and degassed by sparging with argon for 5-6 minutes. To this, ethyl bromodifluoroacetate (49 µL, 0.375 mmol, 2.5 equiv) was added under Argon. The reaction mixture was then sonicated and irradiated with two Kessil 427nm LED lamps for 24 h at approximately 55 °C. Hexafluorobenzene was then added as an internal standard, and the mixture was then transferred

to an NMR tube. The yield of **10** was determined by ^{19}F NMR to be 32%, and the amount of trifluoroacetic anhydride recovered was found to be 99%.

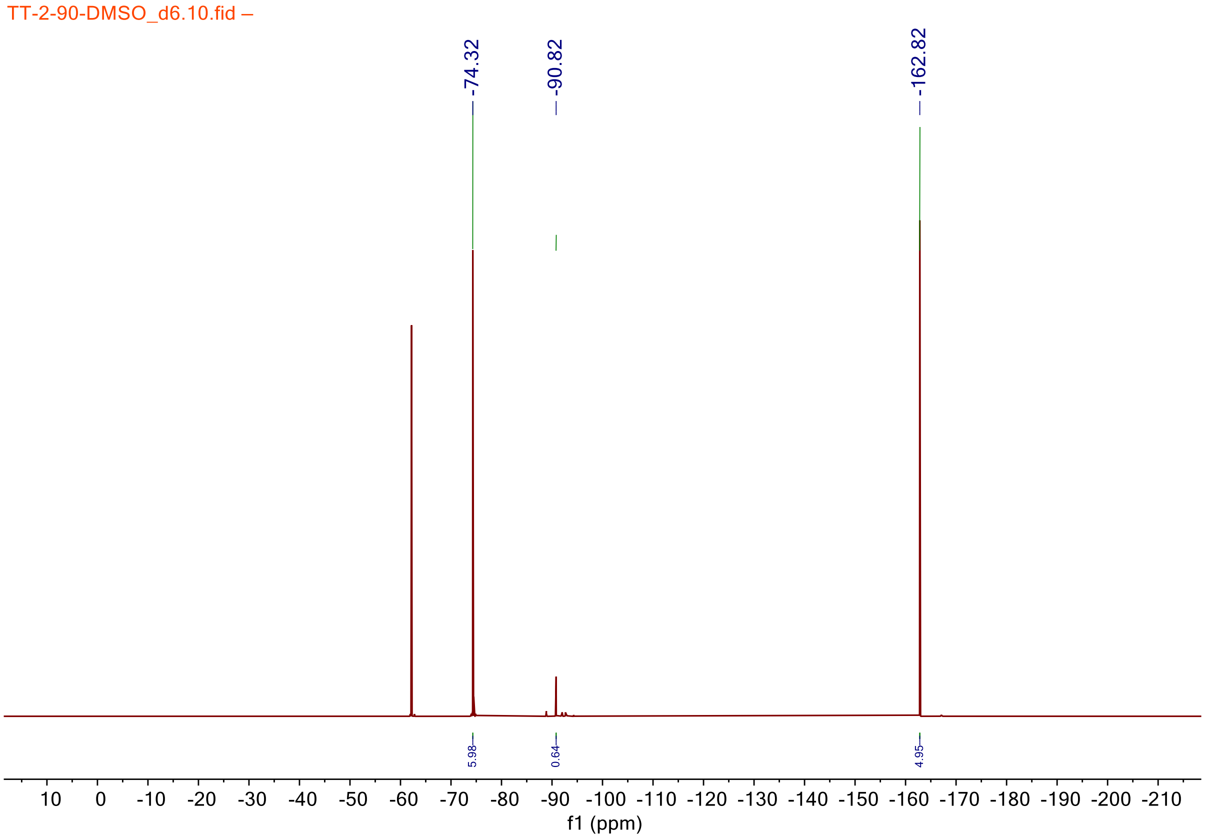

Figure S11. ^{19}F NMR (376 MHz, DMSO-D$_6$) for trifluoroacetic anhydride as an additive.

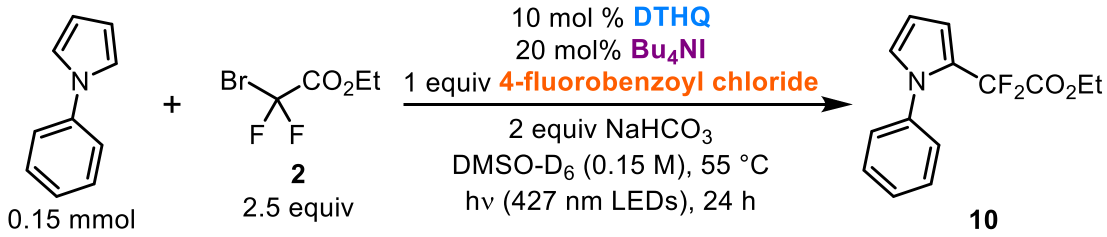

An oven-dried one-dram borosilicate glass vial equipped with a magnetic stir bar was charged with 1-phenylpyrrole (25 mg, 0.15 mmol, 1 equiv), 2,5-di-*tert*-butylhydroquinone (7.0 mg, 0.03 mmol, 10 mol%) tetra-*N*-butyl ammonium iodide (13 mg, 0.030 mmol, 20 mol%), $NaHCO_3$ (25.0 mg, 0.30 mmol, 2 equiv), 4-fluorobenzoyl chloride (18 μL, 0.15 mmol, 1 equiv) in DMSO-D$_6$ (1 mL, 0.15 M), and degassed by sparging with argon for 5-6 minutes. To this, ethyl bromodifluoroacetate (49 μL, 0.375 mmol, 2.5 equiv) was added under Argon. The reaction mixture was then sonicated and irradiated with two Kessil 427nm LED lamps for 24 h at approximately 55 °C.

Hexafluorobenzene was then added as an internal standard, and the mixture was then transferred to an NMR tube. The yield of **10** was determined by ^{19}F NMR to be 31%, and the amount of 4-fluorobenzoyl chloride recovered was found to be 73%.

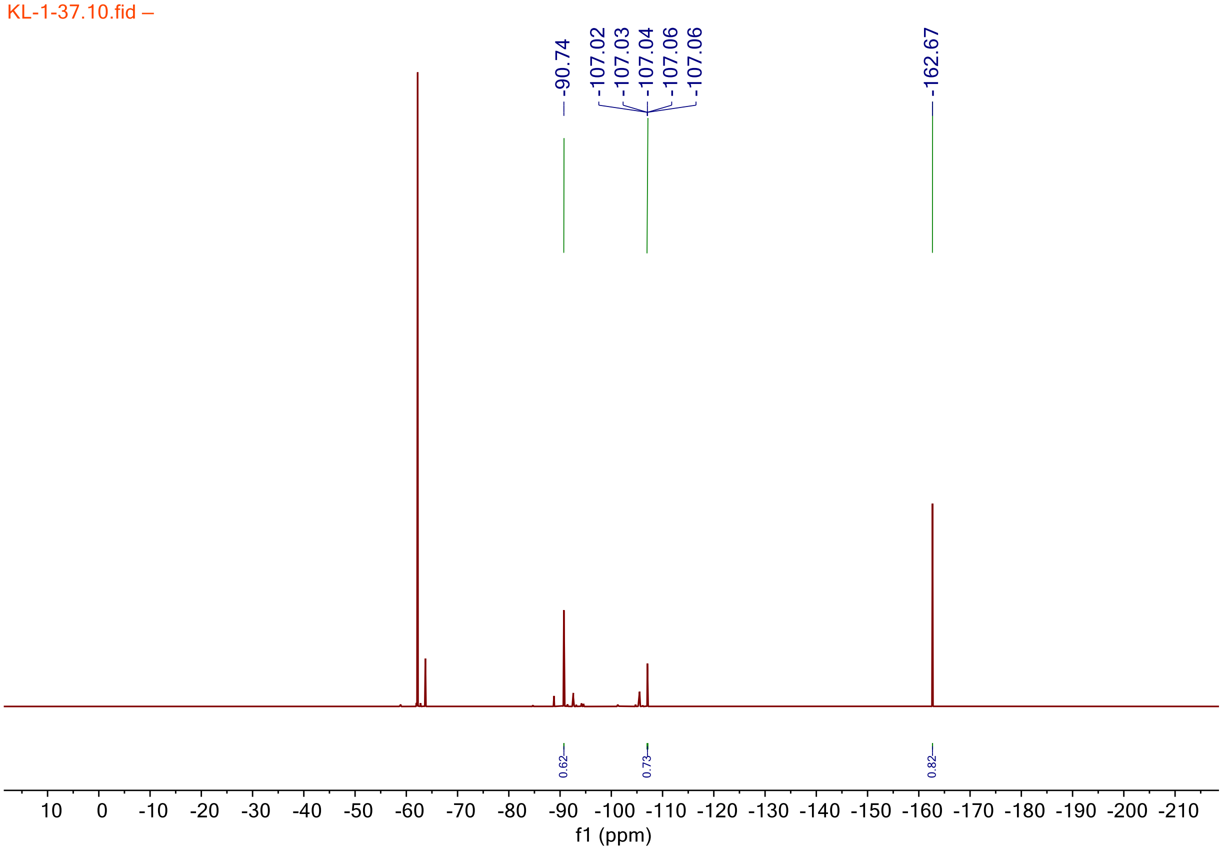

Figure S12. ^{19}F NMR (376 MHz, DMSO-D$_6$) for 4-fluorobenzoyl chloride as an additive.

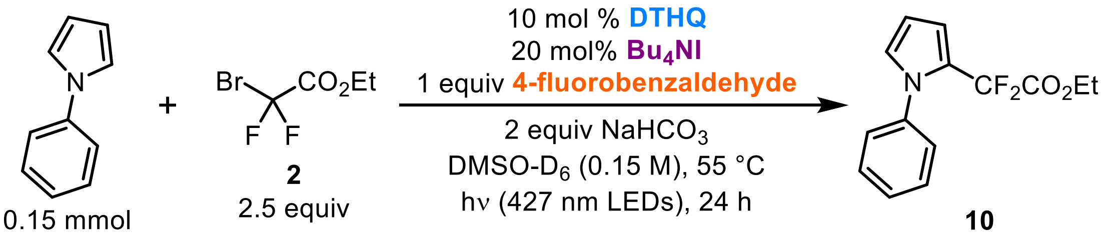

An oven-dried one-dram borosilicate glass vial equipped with a magnetic stir bar was charged with 1-phenylpyrrole (25 mg, 0.15 mmol, 1 equiv), 2,5-di-*tert*-butylhydroquinone (7.0 mg, 0.03 mmol, 10 mol%) tetra-*N*-butyl ammonium iodide (13 mg, 0.030 mmol, 20 mol%), $NaHCO_3$ (25.0 mg, 0.30 mmol, 2 equiv), 4-fluorobenzaldehyde (16 µL, 0.15 mmol, 1 equiv) in DMSO-D$_6$ (1 mL, 0.15 M), and degassed by sparging with argon for 5-6 minutes. To this, ethyl bromodifluoroacetate (49 µL, 0.375 mmol, 2.5 equiv) was added under Argon. The reaction mixture was then sonicated and

irradiated with two Kessil 427nm LED lamps for 24 h at approximately 55 °C. Hexafluorobenzene was then added as an internal standard, and the mixture was then transferred to an NMR tube. The yield of **10** was determined by ^{19}F NMR to be 70%, and the amount of 4-fluorobenzaldehyde recovered was found to be 80%.

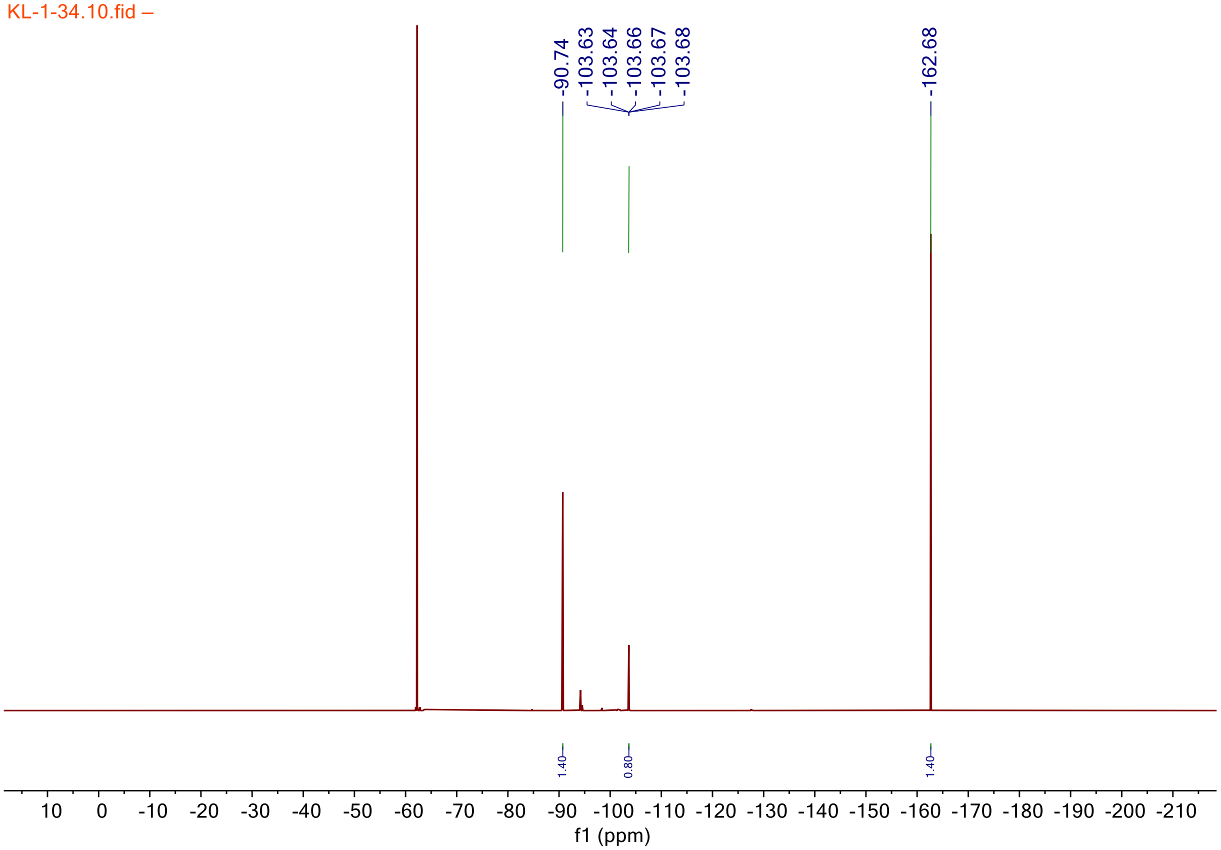

Figure S13. ^{19}F NMR (376 MHz, DMSO-D$_6$) for 4-fluorobenzaldehyde as an additive.

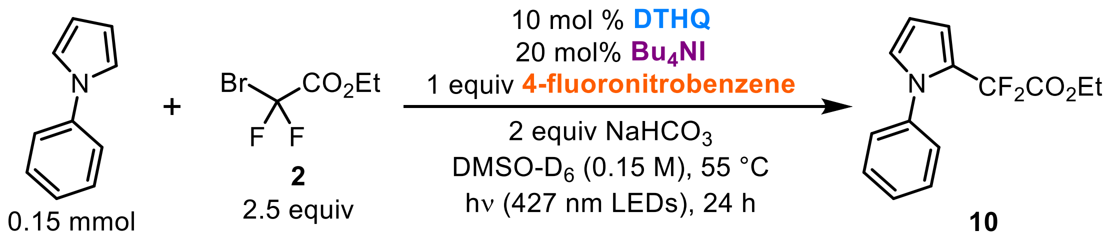

An oven-dried one-dram borosilicate glass vial equipped with a magnetic stir bar was charged with 1-phenylpyrrole (25 mg, 0.15 mmol, 1 equiv), 2,5-di-*tert*-butylhydroquinone (7.0 mg, 0.03 mmol, 10 mol%) tetra-*N*-butyl ammonium iodide (13 mg, 0.030 mmol, 20 mol%), $NaHCO_3$ (25.0 mg,

0.30 mmol, 2 equiv), 4-fluoronitrobenzene (16 µL, 0.15 mmol, 1 equiv) in DMSO-D$_6$ (1 mL, 0.15 M), and degassed by sparging with argon for 5-6 minutes. To this, ethyl bromodifluoroacetate (49 µL, 0.375 mmol, 2.5 equiv) was added under Argon. The reaction mixture was then sonicated and irradiated with two Kessil 427nm LED lamps for 24 h at approximately 55 °C. Hexafluorobenzene was then added as an internal standard, and the mixture was then transferred to an NMR tube. The yield of **10** was determined by ^{19}F NMR to be 23%, and the amount of 4-fluoronitrobenzene recovered was found to be 93%.

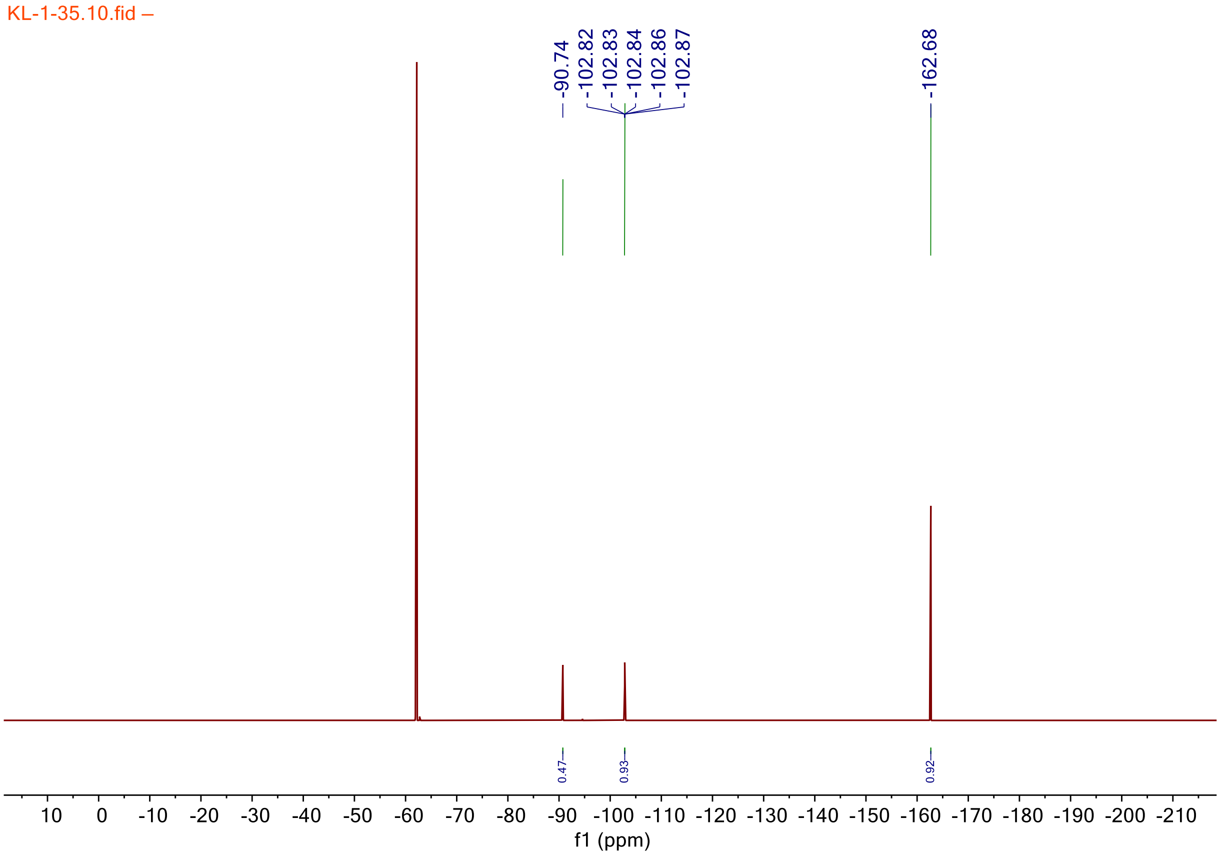

Figure S14. ^{19}F NMR (376 MHz, DMSO-D$_6$) for 4-fluoronitrobenzene as an additive.

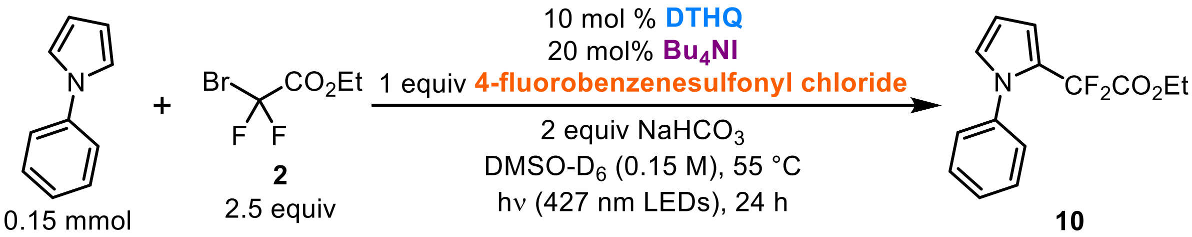

An oven-dried one-dram borosilicate glass vial equipped with a magnetic stir bar was charged with 1-phenylpyrrole (25 mg, 0.15 mmol, 1 equiv), 2,5-di-*tert*-butylhydroquinone (7.0 mg, 0.03 mmol,

10 mol%) tetra-*N*-butyl ammonium iodide (13 mg, 0.030 mmol, 20 mol%), $NaHCO_3$ (25.0 mg, 0.30 mmol, 2 equiv), 4-fluorobenzenesulfonyl chloride (21 mg, 0.15 mmol, 1 equiv) in DMSO-D_6 (1 mL, 0.15 M), and degassed by sparging with argon for 5-6 minutes. To this, ethyl bromodifluoroacetate (49 µL, 0.375 mmol, 2.5 equiv) was added under Argon. The reaction mixture was then sonicated and irradiated with two Kessil 427nm LED lamps for 24 h at approximately 55 °C. Hexafluorobenzene was then added as an internal standard, and the mixture was then transferred to an NMR tube. The yield of **10** was determined by ^{19}F NMR to be 11%, and the amount of 4-fluorobenzenesulfonyl chloride recovered was found to be 77%.

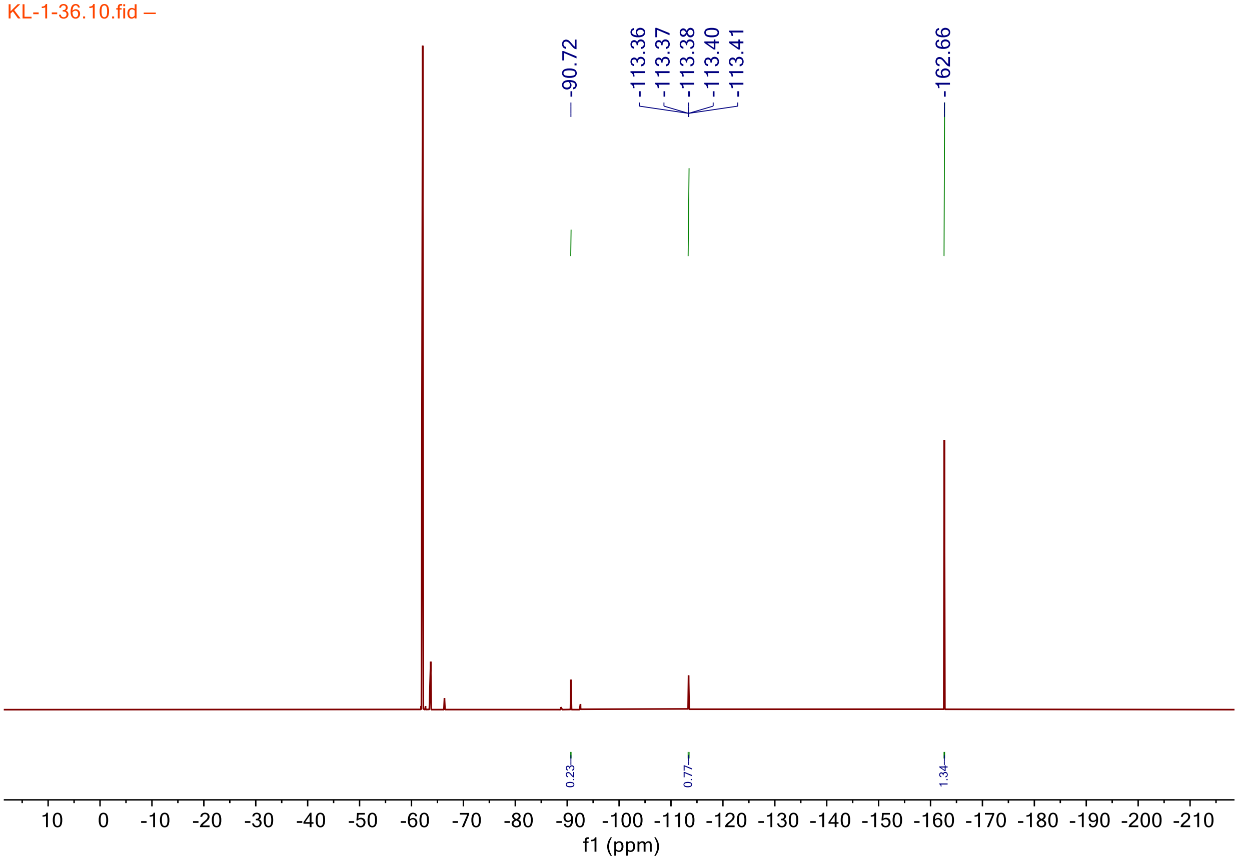

Figure S15. ^{19}F NMR (376 MHz, DMSO-D_6) for 4-fluorobenzenesulfonyl chloride as an additive.

Table S2. Summary of the sensitive functional group screen.

Additive	% Yield Desired Product	% Additive Recovered
None	78%	-
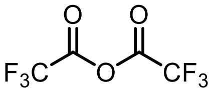	32%	99%
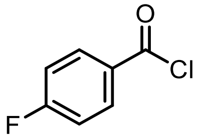	31%	73%
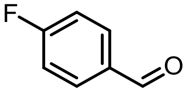	70%	80%
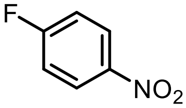	23%	93%
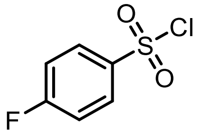	11%	77%

J. Finkelstein Displacement Studies

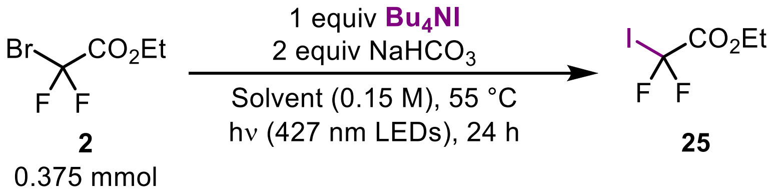

An oven-dried one-dram borosilicate glass vial equipped with a magnetic stir bar was charged with tetra-*N*-butyl ammonium iodide (139 mg, 0.375 mmol, 1 equiv) and $NaHCO_3$ (63 mg, 0.75 mmol, 2 equiv). Solvent (DMSO-D_6/Acetone-D_6) was added (1 mL, 0.15 M), and the reaction mixture was degassed by sparging with argon for 5-6 minutes. To this, ethyl bromodifluoroacetate (**2**) (49 μL, 0.375 mmol, 1 equiv) was added under argon. The reaction mixture was then sonicated and irradiated with two Kessil 427nm LED lamps for 24 h at approximately 55 °C. Following the reaction, hexafluorobenzene was added as an external ^{19}F NMR standard (δ −164.9 ppm).

To compare with the spectra of the reaction mixture, two control samples were prepared in both DMSO-D_6 and Acetone-D_6: one containing ethyl bromodifluoroacetate (**2**) (49 μL, 0.375 mmol, 1 equiv), tetra-*N*-butyl ammonium iodide (139 mg, 0.375 mmol, 1 equiv) and hexafluorobenzene, and the other containing ethyl difluoroiodoacetate (**25**) (55 μL, 0.375 mmol, 1 equiv), tetra-*N*-butyl ammonium iodide (139 mg, 0.375 mmol, 1 equiv) and hexafluorobenzene.

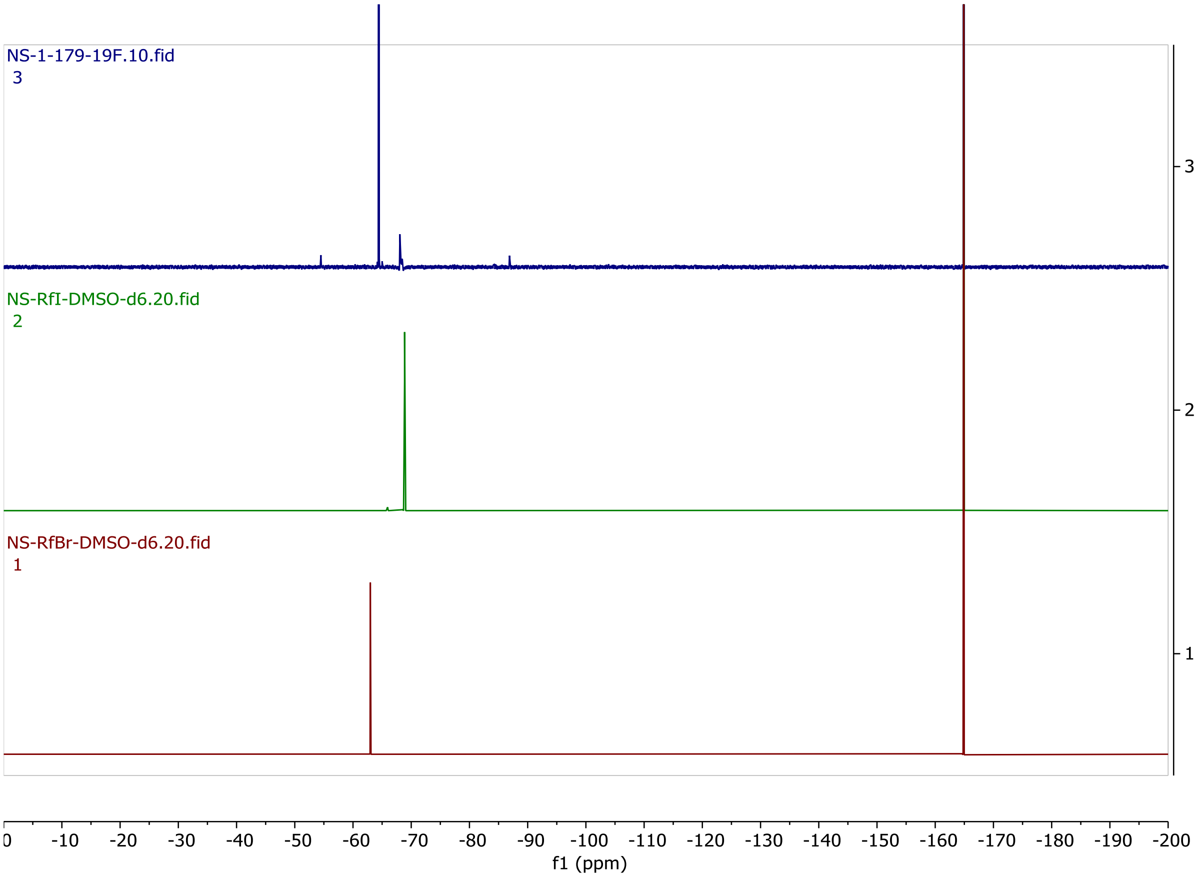

Figure S16. ^{19}F NMR (376 MHz) spectra for the Finkelstein displacement control reaction in DMSO-D$_6$. Top (navy) trace: crude reaction mixture; middle trace (green): **25** + 1 equiv Bu_4NI; bottom (red) trace: **2** + 1 equiv Bu_4NI. All spectra were referenced using hexafluorobenzene (δ −164.9 ppm).

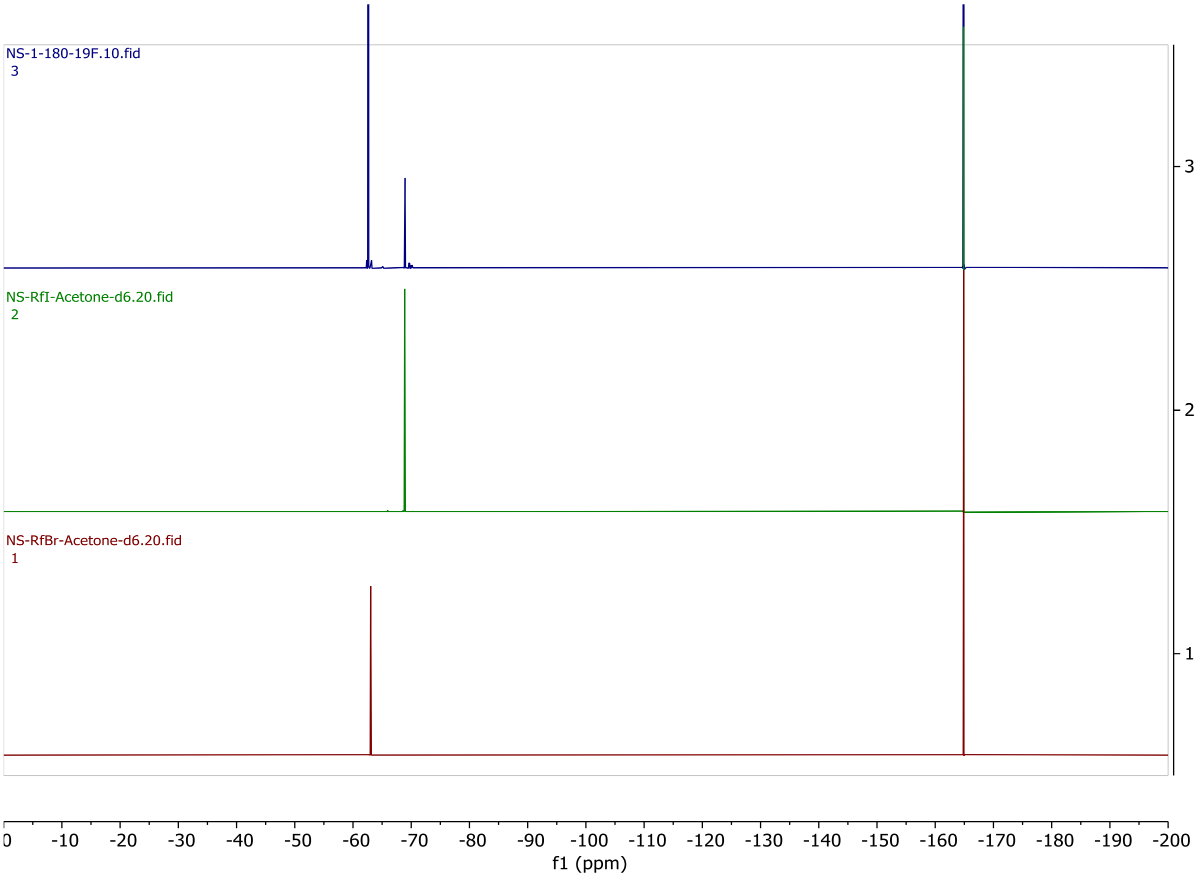

Figure S17. ^{19}F NMR (376 MHz) spectra for the Finkelstein displacement control reaction in Acetone-D_6. Top (navy) trace: crude reaction mixture; middle trace (green): **25** + 1 equiv Bu_4NI; bottom (red) trace: **2** + 1 equiv Bu_4NI. All spectra were referenced using hexafluorobenzene (δ −164.9 ppm).

K. Computational Studies

K.1. *Thermodynamic Stability of Difluoroalkyl Complexes*

To assess the favorability of reactive reagent complex formation, density functional theory calculations were performed on all possible combinations of *gem*-difluoroalkyl halides with both interacting and non-interacting iodide and bromide ions present. Calculations used the Gaussian16 program, initially in a vacuum state using the B3LYP and M06 functionals with the Grimme *et al*. D3BJ correction applied to the B3LYP calculations.[2–5] All optimizations and frequency calculations were performed using the def2-SVPP, def2-TZVPP and def2-QZVPP basis sets, this to project out to the continuous basis set limit.[6] Trend and calculation consistency in the initial tests led to a focus on the B3LYP with dispersion correction calculations both in vacuum and in DMSO polarized continuum method solvation.

Table S3. Summary of relative free energy calculation values of difluoroalkyl complexes.

**Complex**	**PDB Index**	**vacuum rel. free energy** (kcal/mol)	**DMSO rel. free energy** (kcal/mol)
gem-difluoroalkyl bromide ••• Br^-	SS1	1.44	1.92
gem-difluoroalkyl bromide ••• I^-	SS2	3.26	1.51
gem-difluoroalkyl iodide ••• Br^-	SS3	**0.00**	0.31
gem-difluoroalkyl iodide ••• I^-	SS4	2.57	**0.00**
T.S.	SS5	18.06	23.44

To make comparisons between states, free ions need to be included with the systems. These ions are either separate or halogen-bonded to the *gem*-difluoroalkyl halide reagent. After a single halogen-bonding interaction, the negatively charged complex repels direct interaction with additional ions. This makes each system calculation a summation of the specific halogen-bonded complex and two free ions, either bromide or iodide, to maintain a constant system composition. In the case of the transition state, the formal negative charge of the activated complex counters additional interactions with anions, so an active halogen bond will not impact the leaving group behavior aside from potentially competing with anion interaction with the S_N2 interaction site. These interaction site characteristics are explored later in section K.4 of this SI text.

In vacuum calculations shown in Table S3, the bromine tends to show stronger favorability for association in the molecular complex, with iodide ions more stable as free ions in the surrounding

solution. In a polar solvent, this behavior is understandably inverted. The smaller bromide ions have a stronger solvation free energy than do larger iodide ions. There is a greater overall thermodynamic favorability for *gem*-difluoroalkyl iodide over bromide, and this favorability is a combination of the expectedly stronger halogen bonds that can form to iodine and the solvation favorability of bromide over iodide.

Solvation plays a significant role in the transition state behavior. There is a desolvation penalty in forming the activated complex in DMSO that is not observed in the vacuum calculations, amounting to an approximately 5 kcal/mol increase in barrier height. This large activation energy barrier acts as a kinetic barrier for conversion of *gem*-difluoroalkyl bromide in the presence of iodide ions to the thermodynamically favored *gem*-difluoralkyl iodide form. While not shown, the diiodo- and dibromo- transition states also exist, though ion exchange in these cases does not impact the population of reagents in solution. In general, solvation plays a strong role in the exchange favorability, and this overall process is supported with increasing polarity of the polar aprotic solvent.

The absolute enthalpy and entropy terms for these systems were also computed from the quantum partition function from the frequency calculation. From these state functions, an estimation of the temperature dependence of the free energy can be evaluated. It turns out that the relative free energy is not particularly sensitive to temperature over a 100 K temperature window centered near the reaction temperature of 350 K. The main use of temperature would be to increase the conversion rate from the *gem*-difluoroalkyl bromide to *gem*-difluoroalkyl iodide, increasing overall efficiency of the reaction through formation of the more reactive iodide complex.

PDB files of the optimized complexes and the transition state complex from the QZVPP basis set calculations **in DMSO continuum solvent** are presented on the following pages. In each indexed PDB file the final B3LYP energy is included as a REMARK near the top of the file. In the case of transition states, the number of imaginary frequencies and the specific frequency value are also included on separate REMARK lines. This format is used for all optimized geometries of indexed fragments and structures listed in the below sections and tables.

K.2. *Exploration of alternative mechanisms to Finkelstein reaction*

A common alternative mechanism for formation of *gem*-difluoroalkyl iodide from the bromine containing precursor in the presence of an iodide salt could be by way of direct loss of the bromine atom in the form of iodine monobromide. This would leave a transient difluoroalkyl anion in solution, and the iodine monobromide could rotate and reattach via the iodine atom to form the thermodynamically preferred arrangement of species. To investigate this possibility, the absolute free energy of the system of difluoroalkyl anion, iodine monobromide, a free bromide ion, and a free iodide ion was computed and compared to the transition state system of SS5 with free bromide and iodide.

Table S4. Summary of absolute free energy estimation calculations for components of the alternative mechanism state involving loss of IBr.

**Complex**	**PDB Index**	**DMSO abs. free energy** (Hartree)
difluoroalkyl anion (Def2SVPP)	-	-505.209899
difluoroalkyl anion (Def2TZVPP)	-	-505.838875
difluoroalkyl anion (Def2QZVPP)	SS6	-505.870604
IBr (Def2SVPP)	-	-2871.697089
IBr (Def2TZVPP)	-	-2872.024703
IBr (Def2QZVPP)	SS7	-2872.080834
I^- (Def2SVPP)	-	-297.991656
I^- (Def2TZVPP)	-	-298.009491
I^- (Def2QZVPP)	-	-298.010962
Br^- (Def2SVPP)	-	-2574.053397
Br^- (Def2TZVPP)	-	-2574.386661
Br^- (Def2QZVPP)	-	-2574.442608

The CBS relative free energy estimation for this state can be projected from the provided series values provided, using the minimum absolute free energy state of the iodide halogen bonded *gem*-difluoroalkyl iodide to provide a CBS state energy estimate of 49.02 kcal/mol. This is over 25 kcal/mol higher in free energy than the Finkelstein transition state detailed in Table S3, indicating this to be a highly unlikely mechanistic route for the reaction progression.

K.3. *Absolute activation energies for Finkelstein reaction conversions as a function of substrate*

To further investigate the feasibility of conversion of the *gem*-difluoroalkyl bromide to *gem*-difluoroalkyl iodide in the presence of an iodide salt via an S_N2 mechanism, we performed analogous thermodynamic and transition state calculations for a series of alternate substrate chemistries. This series spans replacing the fluorine atoms with hydrogen, chlorine, and bromine in different arrangements. The expectation is that the iodide form of the substrates (halogen bonded to an iodide atom) will be thermodynamically favored just like difluoroalkyl substrate. The activation energy barrier for conversion should grow with the size of the halogen atoms because of increased steric strain in the resulting transition state. So, the activation energy barrier heights should generally run according to the Br > Cl > F > H series. Tables S5-S9 summarize the results of these calculations in a manner similar to Table S3.

Table S5. Summary of relative free energy calculation values of HH-alkyl complexes.

**Complex**	**PDB Index**	**vacuum rel. free energy** (kcal/mol)	**DMSO rel. free energy** (kcal/mol)
gem-HH-alkyl bromide ••• Br^-	SS8	3.65	3.35
gem-HH-alkyl bromide ••• I^-	SS9	4.91	2.97
gem-HH-alkyl iodide ••• Br^-	SS10	0.00	0.50
gem-HH-alkyl iodide ••• I^-	SS11	2.04	0.00
T.S.	SS12	5.94	14.99

Table S6. Summary of relative free energy calculation values of HF-alkyl complexes.

**Complex**	**PDB Index**	**vacuum rel. free energy** (kcal/mol)	**DMSO rel. free energy** (kcal/mol)
gem-HF-alkyl bromide ••• Br^-	SS13	2.41	2.33
gem-HF-alkyl bromide ••• I^-	SS14	4.04	1.85
gem-HF-alkyl iodide ••• Br^-	SS15	0.00	0.51
gem-HF-alkyl iodide ••• I^-	SS16	2.41	0.00
T.S.	SS17	10.31	17.24

Table S7. Summary of relative free energy calculation values of FCl-alkyl complexes.

Complex	PDB Index	vacuum rel. free energy (kcal/mol)	DMSO rel. free energy (kcal/mol)
gem-FCl-alkyl bromide ••• Br^-	SS18	2.85	3.40
gem-FCl-alkyl bromide ••• I^-	SS19	4.86	3.06
gem-FCl-alkyl iodide ••• Br^-	SS20	0.00	0.54
gem-FCl-alkyl iodide ••• I^-	SS21	2.53	0.00
T.S.	SS22	17.80	23.96

Table S8. Summary of relative free energy calculation values of FBr-alkyl complexes.

Complex	PDB Index	vacuum rel. free energy (kcal/mol)	DMSO rel. free energy (kcal/mol)
gem-FBr-alkyl bromide ••• Br^-	SS23	3.15	3.75
gem-FBr-alkyl bromide ••• I^-	SS24	5.17	3.08
gem-FBr-alkyl iodide ••• Br^-	SS25	0.00	0.45
gem-FBr-alkyl iodide ••• I^-	SS26	2.54	0.00
T.S.	SS27	22.24	25.56

Table S9. Summary of relative free energy calculation values of ClCl-alkyl complexes.

Complex	PDB Index	vacuum rel. free energy (kcal/mol)	DMSO rel. free energy (kcal/mol)
gem-ClCl-alkyl bromide ••• Br^-	SS28	4.50	5.04
gem-ClCl-alkyl bromide ••• I^-	SS29	6.33	4.58
gem-ClCl-alkyl iodide ••• Br^-	SS30	0.00	0.59
gem-ClCl-alkyl iodide ••• I^-	SS31	2.58	0.00
T.S.	SS32	23.54	25.15

In all of these cases, the expectation was supported by the calculations. The iodide complex halogen bonded to an iodide ion always presented as the lowest free energy state. The substrates with larger halogen atoms consistently have higher activation energy barriers, with the FBr combination having an activation energy barrier a little more than 2 kcal/mol higher than the FF case focused on in the main text. As there is experimental evidence for Finkelstein reaction occurrence for the FBr case, this series of compounds provides further evidence supporting the proposed mechanism in this study.

K.4. *Structural scan of reagents susceptible to Finkelstein S_N2 conversion from bromide to iodide form*

The above calculations support the formation of the *gem*-difluoroalkyl iodide complex as the thermodynamically preferred state, and they also indicate that the pathway to reaching this preferred state goes by way of the S_N2 mechanism. We can visualize the likelihood of such reactivity by mapping the nucleophilic frontier density as a function of different functional groups than the ester group.

In the visualized maps below, blue coloring shows sites for nucleophiles to attack and formation of the “next bond”. The sigma hole for the halogen bond is clear, and the presence of the ester opens the backside for S_N2 substitution. We can look at the likelihood for such an exchange process across a series of R-groups attached to the halogenated carbon center (see below). The key takeaway from the series of compounds below is that the Finkelstein reaction is an option if the R-group involves both (1) an sp^2-hybridized site bonded to the halogenated carbon center and (2) the lack of a competing site for a nucleophile to attack.

Some examples for $BrCF_2$-R structures:

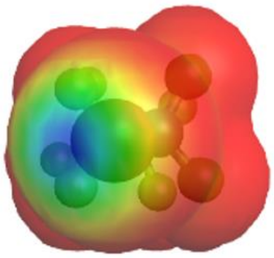

R = $–CF_2CF_2CF_3$
Bromine end

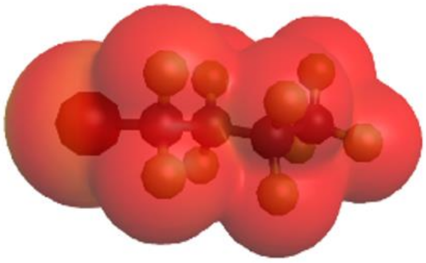

R = $–CF_2CF_2CF_3$
S_N2 backside

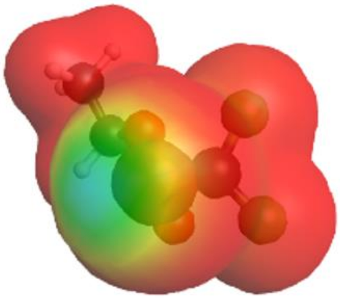

R = $–CO_2CH_2CH_3$
Bromine end

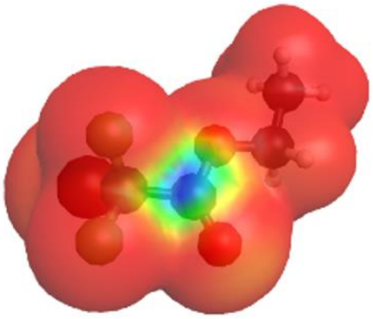

R = $–CO_2CH_2CH_3$
S_N2 backside

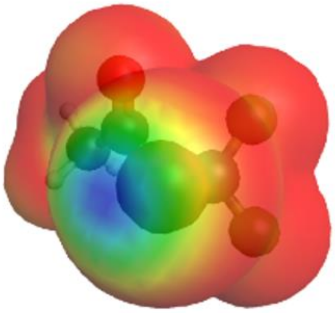

R = $-NHCOCH_3$
Bromine end

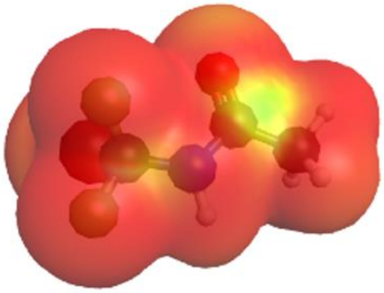

R = $-NHCOCH_3$
S_N2 backside

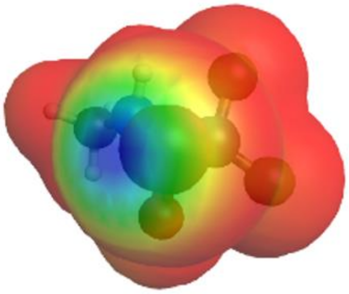

R = $-CONHCH_3$
Bromine end

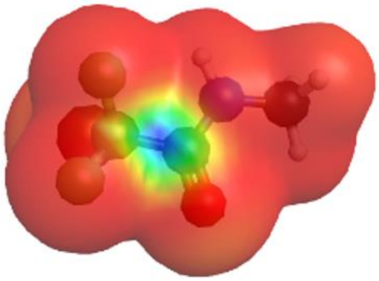

R = $-CONHCH_3$
S_N2 backside

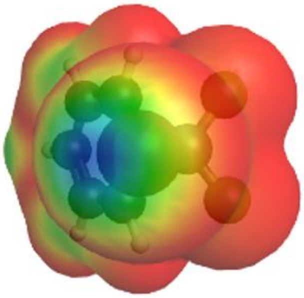

R = $-C_6H_5$
Bromine end

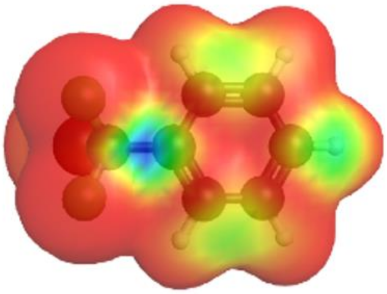

R = $-C_6H_5$
S_N2 backside

```
HEADER
REMARK Title: gem-difluoroalkyl bromide-br system
REMARK PDB Index: SS1
REMARK B3LYP/Def2QZVPP/GD3BJ
REMARK B3LYP Energy (Hartree): -5654.41479448
HETATM    1  C           1      -3.752  -1.414   0.444                          C
HETATM    2  C           1      -3.094  -0.339  -0.390                          C
HETATM    3  O           1      -1.903   0.073   0.304                          O
HETATM    4  C           1      -1.113   0.936  -0.343                          C
HETATM    5  O           1      -1.377   1.463  -1.391                          O
HETATM    6  C           1       0.211   1.109   0.393                          C
HETATM    7  F           1       0.002   1.227   1.734                          F
HETATM    8  F           1       0.804   2.254  -0.011                          F
HETATM    9 Br           1       1.380  -0.472   0.010                         Br
HETATM   10  H           1      -2.811  -0.704  -1.376                          H
HETATM   11  H           1      -3.741   0.528  -0.528                          H
HETATM   12  H           1      -4.660  -1.760  -0.050                          H
HETATM   13  H           1      -3.082  -2.261   0.573                          H
HETATM   14  H           1      -4.019  -1.033   1.429                          H
HETATM   15 Br           1       3.147  -2.860  -0.478                         Br
CONECT    1    2   12   13   14
CONECT    2    1    3   10   11
CONECT    3    2    4
CONECT    4    3    5    6
CONECT    5    4
CONECT    6    4    7    8    9
CONECT    7    6
CONECT    8    6
CONECT    9    6   15
CONECT   10    2
CONECT   11    2
CONECT   12    1
CONECT   13    1
CONECT   14    1
CONECT   15    9
END
```

```
HEADER
REMARK Title: gem-difluoroalkyl bromide-i system
REMARK PDB Index: SS2
REMARK B3LYP/Def2QZVPP/GD3BJ
REMARK B3LYP Energy (Hartree): -3377.98850074
HETATM    1  C           1      -3.547   3.037   0.553                          C
HETATM    2  C           1      -3.565   1.864  -0.398                          C
HETATM    3  O           1      -2.889   0.772   0.254                          O
HETATM    4  C           1      -2.689  -0.322  -0.486                          C
HETATM    5  O           1      -3.116  -0.499  -1.595                          O
HETATM    6  C           1      -1.772  -1.300   0.242                          C
HETATM    7 Br           1       0.122  -0.656   0.081                         Br
HETATM    8  F           1      -1.903  -2.530  -0.297                          F
HETATM    9  F           1      -2.118  -1.403   1.553                          F
HETATM   10  H           1      -4.579   1.551  -0.648                          H
HETATM   11  H           1      -3.044   2.084  -1.329                          H
HETATM   12  H           1      -4.045   3.891   0.095                          H
HETATM   13  H           1      -4.063   2.793   1.481                          H
HETATM   14  H           1      -2.524   3.320   0.793                          H
HETATM   15  I           1       3.193   0.399  -0.074                          I
CONECT    1    2   12   13   14
CONECT    2    1    3   10   11
CONECT    3    2    4
CONECT    4    3    5    6
CONECT    5    4
CONECT    6    4    7    8    9
CONECT    7    6   15
CONECT    8    6
CONECT    9    6
CONECT   10    2
CONECT   11    2
CONECT   12    1
CONECT   13    1
CONECT   14    1
CONECT   15    7
END
```

```
HEADER
REMARK Title: gem-difluoroalkyl iodide-br system
REMARK PDB Index: PDB Index: SS3
REMARK B3LYP/Def2QZVPP/GD3BJ
REMARK B3LYP Energy (Hartree): -3377.99353452
HETATM    1  C           1       3.574   2.879   0.561                          C
HETATM    2  C           1       3.445   1.728  -0.409                          C
HETATM    3  O           1       2.708   0.688   0.254                          O
HETATM    4  C           1       2.391  -0.379  -0.495                          C
HETATM    5  O           1       2.773  -0.560  -1.622                          O
HETATM    6  C           1       1.414  -1.278   0.235                          C
HETATM    7  F           1       1.765  -1.402   1.553                          F
HETATM    8  F           1       1.470  -2.528  -0.297                          F
HETATM    9  I           1      -0.666  -0.418   0.057                          I
HETATM   10  H           1       2.906   2.015  -1.312                          H
HETATM   11  H           1       4.417   1.337  -0.713                          H
HETATM   12  H           1       4.125   3.697   0.096                          H
HETATM   13  H           1       2.591   3.245   0.853                          H
HETATM   14  H           1       4.107   2.571   1.460                          H
HETATM   15 Br           1      -3.452   0.684  -0.096                         Br
CONECT    1    2   12   13   14
CONECT    2    1    3   10   11
CONECT    3    2    4
CONECT    4    3    5    6
CONECT    5    4
CONECT    6    4    7    8    9
CONECT    7    6
CONECT    8    6
CONECT    9    6   15
CONECT   10    2
CONECT   11    2
CONECT   12    1
CONECT   13    1
CONECT   14    1
CONECT   15    9
END
```

```
HEADER
REMARK Title: gem-difluoroalkyl iodide-i system
REMARK PDB Index: SS4
REMARK B3LYP/Def2QZVPP/GD3BJ
REMARK B3LYP Energy (Hartree): -1101.56602604
HETATM    1  C           1       -3.785  -1.394   0.453                          C
HETATM    2  C           1       -3.116  -0.336  -0.393                          C
HETATM    3  O           1       -1.949   0.108   0.319                          O
HETATM    4  C           1       -1.155   0.971  -0.332                          C
HETATM    5  O           1       -1.410   1.466  -1.399                          O
HETATM    6  C           1        0.150   1.167   0.412                          C
HETATM    7  F           1       -0.067   1.265   1.758                          F
HETATM    8  F           1        0.722   2.335   0.024                          F
HETATM    9  I           1        1.523  -0.581  -0.026                          I
HETATM   10  H           1       -2.805  -0.725  -1.362                          H
HETATM   11  H           1       -3.769   0.519  -0.573                          H
HETATM   12  H           1       -4.677  -1.764  -0.054                          H
HETATM   13  H           1       -3.111  -2.231   0.623                          H
HETATM   14  H           1       -4.080  -0.989   1.420                          H
HETATM   15  I           1        3.523  -3.063  -0.552                          I
CONECT    1    2   12   13   14
CONECT    2    1    3   10   11
CONECT    3    2    4
CONECT    4    3    5    6
CONECT    5    4
CONECT    6    4    7    8    9
CONECT    7    6
CONECT    8    6
CONECT    9    6   15
CONECT   10    2
CONECT   11    2
CONECT   12    1
CONECT   13    1
CONECT   14    1
CONECT   15    9
END
```

```
HEADER
REMARK Title: activated complex system
REMARK PDB Index: SS5
REMARK B3LYP/Def2QZVPP/GD3BJ
REMARK B3LYP Energy (Hartree): -3377.96578777
REMARK # Imaginary Frequencies: 1
REMARK Imaginary Frequency (cm^-1): -296.3080
HETATM    1  C           1       0.873   3.765  -0.684                          C
HETATM    2  C           1       0.763   2.741   0.422                          C
HETATM    3  O           1       0.566   1.465  -0.201                          O
HETATM    4  C           1       0.455   0.435   0.641                          C
HETATM    5  O           1       0.471   0.477   1.840                          O
HETATM    6  C           1       0.263  -0.835  -0.129                          C
HETATM    7  F           1       0.263  -0.923  -1.420                          F
HETATM    8  F           1       0.161  -1.964   0.487                          F
HETATM    9  I           1      -2.525  -0.256  -0.046                          I
HETATM   10  H           1       1.668   2.695   1.028                          H
HETATM   11  H           1      -0.081   2.941   1.083                          H
HETATM   12  H           1       1.714   3.536  -1.336                          H
HETATM   13  H           1      -0.036   3.781  -1.284                          H
HETATM   14  H           1       1.025   4.757  -0.256                          H
HETATM   15 Br           1       2.957  -0.860  -0.083                         Br
CONECT    1    2   12   13   14
CONECT    2    1    3   10   11
CONECT    3    2    4
CONECT    4    3    5    6
CONECT    5    4
CONECT    6    4    7    8
CONECT    7    6
CONECT    8    6
CONECT   10    2
CONECT   11    2
CONECT   12    1
CONECT   13    1
CONECT   14    1
END
```

```
HEADER
REMARK Title: difluoroalkyl anion
REMARK PDB Index: SS6
REMARK B3LYP/Def2QZVPP/GD3BJ
REMARK B3LYP Energy (Hartree): -505.892762160
HETATM    1  C           1      -3.747  -1.403   0.466                         C
HETATM    2  C           1      -3.062  -0.368  -0.399                         C
HETATM    3  O           1      -1.903   0.096   0.308                         O
HETATM    4  C           1      -1.133   1.034  -0.333                         C
HETATM    5  O           1      -1.501   1.549  -1.395                         O
HETATM    6  C           1       0.162   1.182   0.258                         C
HETATM    7  F           1       0.177   1.025   1.657                         F
HETATM    8  F           1       0.693   2.465   0.041                         F
HETATM    9  H           1      -2.754  -0.792  -1.357                         H
HETATM   10  H           1      -3.724   0.473  -0.609                         H
HETATM   11  H           1      -4.637  -1.777  -0.040                         H
HETATM   12  H           1      -3.085  -2.246   0.664                         H
HETATM   13  H           1      -4.052  -0.972   1.420                         H
CONECT    1    2   11   12   13
CONECT    2    1    3    9   10
CONECT    3    2    4
CONECT    4    3    5    6
CONECT    5    4
CONECT    6    4    7    8
CONECT    7    6
CONECT    8    6
CONECT    9    2
CONECT   10    2
CONECT   11    1
CONECT   12    1
CONECT   13    1
END
```

```
HEADER
REMARK Title: IBr
REMARK PDB Index: SS7
REMARK B3LYP/Def2QZVPP/GD3BJ
REMARK B3LYP Energy (Hartree): -2872.05581746
HETATM    1 Br           1       1.551  -0.748  -0.012                          Br
HETATM    2  I           1       3.007  -2.749  -0.359                           I
CONECT    1    2
CONECT    2    1
END
```

```
HEADER
REMARK Title: HH-Br•••Br-
REMARK PDB Index: SS8
REMARK B3LYP/Def2QZVPP/GD3BJ
REMARK B3LYP Energy (Hartree): -5455.94114053
HETATM    1  C           1      -3.855  -1.333   0.489                           C
HETATM    2  C           1      -3.122  -0.357  -0.399                           C
HETATM    3  O           1      -1.905   0.035   0.291                           O
HETATM    4  C           1      -1.097   0.883  -0.342                           C
HETATM    5  O           1      -1.328   1.374  -1.422                           O
HETATM    6  C           1       0.154   1.148   0.451                           C
HETATM    7  H           1      -0.024   1.129   1.518                           H
HETATM    8  H           1       0.614   2.074   0.135                           H
HETATM    9 Br           1       1.457  -0.291   0.075                          Br
HETATM   10  H           1      -2.842  -0.801  -1.352                           H
HETATM   11  H           1      -3.707   0.539  -0.595                           H
HETATM   12  H           1      -4.773  -1.648  -0.005                           H
HETATM   13  H           1      -3.249  -2.216   0.681                           H
HETATM   14  H           1      -4.118  -0.873   1.440                           H
HETATM   15 Br           1       3.789  -2.915  -0.647                          Br
CONECT    1    2   12   13   14
CONECT    2    1    3   10   11
CONECT    3    2    4
CONECT    4    3    5    6
CONECT    5    4
CONECT    6    4    7    8    9
CONECT    7    6
CONECT    8    6
CONECT    9    6   15
CONECT   10    2
CONECT   11    2
CONECT   12    1
CONECT   13    1
CONECT   14    1
CONECT   15    9
END
```

```
HEADER
REMARK Title: HH-Br•••I-
REMARK PDB Index: SS9
REMARK B3LYP/Def2QZVPP/GD3BJ
REMARK B3LYP Energy (Hartree): -3179.50925788
HETATM    1  C           1      -3.862  -1.335   0.470                          C
HETATM    2  C           1      -3.134  -0.344  -0.404                          C
HETATM    3  O           1      -1.917   0.041   0.290                          O
HETATM    4  C           1      -1.109   0.896  -0.334                          C
HETATM    5  O           1      -1.340   1.397  -1.410                          O
HETATM    6  C           1       0.140   1.156   0.462                          C
HETATM    7  H           1      -0.039   1.131   1.529                          H
HETATM    8  H           1       0.602   2.083   0.151                          H
HETATM    9 Br           1       1.446  -0.282   0.082                         Br
HETATM   10  H           1      -2.855  -0.772  -1.365                          H
HETATM   11  H           1      -3.722   0.553  -0.584                          H
HETATM   12  H           1      -4.782  -1.644  -0.025                          H
HETATM   13  H           1      -3.254  -2.221   0.647                          H
HETATM   14  H           1      -4.123  -0.892   1.430                          H
HETATM   15  I           1       3.941  -3.018  -0.618                          I
CONECT    1    2   12   13   14
CONECT    2    1    3   10   11
CONECT    3    2    4
CONECT    4    3    5    6
CONECT    5    4
CONECT    6    4    7    8    9
CONECT    7    6
CONECT    8    6
CONECT    9    6   15
CONECT   10    2
CONECT   11    2
CONECT   12    1
CONECT   13    1
CONECT   14    1
CONECT   15    9
END
```

```
HEADER
REMARK Title: HH-I•••Br-
REMARK PDB Index: SS10
REMARK B3LYP/Def2QZVPP/GD3BJ
REMARK B3LYP Energy (Hartree): -3179.51350037
HETATM    1  C           1      -3.888  -1.308   0.489                         C
HETATM    2  C           1      -3.140  -0.342  -0.397                         C
HETATM    3  O           1      -1.929   0.044   0.300                         O
HETATM    4  C           1      -1.102   0.880  -0.336                         C
HETATM    5  O           1      -1.330   1.357  -1.426                         O
HETATM    6  C           1       0.139   1.137   0.456                         C
HETATM    7  H           1      -0.033   1.090   1.523                         H
HETATM    8  H           1       0.598   2.069   0.158                         H
HETATM    9  I           1       1.607  -0.434   0.015                         I
HETATM   10  H           1      -2.859  -0.793  -1.347                         H
HETATM   11  H           1      -3.720   0.556  -0.602                         H
HETATM   12  H           1      -4.806  -1.619  -0.009                         H
HETATM   13  H           1      -3.290  -2.196   0.690                         H
HETATM   14  H           1      -4.154  -0.843   1.437                         H
HETATM   15 Br           1       3.901  -2.848  -0.632                        Br
CONECT    1    2   12   13   14
CONECT    2    1    3   10   11
CONECT    3    2    4
CONECT    4    3    5    6
CONECT    5    4
CONECT    6    4    7    8    9
CONECT    7    6
CONECT    8    6
CONECT    9    6   15
CONECT   10    2
CONECT   11    2
CONECT   12    1
CONECT   13    1
CONECT   14    1
CONECT   15    9
END
```

```
HEADER
REMARK Title: HH-I•••I-
REMARK PDB Index: SS11
REMARK B3LYP/Def2QZVPP/GD3BJ
REMARK B3LYP Energy (Hartree): -903.081491737
HETATM    1  C           1      -3.890  -1.312   0.481                         C
HETATM    2  C           1      -3.155  -0.327  -0.394                         C
HETATM    3  O           1      -1.940   0.055   0.300                         O
HETATM    4  C           1      -1.117   0.896  -0.334                         C
HETATM    5  O           1      -1.349   1.379  -1.421                         O
HETATM    6  C           1       0.125   1.152   0.455                         C
HETATM    7  H           1      -0.043   1.104   1.523                         H
HETATM    8  H           1       0.587   2.082   0.156                         H
HETATM    9  I           1       1.593  -0.423   0.017                         I
HETATM   10  H           1      -2.879  -0.760  -1.353                         H
HETATM   11  H           1      -3.741   0.571  -0.580                         H
HETATM   12  H           1      -4.811  -1.618  -0.013                         H
HETATM   13  H           1      -3.287  -2.200   0.661                         H
HETATM   14  H           1      -4.149  -0.864   1.440                         H
HETATM   15  I           1       4.047  -2.988  -0.615                         I
CONECT    1    2   12   13   14
CONECT    2    1    3   10   11
CONECT    3    2    4
CONECT    4    3    5    6
CONECT    5    4
CONECT    6    4    7    8    9
CONECT    7    6
CONECT    8    6
CONECT    9    6   15
CONECT   10    2
CONECT   11    2
CONECT   12    1
CONECT   13    1
CONECT   14    1
CONECT   15    9
END
```

```
HEADER
REMARK Title: HH-TS
REMARK PDB Index: SS12
REMARK B3LYP/Def2QZVPP/GD3BJ
REMARK B3LYP Energy (Hartree): -3179.49224205
REMARK # Imaginary Frequencies: 1
REMARK Imaginary Frequency (cm^-1): -357.1945
HETATM    1  C           1       0.863   3.801  -0.693                           C
HETATM    2  C           1       0.769   2.787   0.421                           C
HETATM    3  O           1       0.597   1.485  -0.186                           O
HETATM    4  C           1       0.472   0.447   0.645                           C
HETATM    5  O           1       0.510   0.529   1.852                           O
HETATM    6  C           1       0.296  -0.823  -0.100                           C
HETATM    7  H           1       0.265  -0.831  -1.169                           H
HETATM    8  H           1       0.171  -1.727   0.458                           H
HETATM    9  I           1      -2.403  -0.584  -0.149                           I
HETATM   10  H           1       1.670   2.766   1.031                           H
HETATM   11  H           1      -0.082   2.976   1.074                           H
HETATM   12  H           1       1.713   3.593  -1.340                           H
HETATM   13  H           1      -0.044   3.803  -1.296                           H
HETATM   14  H           1       0.992   4.796  -0.267                           H
HETATM   15 Br           1       2.750  -1.265  -0.219                          Br
CONECT    1    2   12   13   14
CONECT    2    1    3   10   11
CONECT    3    2    4
CONECT    4    3    5    6
CONECT    5    4
CONECT    6    4    7    8
CONECT    7    6
CONECT    8    6
CONECT   10    2
CONECT   11    2
CONECT   12    1
CONECT   13    1
CONECT   14    1
END
```

```
HEADER
REMARK Title: HF-Br•••Br-
REMARK PDB Index: SS13
REMARK B3LYP/Def2QZVPP/GD3BJ
REMARK B3LYP Energy (Hartree): -5555.22060529
HETATM    1  C           1      -3.824  -1.367   0.479                         C
HETATM    2  C           1      -3.127  -0.356  -0.397                         C
HETATM    3  O           1      -1.894   0.031   0.276                         O
HETATM    4  C           1      -1.115   0.900  -0.350                         C
HETATM    5  O           1      -1.350   1.443  -1.398                         O
HETATM    6  C           1       0.161   1.124   0.447                         C
HETATM    7  H           1      -0.010   1.136   1.517                         H
HETATM    8  F           1       0.749   2.291   0.063                         F
HETATM    9 Br           1       1.392  -0.376   0.084                        Br
HETATM   10  H           1      -2.864  -0.766  -1.369                         H
HETATM   11  H           1      -3.724   0.540  -0.547                         H
HETATM   12  H           1      -4.752  -1.676  -0.000                         H
HETATM   13  H           1      -3.204  -2.249   0.627                         H
HETATM   14  H           1      -4.066  -0.940   1.451                         H
HETATM   15 Br           1       3.618  -2.988  -0.562                        Br
CONECT    1    2   12   13   14
CONECT    2    1    3   10   11
CONECT    3    2    4
CONECT    4    3    5    6
CONECT    5    4
CONECT    6    4    7    8    9
CONECT    7    6
CONECT    8    6
CONECT    9    6   15
CONECT   10    2
CONECT   11    2
CONECT   12    1
CONECT   13    1
CONECT   14    1
CONECT   15    9
END
```

```
HEADER
REMARK Title: HF-Br•••I-
REMARK PDB Index: SS14
REMARK B3LYP/Def2QZVPP/GD3BJ
REMARK B3LYP Energy (Hartree): -3278.78864808
HETATM    1  C           1      -3.826  -1.369   0.483                           C
HETATM    2  C           1      -3.137  -0.356  -0.396                           C
HETATM    3  O           1      -1.905   0.041   0.273                           O
HETATM    4  C           1      -1.131   0.910  -0.357                           C
HETATM    5  O           1      -1.368   1.446  -1.408                           O
HETATM    6  C           1       0.143   1.146   0.439                           C
HETATM    7  H           1      -0.027   1.164   1.509                           H
HETATM    8  F           1       0.728   2.311   0.047                           F
HETATM    9 Br           1       1.383  -0.354   0.089                          Br
HETATM   10  H           1      -2.872  -0.766  -1.369                           H
HETATM   11  H           1      -3.740   0.537  -0.548                           H
HETATM   12  H           1      -4.753  -1.684   0.006                           H
HETATM   13  H           1      -3.200  -2.247   0.630                           H
HETATM   14  H           1      -4.068  -0.942   1.454                           H
HETATM   15  I           1       3.760  -3.089  -0.535                           I
CONECT    1    2   12   13   14
CONECT    2    1    3   10   11
CONECT    3    2    4
CONECT    4    3    5    6
CONECT    5    4
CONECT    6    4    7    8    9
CONECT    7    6
CONECT    8    6
CONECT    9    6   15
CONECT   10    2
CONECT   11    2
CONECT   12    1
CONECT   13    1
CONECT   14    1
CONECT   15    9
END
```

```
HEADER
REMARK Title: HF-I•••Br-
REMARK PDB Index: SS15
REMARK B3LYP/Def2QZVPP/GD3BJ
REMARK B3LYP Energy (Hartree): -3278.79119196
HETATM    1  C            1      -3.812  -1.377   0.493                          C
HETATM    2  C            1      -3.140  -0.351  -0.383                          C
HETATM    3  O            1      -1.912   0.056   0.281                          O
HETATM    4  C            1      -1.140   0.930  -0.358                          C
HETATM    5  O            1      -1.400   1.461  -1.409                          O
HETATM    6  C            1       0.136   1.160   0.413                          C
HETATM    7  H            1      -0.025   1.193   1.484                          H
HETATM    8  F            1       0.729   2.327   0.005                          F
HETATM    9  I            1       1.508  -0.522   0.043                          I
HETATM   10  H            1      -2.878  -0.753  -1.360                          H
HETATM   11  H            1      -3.757   0.534  -0.528                          H
HETATM   12  H            1      -4.738  -1.702   0.021                          H
HETATM   13  H            1      -3.175  -2.249   0.633                          H
HETATM   14  H            1      -4.054  -0.959   1.469                          H
HETATM   15 Br            1       3.649  -3.000  -0.485                         Br
CONECT    1    2   12   13   14
CONECT    2    1    3   10   11
CONECT    3    2    4
CONECT    4    3    5    6
CONECT    5    4
CONECT    6    4    7    8    9
CONECT    7    6
CONECT    8    6
CONECT    9    6   15
CONECT   10    2
CONECT   11    2
CONECT   12    1
CONECT   13    1
CONECT   14    1
CONECT   15    9
END
```

```
HEADER
REMARK Title: HF-I•••I-
REMARK PDB Index: SS16
REMARK B3LYP/Def2QZVPP/GD3BJ
REMARK B3LYP Energy (Hartree): -1002.35911651
HETATM    1  C           1      -3.816  -1.381   0.469                          C
HETATM    2  C           1      -3.144  -0.339  -0.390                          C
HETATM    3  O           1      -1.922   0.066   0.288                          O
HETATM    4  C           1      -1.151   0.951  -0.336                          C
HETATM    5  O           1      -1.408   1.494  -1.381                          O
HETATM    6  C           1       0.120   1.176   0.444                          C
HETATM    7  H           1      -0.043   1.192   1.515                          H
HETATM    8  F           1       0.714   2.346   0.052                          F
HETATM    9  I           1       1.497  -0.505   0.056                          I
HETATM   10  H           1      -2.874  -0.727  -1.370                          H
HETATM   11  H           1      -3.765   0.544  -0.527                          H
HETATM   12  H           1      -4.737  -1.705  -0.014                          H
HETATM   13  H           1      -3.174  -2.250   0.602                          H
HETATM   14  H           1      -4.067  -0.977   1.449                          H
HETATM   15  I           1       3.762  -3.138  -0.538                          I
CONECT    1    2   12   13   14
CONECT    2    1    3   10   11
CONECT    3    2    4
CONECT    4    3    5    6
CONECT    5    4
CONECT    6    4    7    8    9
CONECT    7    6
CONECT    8    6
CONECT    9    6   15
CONECT   10    2
CONECT   11    2
CONECT   12    1
CONECT   13    1
CONECT   14    1
CONECT   15    9
END
```

```
HEADER
REMARK Title: HF-TS
REMARK PDB Index: SS17
REMARK B3LYP/Def2QZVPP/GD3BJ
REMARK B3LYP Energy (Hartree): -3278.76569609
REMARK # Imaginary Frequencies: 1
REMARK Imaginary Frequency (cm^-1): -370.6498
HETATM    1  C           1       0.883   3.814  -0.693                           C
HETATM    2  C           1       0.759   2.796   0.414                           C
HETATM    3  O           1       0.589   1.494  -0.206                           O
HETATM    4  C           1       0.462   0.460   0.613                           C
HETATM    5  O           1       0.486   0.495   1.819                           O
HETATM    6  C           1       0.290  -0.815  -0.147                           C
HETATM    7  F           1       0.274  -0.796  -1.460                           F
HETATM    8  H           1       0.167  -1.757   0.343                           H
HETATM    9  I           1      -2.462  -0.624   0.137                           I
HETATM   10  H           1       1.648   2.760   1.041                           H
HETATM   11  H           1      -0.105   2.983   1.049                           H
HETATM   12  H           1       1.745   3.602  -1.322                           H
HETATM   13  H           1      -0.011   3.825  -1.314                           H
HETATM   14  H           1       1.011   4.804  -0.258                           H
HETATM   15 Br           1       2.802  -1.284   0.047                          Br
CONECT    1    2   12   13   14
CONECT    2    1    3   10   11
CONECT    3    2    4
CONECT    4    3    5    6
CONECT    5    4
CONECT    6    4    7    8
CONECT    7    6
CONECT    8    6
CONECT   10    2
CONECT   11    2
CONECT   12    1
CONECT   13    1
CONECT   14    1
END
```

```
HEADER
REMARK Title: FCl-Br•••Br-
REMARK PDB Index: SS18
REMARK B3LYP/Def2QZVPP/GD3BJ
REMARK B3LYP Energy (Hartree): -6014.85300469
HETATM    1  C           1      -3.932  -1.274   0.501                         C
HETATM    2  C           1      -3.126  -0.403  -0.428                         C
HETATM    3  O           1      -1.920   0.003   0.289                         O
HETATM    4  C           1      -1.068   0.762  -0.369                         C
HETATM    5  O           1      -1.191   1.183  -1.488                         O
HETATM    6  C           1       0.194   1.012   0.484                         C
HETATM    7  F           1      -0.071   0.895   1.805                         F
HETATM    8 Cl           1       0.841   2.635   0.184                        Cl
HETATM    9 Br           1       1.508  -0.387  -0.010                        Br
HETATM   10  H           1      -2.815  -0.932  -1.326                         H
HETATM   11  H           1      -3.660   0.498  -0.720                         H
HETATM   12  H           1      -4.839  -1.596  -0.009                         H
HETATM   13  H           1      -3.370  -2.159   0.793                         H
HETATM   14  H           1      -4.220  -0.727   1.398                         H
HETATM   15 Br           1       3.662  -2.763  -0.785                        Br
CONECT    1    2   12   13   14
CONECT    2    1    3   10   11
CONECT    3    2    4
CONECT    4    3    5    6
CONECT    5    4
CONECT    6    4    7    8    9
CONECT    7    6
CONECT    8    6
CONECT    9    6   15
CONECT   10    2
CONECT   11    2
CONECT   12    1
CONECT   13    1
CONECT   14    1
CONECT   15    9
END
```

```
HEADER
REMARK Title: FCl-Br•••I-
REMARK PDB Index: SS19
REMARK B3LYP/Def2QZVPP/GD3BJ
REMARK B3LYP Energy (Hartree): -3738.42099735
HETATM    1  C           1      -3.934  -1.279   0.496                           C
HETATM    2  C           1      -3.140  -0.390  -0.426                           C
HETATM    3  O           1      -1.932   0.014   0.288                           O
HETATM    4  C           1      -1.084   0.779  -0.368                           C
HETATM    5  O           1      -1.210   1.205  -1.484                           O
HETATM    6  C           1       0.178   1.030   0.484                           C
HETATM    7  F           1      -0.084   0.910   1.805                           F
HETATM    8 Cl           1       0.828   2.650   0.185                          Cl
HETATM    9 Br           1       1.496  -0.374  -0.010                          Br
HETATM   10  H           1      -2.831  -0.904  -1.334                           H
HETATM   11  H           1      -3.681   0.512  -0.702                           H
HETATM   12  H           1      -4.843  -1.598  -0.012                           H
HETATM   13  H           1      -3.365  -2.166   0.769                           H
HETATM   14  H           1      -4.217  -0.748   1.403                           H
HETATM   15  I           1       3.810  -2.892  -0.774                           I
CONECT    1    2   12   13   14
CONECT    2    1    3   10   11
CONECT    3    2    4
CONECT    4    3    5    6
CONECT    5    4
CONECT    6    4    7    8    9
CONECT    7    6
CONECT    8    6
CONECT    9    6   15
CONECT   10    2
CONECT   11    2
CONECT   12    1
CONECT   13    1
CONECT   14    1
CONECT   15    9
END
```

```
HEADER
REMARK Title: FCl-I•••Br-
REMARK PDB Index: SS20
REMARK B3LYP/Def2QZVPP/GD3BJ
REMARK B3LYP Energy (Hartree): -3738.42546907
HETATM    1  C           1      -3.929  -1.285   0.497                         C
HETATM    2  C           1      -3.144  -0.381  -0.419                         C
HETATM    3  O           1      -1.944   0.033   0.299                         O
HETATM    4  C           1      -1.092   0.800  -0.360                         C
HETATM    5  O           1      -1.232   1.217  -1.481                         O
HETATM    6  C           1       0.169   1.040   0.473                         C
HETATM    7  F           1      -0.092   0.935   1.806                         F
HETATM    8 Cl           1       0.818   2.667   0.167                        Cl
HETATM    9  I           1       1.637  -0.544  -0.069                         I
HETATM   10  H           1      -2.830  -0.886  -1.330                         H
HETATM   11  H           1      -3.698   0.514  -0.691                         H
HETATM   12  H           1      -4.834  -1.612  -0.013                         H
HETATM   13  H           1      -3.349  -2.167   0.765                         H
HETATM   14  H           1      -4.218  -0.764   1.407                         H
HETATM   15 Br           1       3.729  -2.819  -0.731                        Br
CONECT    1    2   12   13   14
CONECT    2    1    3   10   11
CONECT    3    2    4
CONECT    4    3    5    6
CONECT    5    4
CONECT    6    4    7    8    9
CONECT    7    6
CONECT    8    6
CONECT    9    6   15
CONECT   10    2
CONECT   11    2
CONECT   12    1
CONECT   13    1
CONECT   14    1
CONECT   15    9
END
```

```
HEADER
REMARK Title: FCl-I•••I-
REMARK PDB Index: SS21
REMARK B3LYP/Def2QZVPP/GD3BJ
REMARK B3LYP Energy (Hartree): -1461.99344460
HETATM    1  C            1      -3.950  -1.274   0.494                       C
HETATM    2  C            1      -3.146  -0.393  -0.428                       C
HETATM    3  O            1      -1.957   0.033   0.300                       O
HETATM    4  C            1      -1.103   0.801  -0.355                       C
HETATM    5  O            1      -1.232   1.207  -1.481                       O
HETATM    6  C            1       0.146   1.057   0.489                       C
HETATM    7  F            1      -0.121   0.943   1.819                       F
HETATM    8 Cl            1       0.781   2.689   0.190                      Cl
HETATM    9  I            1       1.646  -0.515  -0.049                       I
HETATM   10  H            1      -2.819  -0.921  -1.322                       H
HETATM   11  H            1      -3.693   0.497  -0.731                       H
HETATM   12  H            1      -4.846  -1.611  -0.025                       H
HETATM   13  H            1      -3.378  -2.150   0.794                       H
HETATM   14  H            1      -4.255  -0.730   1.386                       H
HETATM   15  I            1       3.917  -2.882  -0.763                       I
CONECT    1    2   12   13   14
CONECT    2    1    3   10   11
CONECT    3    2    4
CONECT    4    3    5    6
CONECT    5    4
CONECT    6    4    7    8    9
CONECT    7    6
CONECT    8    6
CONECT    9    6   15
CONECT   10    2
CONECT   11    2
CONECT   12    1
CONECT   13    1
CONECT   14    1
CONECT   15    9
END
```

```
HEADER
REMARK Title: FCl-TS
REMARK PDB Index: SS22
REMARK B3LYP/Def2QZVPP/GD3BJ
REMARK B3LYP Energy (Hartree): -3738.38903652
REMARK # Imaginary Frequencies: 1
REMARK Imaginary Frequency (cm^-1): -333.5019
HETATM    1  C           1       0.864   3.778  -0.692                          C
HETATM    2  C           1       0.753   2.767   0.421                          C
HETATM    3  O           1       0.594   1.459  -0.193                          O
HETATM    4  C           1       0.472   0.437   0.634                          C
HETATM    5  O           1       0.498   0.459   1.837                          O
HETATM    6  C           1       0.297  -0.849  -0.141                          C
HETATM    7  F           1       0.272  -0.859  -1.439                          F
HETATM    8 Cl           1       0.112  -2.337   0.632                         Cl
HETATM    9  I           1      -2.565  -0.067  -0.060                          I
HETATM   10  H           1       1.644   2.740   1.045                          H
HETATM   11  H           1      -0.112   2.947   1.056                          H
HETATM   12  H           1       1.727   3.570  -1.322                          H
HETATM   13  H           1      -0.032   3.776  -1.310                          H
HETATM   14  H           1       0.984   4.771  -0.263                          H
HETATM   15 Br           1       3.028  -0.837  -0.144                         Br
CONECT    1    2   12   13   14
CONECT    2    1    3   10   11
CONECT    3    2    4
CONECT    4    3    5    6
CONECT    5    4
CONECT    6    4    7    8
CONECT    7    6
CONECT    8    6
CONECT   10    2
CONECT   11    2
CONECT   12    1
CONECT   13    1
CONECT   14    1
END
```

```
HEADER
REMARK Title: FBr-Br•••Br-
REMARK PDB Index: SS23
REMARK B3LYP/Def2QZVPP/GD3BJ
REMARK B3LYP Energy (Hartree): -8128.84666478
HETATM    1  C           1      -4.043  -1.173   0.525                           C
HETATM    2  C           1      -3.152  -0.422  -0.430                           C
HETATM    3  O           1      -1.942  -0.052   0.299                           O
HETATM    4  C           1      -1.030   0.623  -0.371                           C
HETATM    5  O           1      -1.096   0.979  -1.517                           O
HETATM    6  C           1       0.210   0.877   0.508                           C
HETATM    7  F           1      -0.044   0.647   1.814                           F
HETATM    8 Br           1       0.771   2.745   0.329                          Br
HETATM    9 Br           1       1.629  -0.364  -0.076                          Br
HETATM   10  H           1      -2.853  -1.028  -1.283                           H
HETATM   11  H           1      -3.613   0.492  -0.796                           H
HETATM   12  H           1      -4.956  -1.464   0.007                           H
HETATM   13  H           1      -3.554  -2.075   0.889                           H
HETATM   14  H           1      -4.315  -0.552   1.376                           H
HETATM   15 Br           1       3.981  -2.486  -0.956                          Br
CONECT    1    2   12   13   14
CONECT    2    1    3   10   11
CONECT    3    2    4
CONECT    4    3    5    6
CONECT    5    4
CONECT    6    4    7    8    9
CONECT    7    6
CONECT    8    6
CONECT    9    6   15
CONECT   10    2
CONECT   11    2
CONECT   12    1
CONECT   13    1
CONECT   14    1
CONECT   15    9
END
```

```
HEADER
REMARK Title: FBr-Br•••I-
REMARK PDB Index: SS24
REMARK B3LYP/Def2QZVPP/GD3BJ
REMARK B3LYP Energy (Hartree): -5852.41462793
HETATM    1  C           1      -4.063  -1.158   0.512                       C
HETATM    2  C           1      -3.158  -0.424  -0.443                       C
HETATM    3  O           1      -1.956  -0.044   0.298                       O
HETATM    4  C           1      -1.037   0.623  -0.369                       C
HETATM    5  O           1      -1.087   0.964  -1.521                       O
HETATM    6  C           1       0.191   0.891   0.523                       C
HETATM    7  F           1      -0.075   0.667   1.827                       F
HETATM    8 Br           1       0.748   2.758   0.335                      Br
HETATM    9 Br           1       1.626  -0.352  -0.037                      Br
HETATM   10  H           1      -2.847  -1.043  -1.282                       H
HETATM   11  H           1      -3.612   0.486  -0.829                       H
HETATM   12  H           1      -4.969  -1.456  -0.014                       H
HETATM   13  H           1      -3.580  -2.055   0.896                       H
HETATM   14  H           1      -4.345  -0.523   1.349                       H
HETATM   15  I           1       4.155  -2.589  -0.924                       I
CONECT    1    2   12   13   14
CONECT    2    1    3   10   11
CONECT    3    2    4
CONECT    4    3    5    6
CONECT    5    4
CONECT    6    4    7    8    9
CONECT    7    6
CONECT    8    6
CONECT    9    6   15
CONECT   10    2
CONECT   11    2
CONECT   12    1
CONECT   13    1
CONECT   14    1
CONECT   15    9
END
```

```
HEADER
REMARK Title: FBr-I•••Br-
REMARK PDB Index: SS25
REMARK B3LYP/Def2QZVPP/GD3BJ
REMARK B3LYP Energy (Hartree): -5852.41937799
HETATM    1  C            1      -4.024  -1.204   0.525                         C
HETATM    2  C            1      -3.153  -0.427  -0.428                         C
HETATM    3  O            1      -1.960  -0.017   0.304                         O
HETATM    4  C            1      -1.063   0.686  -0.366                         C
HETATM    5  O            1      -1.154   1.039  -1.514                         O
HETATM    6  C            1       0.174   0.948   0.494                         C
HETATM    7  F            1      -0.091   0.775   1.816                         F
HETATM    8 Br            1       0.765   2.803   0.250                        Br
HETATM    9  I            1       1.744  -0.505  -0.099                         I
HETATM   10  H            1      -2.832  -1.025  -1.278                         H
HETATM   11  H            1      -3.644   0.470  -0.799                         H
HETATM   12  H            1      -4.924  -1.527   0.004                         H
HETATM   13  H            1      -3.506  -2.088   0.895                         H
HETATM   14  H            1      -4.321  -0.590   1.373                         H
HETATM   15 Br            1       3.981  -2.591  -0.858                        Br
CONECT    1    2   12   13   14
CONECT    2    1    3   10   11
CONECT    3    2    4
CONECT    4    3    5    6
CONECT    5    4
CONECT    6    4    7    8    9
CONECT    7    6
CONECT    8    6
CONECT    9    6   15
CONECT   10    2
CONECT   11    2
CONECT   12    1
CONECT   13    1
CONECT   14    1
CONECT   15    9
END
```

```
HEADER
REMARK Title: FBr-I•••I-
REMARK PDB Index: SS26
REMARK B3LYP/Def2QZVPP/GD3BJ
REMARK B3LYP Energy (Hartree): -3575.98734707
HETATM    1  C           1      -4.006  -1.227   0.516                          C
HETATM    2  C           1      -3.158  -0.413  -0.427                          C
HETATM    3  O           1      -1.971   0.009   0.306                          O
HETATM    4  C           1      -1.083   0.728  -0.362                          C
HETATM    5  O           1      -1.182   1.087  -1.507                          O
HETATM    6  C           1       0.154   0.992   0.495                          C
HETATM    7  F           1      -0.112   0.837   1.819                          F
HETATM    8 Br           1       0.777   2.830   0.225                         Br
HETATM    9  I           1       1.709  -0.503  -0.079                          I
HETATM   10  H           1      -2.829  -0.988  -1.290                          H
HETATM   11  H           1      -3.671   0.479  -0.779                          H
HETATM   12  H           1      -4.902  -1.560  -0.006                          H
HETATM   13  H           1      -3.467  -2.106   0.866                          H
HETATM   14  H           1      -4.311  -0.635   1.377                          H
HETATM   15  I           1       4.043  -2.780  -0.836                          I
CONECT    1    2   12   13   14
CONECT    2    1    3   10   11
CONECT    3    2    4
CONECT    4    3    5    6
CONECT    5    4
CONECT    6    4    7    8    9
CONECT    7    6
CONECT    8    6
CONECT    9    6   15
CONECT   10    2
CONECT   11    2
CONECT   12    1
CONECT   13    1
CONECT   14    1
CONECT   15    9
END
```

```
HEADER
REMARK Title: FBr-TS
REMARK PDB Index: SS27
REMARK B3LYP/Def2QZVPP/GD3BJ
REMARK B3LYP Energy (Hartree): -5852.38064744
REMARK # Imaginary Frequencies: 1
REMARK Imaginary Frequency (cm^-1): -336.7053
HETATM    1  C           1       1.401   3.516  -0.282                           C
HETATM    2  C           1       0.093   2.900   0.160                           C
HETATM    3  O           1      -0.035   1.531  -0.320                           O
HETATM    4  C           1       0.518   0.585   0.419                           C
HETATM    5  O           1       1.141   0.717   1.439                           O
HETATM    6  C           1       0.261  -0.776  -0.194                           C
HETATM    7  F           1      -0.414  -0.900  -1.296                           F
HETATM    8 Br           1       0.888  -2.348   0.588                          Br
HETATM    9  I           1      -2.255  -0.596   1.361                           I
HETATM   10  H           1      -0.008   2.892   1.242                           H
HETATM   11  H           1      -0.761   3.407  -0.278                           H
HETATM   12  H           1       2.249   2.992   0.151                           H
HETATM   13  H           1       1.492   3.495  -1.367                           H
HETATM   14  H           1       1.433   4.555   0.046                           H
HETATM   15 Br           1       2.533  -0.214  -1.606                          Br
CONECT    1    2   12   13   14
CONECT    2    1    3   10   11
CONECT    3    2    4
CONECT    4    3    5    6
CONECT    5    4
CONECT    6    4    7    8
CONECT    7    6
CONECT    8    6
CONECT   10    2
CONECT   11    2
CONECT   12    1
CONECT   13    1
CONECT   14    1
END
```

```
HEADER
REMARK Title: ClCl-Br•••Br-
REMARK PDB Index: SS28
REMARK B3LYP/Def2QZVPP/GD3BJ
REMARK B3LYP Energy (Hartree): -6375.20270674
HETATM    1  C           1      -3.765  -1.426   0.445                         C
HETATM    2  C           1      -3.110  -0.376  -0.414                         C
HETATM    3  O           1      -1.868   0.013   0.244                         O
HETATM    4  C           1      -1.123   0.910  -0.371                         C
HETATM    5  O           1      -1.384   1.461  -1.406                         O
HETATM    6  C           1       0.203   1.136   0.391                         C
HETATM    7 Cl           1      -0.075   1.290   2.144                        Cl
HETATM    8 Cl           1       1.016   2.592  -0.206                        Cl
HETATM    9 Br           1       1.337  -0.465   0.019                        Br
HETATM   10  H           1      -2.860  -0.749  -1.405                         H
HETATM   11  H           1      -3.726   0.515  -0.521                         H
HETATM   12  H           1      -4.698  -1.738  -0.022                         H
HETATM   13  H           1      -3.125  -2.300   0.549                         H
HETATM   14  H           1      -3.992  -1.035   1.436                         H
HETATM   15 Br           1       3.164  -3.078  -0.564                        Br
CONECT    1    2   12   13   14
CONECT    2    1    3   10   11
CONECT    3    2    4
CONECT    4    3    5    6
CONECT    5    4
CONECT    6    4    7    8    9
CONECT    7    6
CONECT    8    6
CONECT    9    6   15
CONECT   10    2
CONECT   11    2
CONECT   12    1
CONECT   13    1
CONECT   14    1
CONECT   15    9
END
```

```
HEADER
REMARK Title: ClCl-Br•••I-
REMARK PDB Index: SS29
REMARK B3LYP/Def2QZVPP/GD3BJ
REMARK B3LYP Energy (Hartree): -4098.77078314
HETATM    1  C           1      -3.782  -1.413   0.452                          C
HETATM    2  C           1      -3.109  -0.386  -0.421                          C
HETATM    3  O           1      -1.881   0.021   0.252                          O
HETATM    4  C           1      -1.131   0.912  -0.366                          C
HETATM    5  O           1      -1.378   1.446  -1.414                          O
HETATM    6  C           1       0.184   1.155   0.409                          C
HETATM    7 Cl           1      -0.101   1.290   2.161                         Cl
HETATM    8 Cl           1       0.983   2.622  -0.175                         Cl
HETATM    9 Br           1       1.342  -0.437   0.026                         Br
HETATM   10  H           1      -2.838  -0.785  -1.396                          H
HETATM   11  H           1      -3.722   0.501  -0.565                          H
HETATM   12  H           1      -4.705  -1.739  -0.026                          H
HETATM   13  H           1      -3.143  -2.283   0.594                          H
HETATM   14  H           1      -4.030  -0.995   1.427                          H
HETATM   15  I           1       3.304  -3.161  -0.640                          I
CONECT    1    2   12   13   14
CONECT    2    1    3   10   11
CONECT    3    2    4
CONECT    4    3    5    6
CONECT    5    4
CONECT    6    4    7    8    9
CONECT    7    6
CONECT    8    6
CONECT    9    6   15
CONECT   10    2
CONECT   11    2
CONECT   12    1
CONECT   13    1
CONECT   14    1
CONECT   15    9
END
```

```
HEADER
REMARK Title: ClCl-I•••Br-
REMARK PDB Index: SS30
REMARK B3LYP/Def2QZVPP/GD3BJ
REMARK B3LYP Energy (Hartree): -4098.77761208
HETATM    1  C           1      -3.819  -1.379   0.477                          C
HETATM    2  C           1      -3.114  -0.390  -0.417                          C
HETATM    3  O           1      -1.908   0.039   0.276                          O
HETATM    4  C           1      -1.128   0.899  -0.360                          C
HETATM    5  O           1      -1.358   1.386  -1.437                          O
HETATM    6  C           1       0.171   1.145   0.411                          C
HETATM    7 Cl           1      -0.102   1.246   2.173                         Cl
HETATM    8 Cl           1       0.944   2.646  -0.147                         Cl
HETATM    9  I           1       1.502  -0.611  -0.060                          I
HETATM   10  H           1      -2.818  -0.830  -1.367                          H
HETATM   11  H           1      -3.721   0.491  -0.616                          H
HETATM   12  H           1      -4.729  -1.721  -0.015                          H
HETATM   13  H           1      -3.189  -2.245   0.673                          H
HETATM   14  H           1      -4.094  -0.921   1.425                          H
HETATM   15 Br           1       3.354  -3.005  -0.693                         Br
CONECT    1    2   12   13   14
CONECT    2    1    3   10   11
CONECT    3    2    4
CONECT    4    3    5    6
CONECT    5    4
CONECT    6    4    7    8    9
CONECT    7    6
CONECT    8    6
CONECT    9    6   15
CONECT   10    2
CONECT   11    2
CONECT   12    1
CONECT   13    1
CONECT   14    1
CONECT   15    9
END
```

```
HEADER
REMARK Title: ClCl-I•••I-
REMARK PDB Index: SS31
REMARK B3LYP/Def2QZVPP/GD3BJ
REMARK B3LYP Energy (Hartree): -1822.34575878
HETATM    1  C           1      -3.824  -1.379   0.474                         C
HETATM    2  C           1      -3.121  -0.388  -0.419                         C
HETATM    3  O           1      -1.922   0.051   0.279                         O
HETATM    4  C           1      -1.141   0.911  -0.357                         C
HETATM    5  O           1      -1.367   1.387  -1.441                         O
HETATM    6  C           1       0.149   1.169   0.418                         C
HETATM    7 Cl           1      -0.124   1.261   2.178                        Cl
HETATM    8 Cl           1       0.918   2.671  -0.138                        Cl
HETATM    9  I           1       1.502  -0.593  -0.055                         I
HETATM   10  H           1      -2.816  -0.830  -1.366                         H
HETATM   11  H           1      -3.732   0.487  -0.625                         H
HETATM   12  H           1      -4.729  -1.728  -0.022                         H
HETATM   13  H           1      -3.190  -2.240   0.676                         H
HETATM   14  H           1      -4.106  -0.920   1.420                         H
HETATM   15  I           1       3.495  -3.111  -0.700                         I
CONECT    1    2   12   13   14
CONECT    2    1    3   10   11
CONECT    3    2    4
CONECT    4    3    5    6
CONECT    5    4
CONECT    6    4    7    8    9
CONECT    7    6
CONECT    8    6
CONECT    9    6   15
CONECT   10    2
CONECT   11    2
CONECT   12    1
CONECT   13    1
CONECT   14    1
CONECT   15    9
END
```

```
HEADER
REMARK Title: ClCl-TS
REMARK PDB Index: SS32
REMARK B3LYP/Def2QZVPP/GD3BJ
REMARK B3LYP Energy (Hartree): -4098.73947933
REMARK # Imaginary Frequencies: 1
REMARK Imaginary Frequency (cm^-1): -276.0769
HETATM    1  C           1       0.870   3.768  -0.716                         C
HETATM    2  C           1       0.750   2.769   0.407                         C
HETATM    3  O           1       0.599   1.456  -0.194                         O
HETATM    4  C           1       0.471   0.442   0.642                         C
HETATM    5  O           1       0.489   0.488   1.845                         O
HETATM    6  C           1       0.303  -0.865  -0.114                         C
HETATM    7 Cl           1       0.265  -0.966  -1.806                        Cl
HETATM    8 Cl           1       0.108  -2.298   0.765                        Cl
HETATM    9  I           1      -2.623  -0.005   0.098                         I
HETATM   10  H           1       1.635   2.751   1.040                         H
HETATM   11  H           1      -0.122   2.955   1.032                         H
HETATM   12  H           1       1.740   3.554  -1.336                         H
HETATM   13  H           1      -0.019   3.758  -1.343                         H
HETATM   14  H           1       0.985   4.767  -0.297                         H
HETATM   15 Br           1       3.089  -0.818   0.037                        Br
CONECT    1    2   12   13   14
CONECT    2    1    3   10   11
CONECT    3    2    4
CONECT    4    3    5    6
CONECT    5    4
CONECT    6    4    7    8
CONECT    7    6
CONECT    8    6
CONECT   10    2
CONECT   11    2
CONECT   12    1
CONECT   13    1
CONECT   14    1
END
```

L. References

(1) Benesi, H. A.; Hildebrand, J. H. A Spectrophotometric Investigation of the Interaction of Iodine with Aromatic Hydrocarbons. *J. Am. Chem. Soc*. **1949**, *71*, 2703-2707.

(2) Grimme, S.; Antony, J.; Ehrlich, S.; Krieg, H. A consistent and accurate ab initio parametrization of density functional dispersion correction (DFT-D) for the 94 elements H-Pu. *J. Chem. Phys.* **2010**, *132*, 154104.

(3) M. J. Frisch, G. W. T., H. B. Schlegel, G. E. Scuseria, M. A. Robb, J. R. Cheeseman, G. Scalmani, V. Barone, G. A. Petersson, H. Nakatsuji, X. Li, M. Caricato, A. V. Marenich, ; J. Bloino, B. G. J., R. Gomperts, B. Mennucci, H. P. Hratchian, J. V. Ortiz, A. F. Izmaylov, J. L. Sonnenberg, D. Williams-Young, F. Ding, F. Lipparini, F. Egidi, J. Goings, B. Peng, A. Petrone, T. Henderson, D. Ranasinghe, V. G. Zakrzewski, J. Gao, N. Rega, G. Zheng, W. Liang, M. Hada, M. Ehara, K. Toyota, R. Fukuda, J. Hasegawa, M. Ishida, T. Nakajima, Y. Honda, O. Kitao, H. Nakai, ; T. Vreven, K. T., J. A. Montgomery, Jr., J. E. Peralta, F. Ogliaro, M. J. Bearpark, J. J. Heyd, E. N. Brothers, K. N. Kudin, V. N. Staroverov, T. A. Keith, R. Kobayashi, J. Normand, K. Raghavachari, A. P. Rendell, J. C. Burant, S. S. Iyengar, J. Tomasi, M. Cossi, J. M. Millam, M. Klene, C. Adamo, R. Cammi, J. W. Ochterski, R. L. Martin, K. Morokuma, O. Farkas, J. B. Foresman, and D. J. Fox. Gaussian 16, Revision C.02. Gaussian, Inc.: Wallingford CT, **2019**.

(4) Stephens, P. J.; Devlin, F. J.; Chabalowski, C. F.; Frisch, M. J. Ab Initio Calculation of Vibrational Absorption and Circular Dichroism Spectra Using Density Functional Force Fields. *J. Phys. Chem.* **1994**, *98*, 11623-11627.

(5) Zhao, Y.; Truhlar, D. G. The M06 suite of density functionals for main group thermochemistry, thermochemical kinetics, noncovalent interactions, excited states, and transition elements: two new functionals and systematic testing of four M06-class functionals and 12 other functionals. *Theor. Chem. Acc.* **2008**, *120*, 215-241.

(6) Weigend, F.; Ahlrichs, R. Balanced basis sets of split valence, triple zeta valence and quadruple zeta valence quality for H to Rn: Design and assessment of accuracy. *Phys. Chem. Chem. Phys.* **2005**, *7*, 3297-3305.

M. NMR Spectra

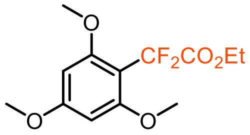

Ethyl 2,2-difluoro-2-(2,4,6-trimethoxyphenyl)acetate (3): ^{1}H NMR (400 MHz, $CDCl_3$)

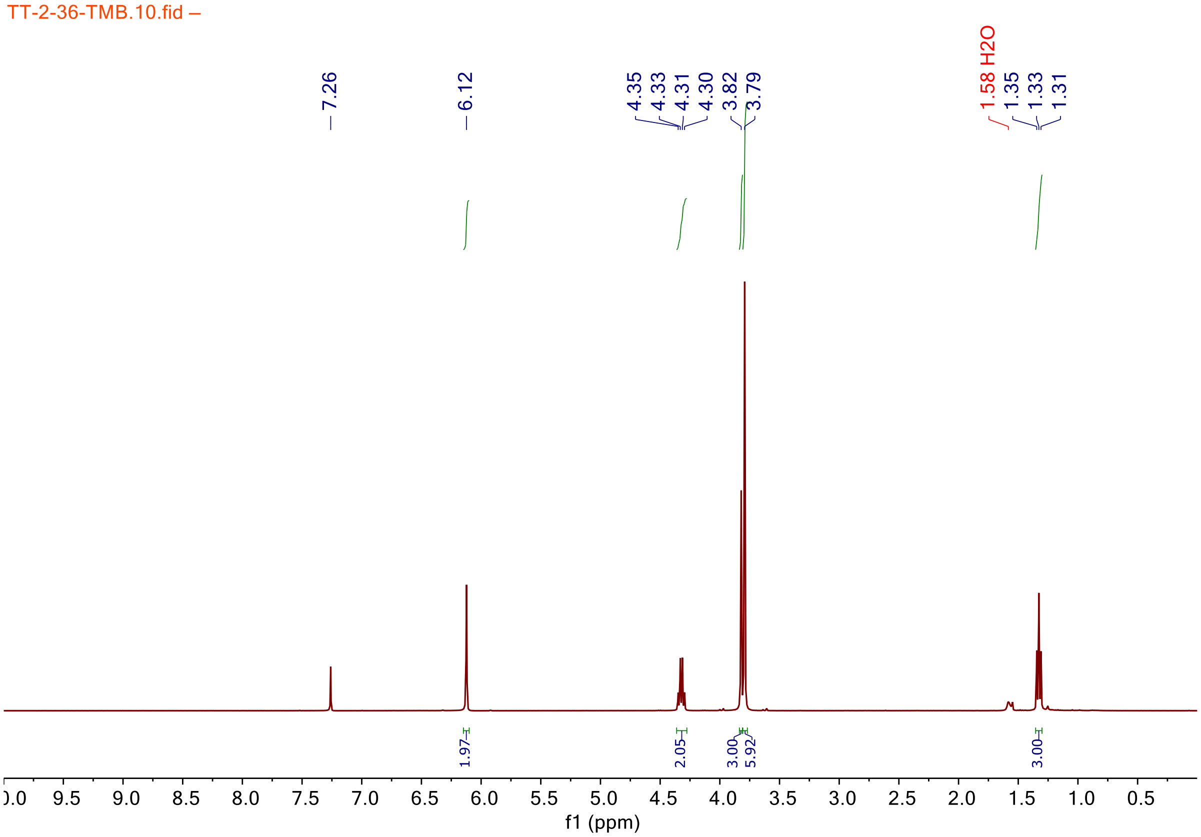

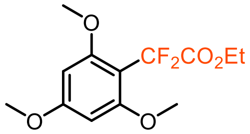

Ethyl 2,2-difluoro-2-(2,4,6-trimethoxyphenyl)acetate (3): $^{13}C\{^{1}H\}$ NMR (201 MHz, $CDCl_3$)

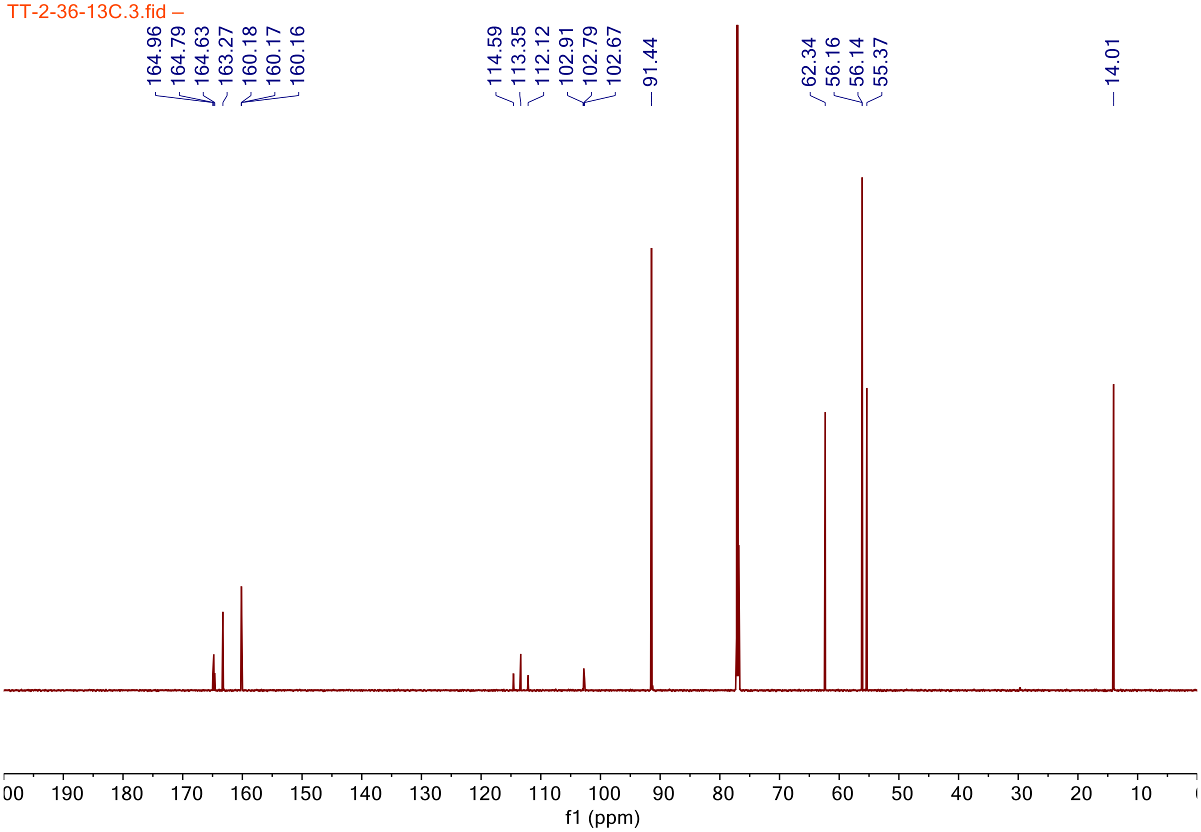

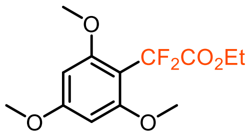

Ethyl 2,2-difluoro-2-(2,4,6-trimethoxyphenyl)acetate (3): ^{19}F NMR (376 MHz, $CDCl_3$)

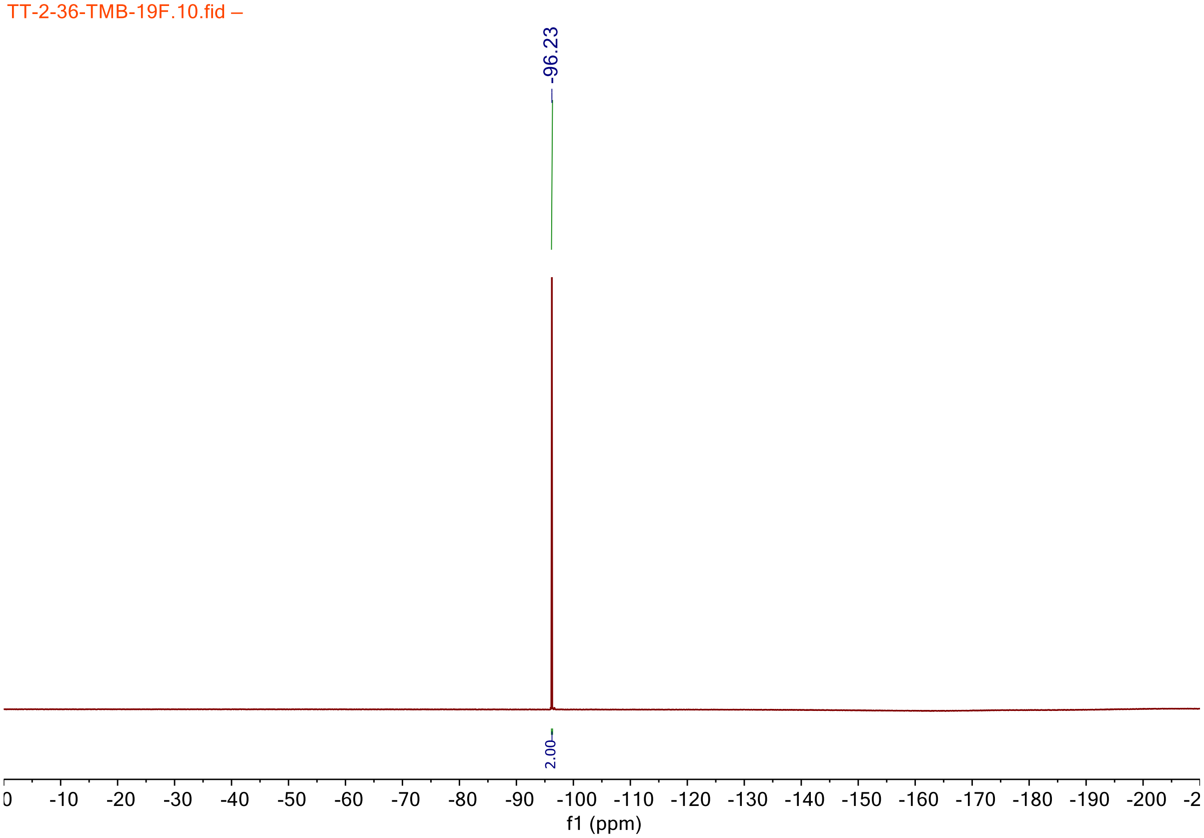

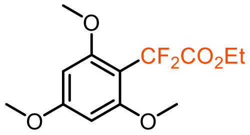

Ethyl 2,2-difluoro-2-(2,4,6-trimethoxyphenyl)acetate (3): ^{19}F NMR (376 MHz, $CDCl_3$) for control reaction without Bu_4NI

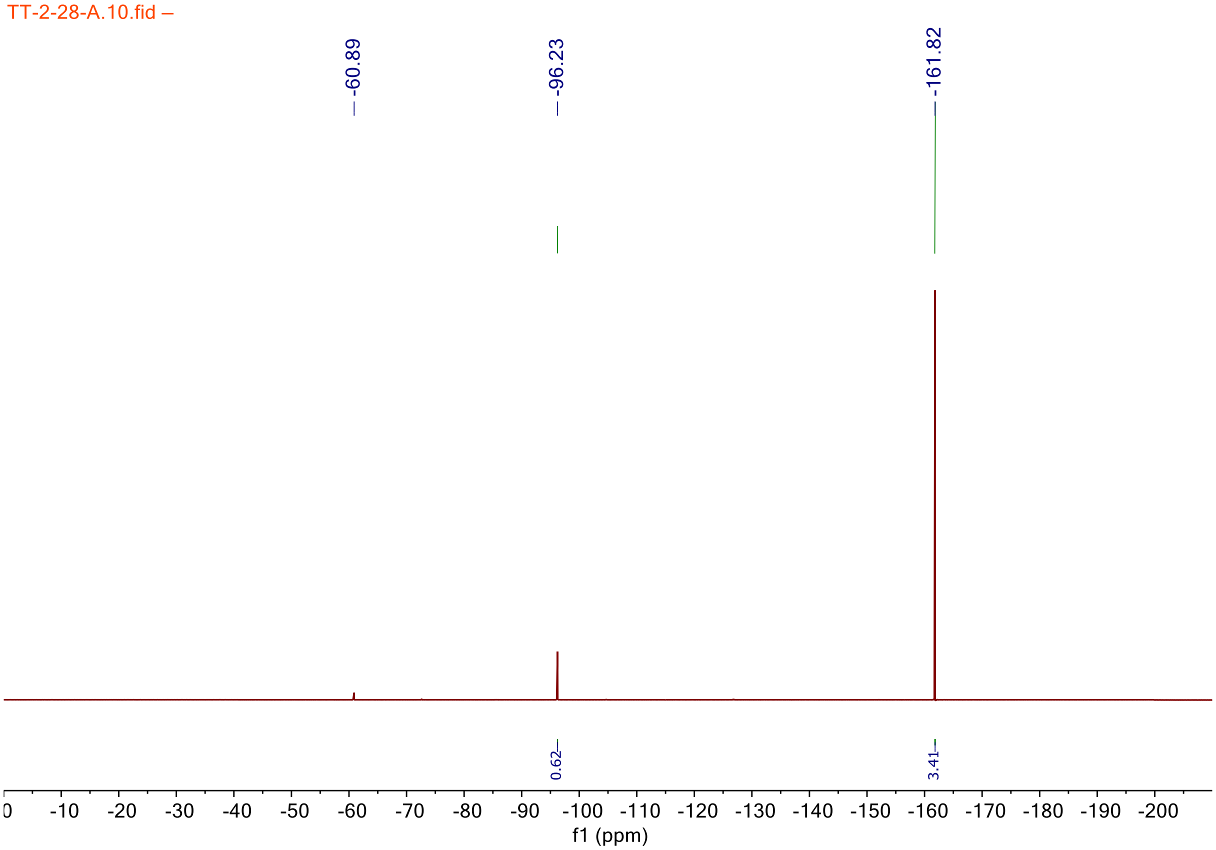

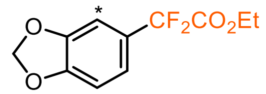

Ethyl 2-(benzo[*d*][1,3]dioxol-5-yl)-2,2-difluoroacetate (4): ^{1}H NMR (400 MHz, $CDCl_3$)

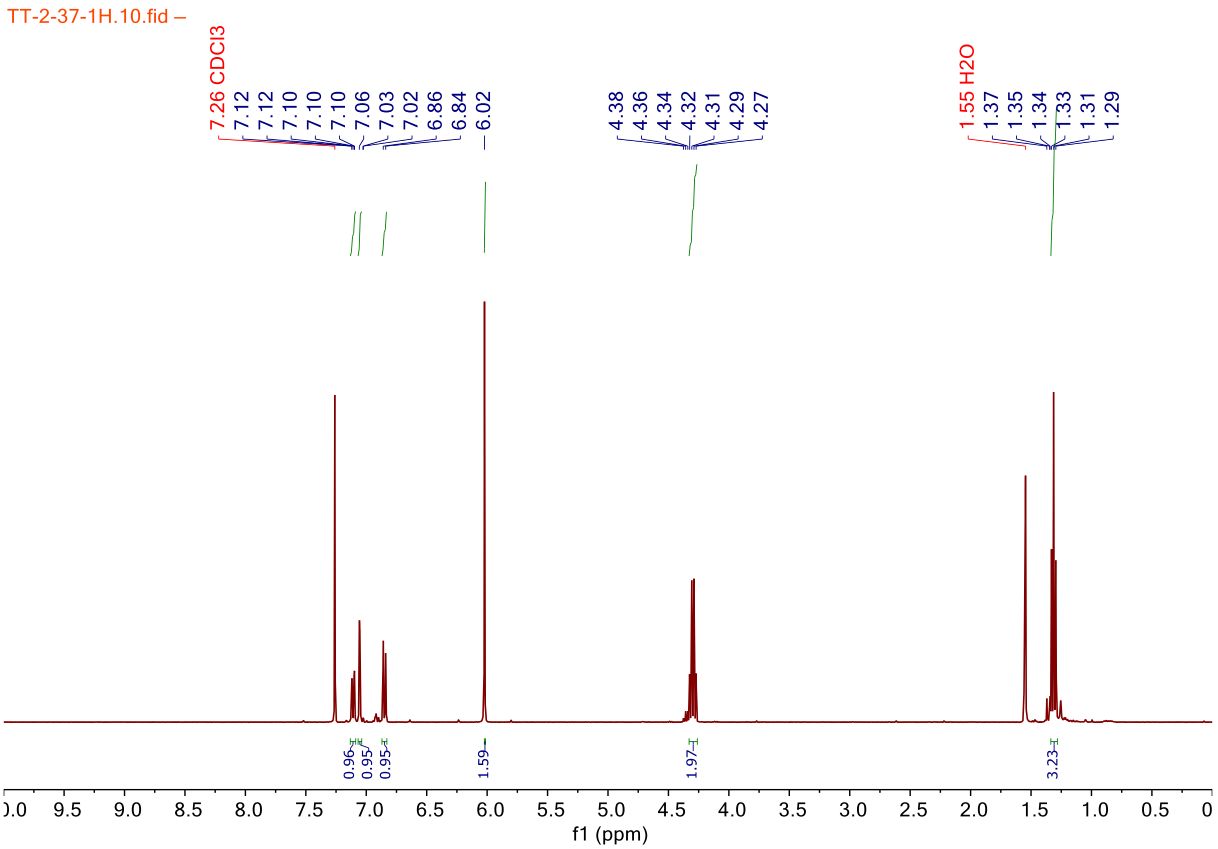

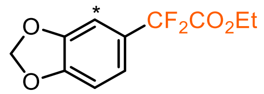

Ethyl 2-(benzo[*d*][1,3]dioxol-5-yl)-2,2-difluoroacetate (4): $^{13}C\{^{1}H\}$ NMR (201 MHz, $CDCl_3$)

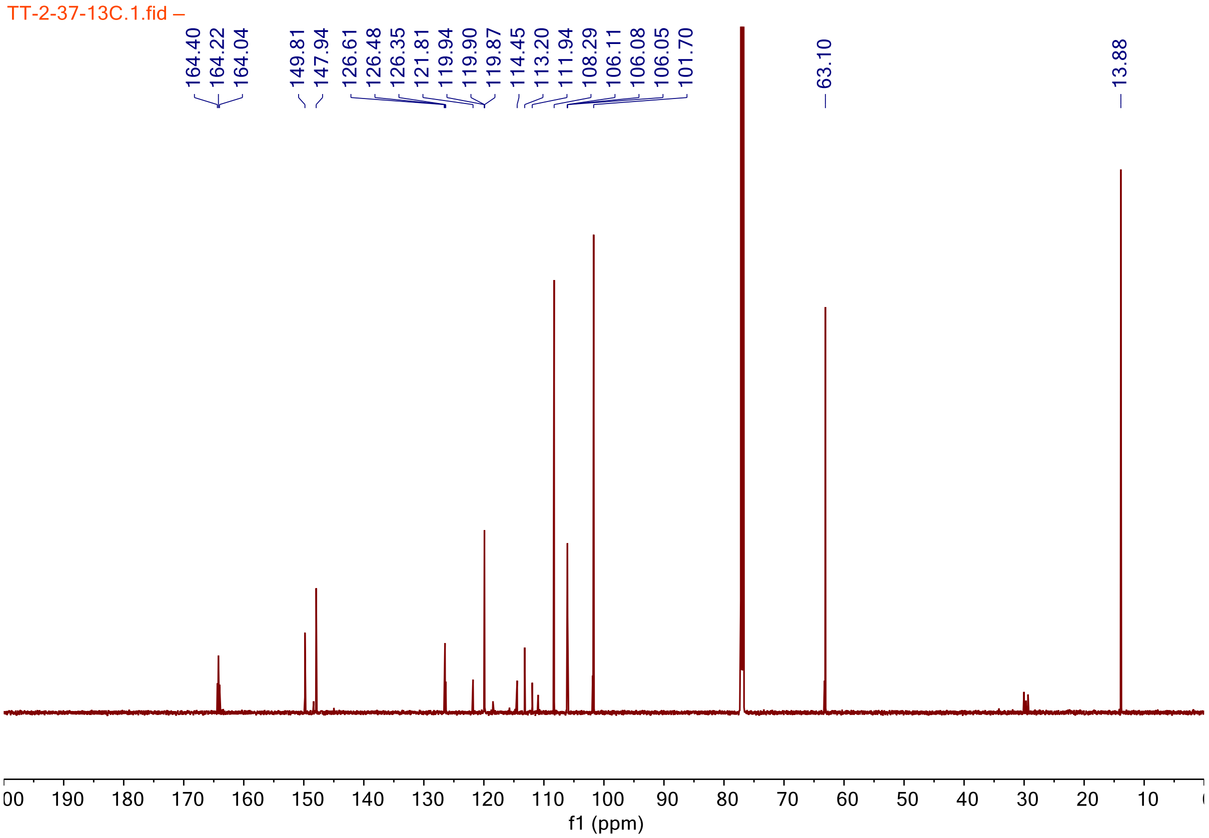

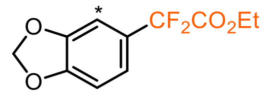

Ethyl 2-(benzo[*d*][1,3]dioxol-5-yl)-2,2-difluoroacetate (4): ^{19}F NMR (376 MHz, $CDCl_3$)

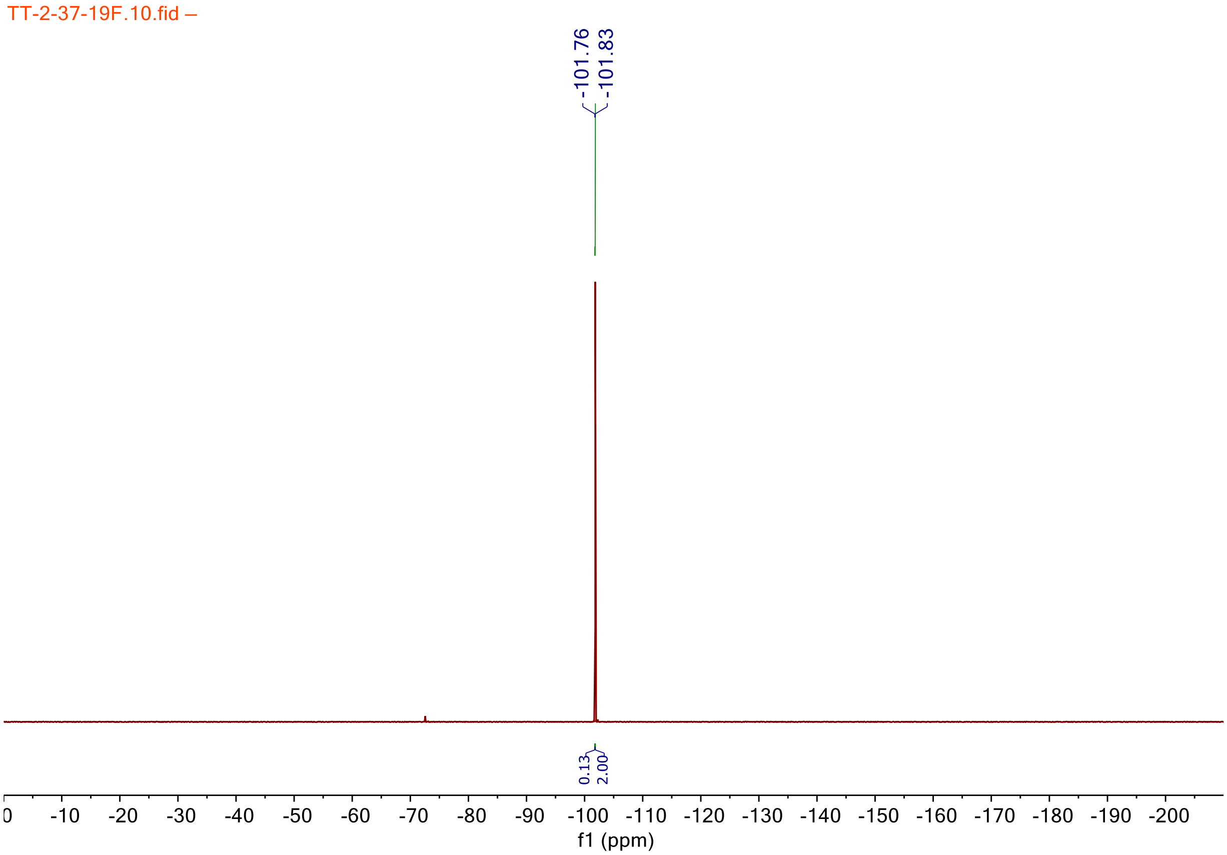

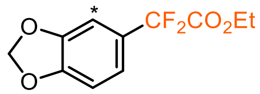

Ethyl 2-(benzo[*d*][1,3]dioxol-5-yl)-2,2-difluoroacetate (4): ^{19}F NMR (376 MHz, $CDCl_3$) for control reaction without Bu_4NI

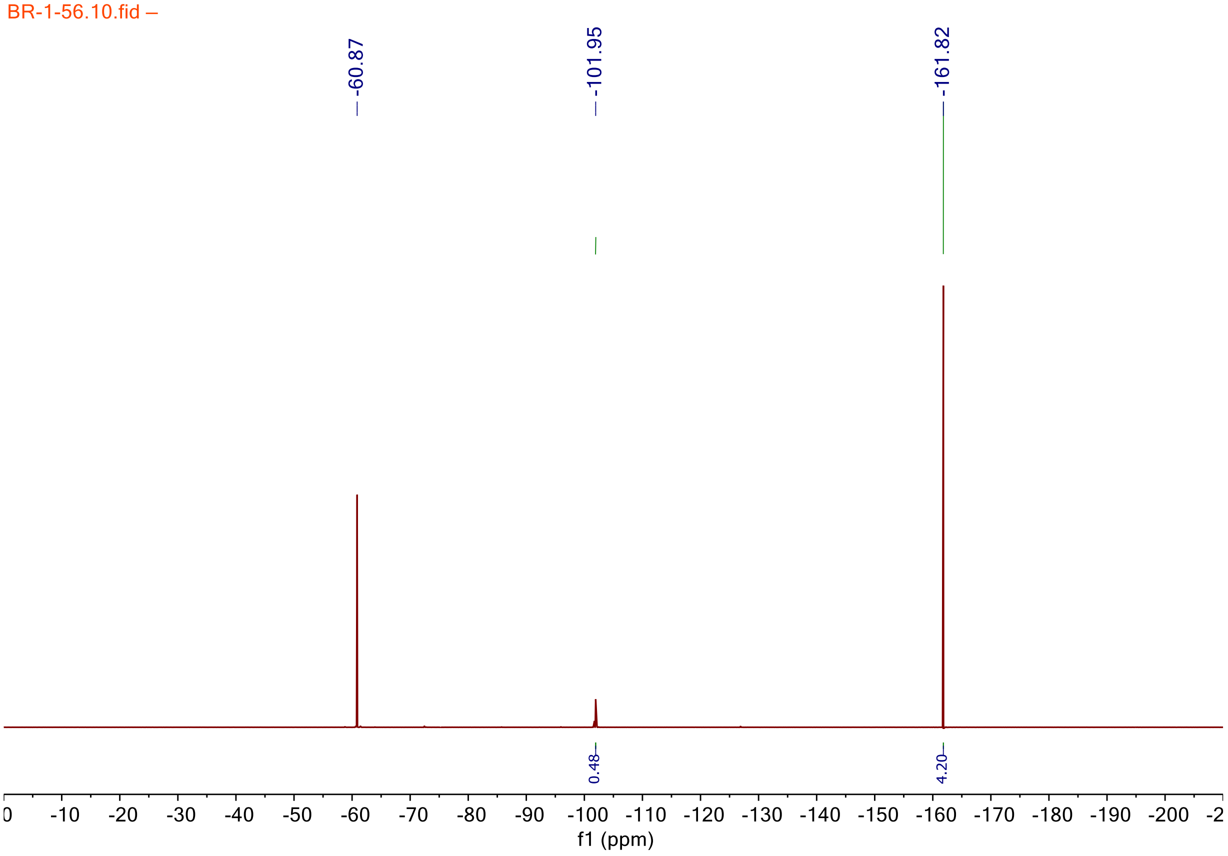

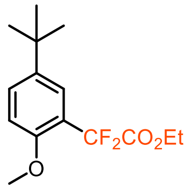

Ethyl 2-(5-(*tert*-butyl)-2-methoxyphenyl)-2,2 difluoroacetate (5): ^{1}H NMR (400 MHz, $CDCl_3$)

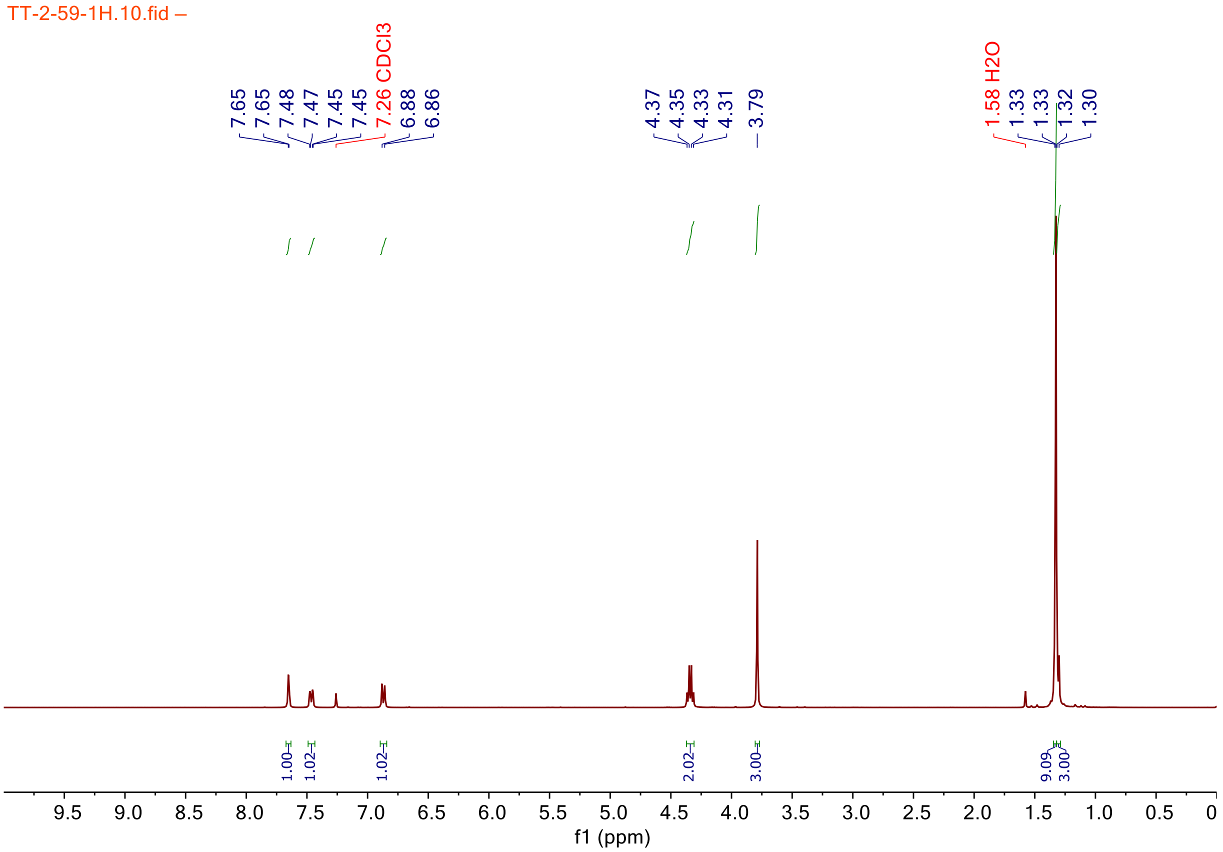

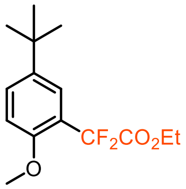

Ethyl 2-(5-(*tert*-butyl)-2-methoxyphenyl)-2,2 difluoroacetate (5): $^{13}C\{^{1}H\}$ NMR (201 MHz, $CDCl_3$)

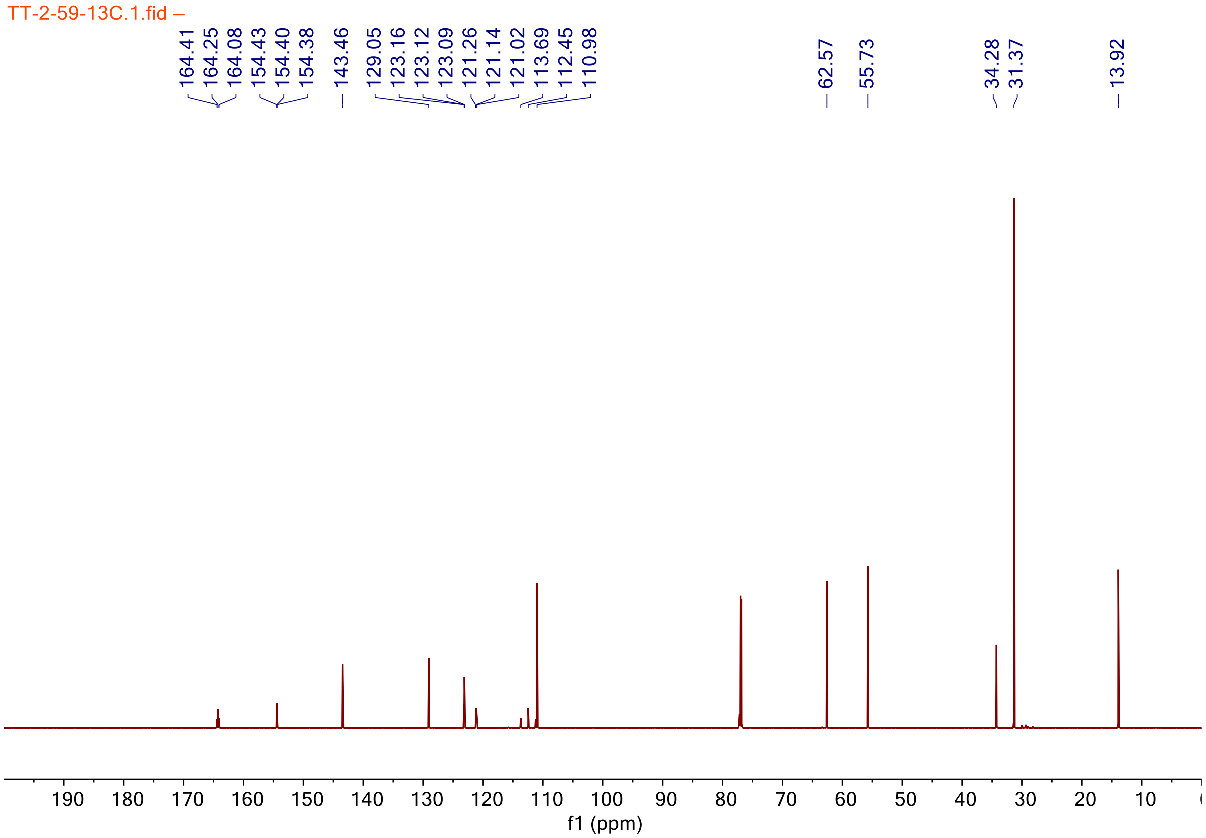

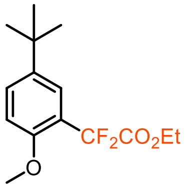

Ethyl 2-(5-(*tert*-butyl)-2-methoxyphenyl)-2,2 difluoroacetate (5):^{19}F NMR (376 MHz, $CDCl_3$)

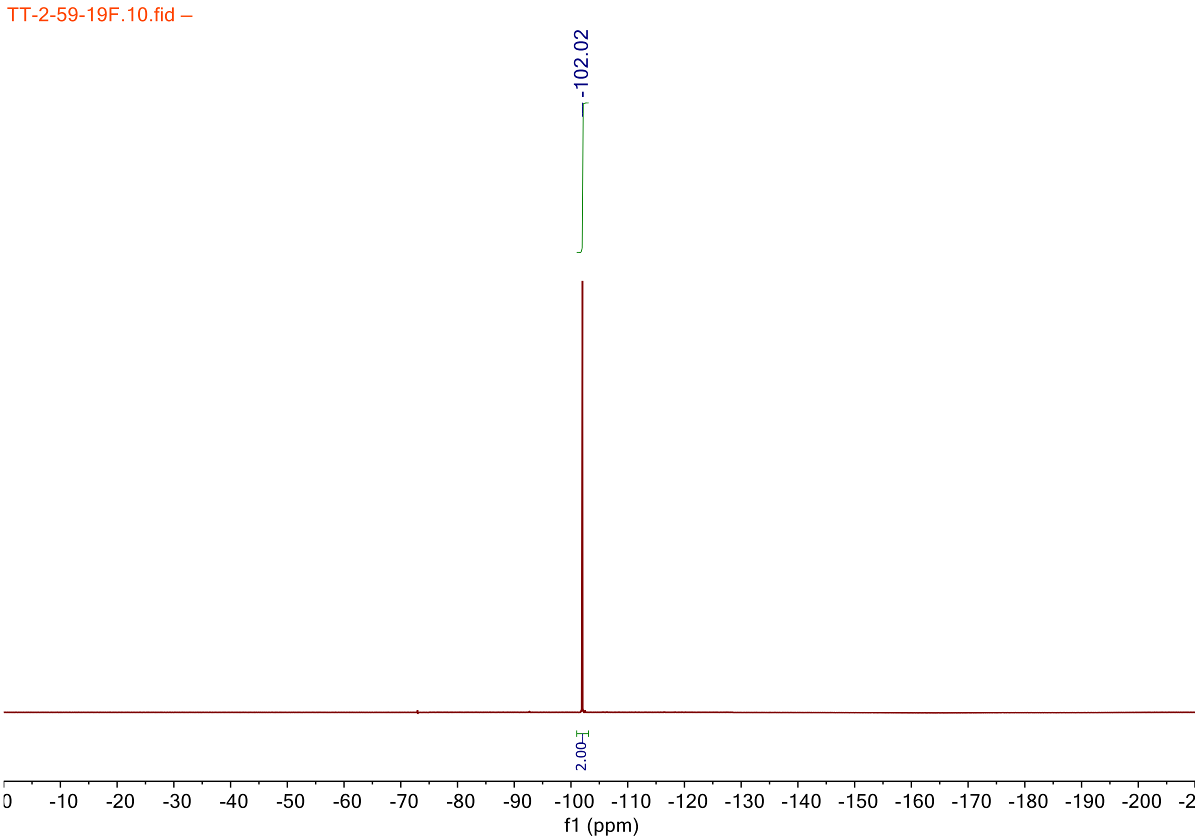

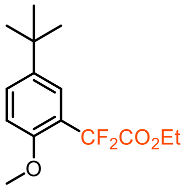

Ethyl 2-(5-(*tert*-butyl)-2-methoxyphenyl)-2,2 difluoroacetate (5): ^{19}F NMR (376 MHz, $CDCl_3$) for control reaction without Bu_4NI

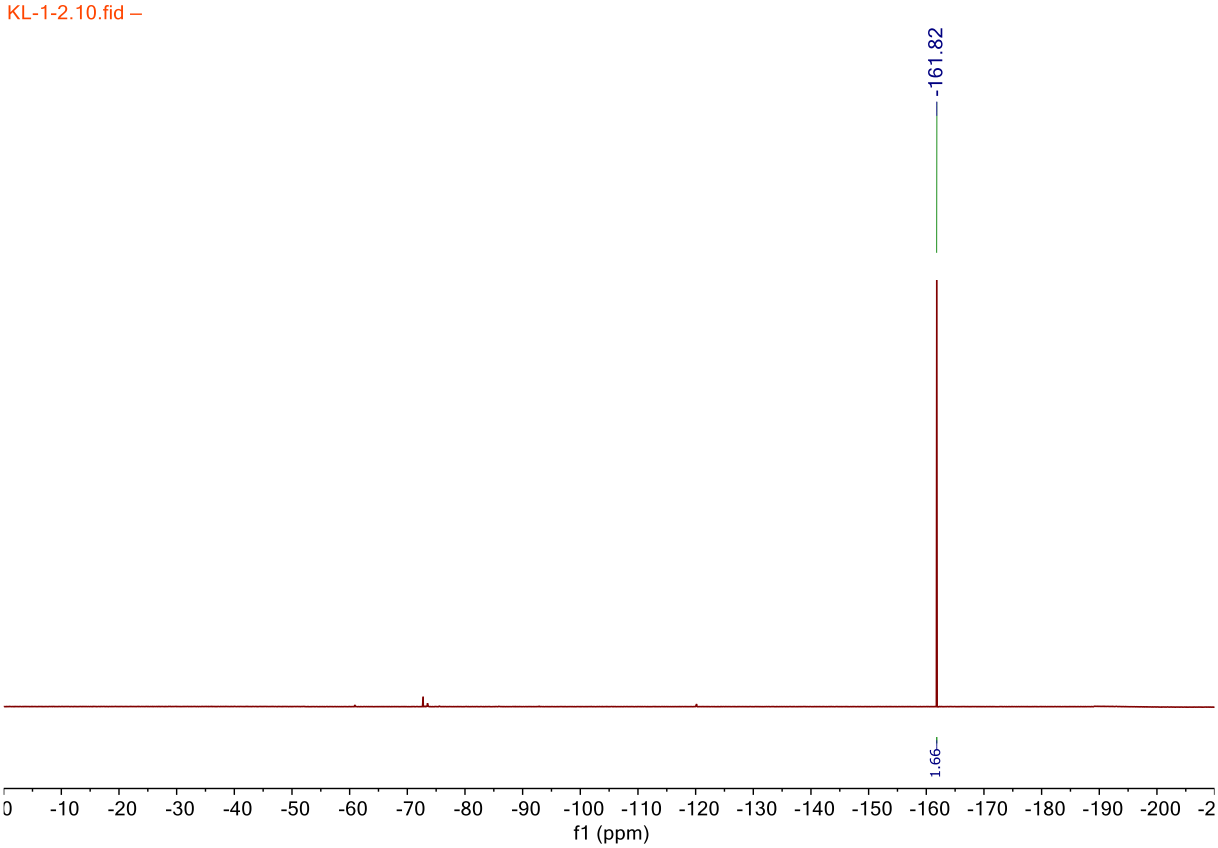

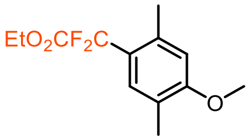

Ethyl 2,2-difluoro-2-(4-methoxy-2,5-dimethylphenyl)acetate (6): ^1H NMR (400 MHz, $CDCl_3$)

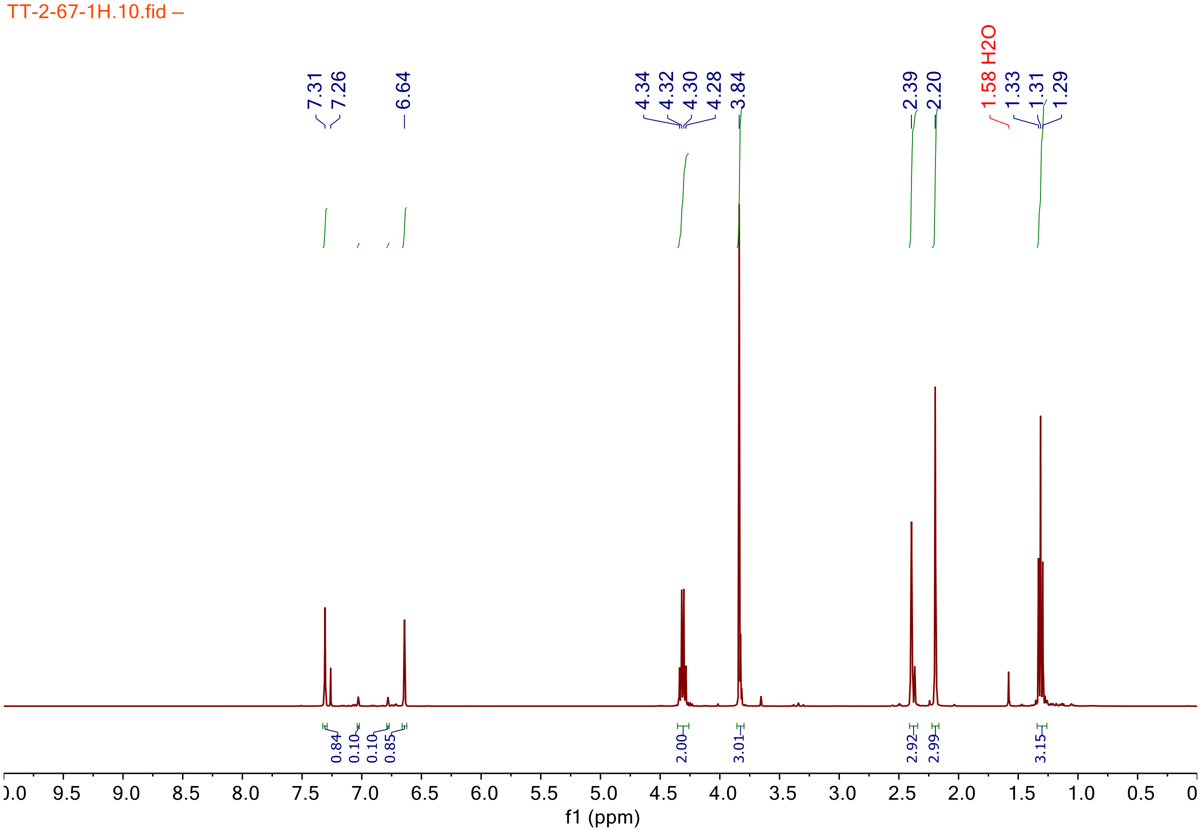

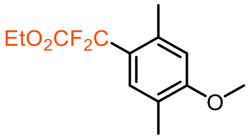

Ethyl 2,2-difluoro-2-(4-methoxy-2,5-dimethylphenyl)acetate (6): $^{13}C\{^{1}H\}$ NMR (201 MHz, $CDCl_3$)

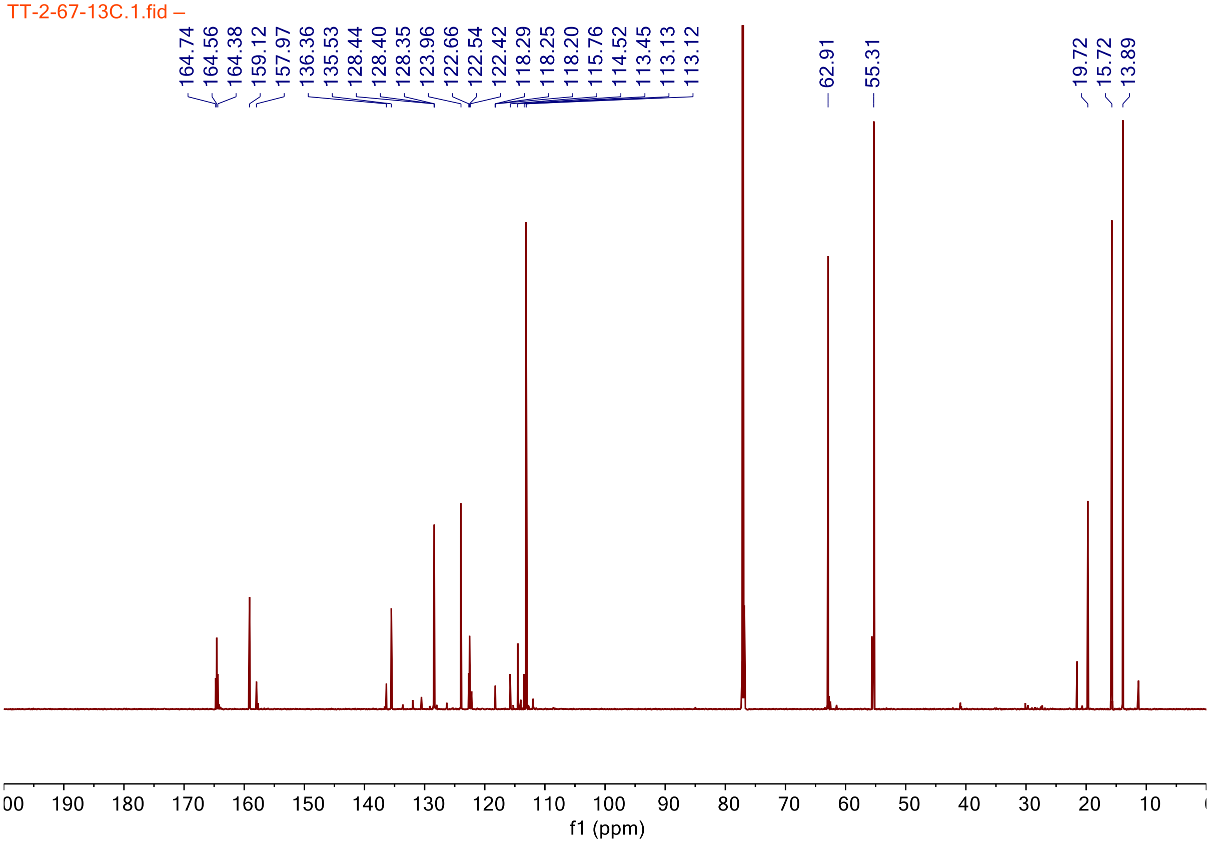

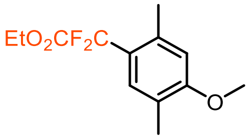

Ethyl 2,2-difluoro-2-(4-methoxy-2,5-dimethylphenyl)acetate (6): ^{19}F NMR (376 MHz, $CDCl_3$)

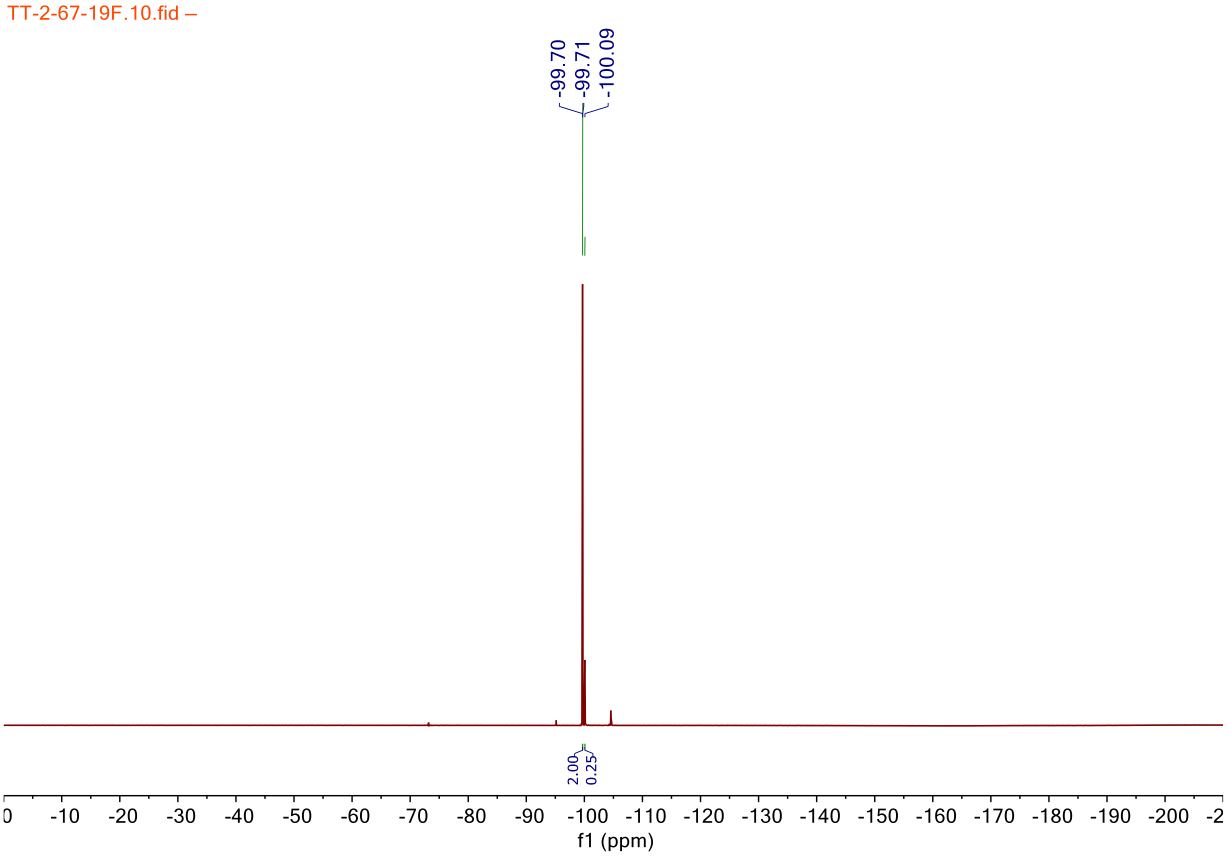

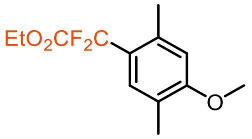

Ethyl 2,2-difluoro-2-(4-methoxy-2,5-dimethylphenyl)acetate (6): ^{19}F NMR (376 MHz, $CDCl_3$) for control reaction without Bu_4I

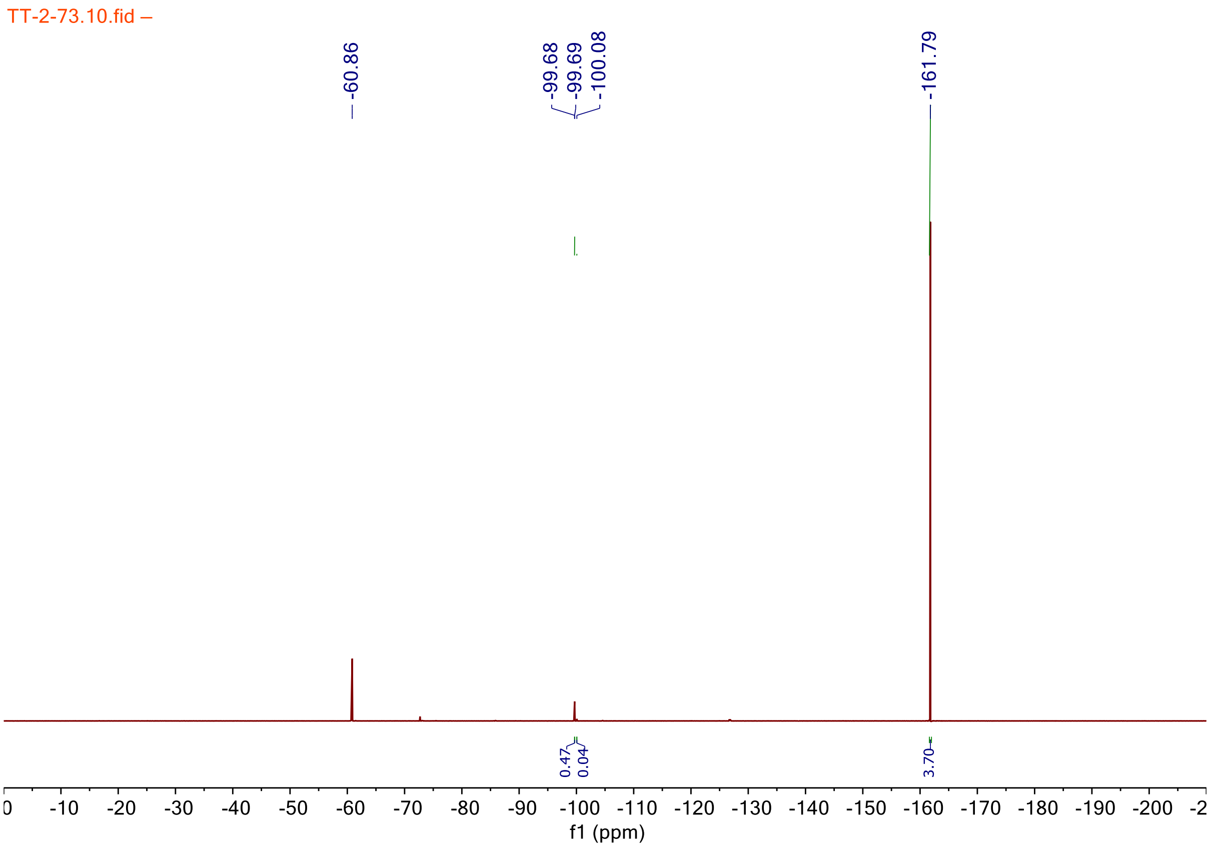

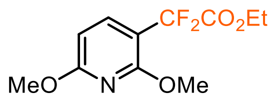

Ethyl 2-(2,6-dimethoxypyridin-3-yl)-2,2-difluoroacetate (7): ^{1}H NMR (400 MHz, $CDCl_3$)

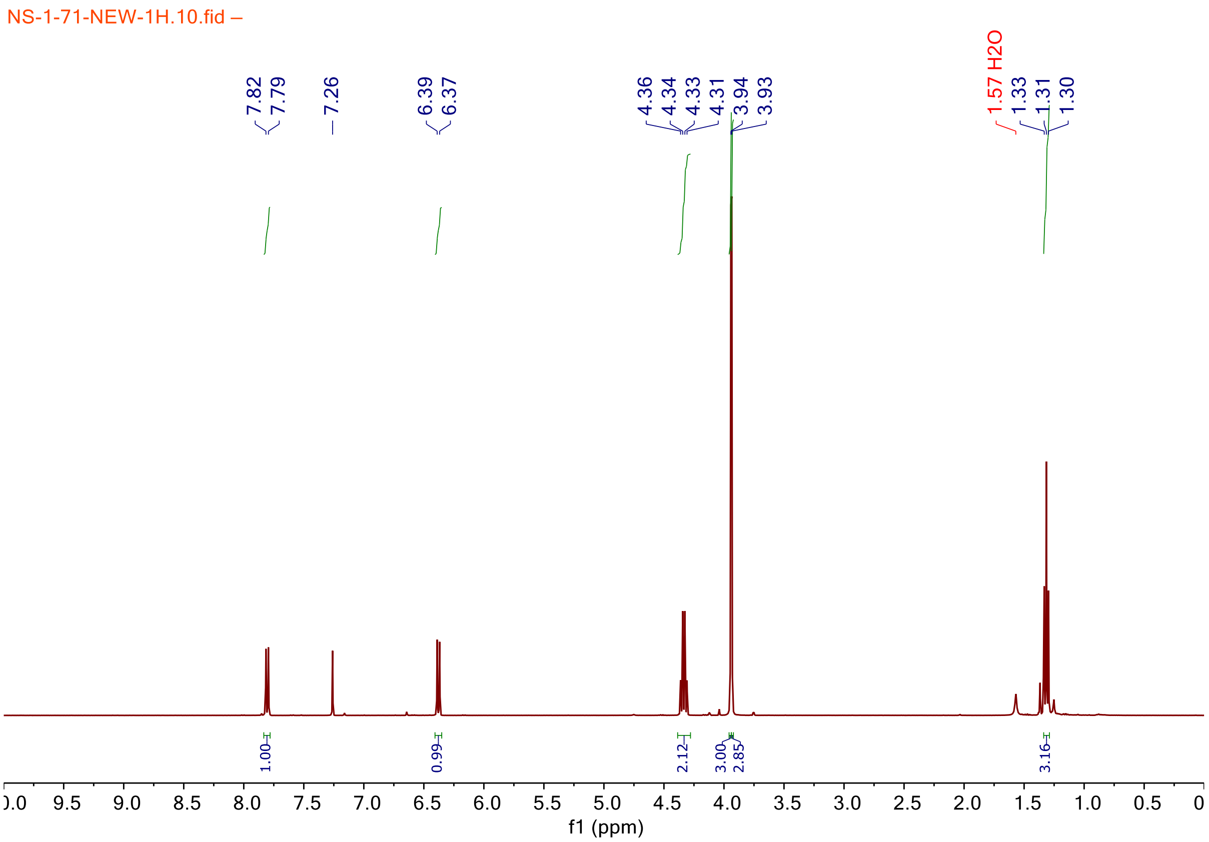

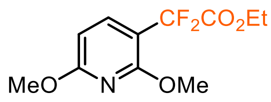

Ethyl 2-(2,6-dimethoxypyridin-3-yl)-2,2-difluoroacetate (7): $^{13}C\{^{1}H\}$ NMR (201 MHz, $CDCl_3$)

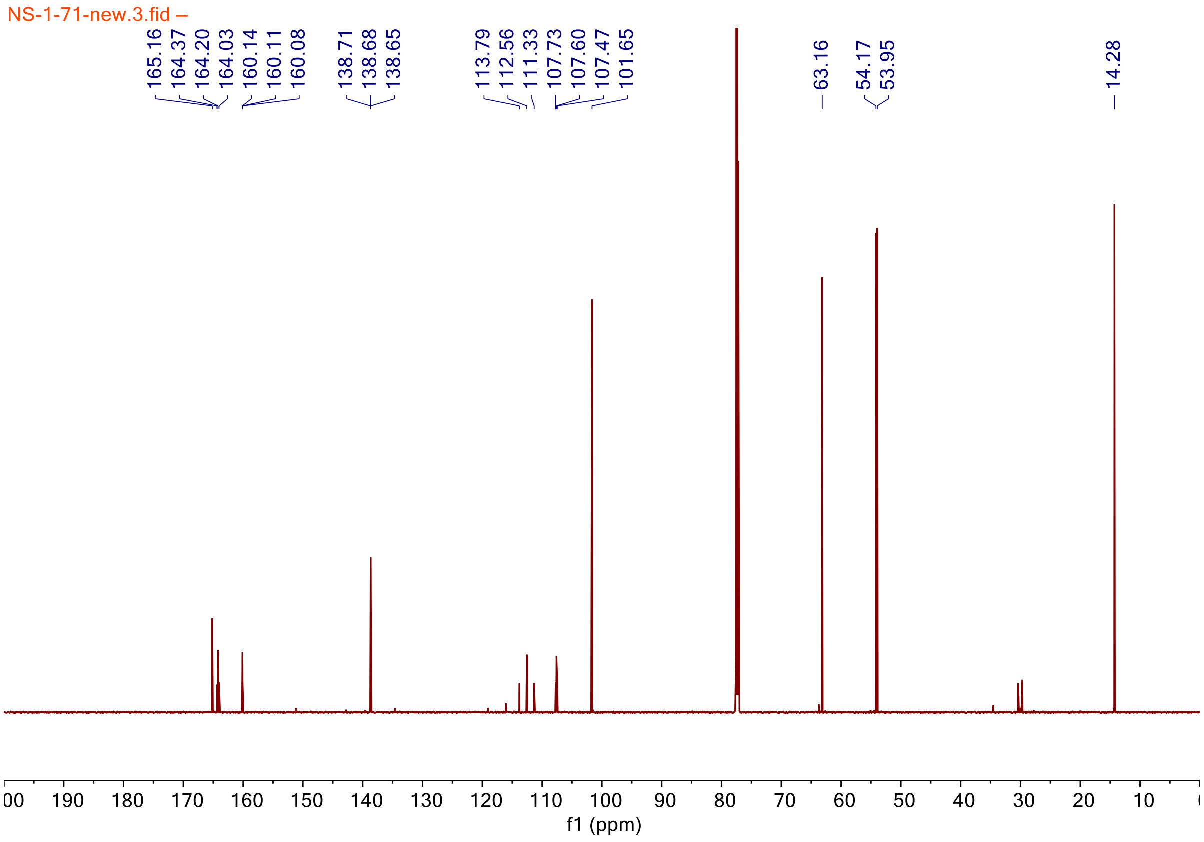

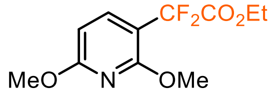

Ethyl 2-(2,6-dimethoxypyridin-3-yl)-2,2-difluoroacetate (7): ^{19}F NMR (376 MHz, $CDCl_3$)

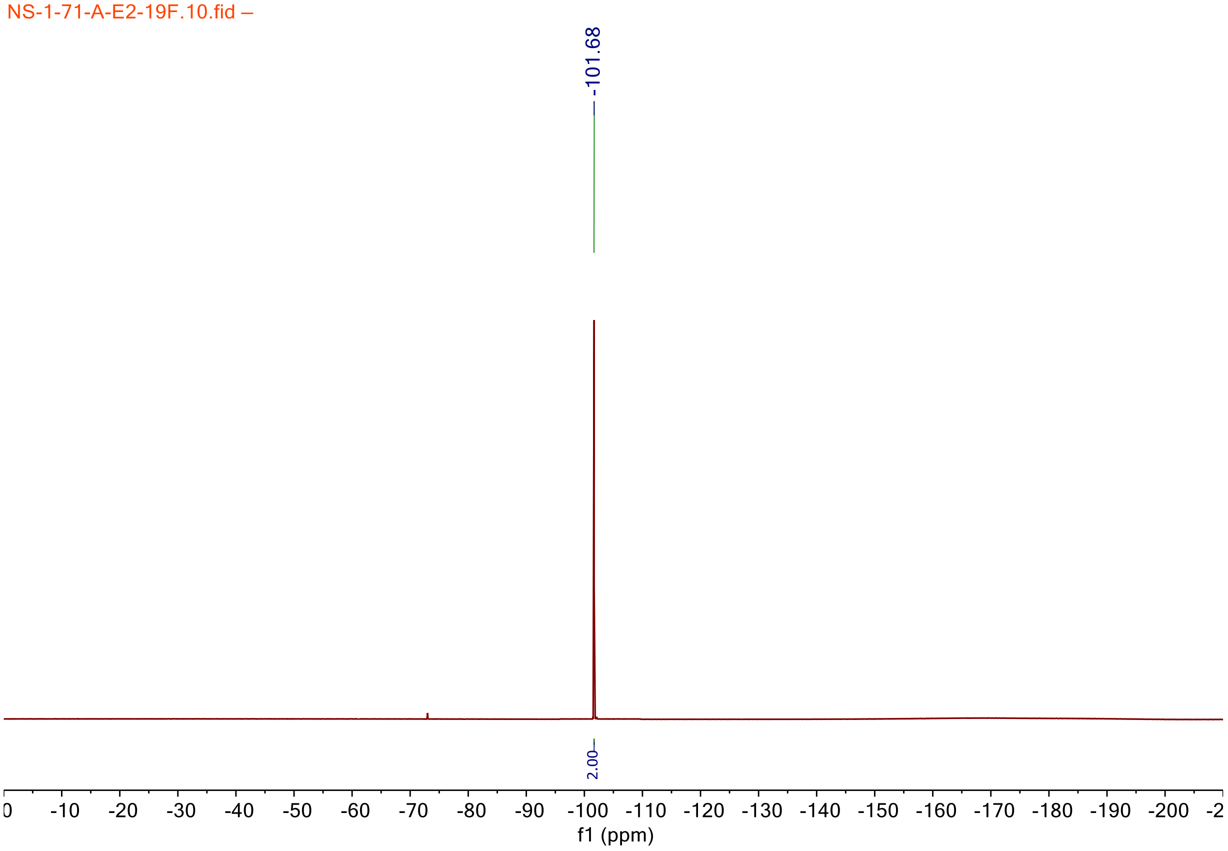

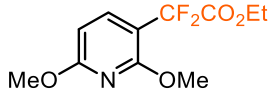

Ethyl 2-(2,6-dimethoxypyridin-3-yl)-2,2-difluoroacetate (7): ^{19}F NMR (376 MHz, $CDCl_3$) for control reaction without Bu_4NI

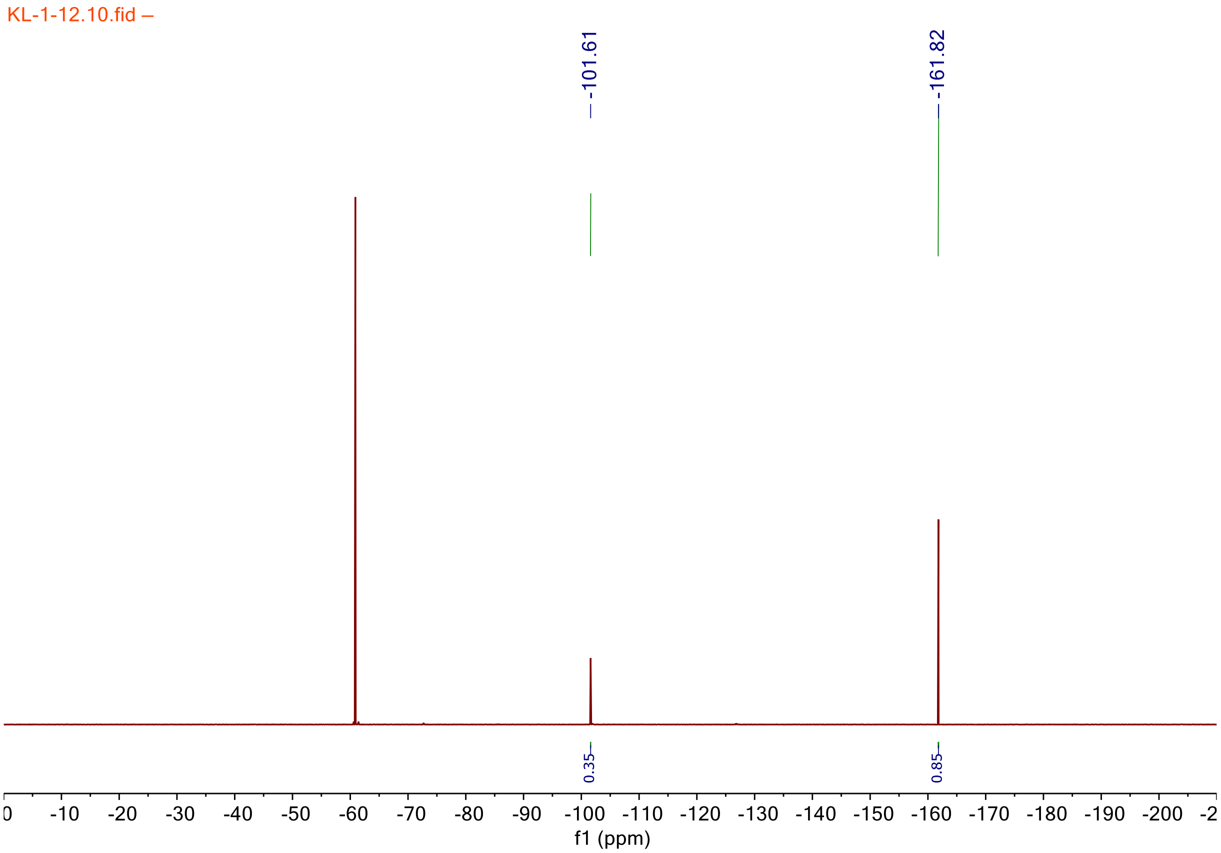

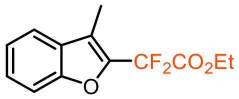

Ethyl 2,2-difluoro-2-(3-methylbenzofuran-2-yl)acetate (8): ^{1}H NMR (400 MHz, $CDCl_3$)

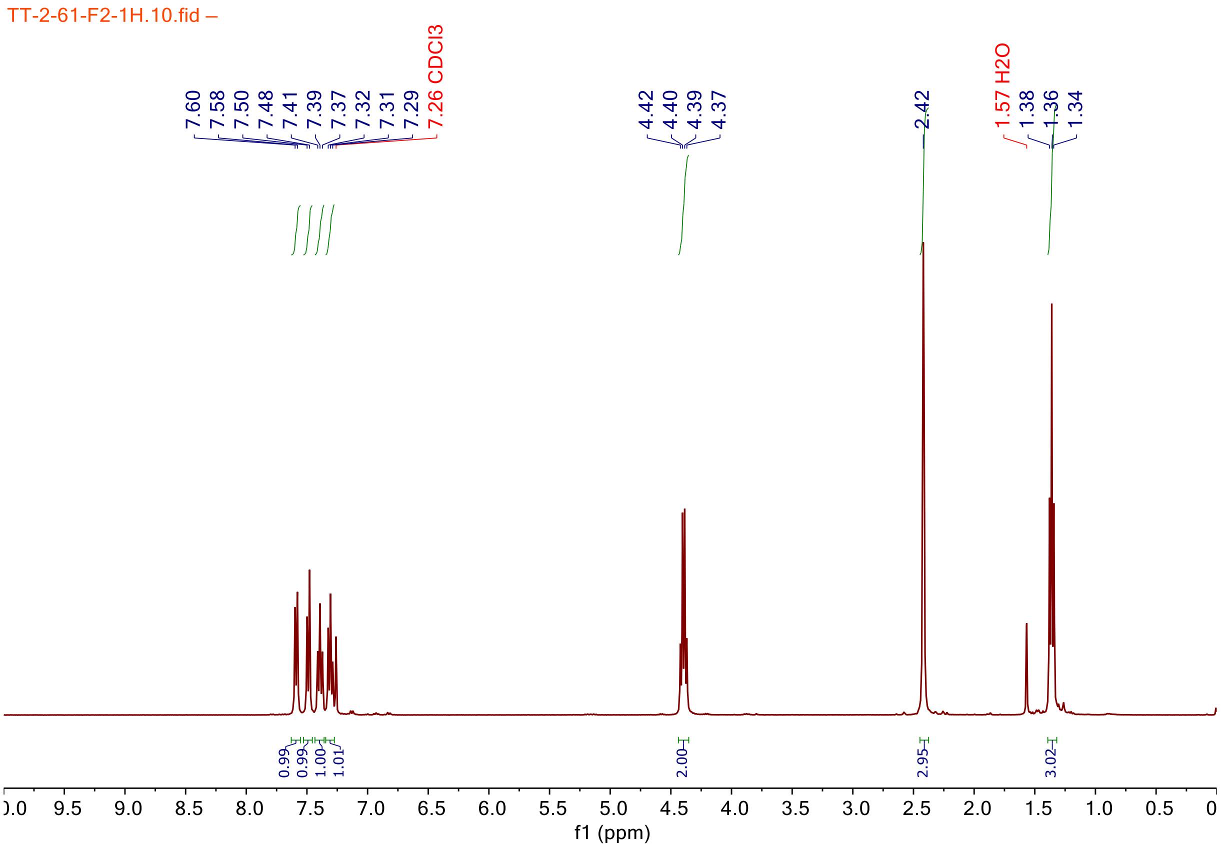

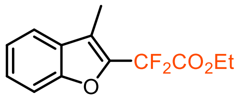

Ethyl 2,2-difluoro-2-(3-methylbenzofuran-2-yl)acetate (8): $^{13}C\{^{1}H\}$ NMR (201 MHz, $CDCl_3$)

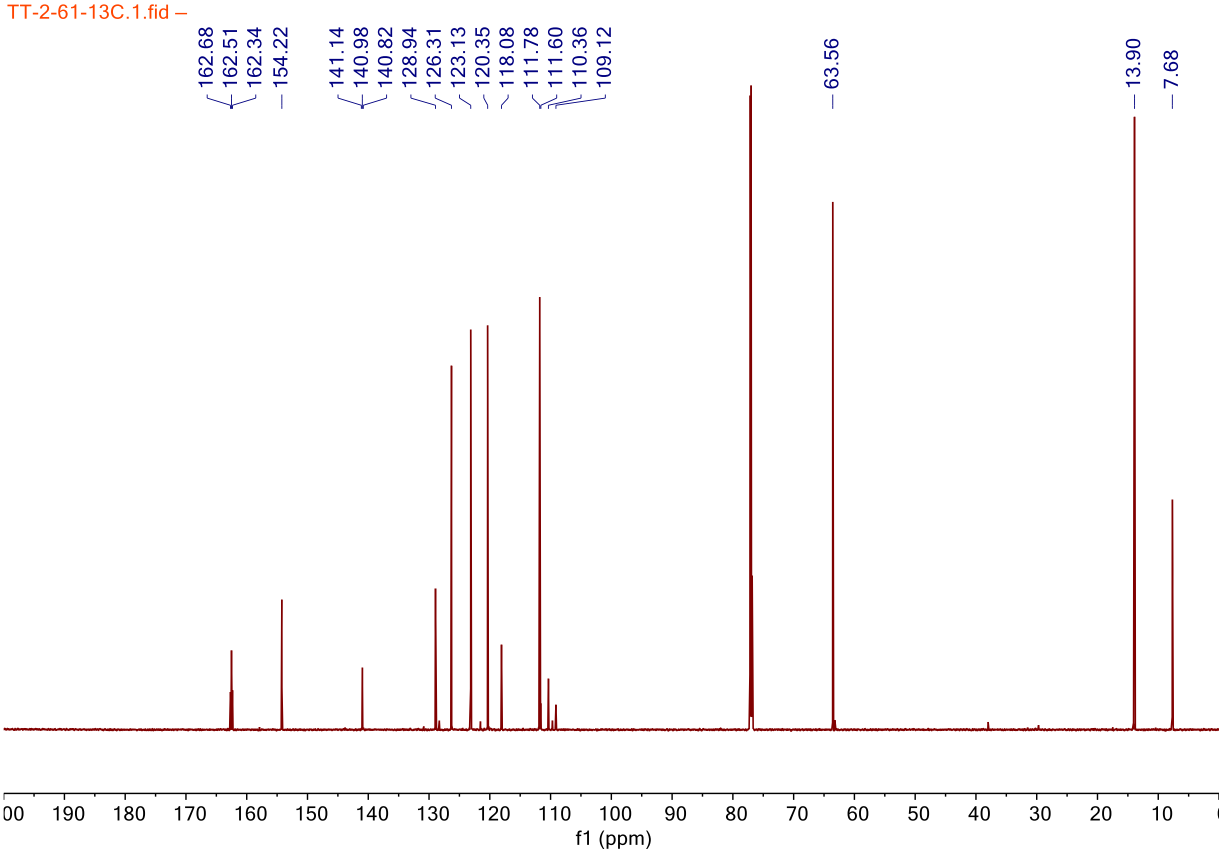

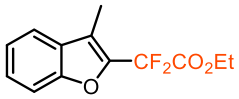

Ethyl 2,2-difluoro-2-(3-methylbenzofuran-2-yl)acetate (8): ^{19}F NMR (376 MHz, $CDCl_3$)

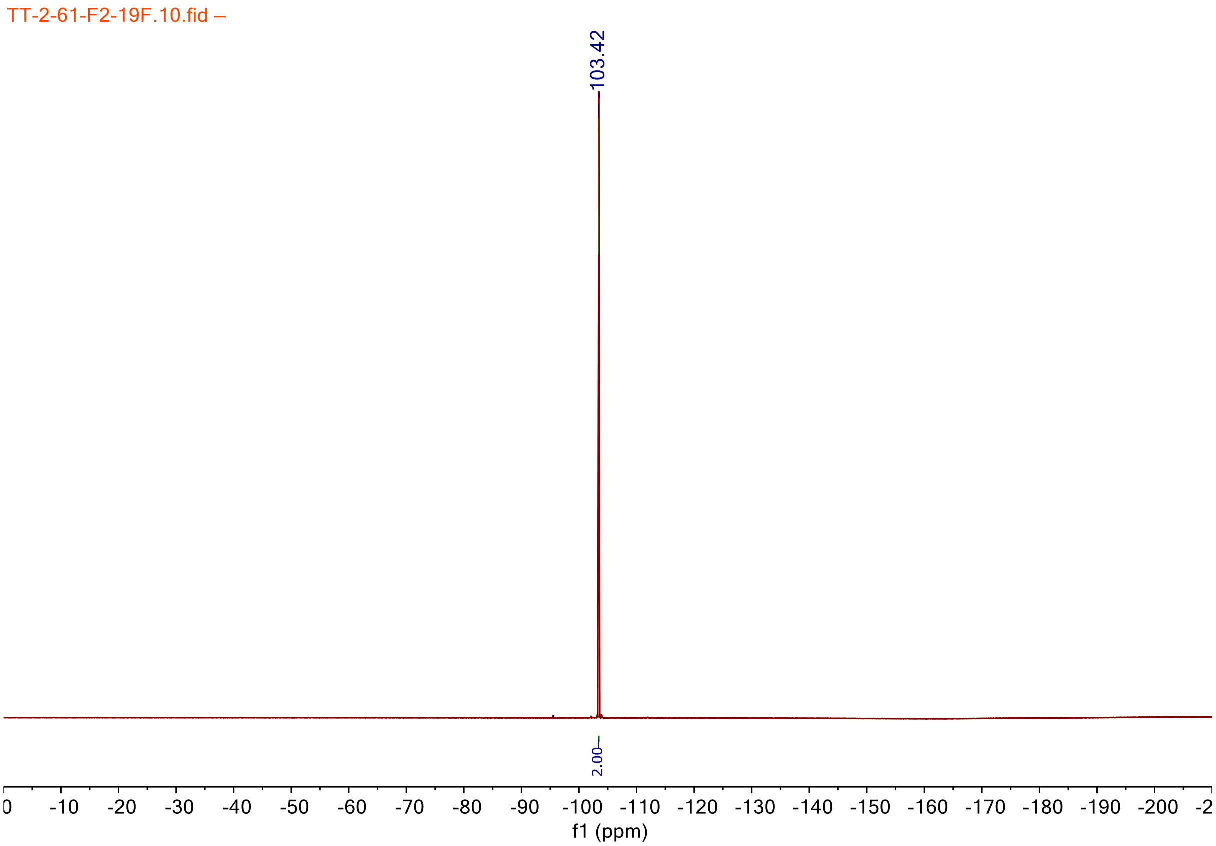

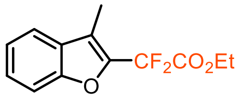

Ethyl 2,2-difluoro-2-(3-methylbenzofuran-2-yl)acetate (8): ^{19}F NMR (376 MHz, $CDCl_3$) for control reaction without Bu_4NI

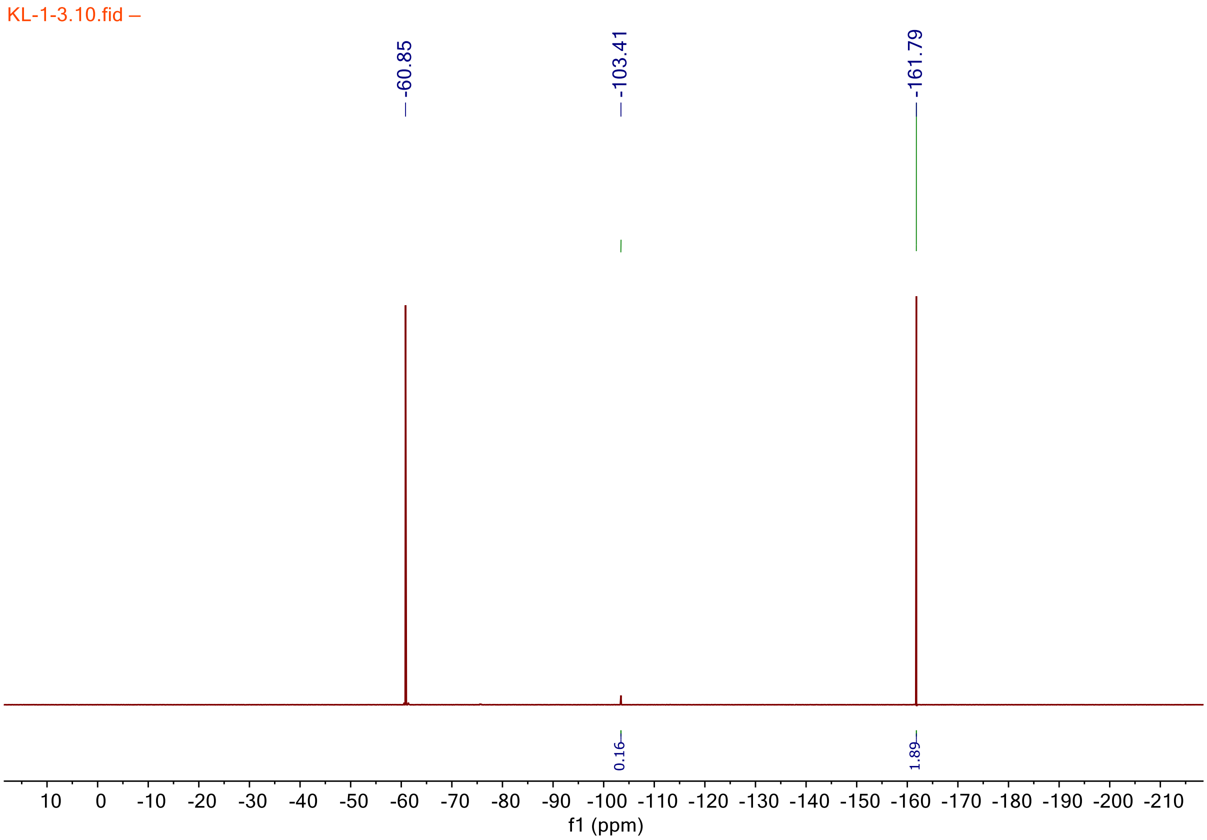

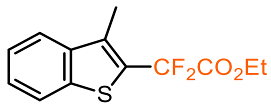

Ethyl 2,2-difluoro-2-(3-methylbenzo[*b*]thiophen-2-yl)acetate (9): ^{1}H NMR (400 MHz, $CDCl_3$)

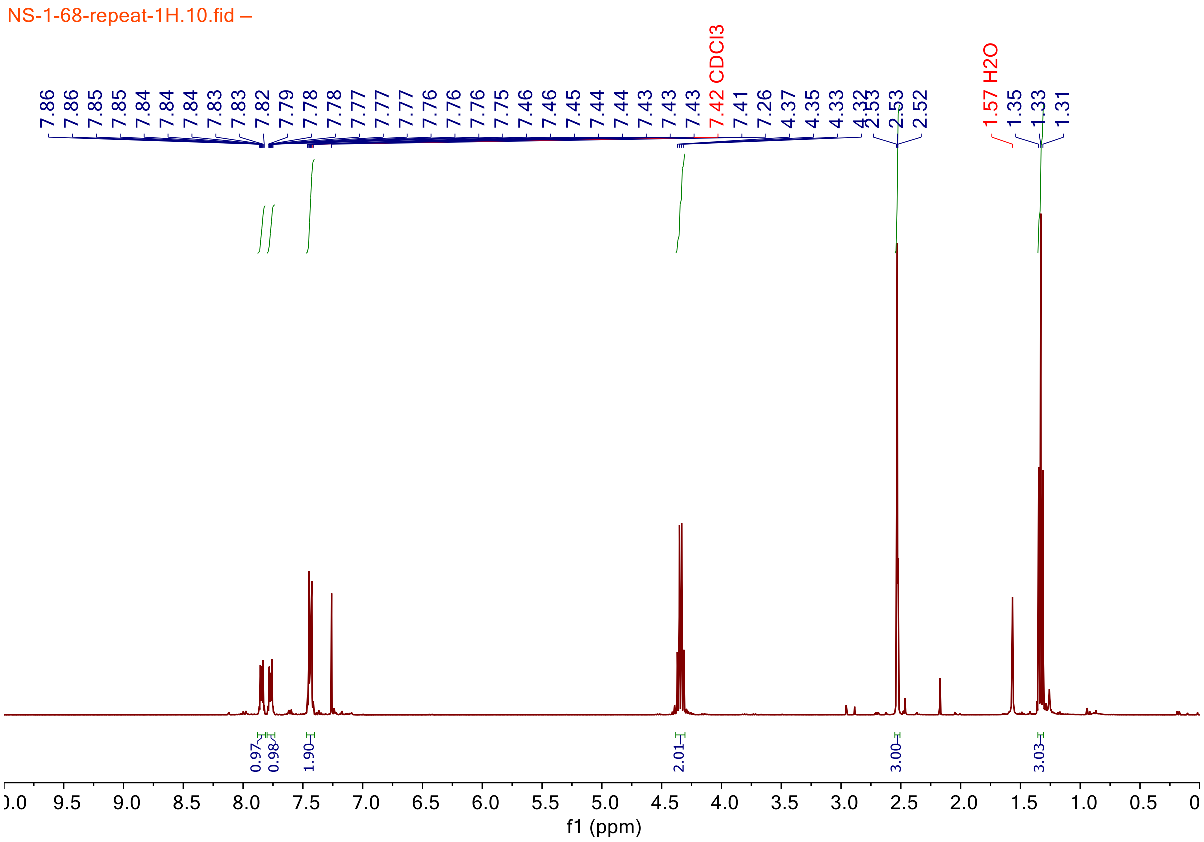

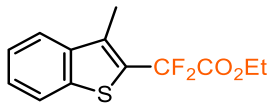

Ethyl 2,2-difluoro-2-(3-methylbenzo[*b*]thiophen-2-yl)acetate (9): $^{13}C\{^{1}H\}$ NMR (201 MHz, $CDCl_3$)

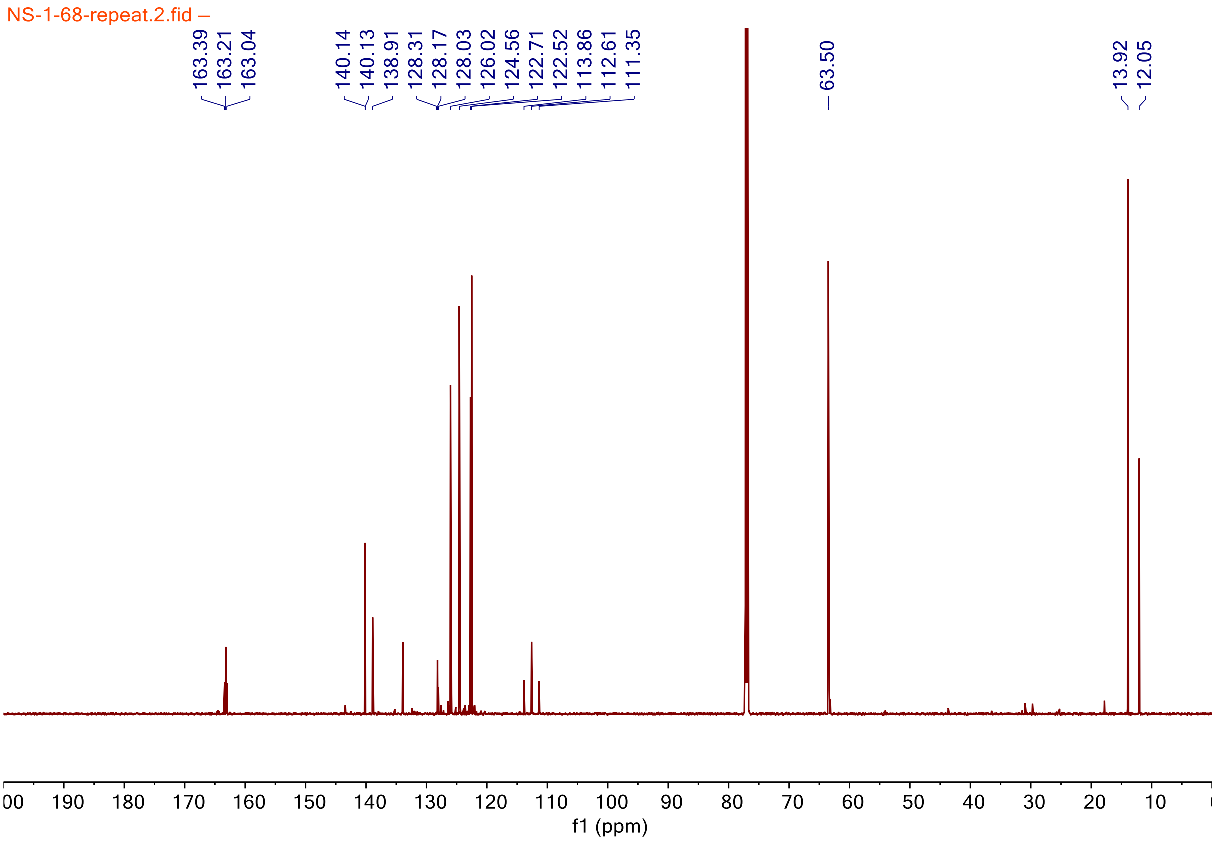

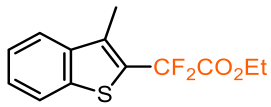

Ethyl 2,2-difluoro-2-(3-methylbenzo[*b*]thiophen-2-yl)acetate (9): ^{19}F NMR (376 MHz, $CDCl_3$)

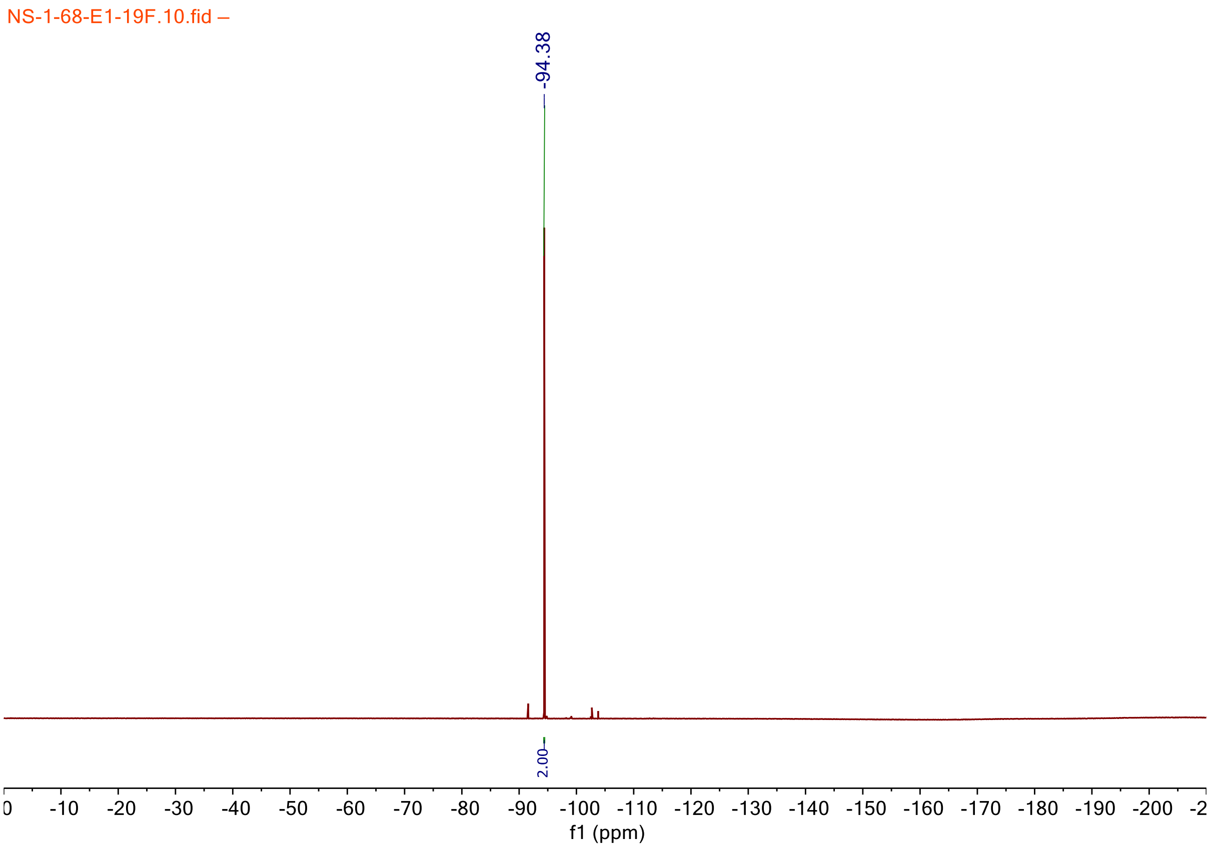

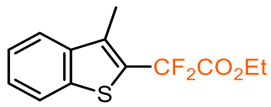

Ethyl 2,2-difluoro-2-(3-methylbenzo[*b*]thiophen-2-yl)acetate (9): ^{19}F NMR (376 MHz, $CDCl_3$) for control reaction without Bu_4NI

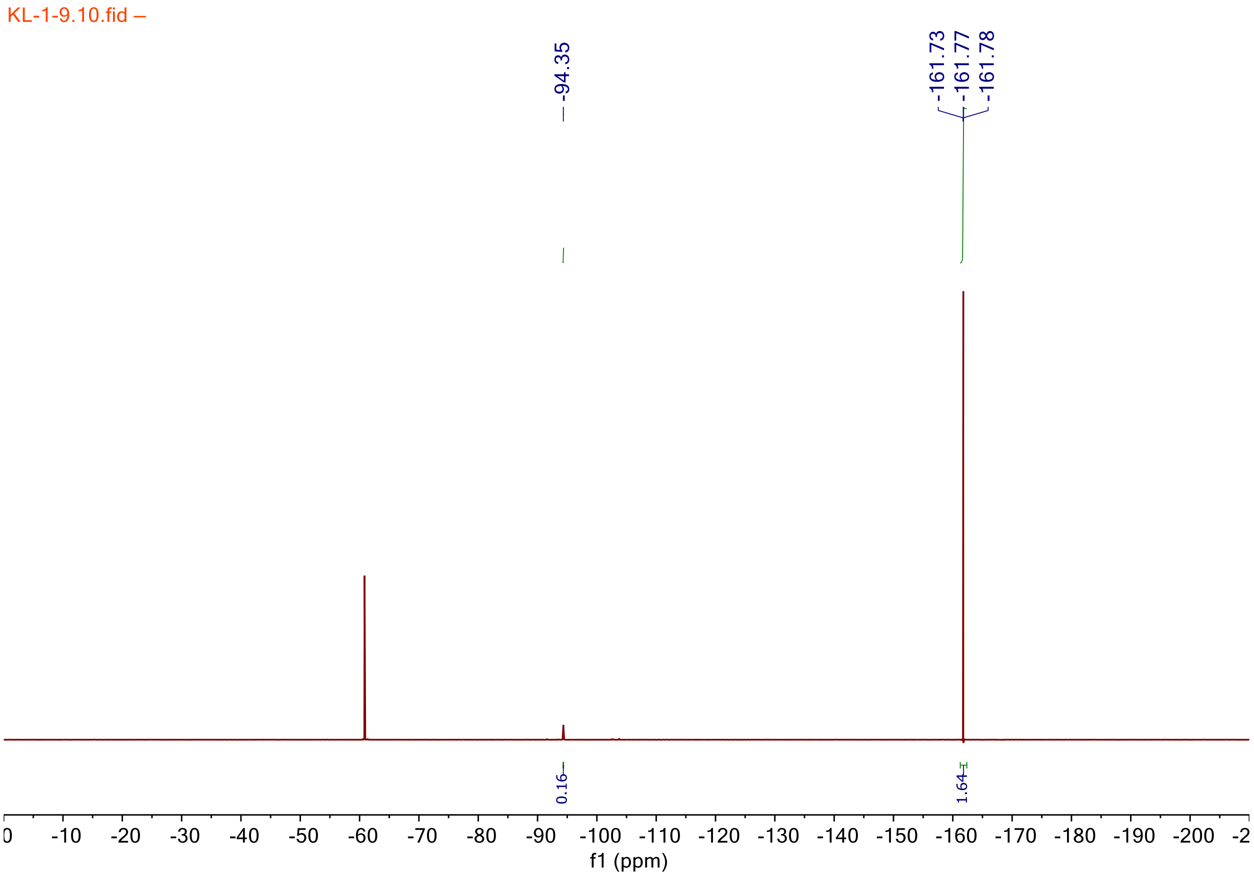

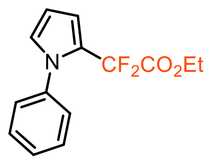

Ethyl 2,2-difluoro-2-(1-phenyl-1*H*-pyrrol-2-yl)acetate (10): ^{1}H NMR (400 MHz, $CDCl_3$)

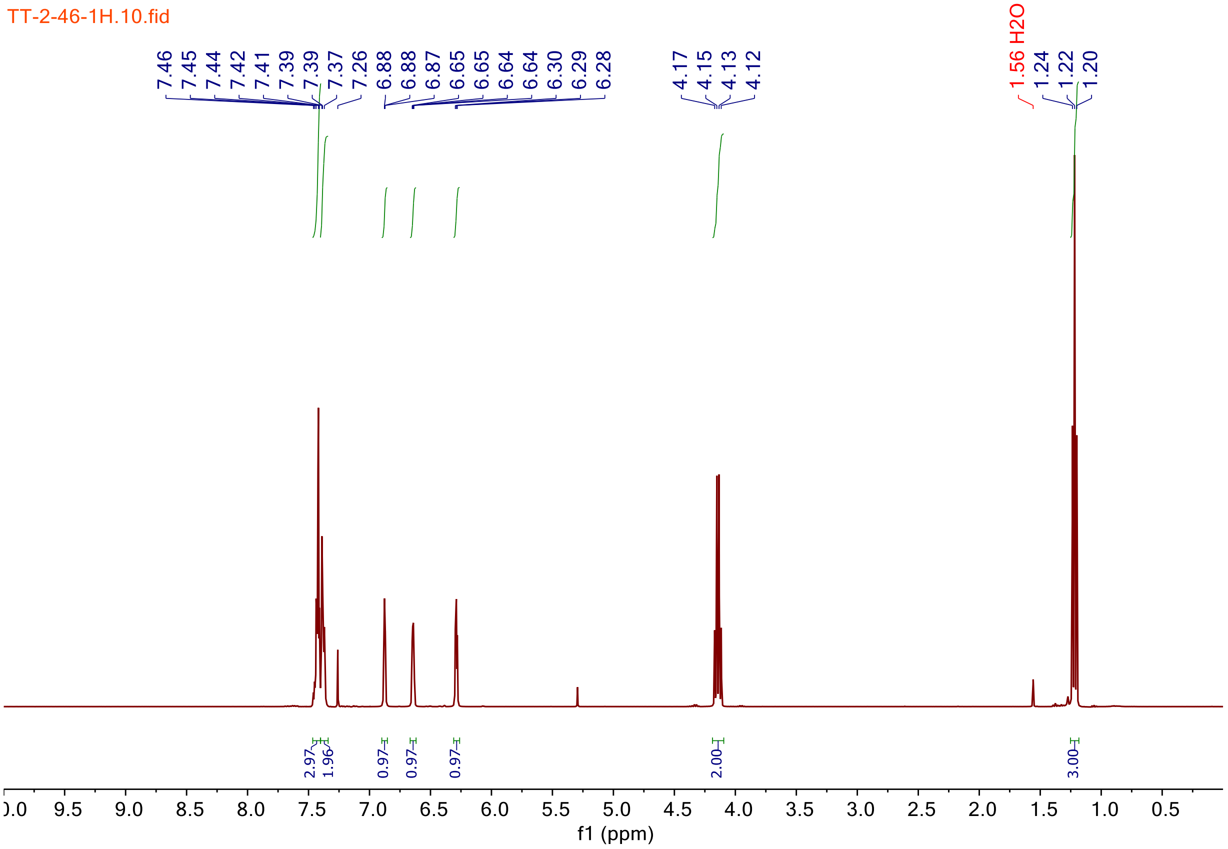

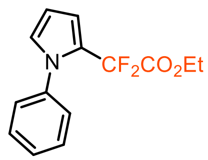

Ethyl 2,2-difluoro-2-(1-phenyl-1*H*-pyrrol-2-yl)acetate (10): $^{13}C\{^{1}H\}$ NMR (201 MHz, $CDCl_3$)

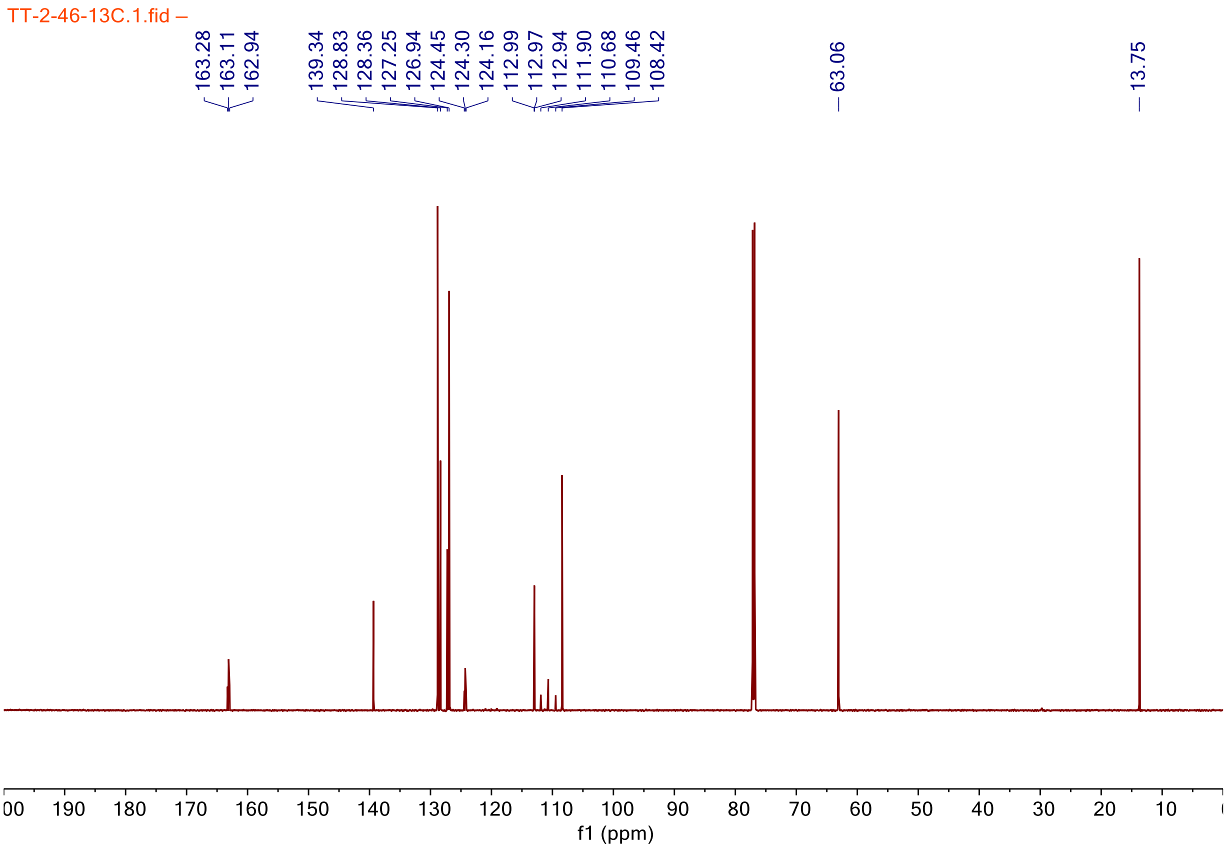

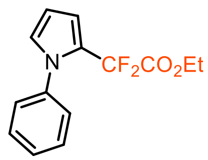

Ethyl 2,2-difluoro-2-(1-phenyl-1*H*-pyrrol-2-yl)acetate (10): ^{19}F NMR (376 MHz, $CDCl_3$)

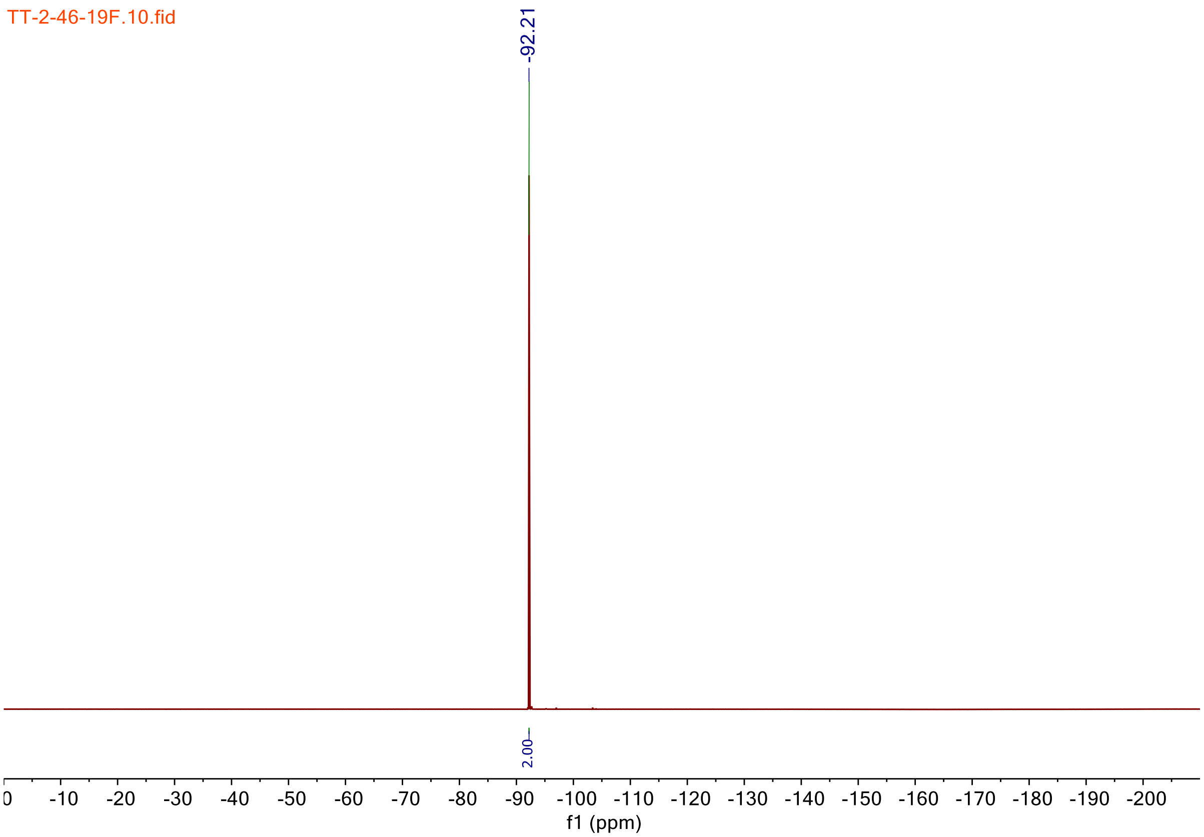

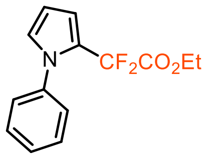

Ethyl 2,2-difluoro-2-(1-phenyl-1*H*-pyrrol-2-yl)acetate (10): ^{19}F NMR (376 MHz, $CDCl_3$) for control reaction without Bu_4NI

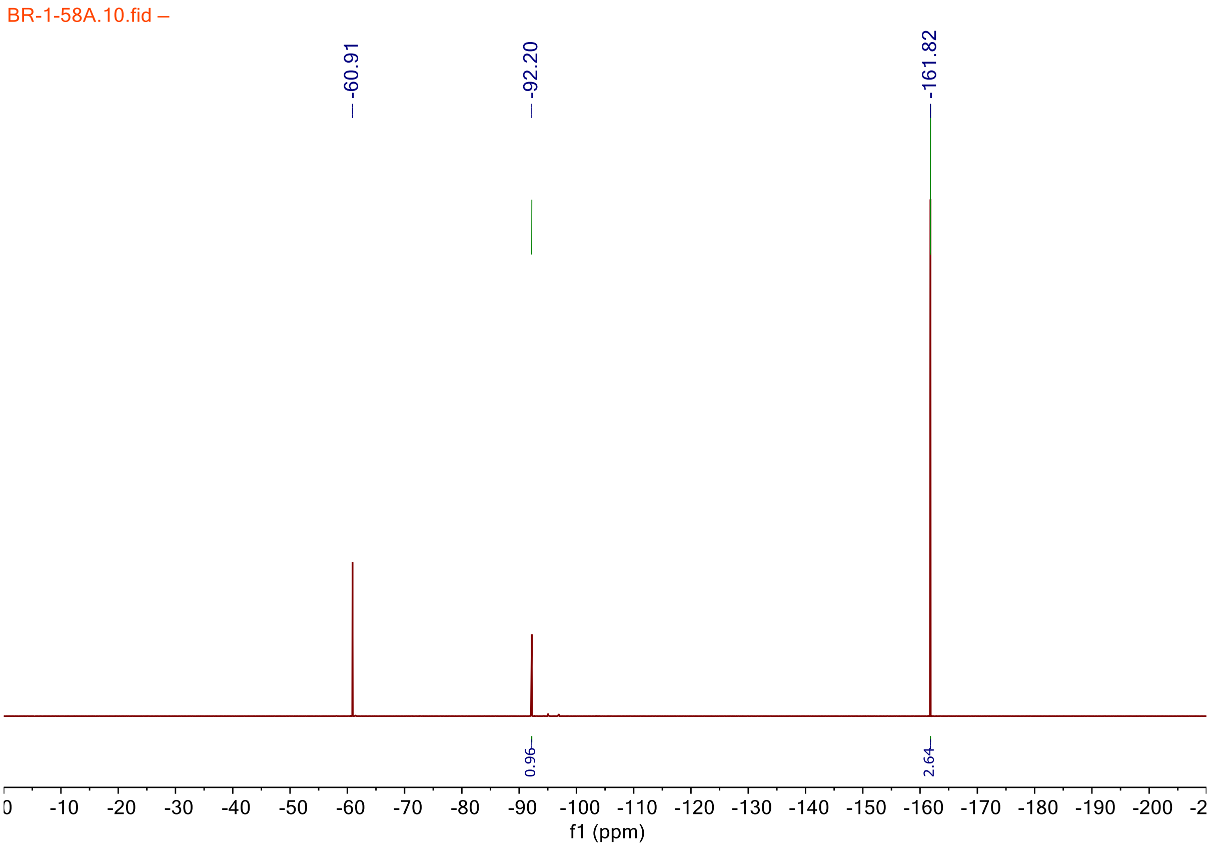

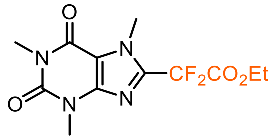

Ethyl 2,2-difluoro-2-(1,3,7-trimethyl-2,6-dioxo-2,3,6,7-tetrahydro-1*H*-purin-8-yl)acetate (11): ^{1}H NMR (400 MHz, $CDCl_3$)

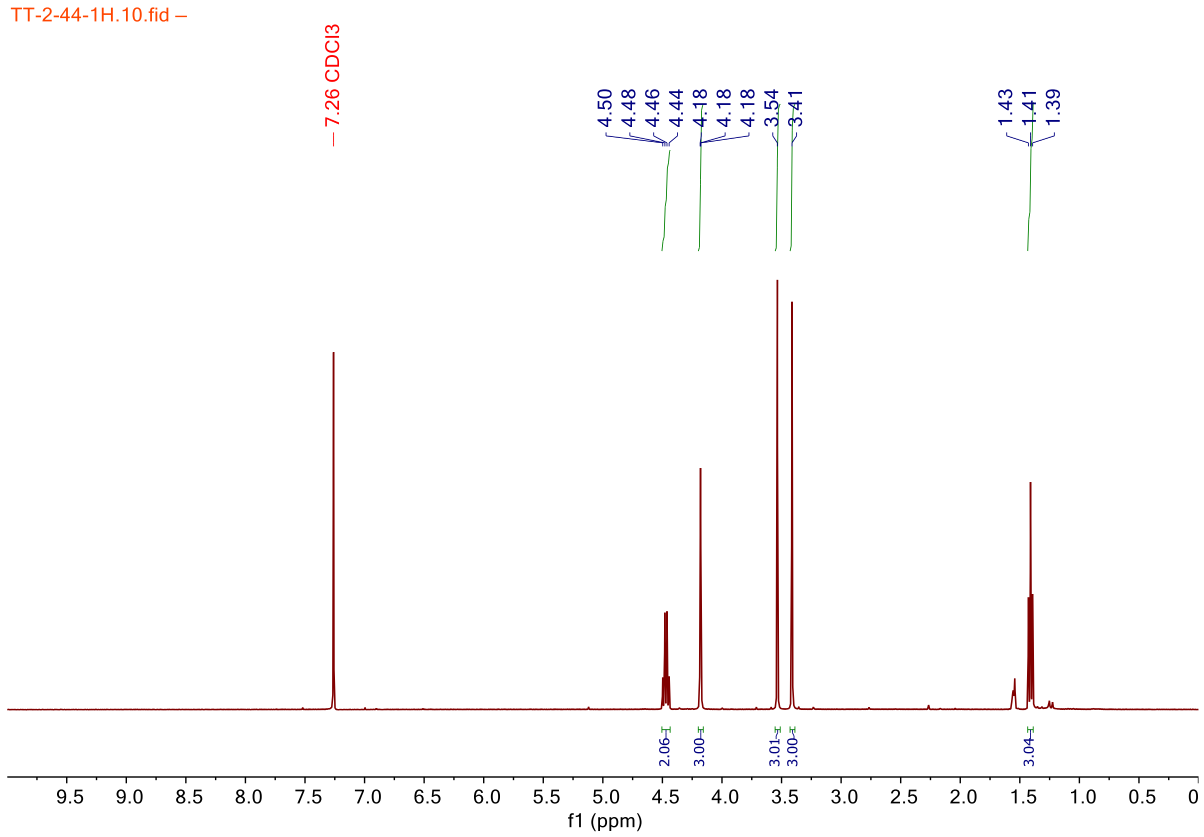

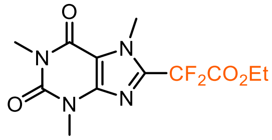

Ethyl 2,2-difluoro-2-(1,3,7-trimethyl-2,6-dioxo-2,3,6,7-tetrahydro-1*H*-purin-8-yl)acetate (11): $^{13}C\{^1H\}$ NMR (201 MHz, $CDCl_3$)

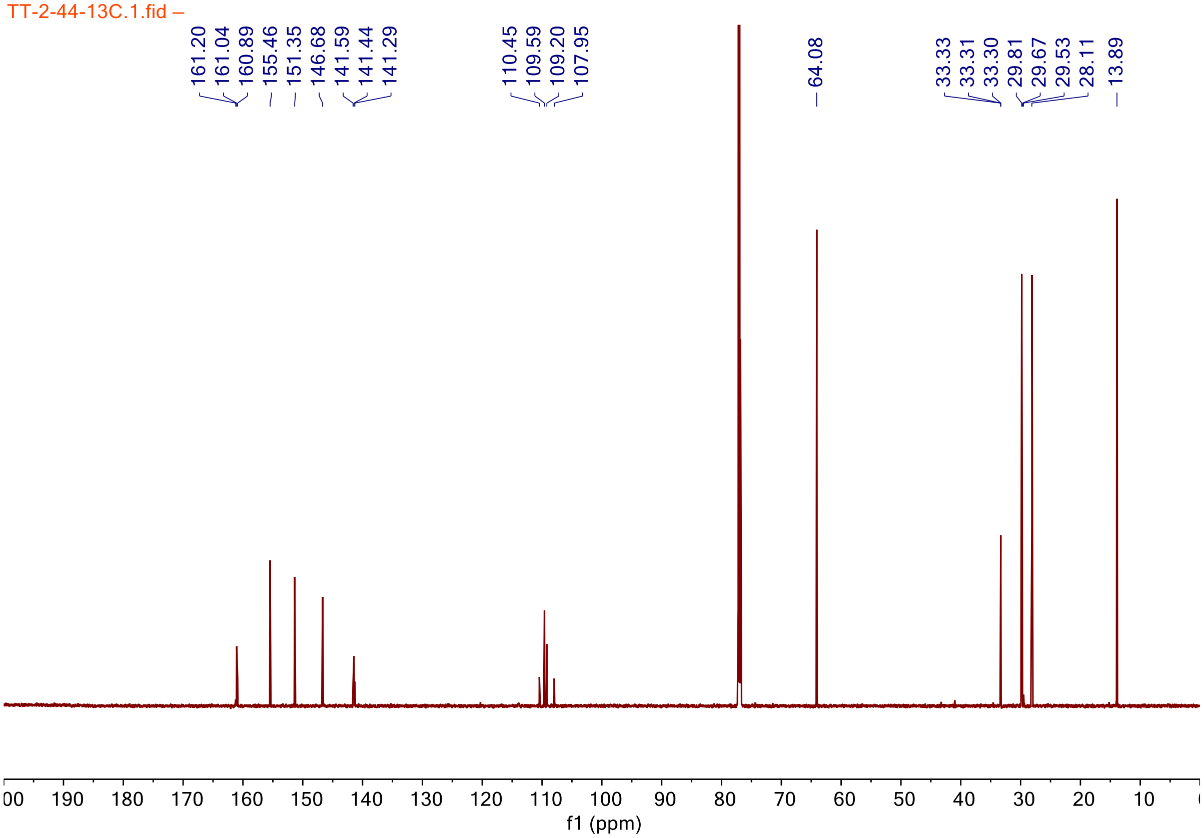

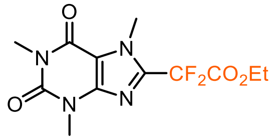

Ethyl 2,2-difluoro-2-(1,3,7-trimethyl-2,6-dioxo-2,3,6,7-tetrahydro-1*H*-purin-8-yl)acetate (11): ^{19}F NMR (376 MHz, $CDCl_3$)

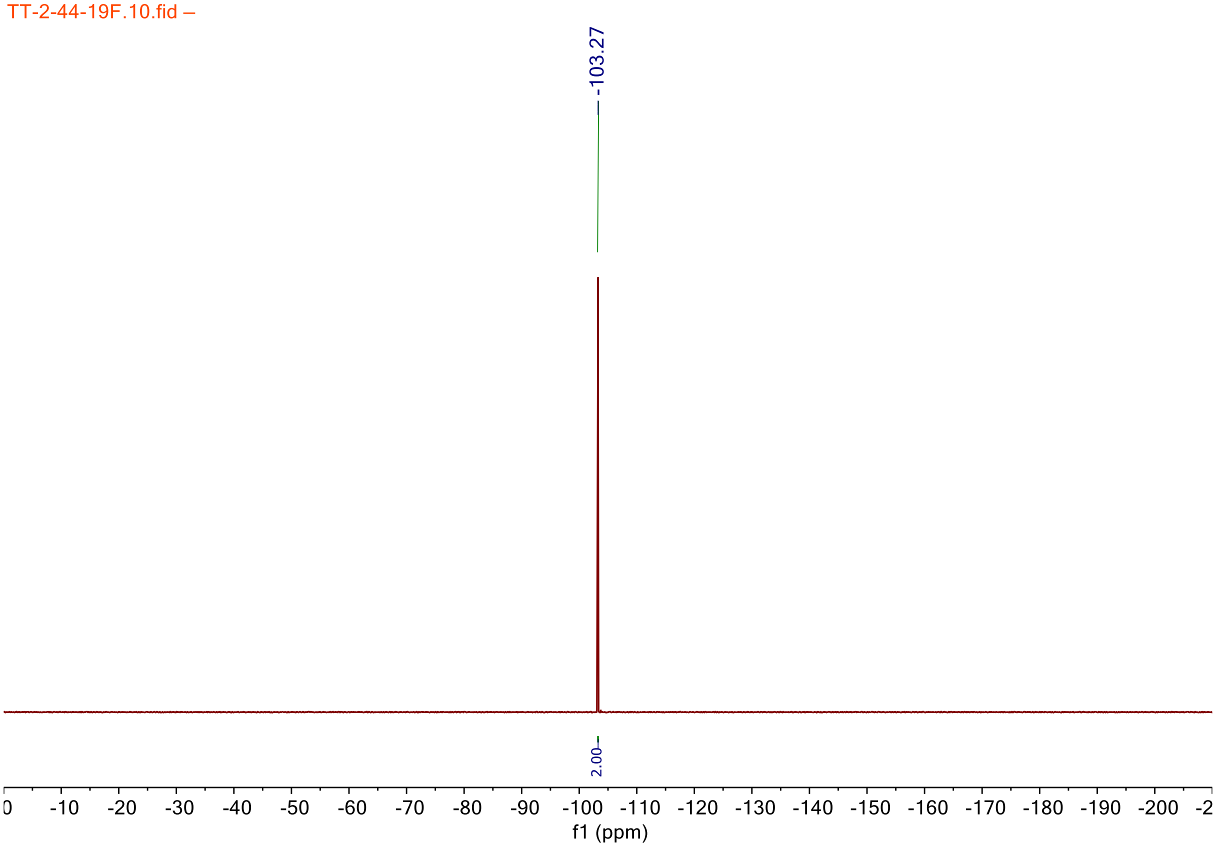

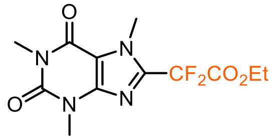

Ethyl 2,2-difluoro-2-(1,3,7-trimethyl-2,6-dioxo-2,3,6,7-tetrahydro-1*H*-purin-8-yl)acetate (11): ^{19}F NMR (376 MHz, $CDCl_3$) for control reaction without Bu_4NI

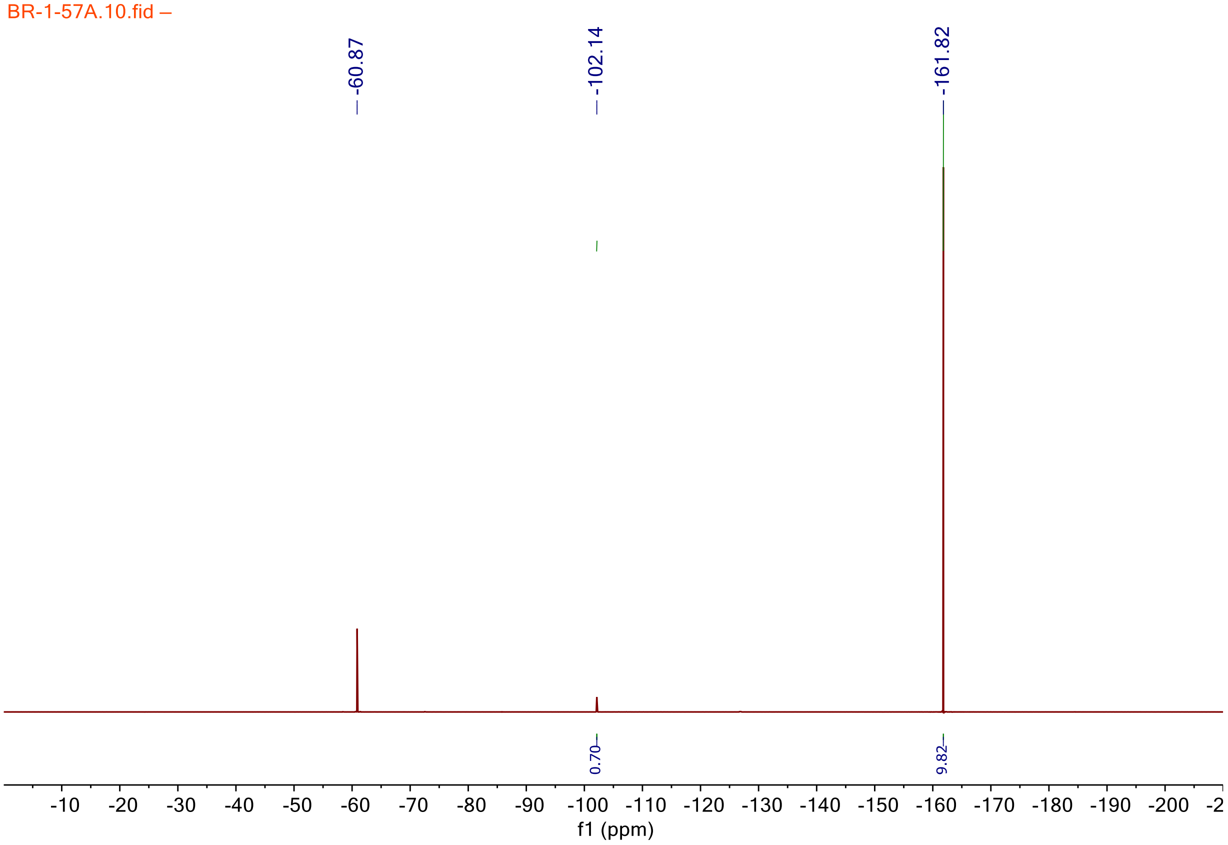

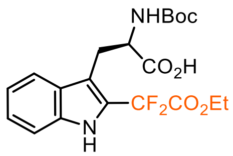

(*R*)-2-((*tert*-butoxycarbonyl)amino)-3-(2-(2-ethoxy-1,1-difluoro-2-oxoethyl)-1*H*-indol-3-yl)propanoic acid (12): ^{1}H NMR (800 MHz, DMSO-D$_6$)

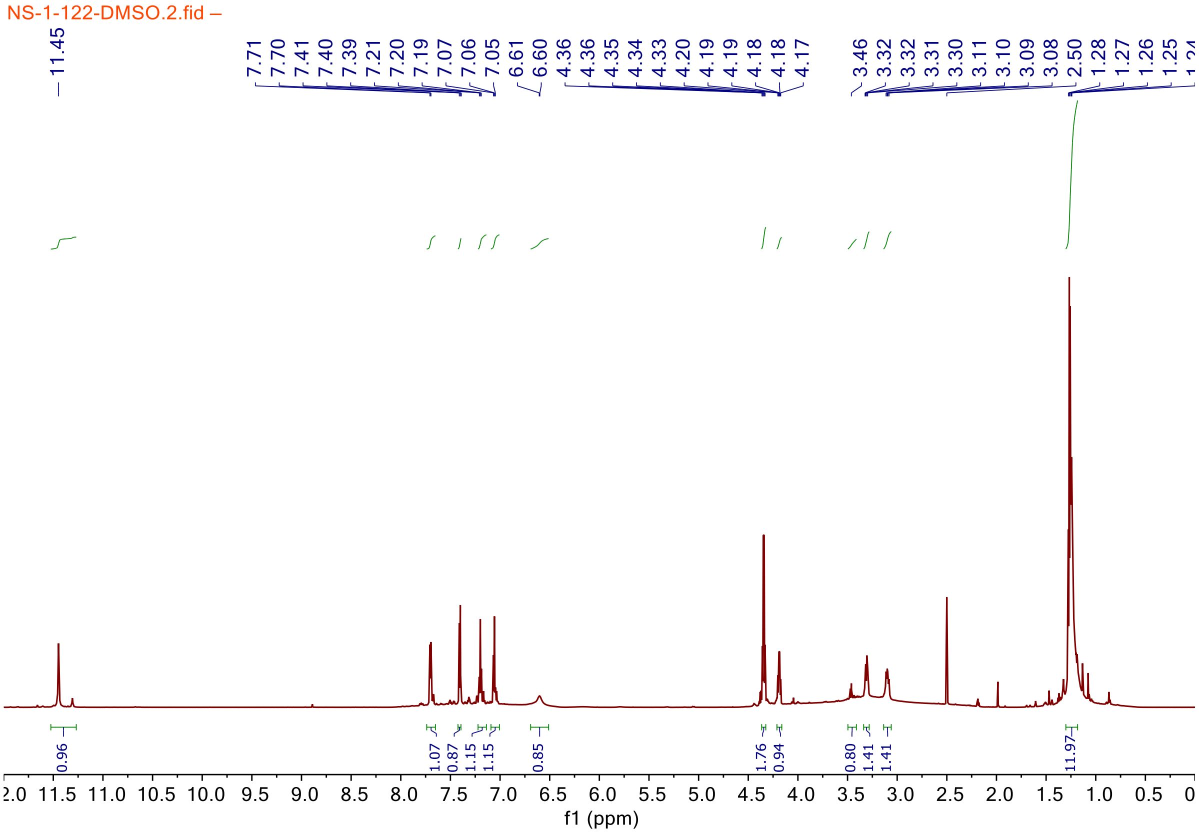

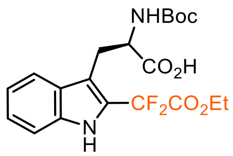

(*R*)-2-((*tert*-butoxycarbonyl)amino)-3-(2-(2-ethoxy-1,1-difluoro-2-oxoethyl)-1*H*-indol-3-yl)propanoic acid (12): $^{13}C\{^{1}H\}$ NMR (201 MHz, DMSO-D_6)

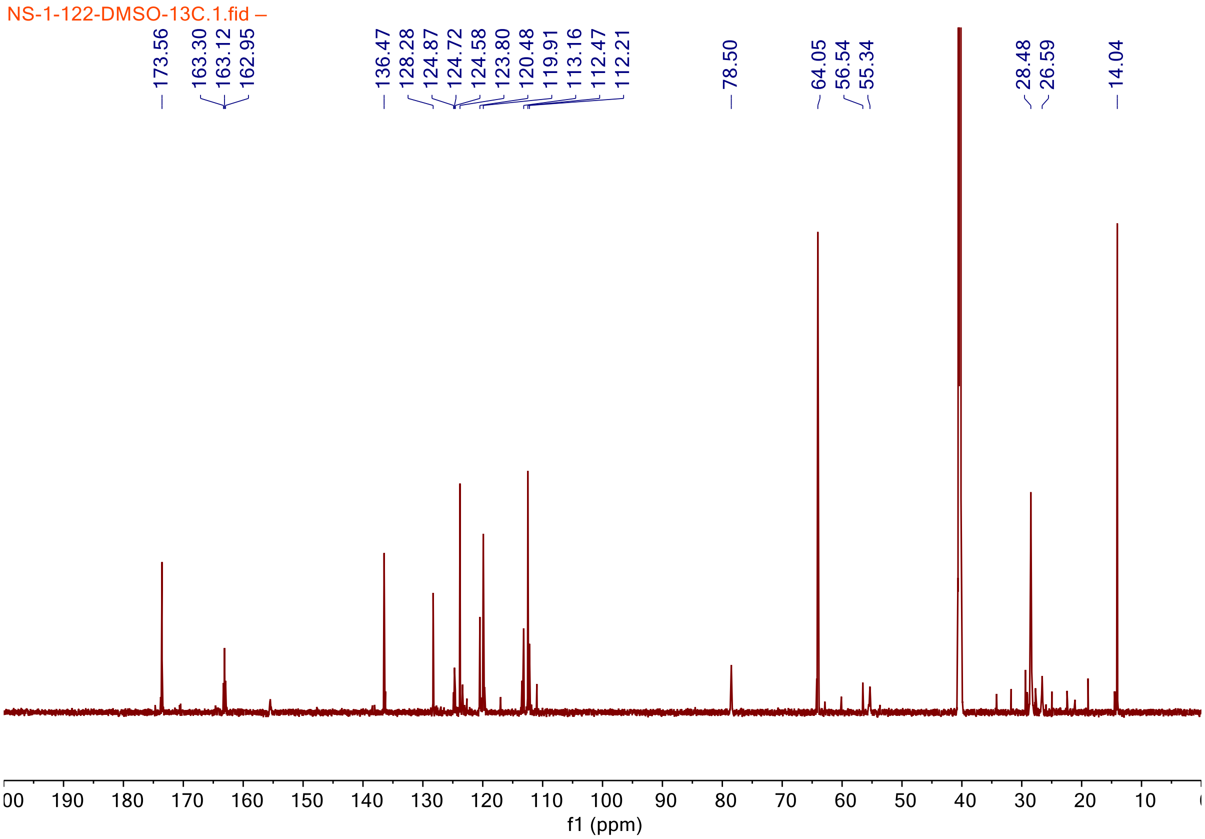

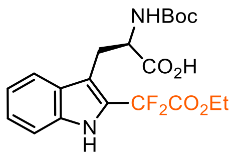

(*R*)-2-((*tert*-butoxycarbonyl)amino)-3-(2-(2-ethoxy-1,1-difluoro-2-oxoethyl)-1*H*-indol-3-yl)propanoic acid (12): ^{19}F NMR (753 MHz, DMSO-D$_6$)

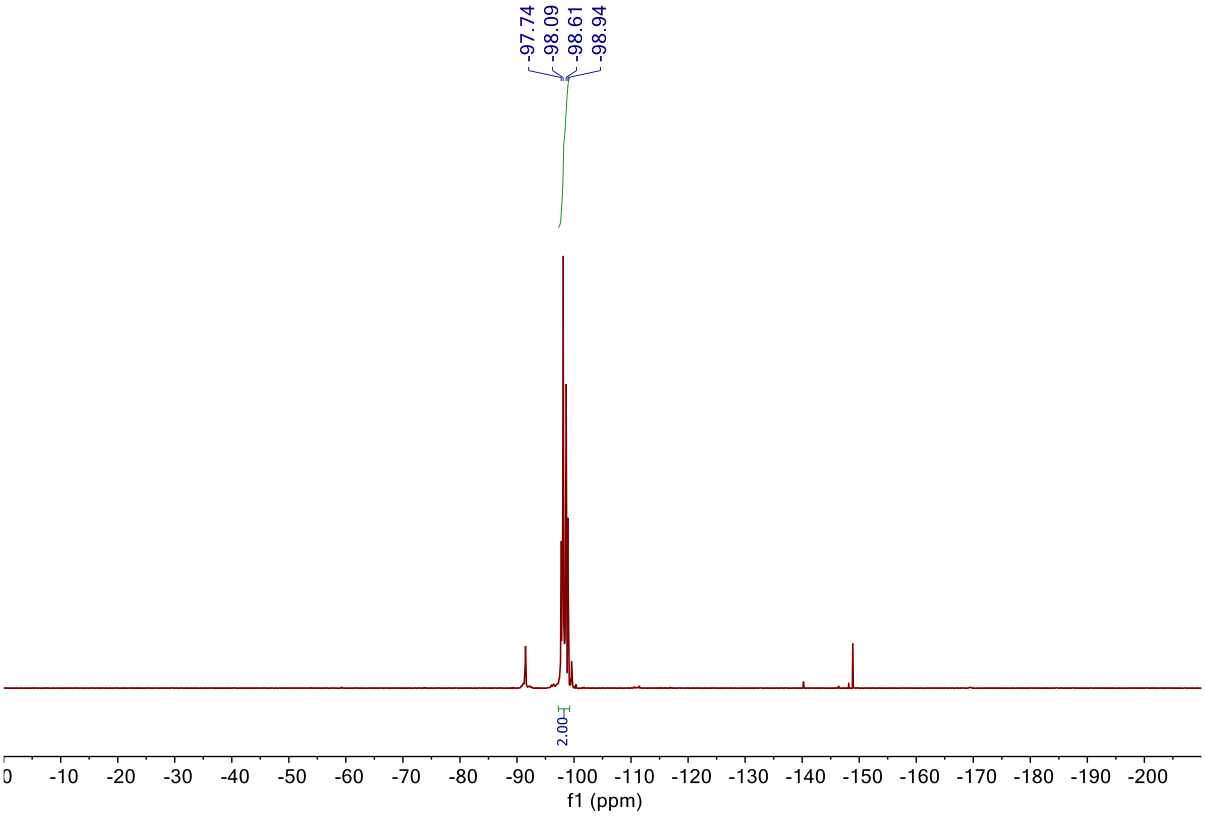

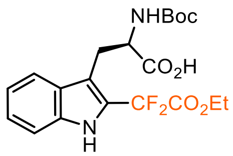

(*R*)-2-((*tert*-butoxycarbonyl)amino)-3-(2-(2-ethoxy-1,1-difluoro-2-oxoethyl)-1*H*-indol-3-yl)propanoic acid (12): ^{19}F NMR (376 MHz, $CDCl_3$) for control reaction without Bu_4NI

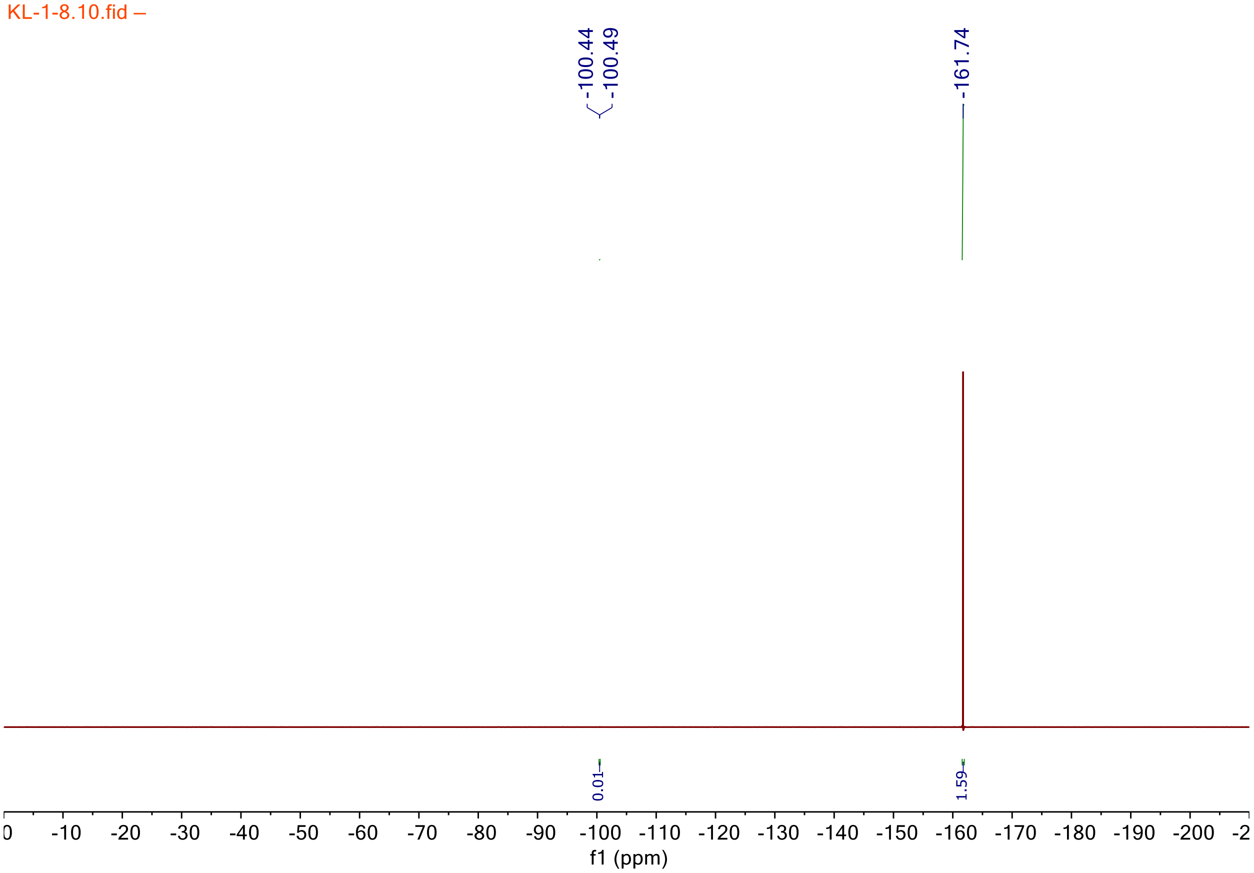

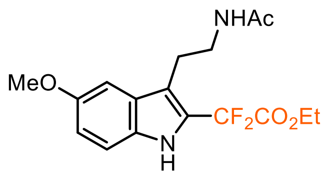

Ethyl 2-(3-(2-acetamidoethyl)-5-methoxy-1*H*-indol-2-yl)-2,2-difluoroacetate (13): ^{1}H NMR (400 MHz, $CDCl_3$)

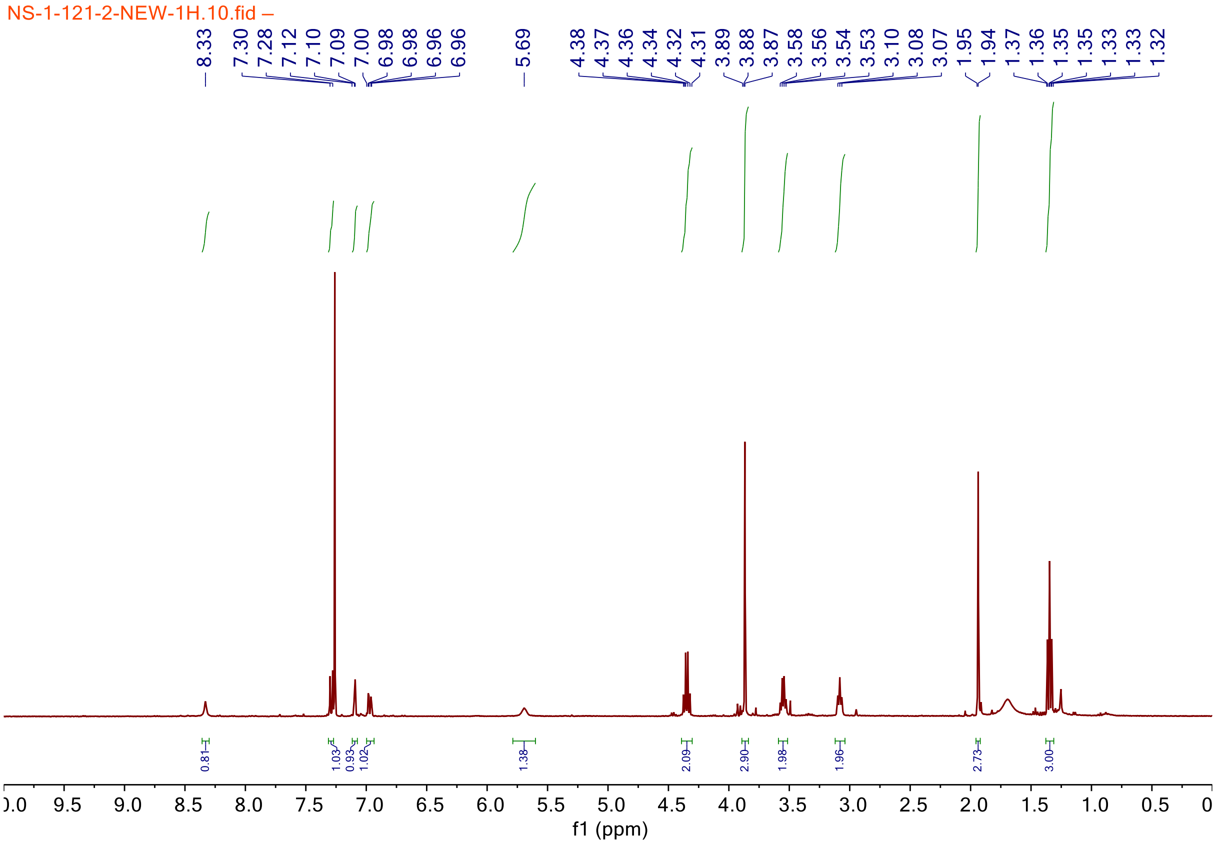

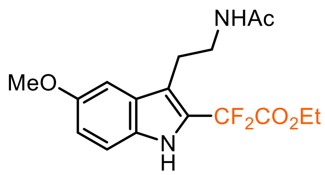

Ethyl 2-(3-(2-acetamidoethyl)-5-methoxy-1*H*-indol-2-yl)-2,2-difluoroacetate (13): $^{13}C\{^{1}H\}$ NMR (201 MHz, $CDCl_3$)

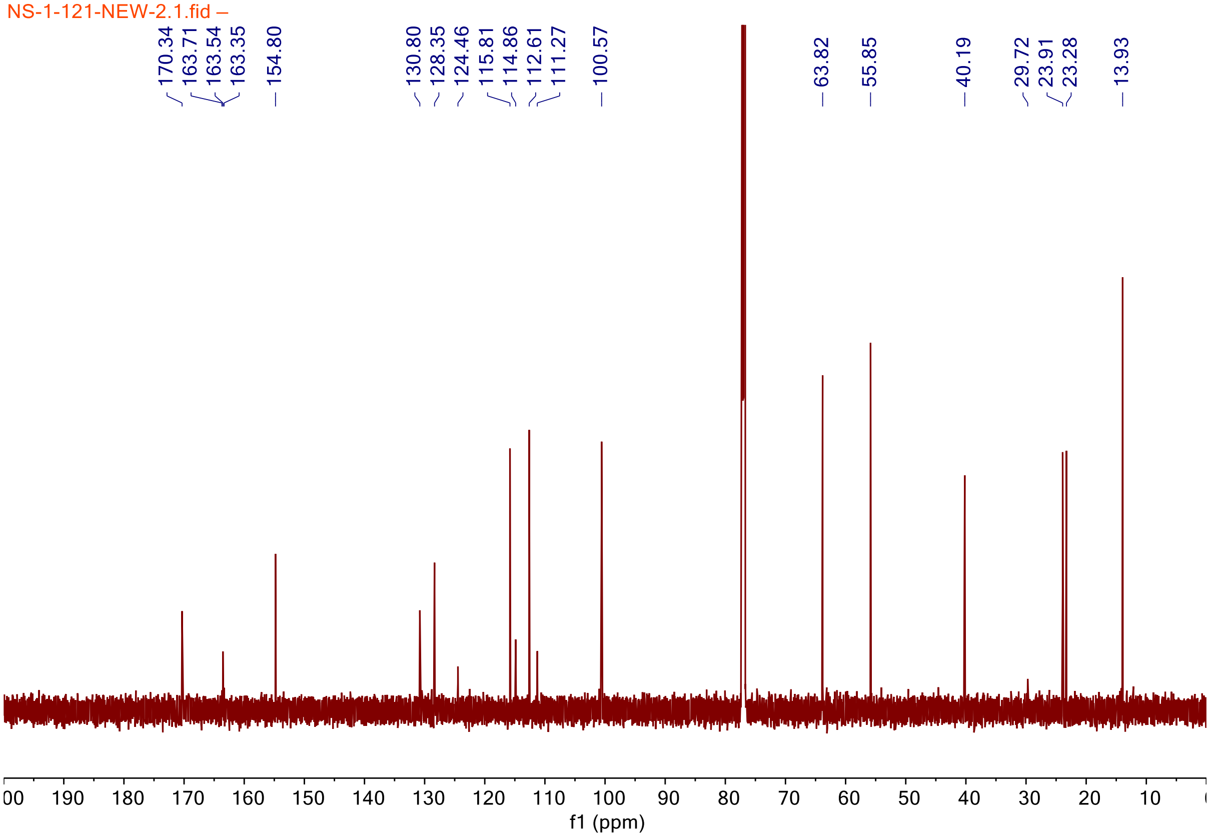

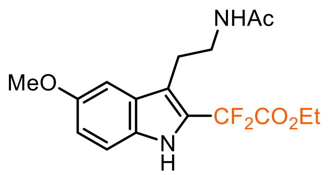

Ethyl 2-(3-(2-acetamidoethyl)-5-methoxy-1*H*-indol-2-yl)-2,2-difluoroacetate (13): ^{19}F NMR (376 MHz, $CDCl_3$)

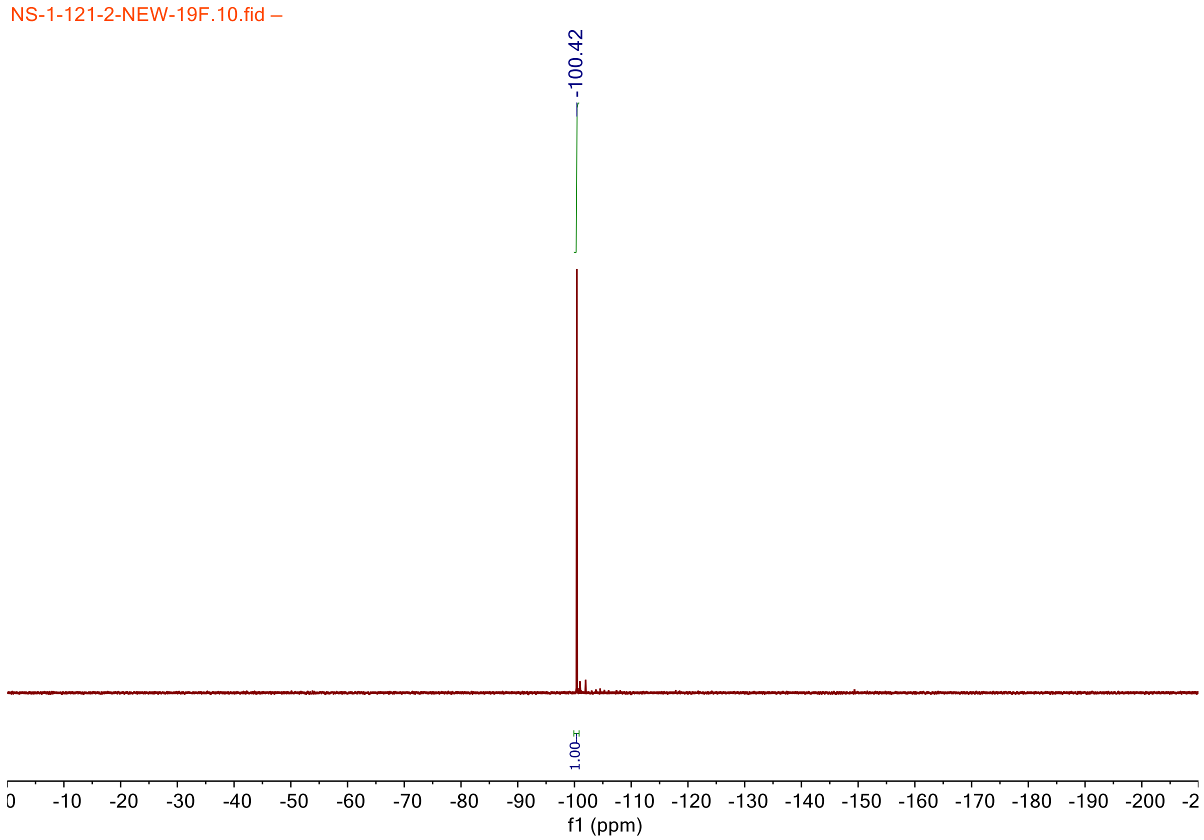

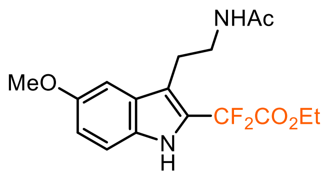

Ethyl 2-(3-(2-acetamidoethyl)-5-methoxy-1*H*-indol-2-yl)-2,2-difluoroacetate (13): ^{19}F NMR (376 MHz, $CDCl_3$) for control reaction without Bu_4NI

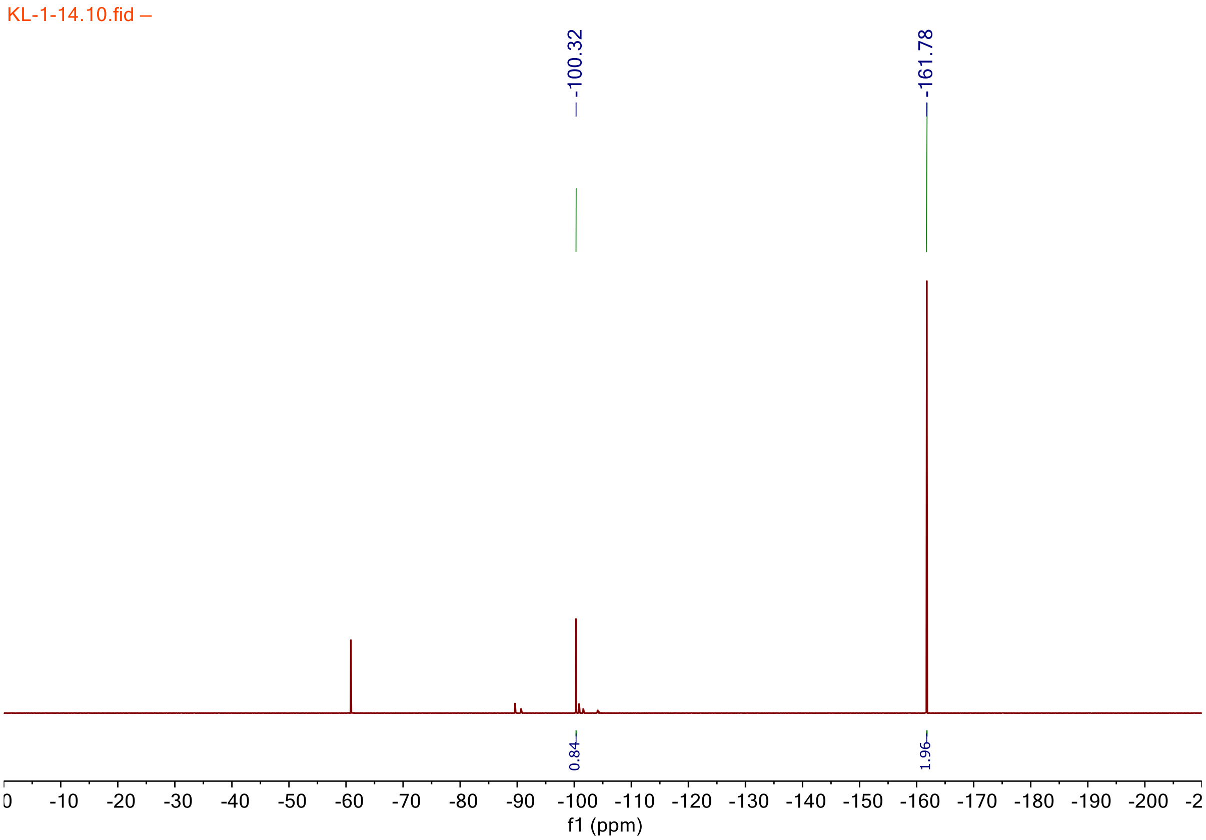

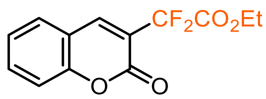

Ethyl 2,2-difluoro-2-(2-oxo-2*H*-chromen-3-yl)acetate (14): ^{1}H NMR (400 MHz, $CDCl_3$)

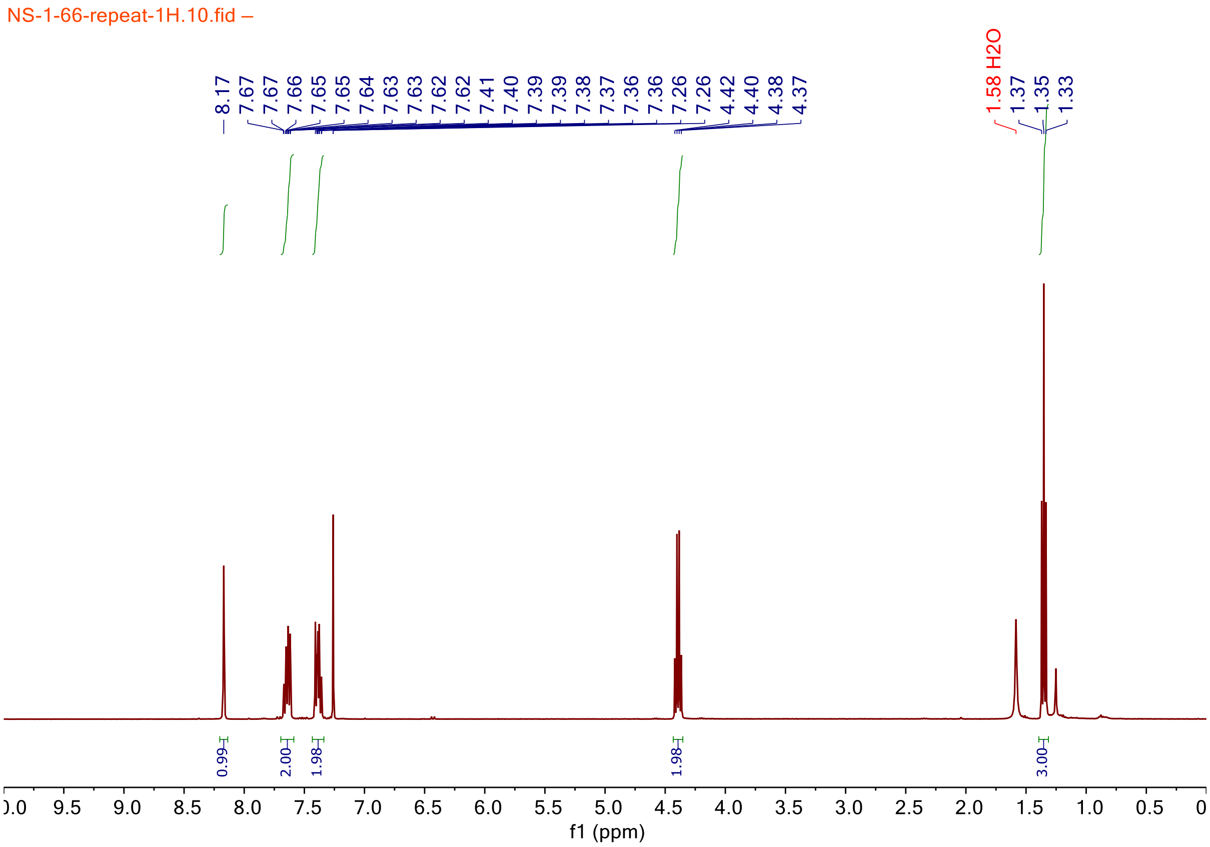

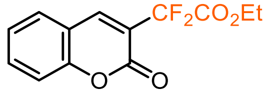

Ethyl 2,2-difluoro-2-(2-oxo-2*H*-chromen-3-yl)acetate (14): $^{13}C\{^{1}H\}$ NMR (201 MHz, $CDCl_3$)

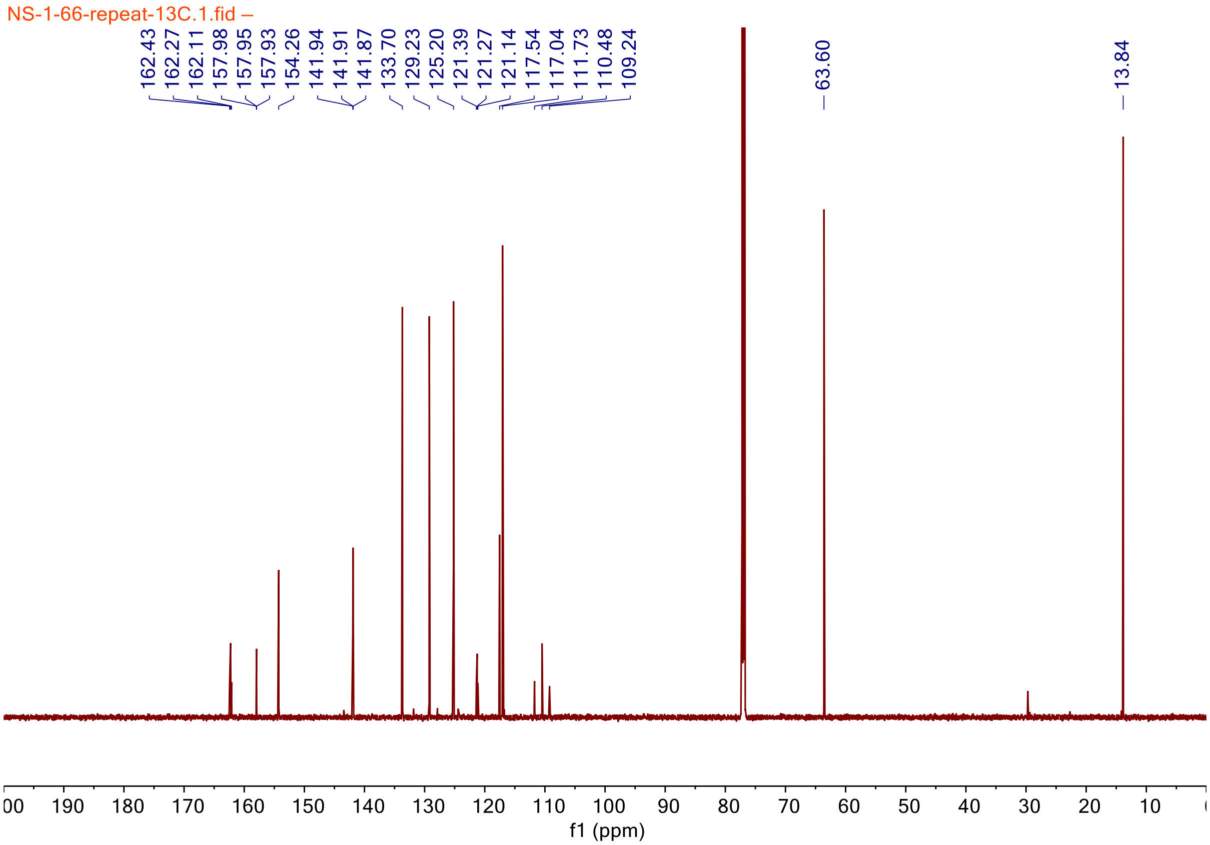

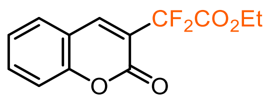

Ethyl 2,2-difluoro-2-(2-oxo-2*H*-chromen-3-yl)acetate (14): ^{19}F NMR (376 MHz, $CDCl_3$)

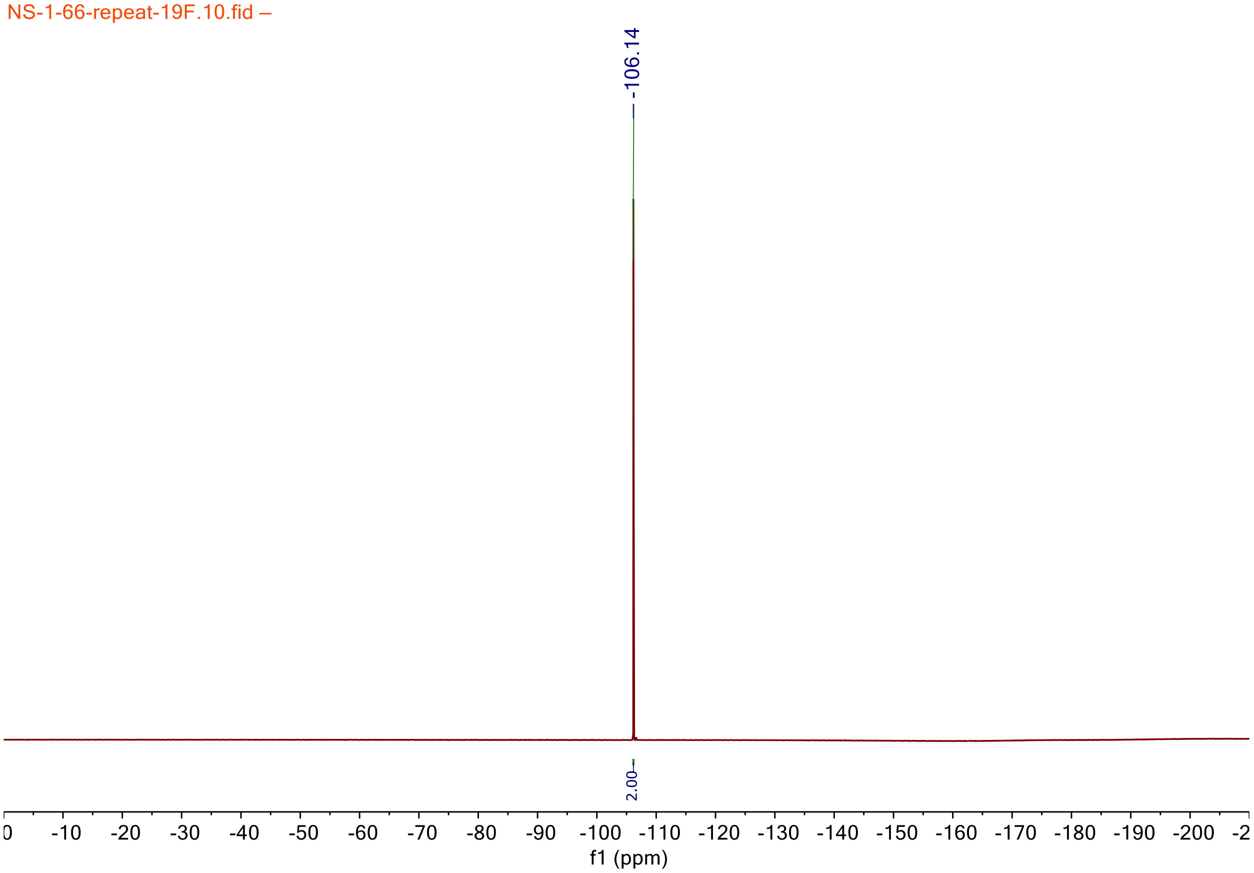

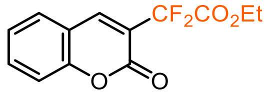

Ethyl 2,2-difluoro-2-(2-oxo-2*H*-chromen-3-yl)acetate (14) ^{19}F NMR (376 MHz, $CDCl_3$) for control reaction without Bu_4NI

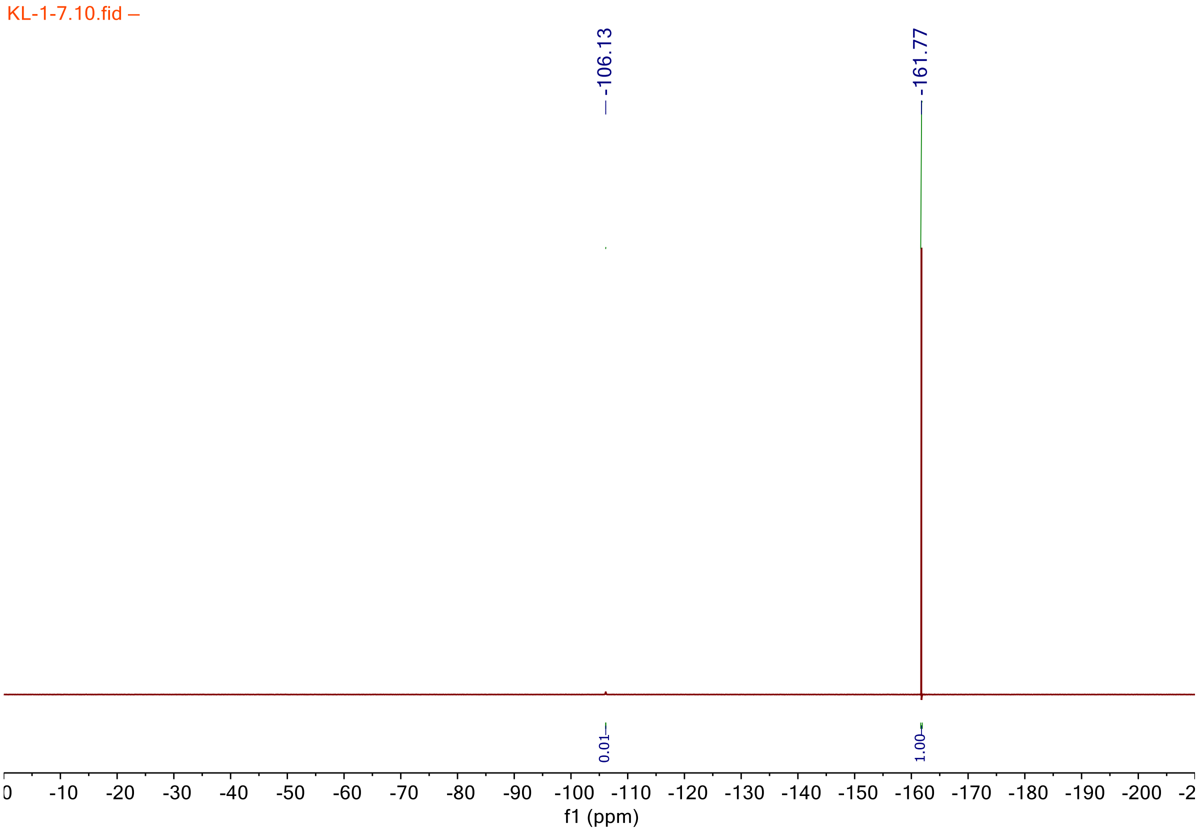

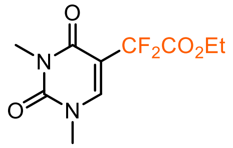

Ethyl 2-(1,3-dimethyl-2,4-dioxo-1,2,3,4-tetrahydropyrimidin-5-yl)-2,2-difluoroacetate (15):

^{1}H NMR (400 MHz, $CDCl_3$)

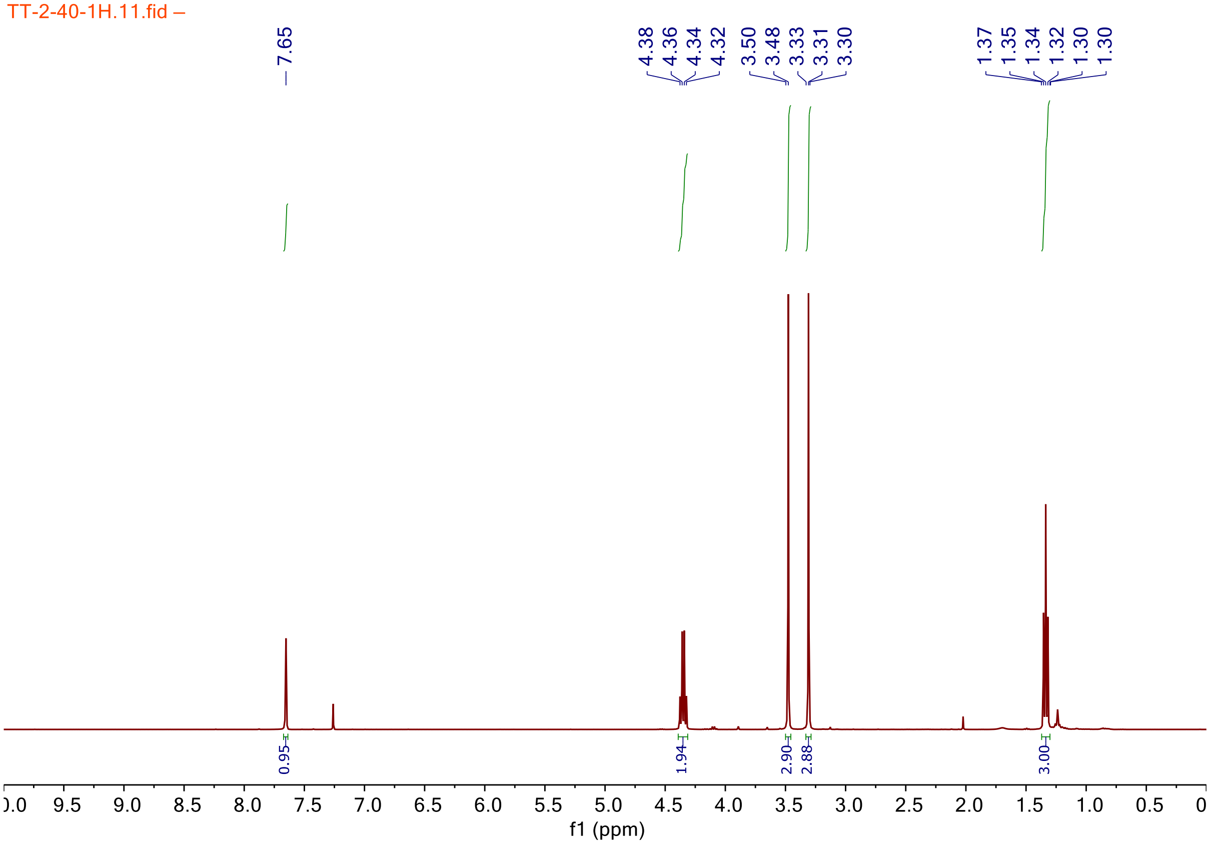

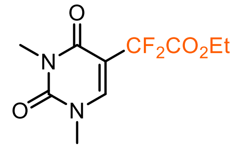

Ethyl 2-(1,3-dimethyl-2,4-dioxo-1,2,3,4-tetrahydropyrimidin-5-yl)-2,2-difluoroacetate (15):
$^{13}C\{^1H\}$ NMR (201 MHz, $CDCl_3$)

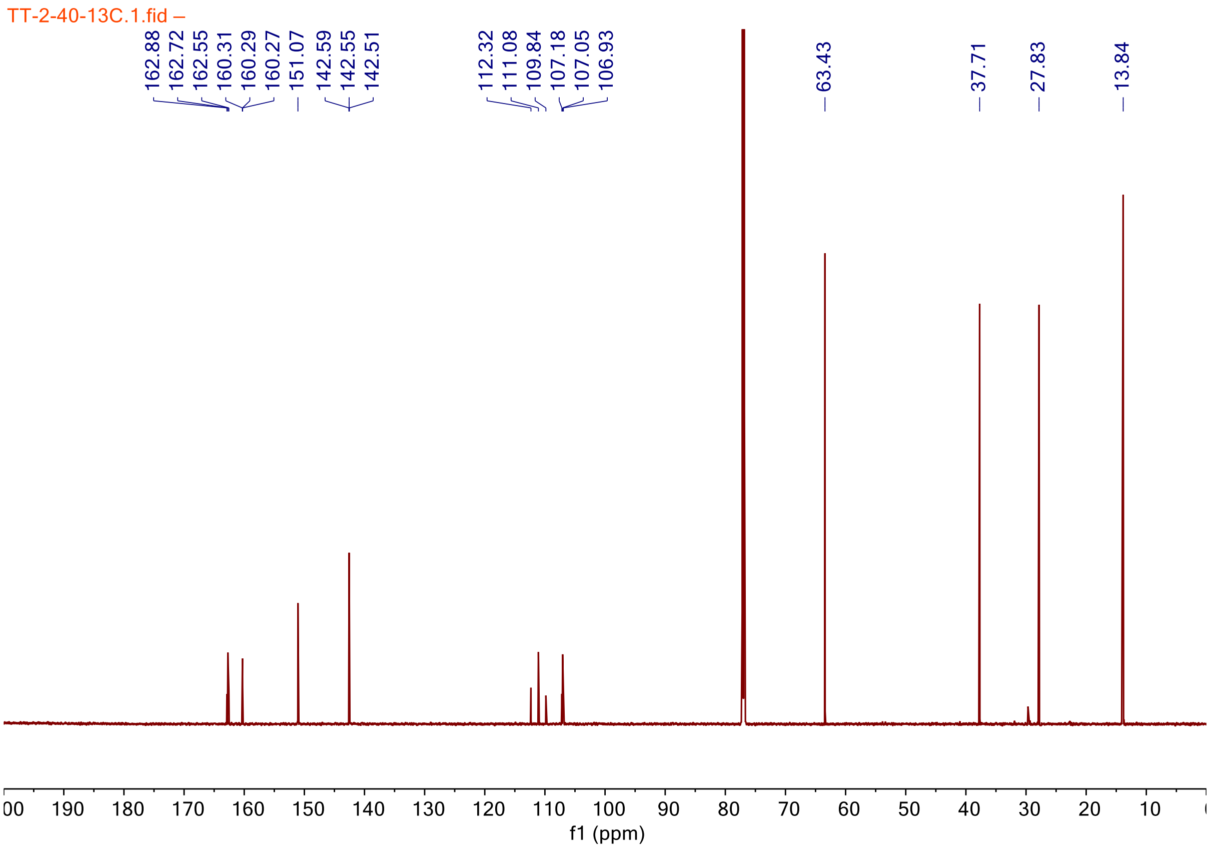

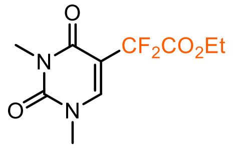

Ethyl 2-(1,3-dimethyl-2,4-dioxo-1,2,3,4-tetrahydropyrimidin-5-yl)-2,2-difluoroacetate (15):
^{19}F NMR (376 MHz, $CDCl_3$)

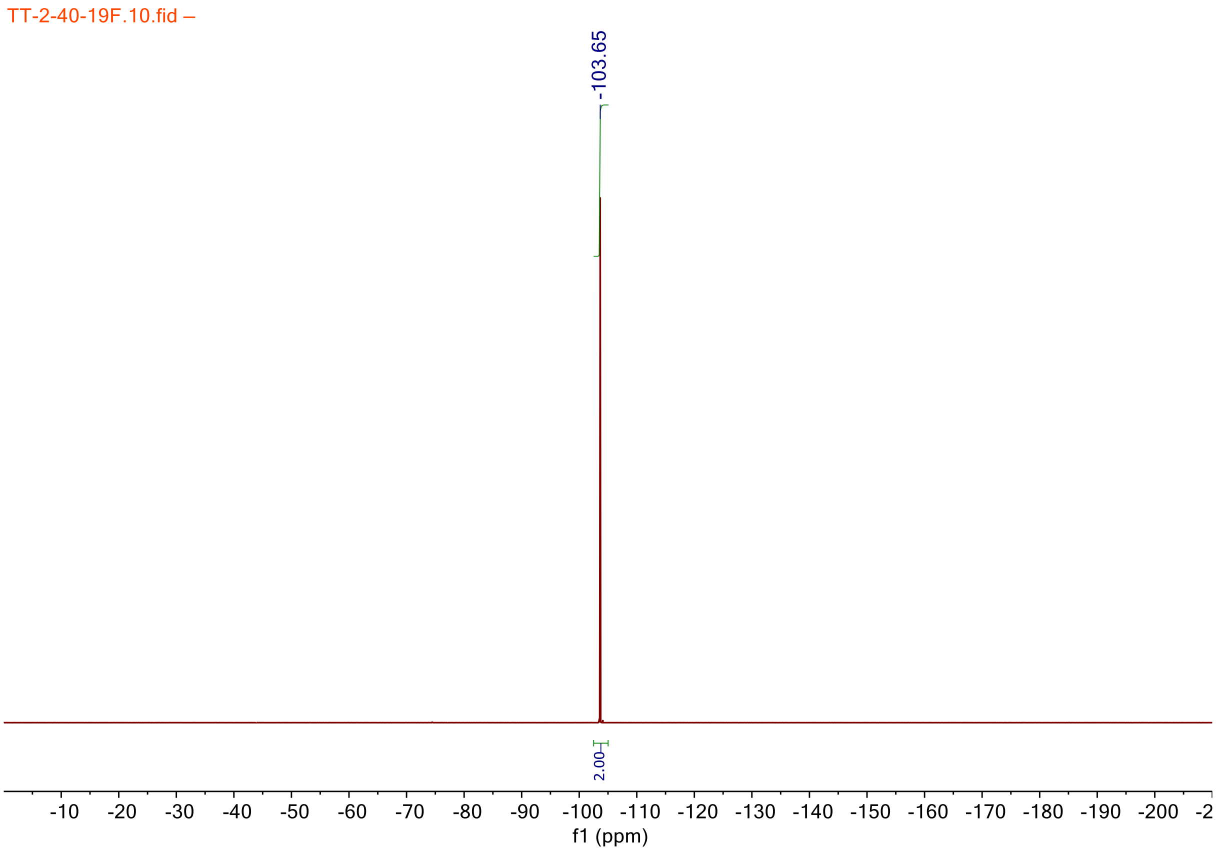

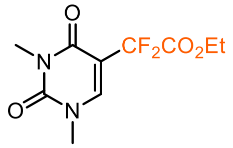

Ethyl 2-(1,3-dimethyl-2,4-dioxo-1,2,3,4-tetrahydropyrimidin-5-yl)-2,2-difluoroacetate (15):
^{19}F NMR (376 MHz, $CDCl_3$) for control reaction without Bu_4NI

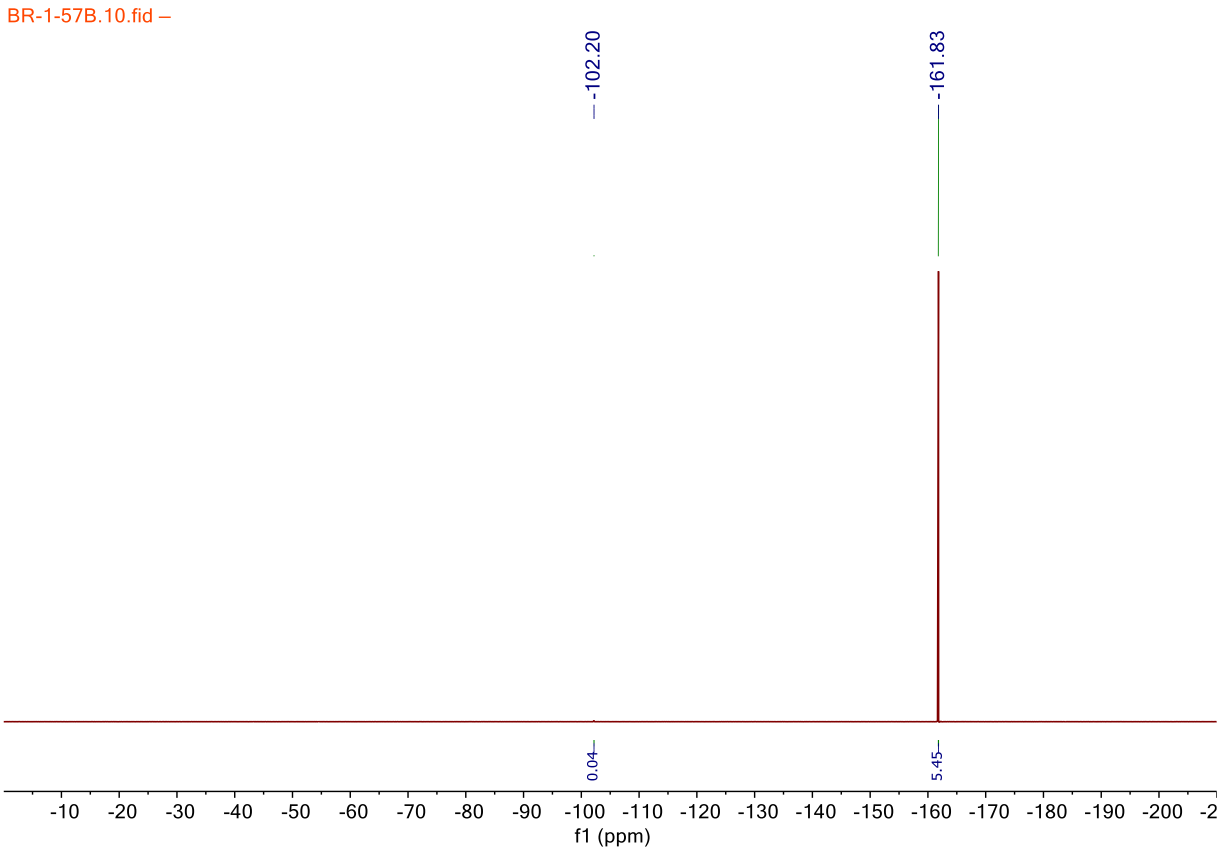

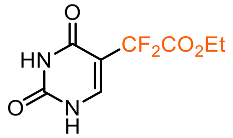

Ethyl 2-(2, 4-dioxo-1, 2, 3, 4-tetrahydropyrimidin-5-yl)-2, 2-difluoroacetate (16): ^{1}H NMR (400 MHz, Acetone-D_6)

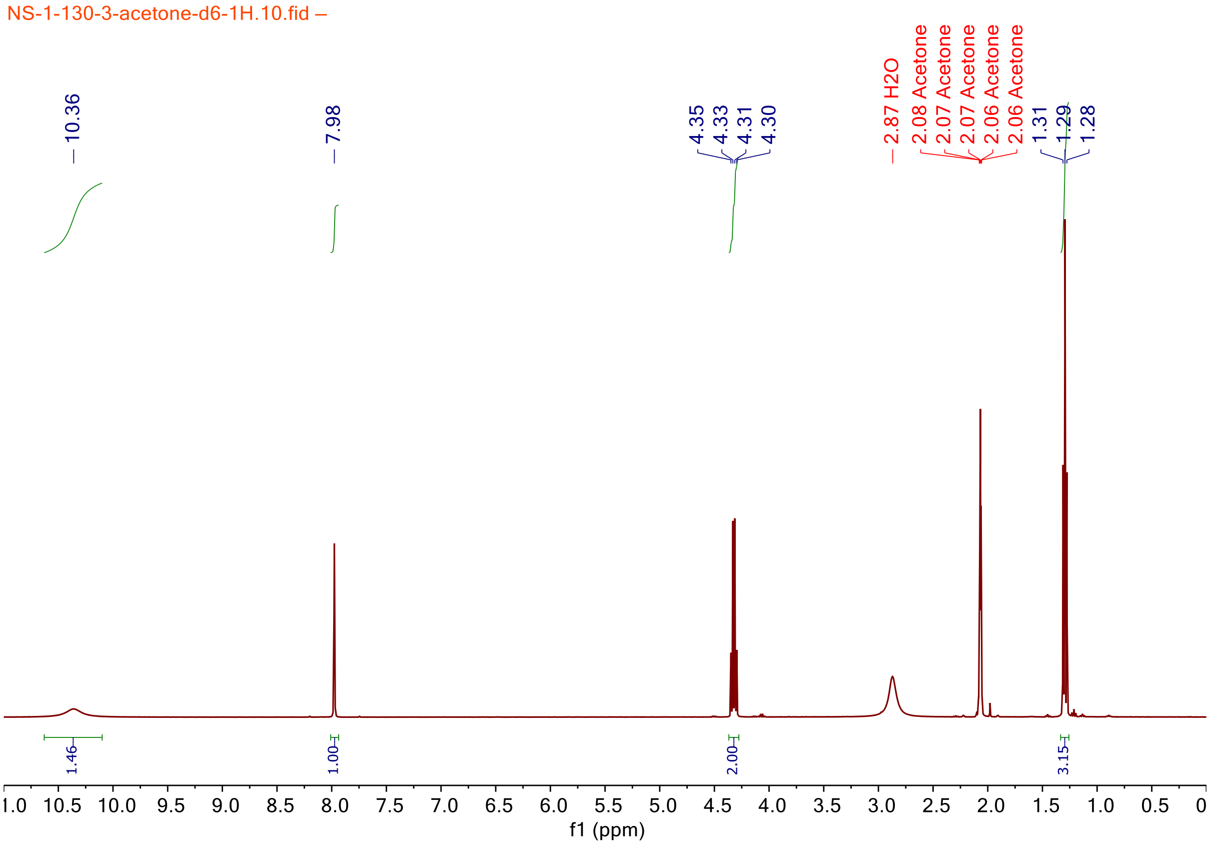

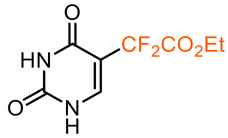

Ethyl 2-(2, 4-dioxo-1, 2, 3, 4-tetrahydropyrimidin-5-yl)-2, 2-difluoroacetate (16): $^{13}C\{^{1}H\}$ NMR (201 MHz, Acetone-D_6)

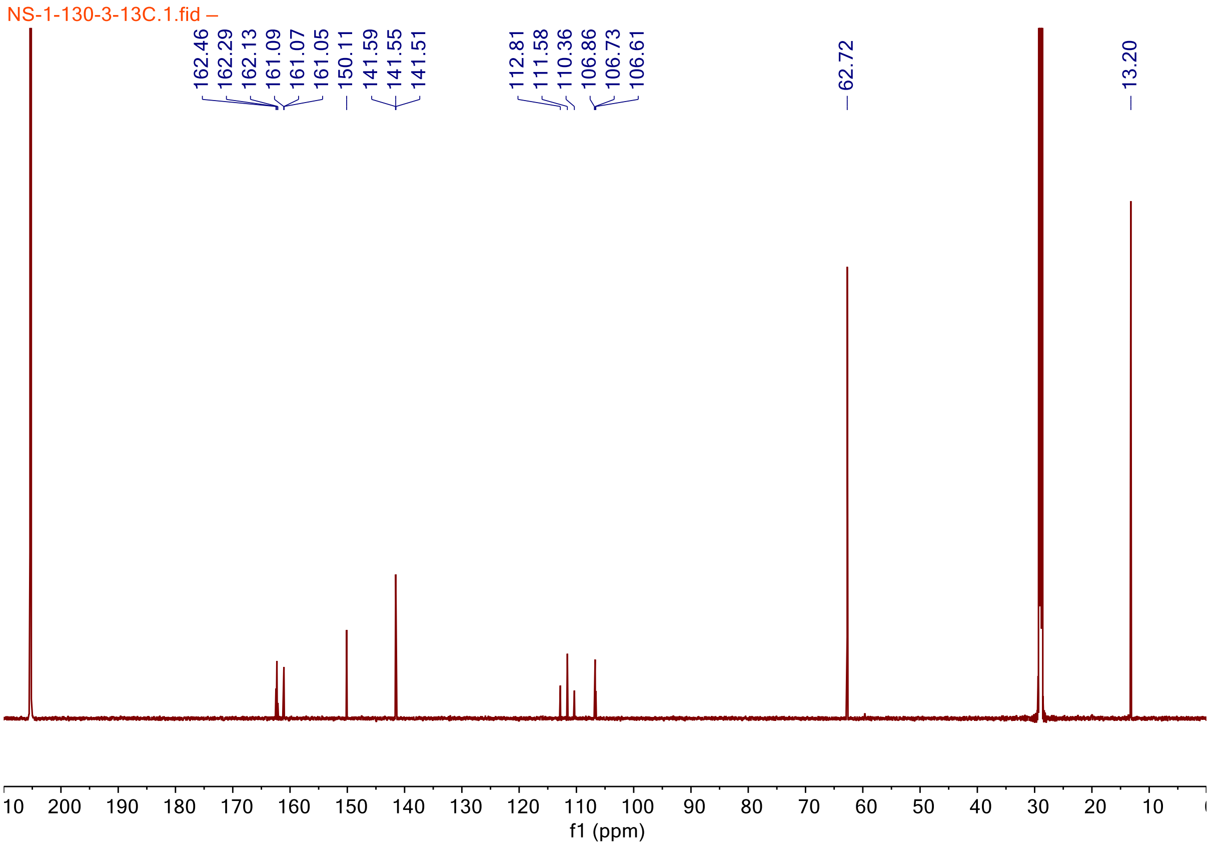

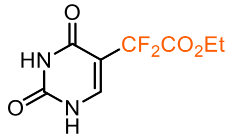

Ethyl 2-(2, 4-dioxo-1, 2, 3, 4-tetrahydropyrimidin-5-yl)-2, 2-difluoroacetate (16): ^{19}F NMR (376 MHz, Acetone-D_6)

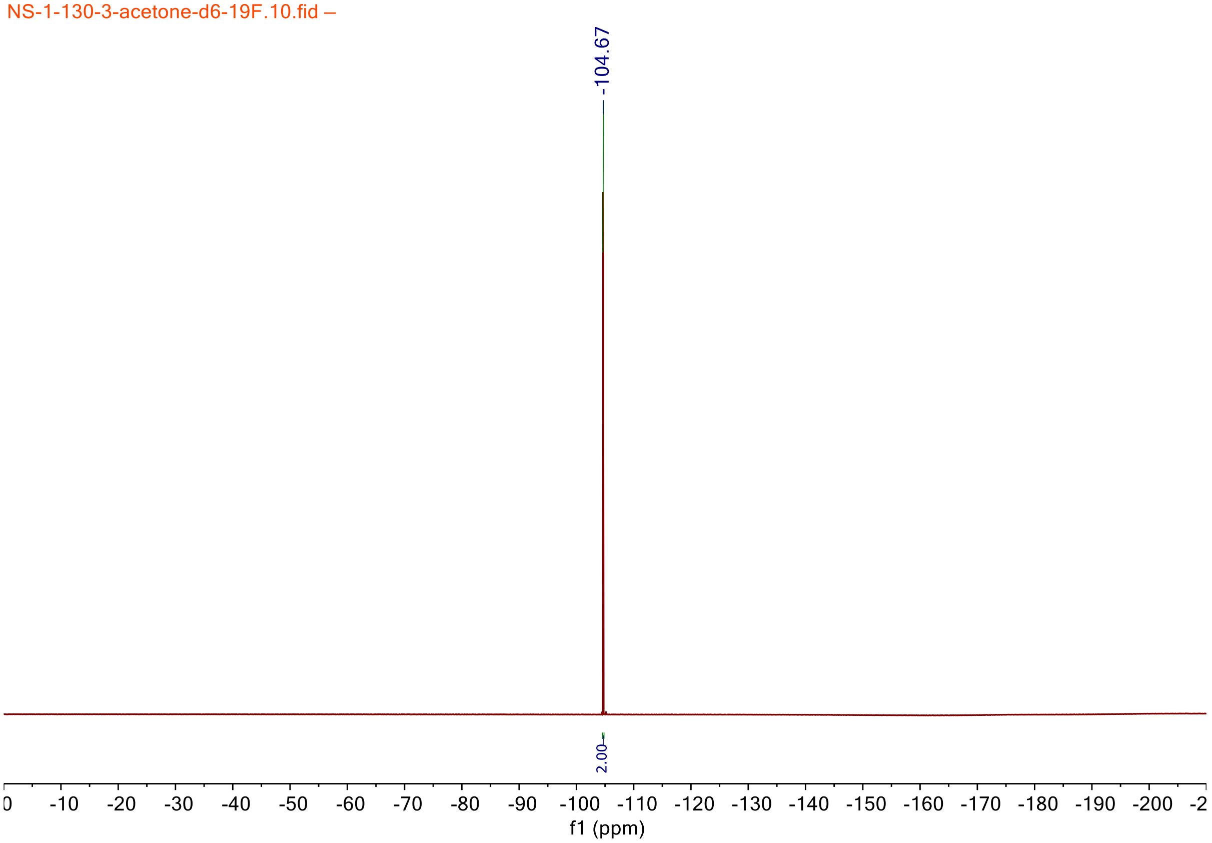

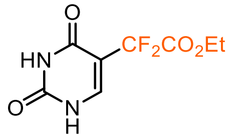

Ethyl 2-(2, 4-dioxo-1, 2, 3, 4-tetrahydropyrimidin-5-yl)-2, 2-difluoroacetate (16): ^{19}F NMR (376 MHz, $CDCl_3$) for control reaction without Bu_4NI

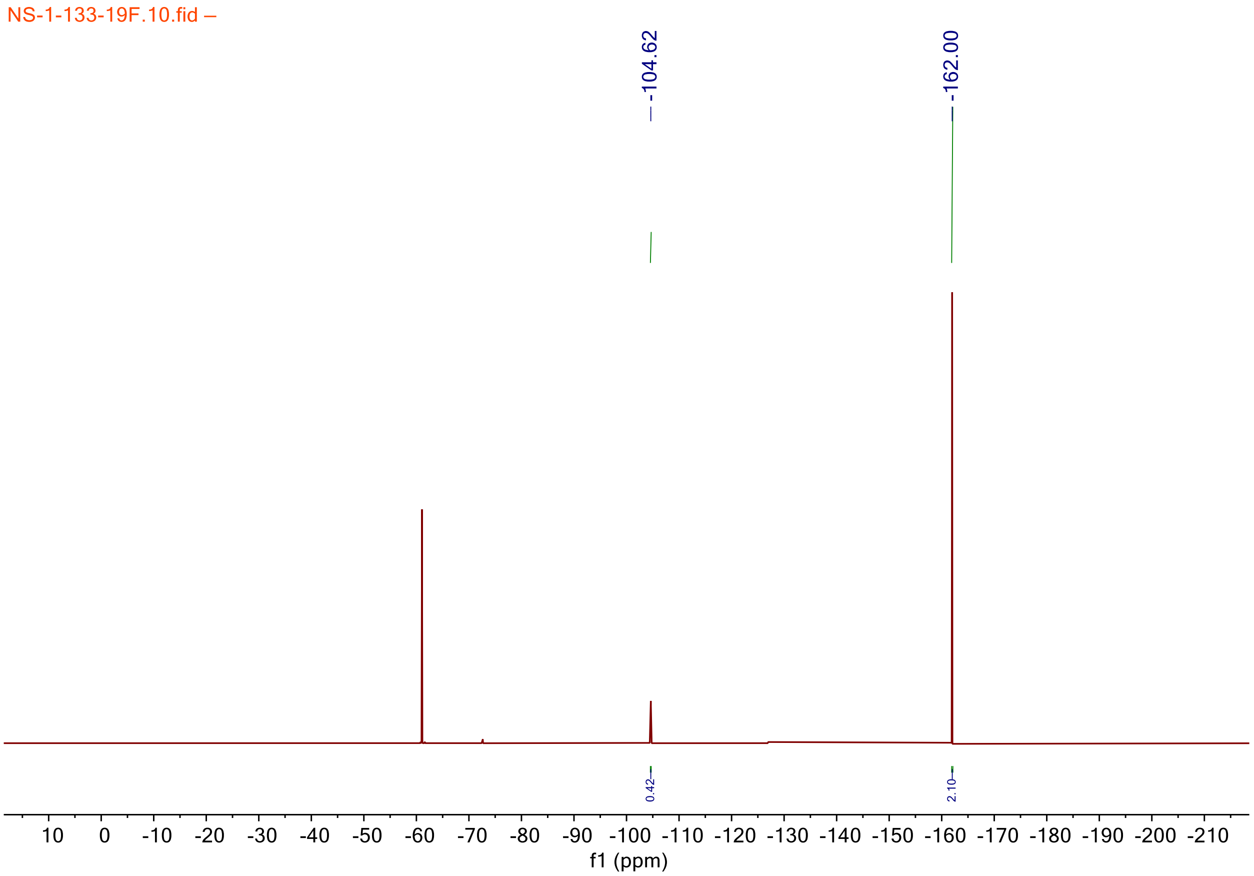

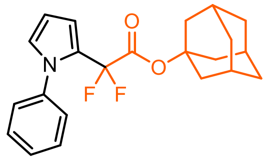

Adamantan-1-yl 2,2-difluoro-2-(1-phenyl-1H-pyrrol-2-yl)acetate (17): ^{1}H NMR (400 MHz, $CDCl_3$)

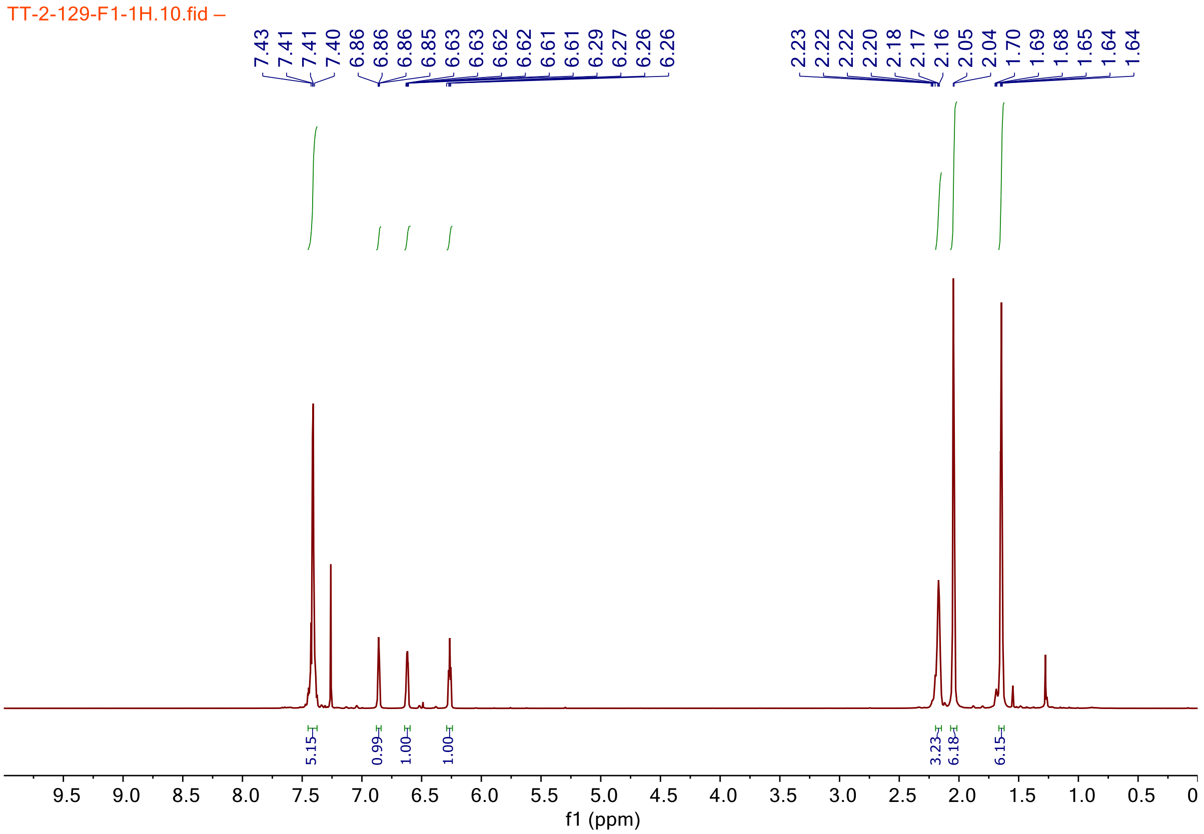

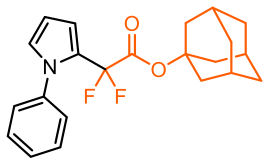

Adamantan-1-yl 2,2-difluoro-2-(1-phenyl-1H-pyrrol-2-yl)acetate (17): $^{13}C\{^{1}H\}$ NMR (201 MHz, $CDCl_3$)

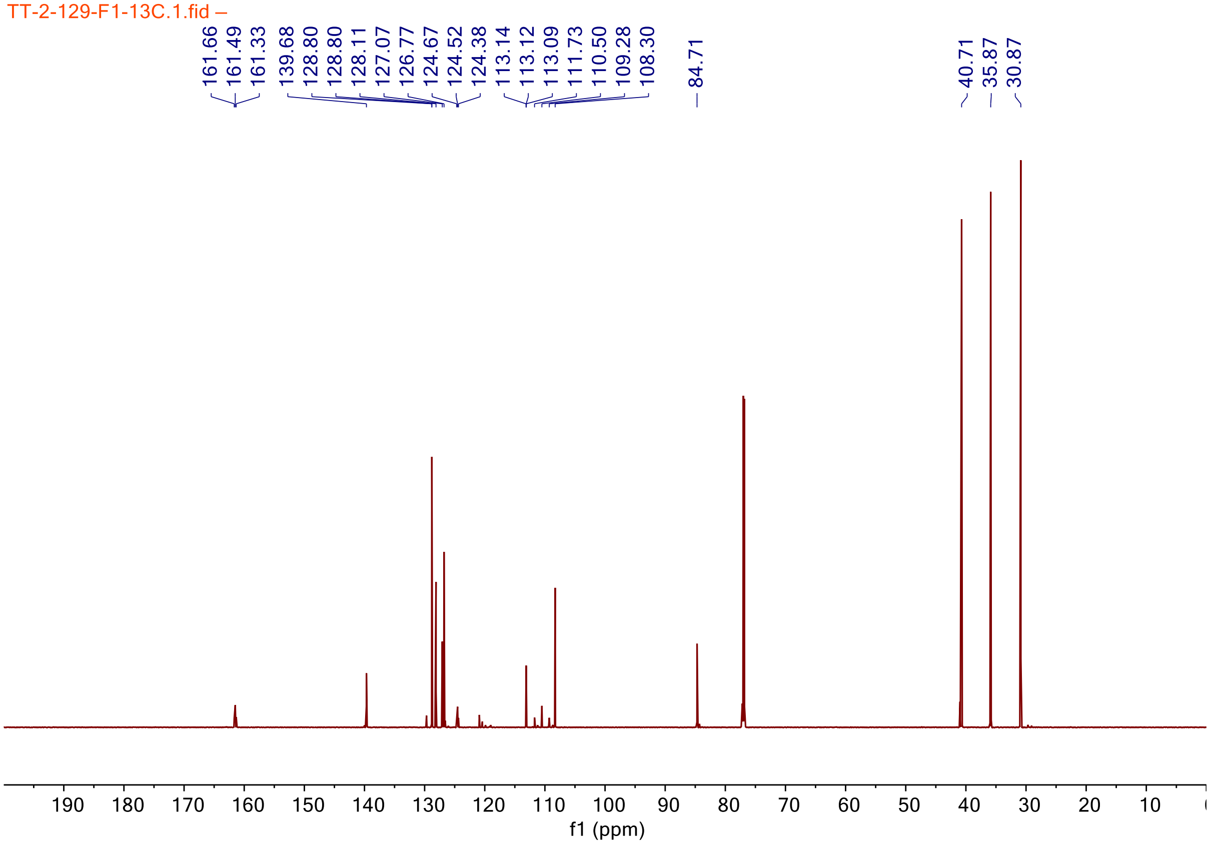

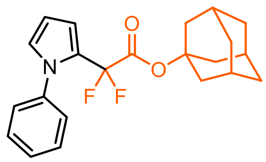

Adamantan-1-yl 2,2-difluoro-2-(1-phenyl-1H-pyrrol-2-yl)acetate (17): ^{19}F NMR (376 MHz, $CDCl_3$)

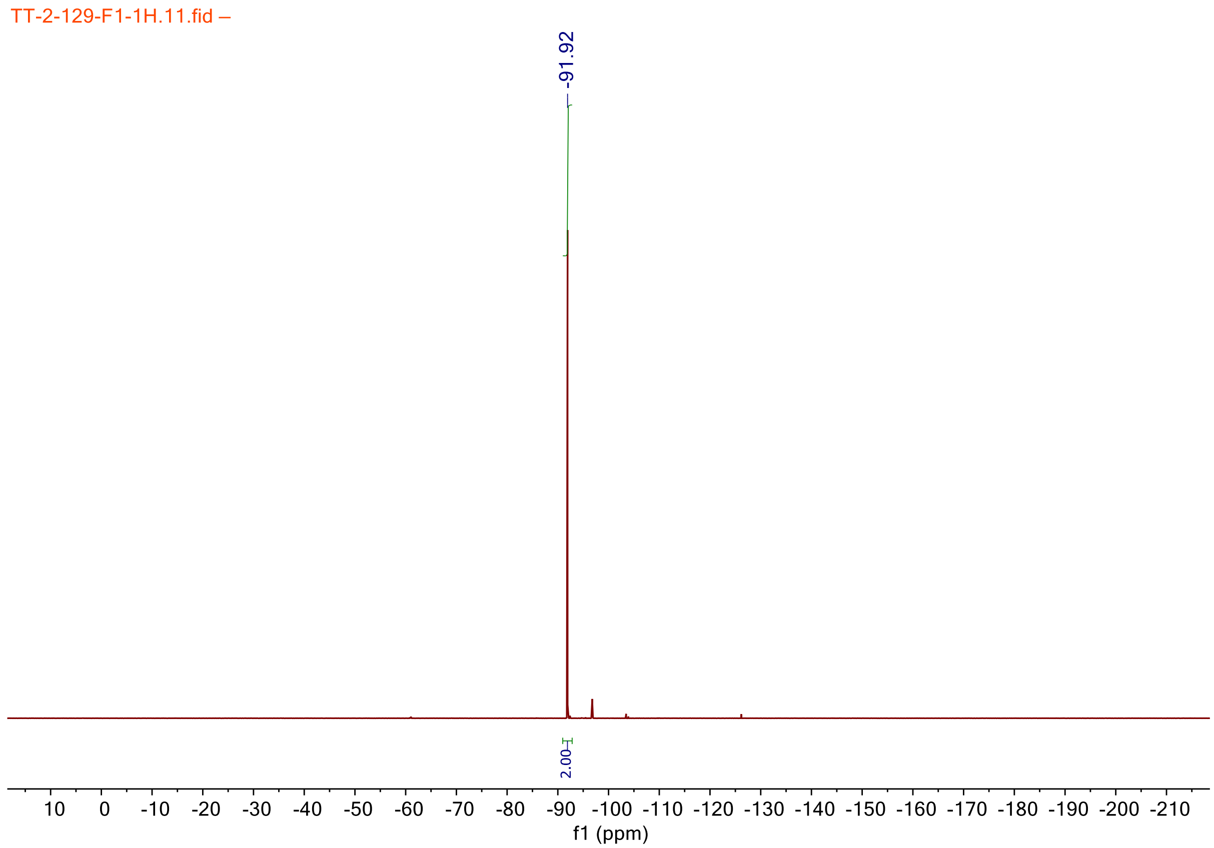

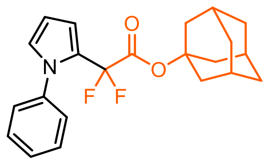

Adamantan-1-yl 2,2-difluoro-2-(1-phenyl-1H-pyrrol-2-yl)acetate (17): ^{19}F NMR (376 MHz, $CDCl_3$) for control reaction without Bu_4NI

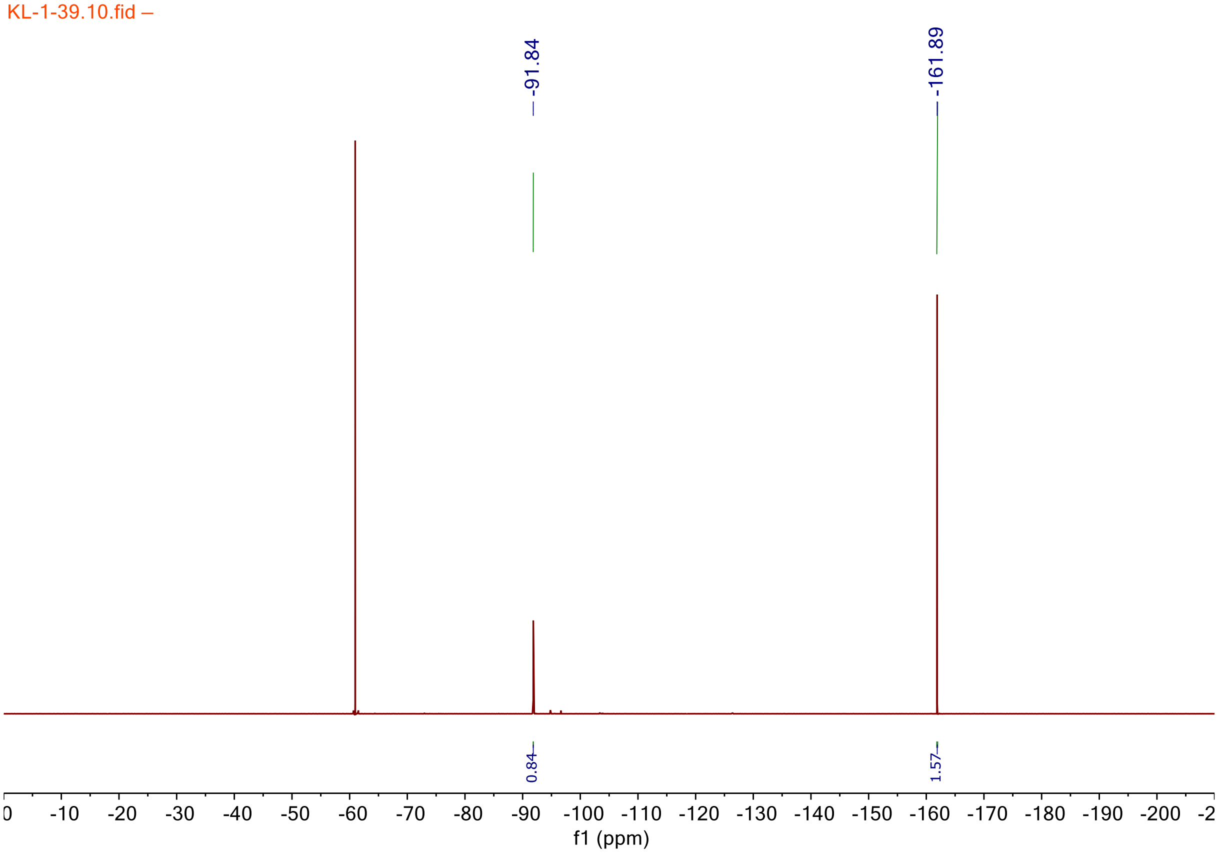

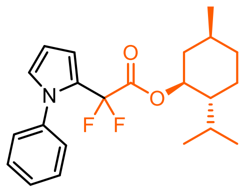

(1*S*,2*R*,5*S*)-2-isopropyl-5-methylcyclohexyl 2,2-difluoro-2-(1-phenyl-1*H*-pyrrol-2-yl)acetate (18): ^{1}H NMR (400 MHz, $CDCl_3$)

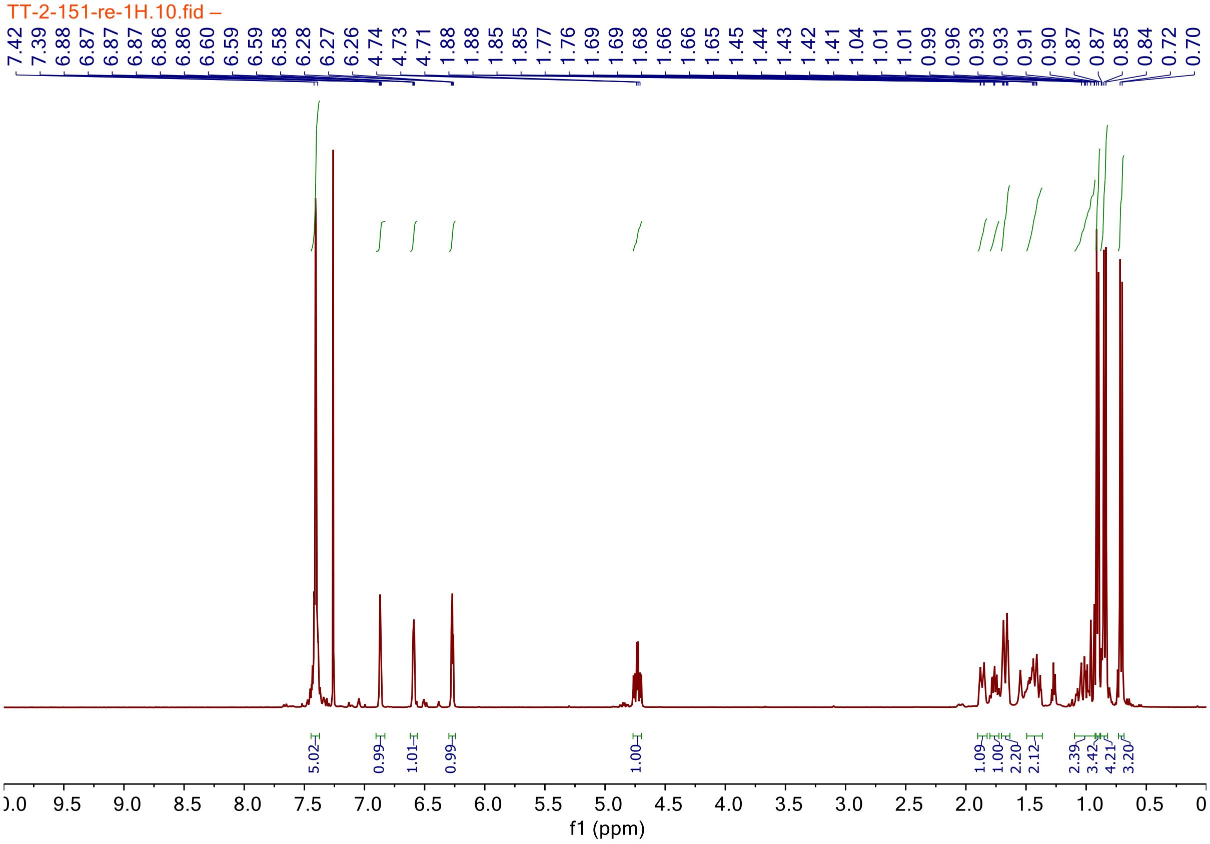

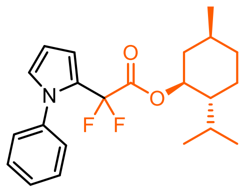

(1*S*,2*R*,5*S*)-2-isopropyl-5-methylcyclohexyl 2,2-difluoro-2-(1-phenyl-1*H*-pyrrol-2-yl)acetate (18): $^{13}C\{^{1}H\}$ NMR (201 MHz, $CDCl_3$)

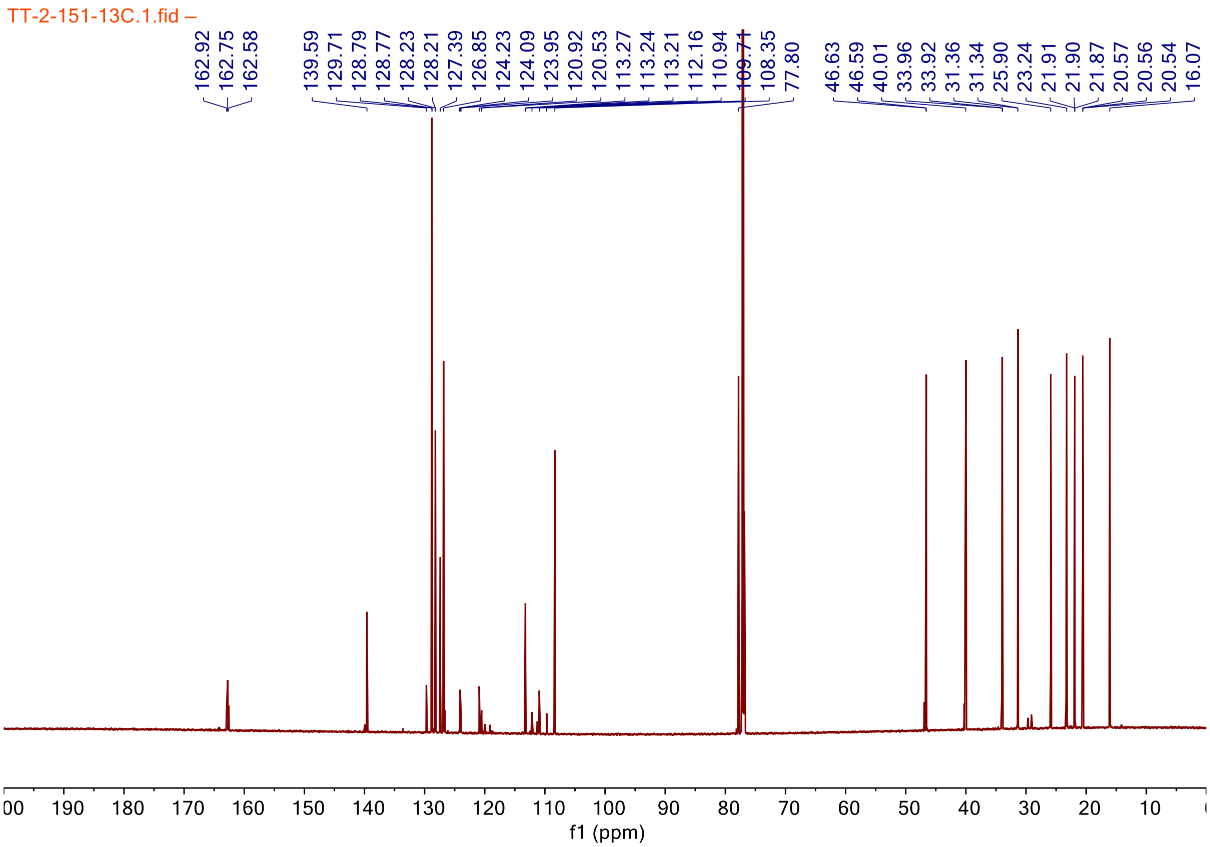

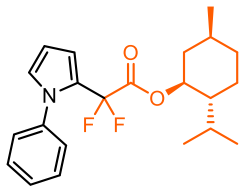

(1*S*,2*R*,5*S*)-2-isopropyl-5-methylcyclohexyl 2,2-difluoro-2-(1-phenyl-1*H*-pyrrol-2-yl)acetate (18): ^{19}F NMR (376 MHz, $CDCl_3$)

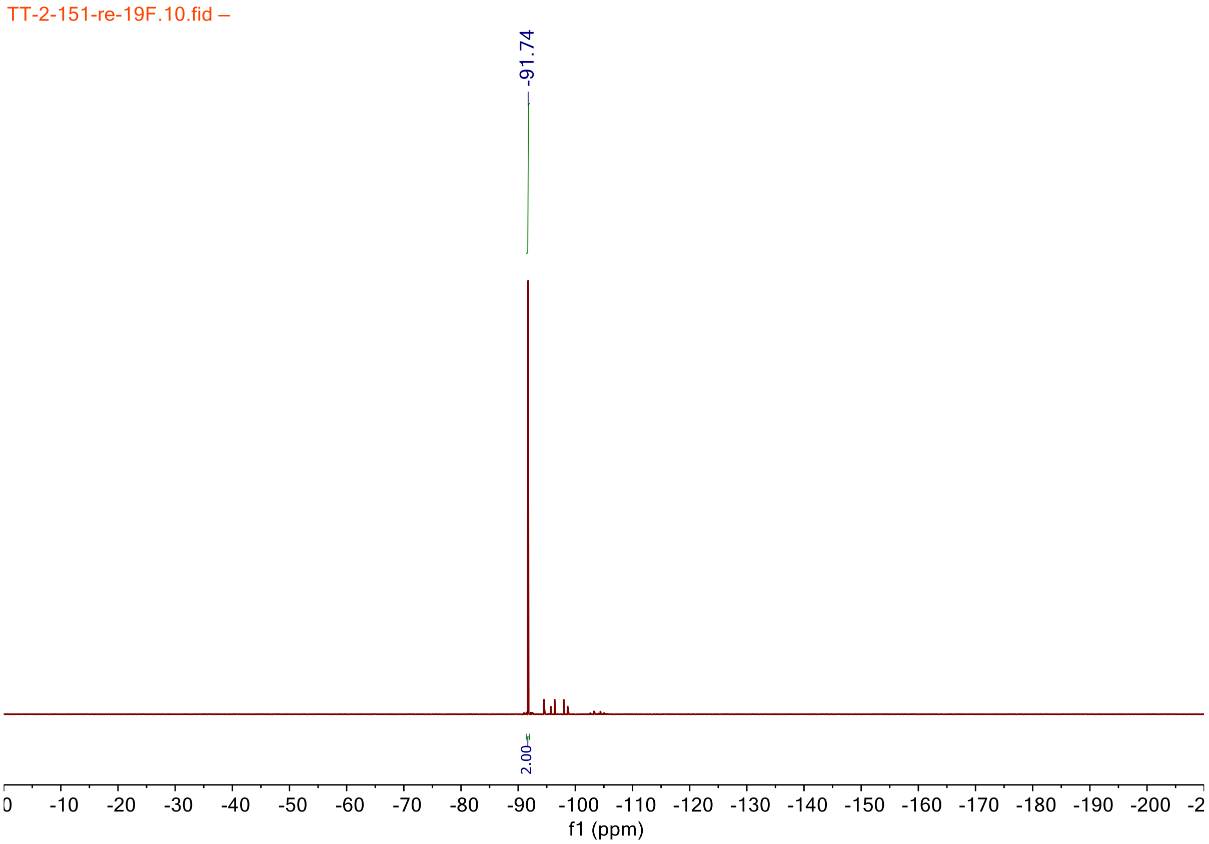

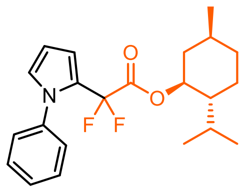

(1*S*,2*R*,5*S*)-2-isopropyl-5-methylcyclohexyl 2,2-difluoro-2-(1-phenyl-1*H*-pyrrol-2-yl)acetate (18): ^{19}F NMR (376 MHz, $CDCl_3$) for control reaction without Bu_4NI

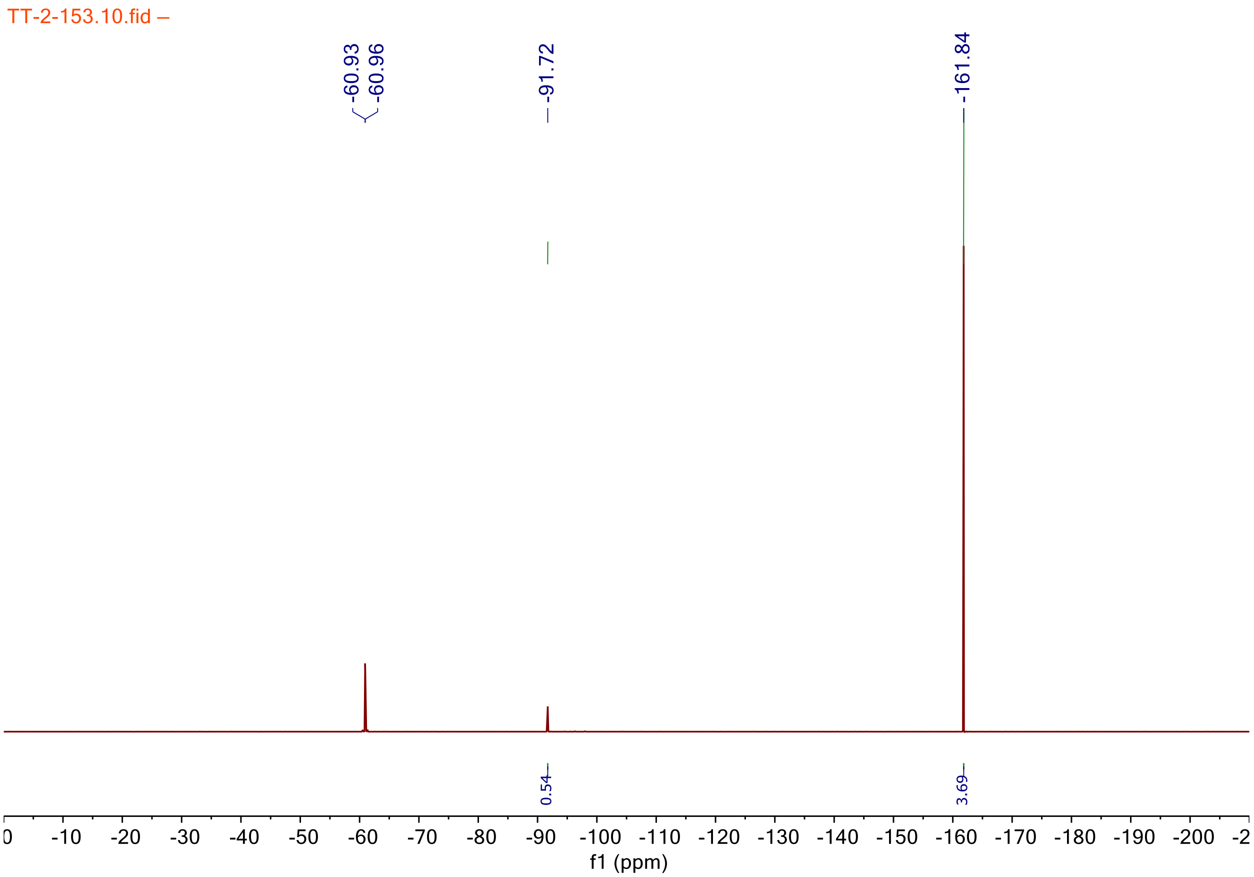

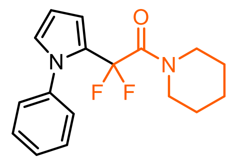

2,2-Difluoro-2-(1-phenyl-1*H*-pyrrol-2-yl)-1-(piperidin-1-yl)ethan-1-one (19): ^{1}H NMR (400 MHz, $CDCl_3$)

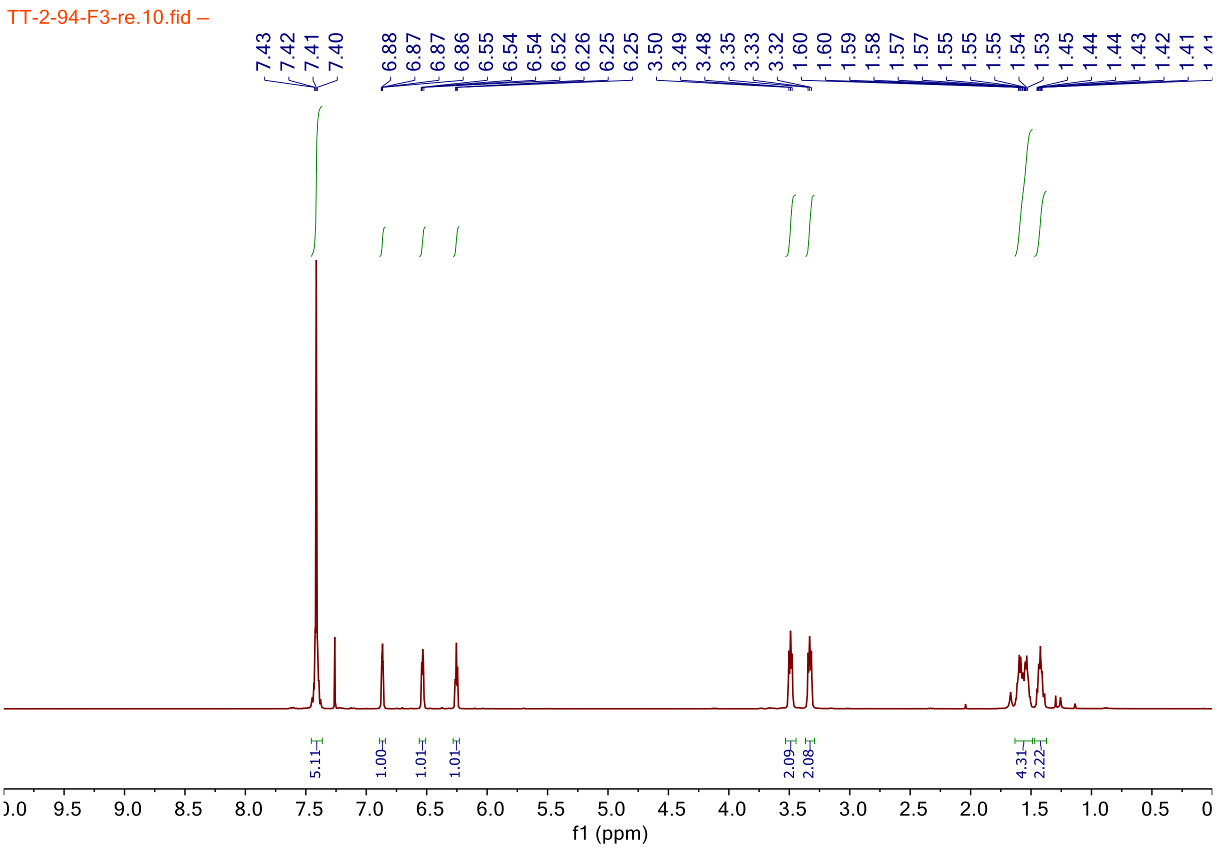

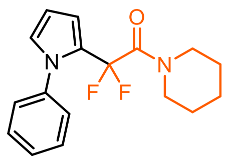

2,2-Difluoro-2-(1-phenyl-1*H*-pyrrol-2-yl)-1-(piperidin-1-yl)ethan-1-one (19): $^{13}C\{^{1}H\}$ NMR (201 MHz, $CDCl_3$)

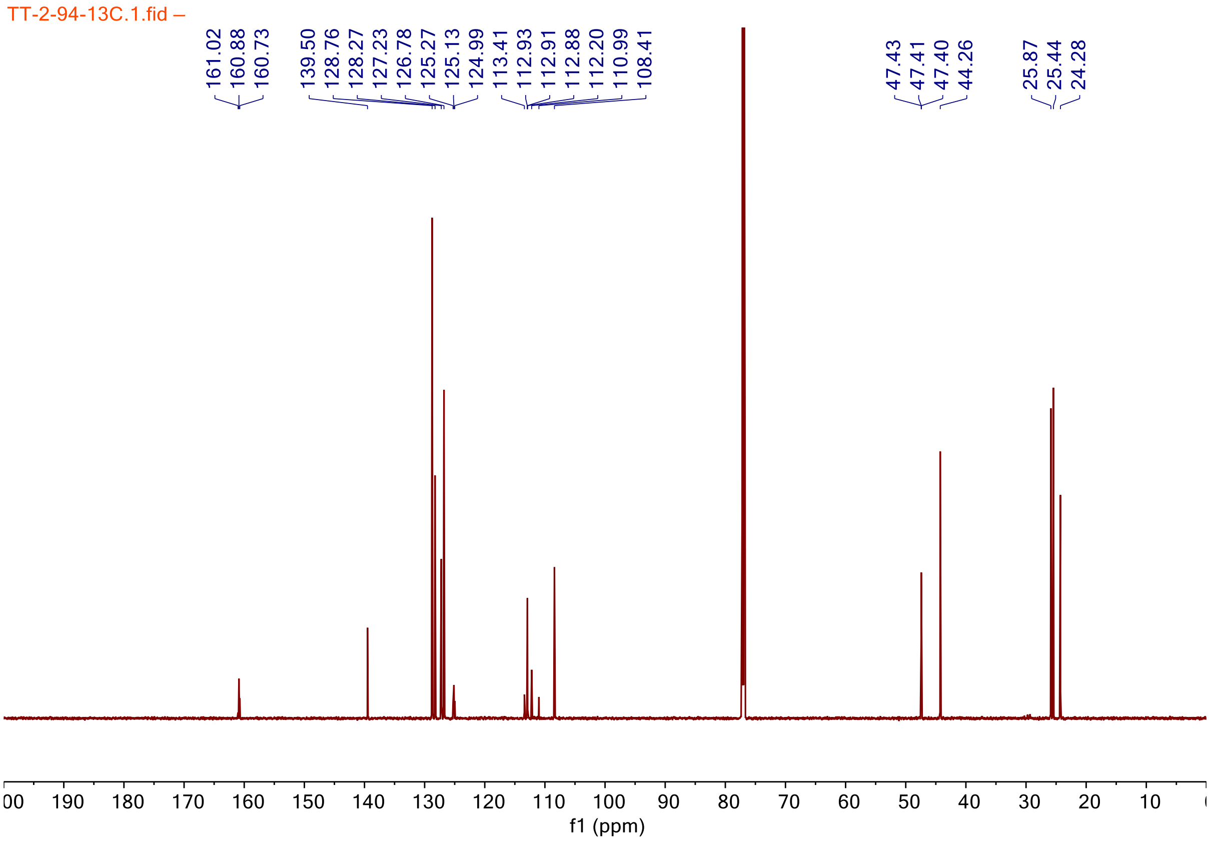

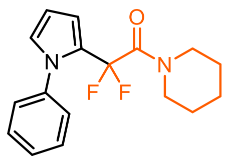

2,2-Difluoro-2-(1-phenyl-1*H*-pyrrol-2-yl)-1-(piperidin-1-yl)ethan-1-one (19): ^{19}F NMR (376 MHz, $CDCl_3$)

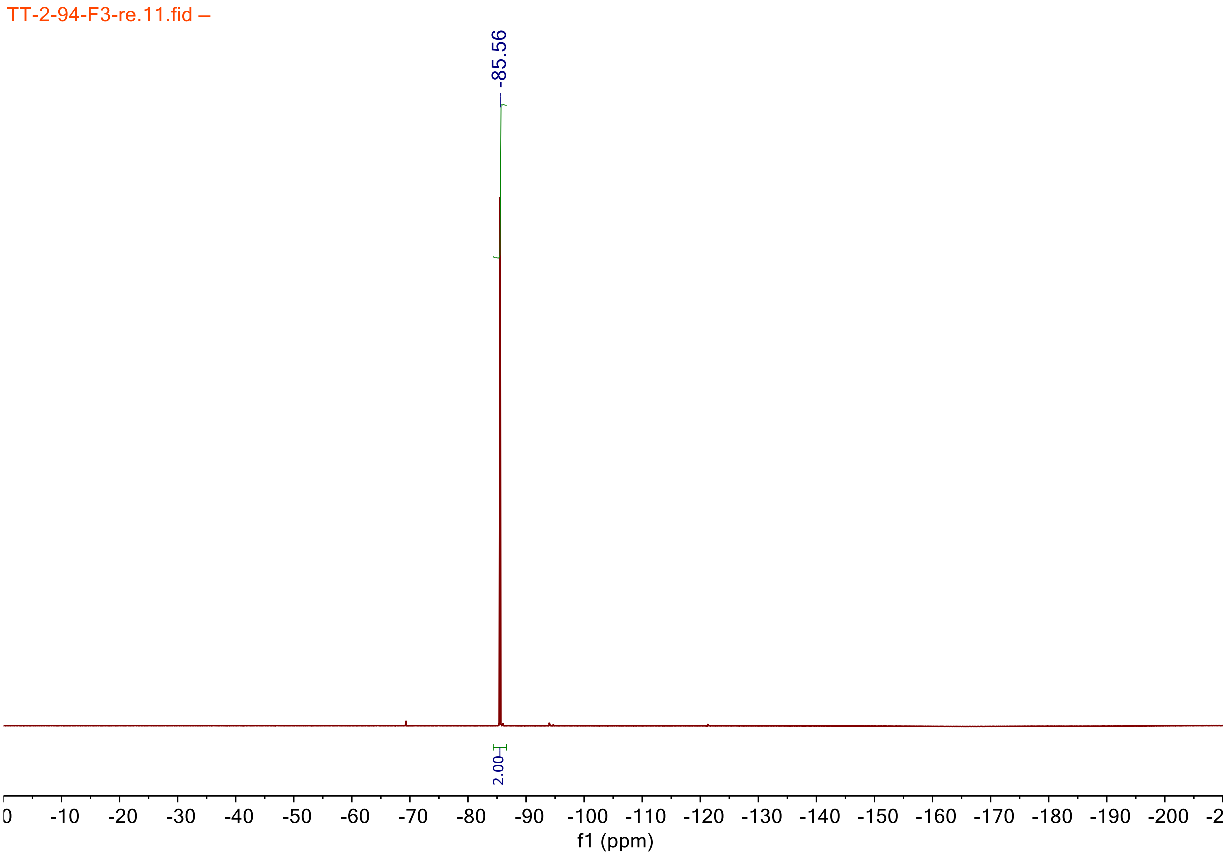

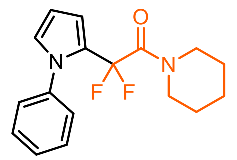

2,2-Difluoro-2-(1-phenyl-1*H*-pyrrol-2-yl)-1-(piperidin-1-yl)ethan-1-one (19): ^{19}F NMR (376 MHz, $CDCl_3$) for control reaction without Bu_4NI

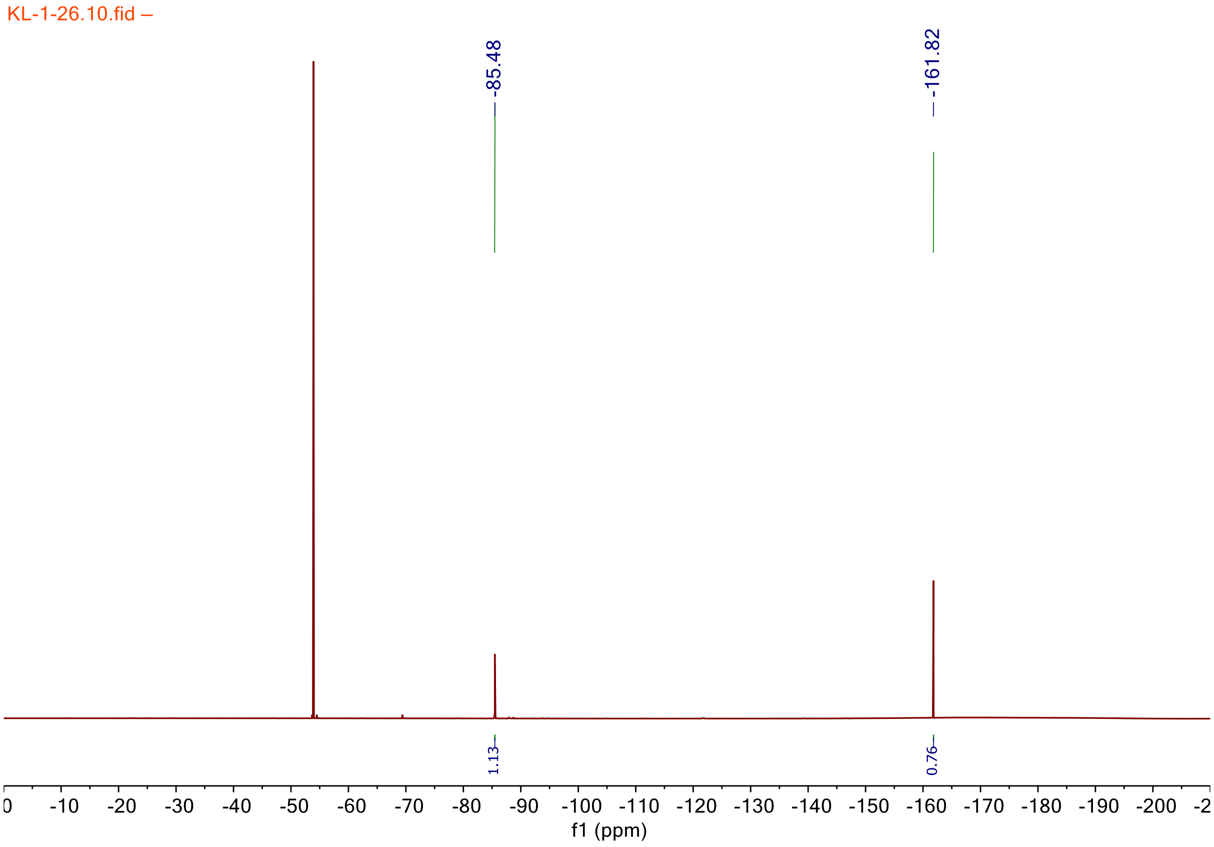

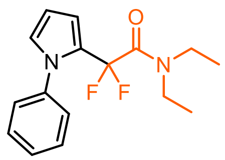

***N*,*N*-diethyl-2,2-difluoro-2-(1-phenyl-1*H*-pyrrol-2-yl)acetamide (20)**: ^{1}H NMR (400 MHz, $CDCl_3$)

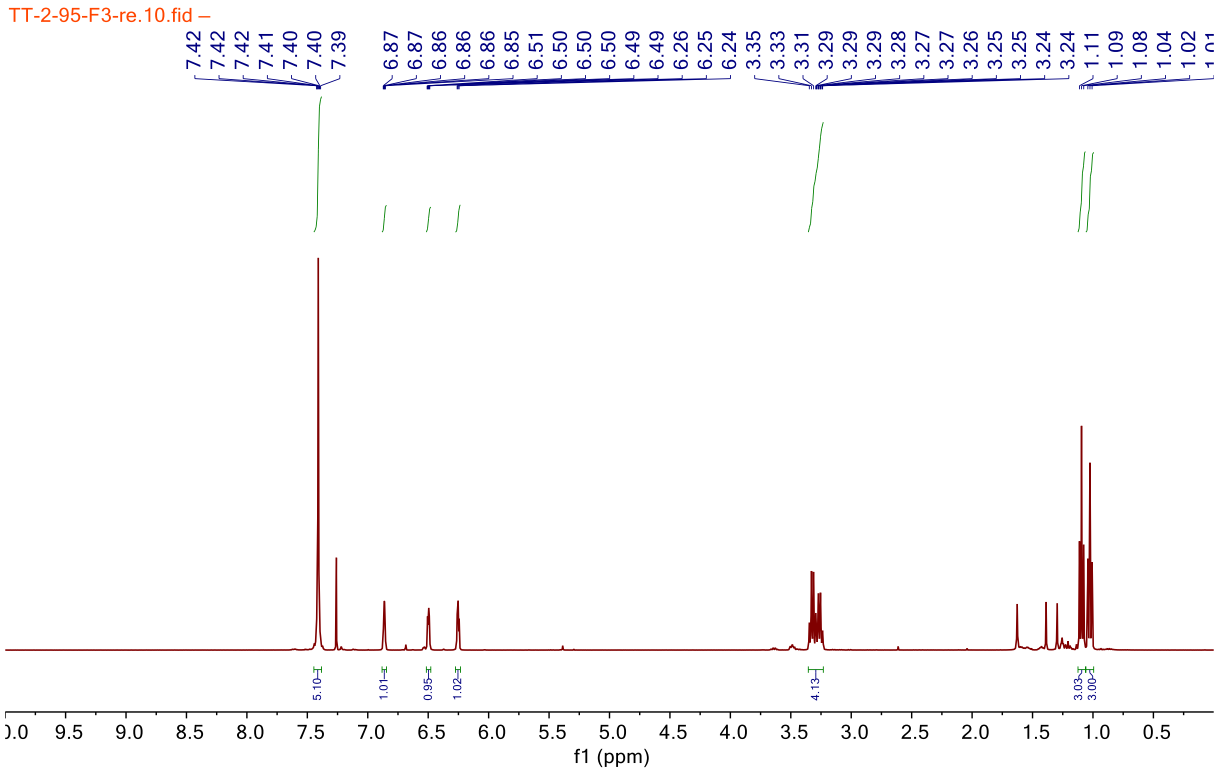

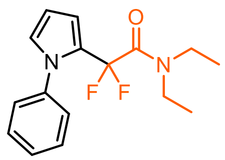

_N_,_N_-diethyl-2,2-difluoro-2-(1-phenyl-1_H_-pyrrol-2-yl)acetamide (20): $^{13}C\{^{1}H\}$ NMR (201 MHz, $CDCl_3$)

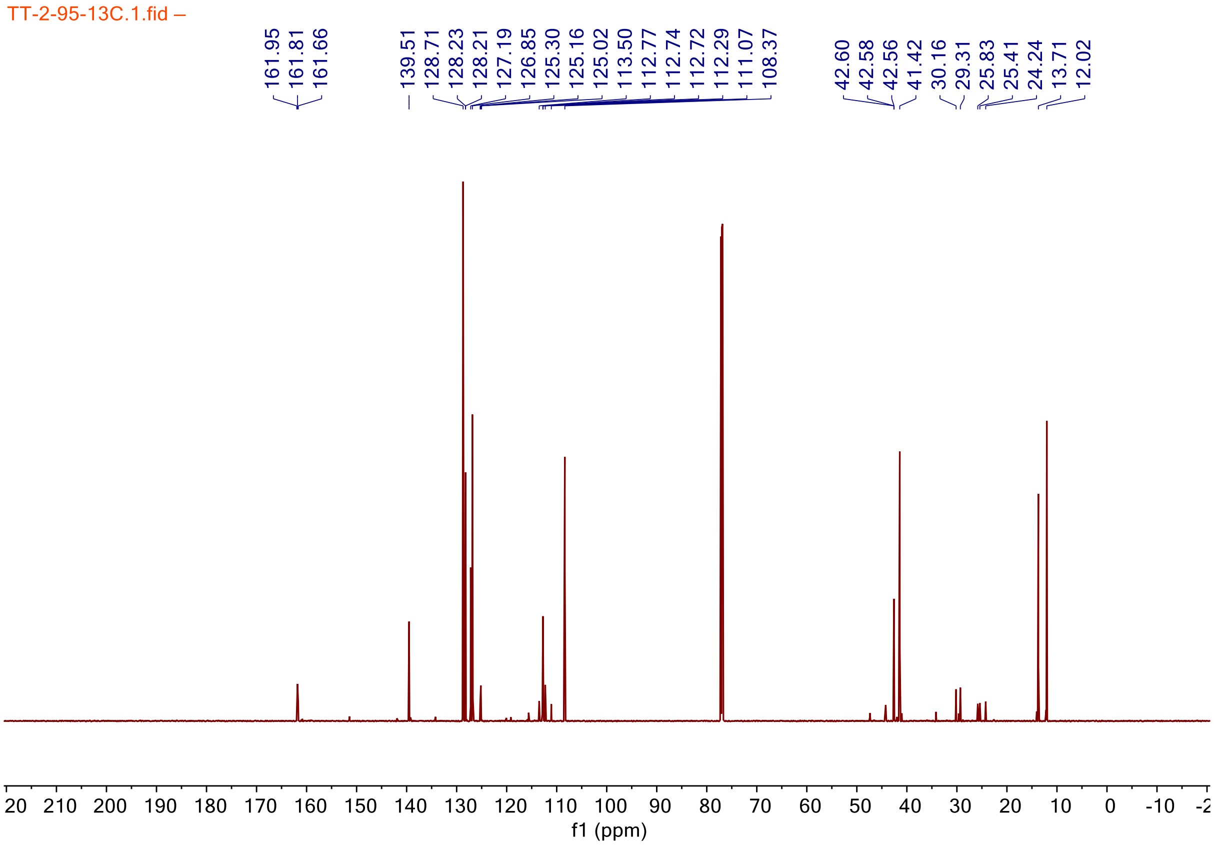

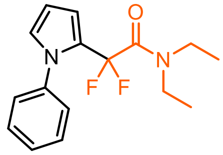

***N*,*N*-diethyl-2,2-difluoro-2-(1-phenyl-1*H*-pyrrol-2-yl)acetamide (20)**: ^{19}F NMR (376 MHz, $CDCl_3$)

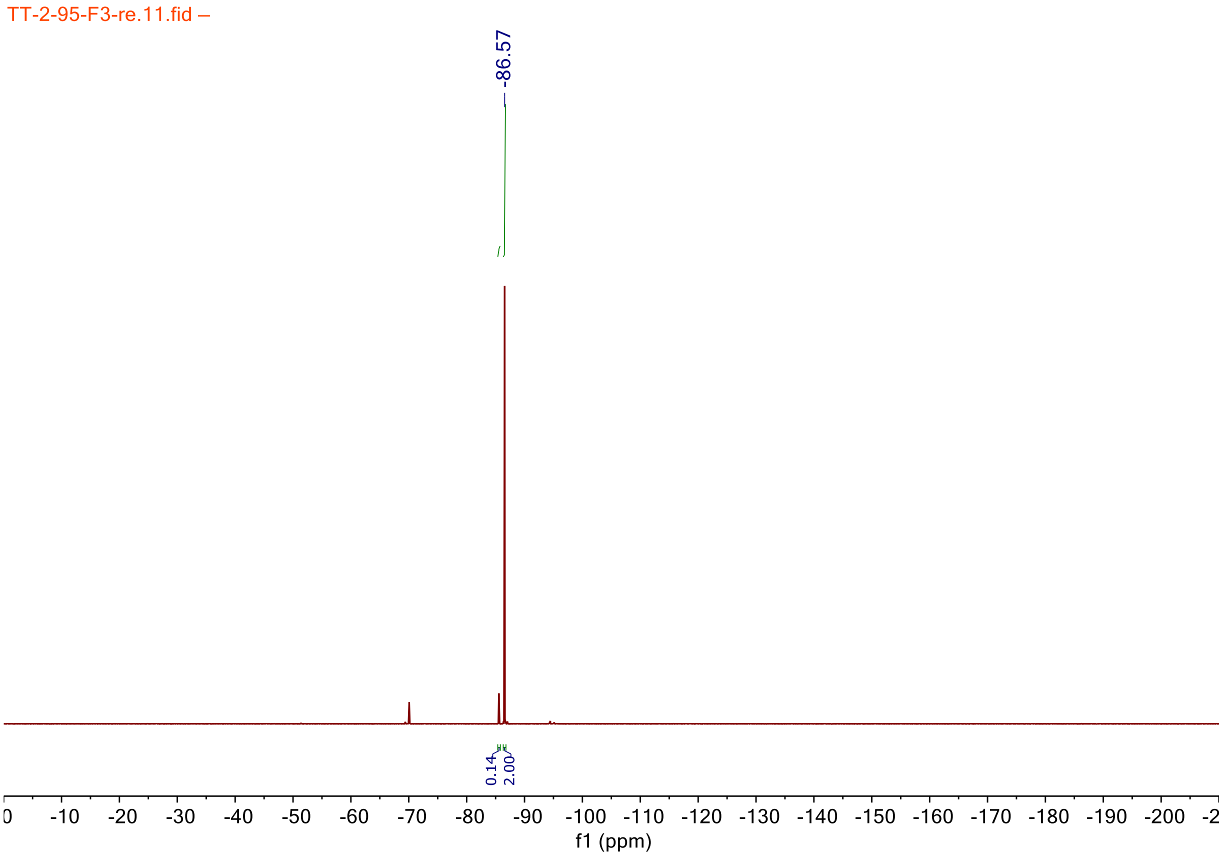

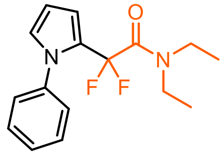

***N*,*N*-diethyl-2,2-difluoro-2-(1-phenyl-1*H*-pyrrol-2-yl)acetamide (20)**: ^{19}F NMR (376 MHz, $CDCl_3$) for control reaction without Bu_4NI

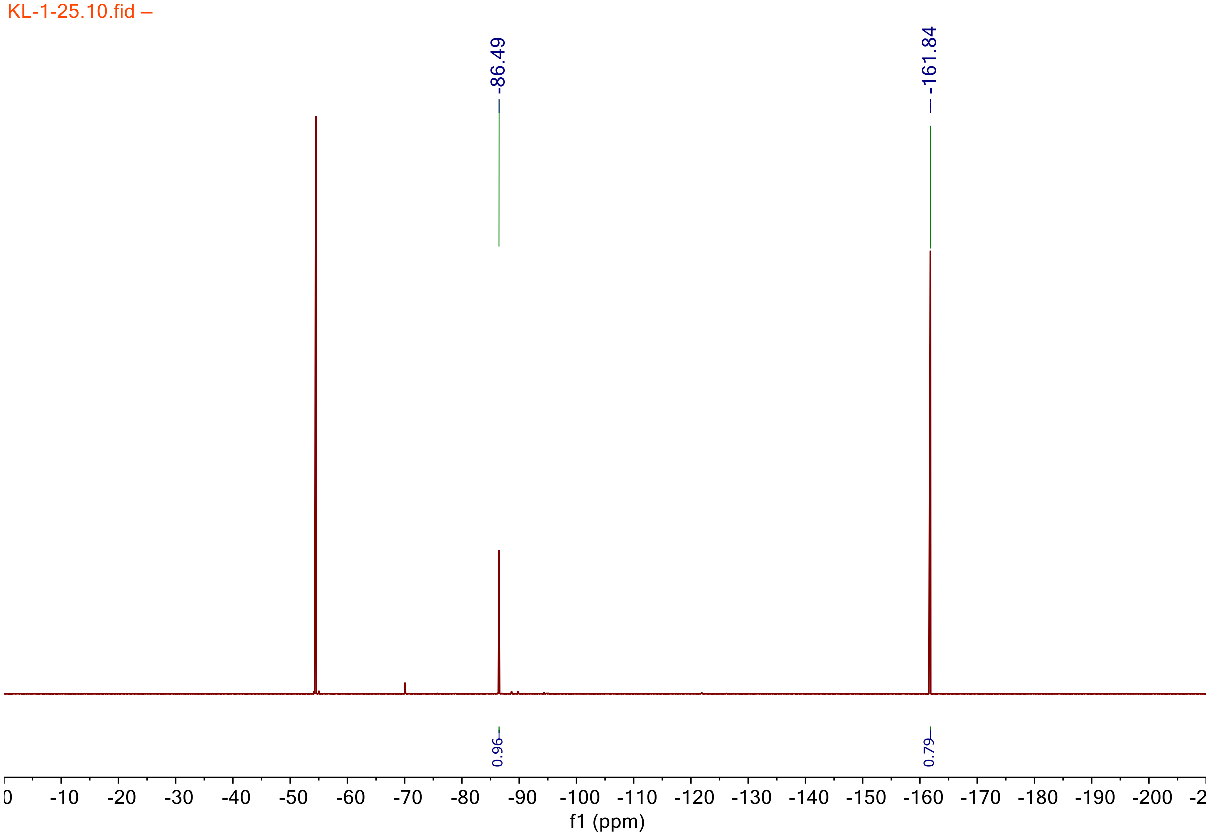

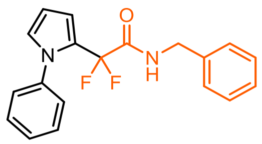

N-benzyl-2,2-difluoro-2-(1-phenyl-1H-pyrrol-2-yl)acetamide (21): ^{1}H NMR (400 MHz, $CDCl_3$)

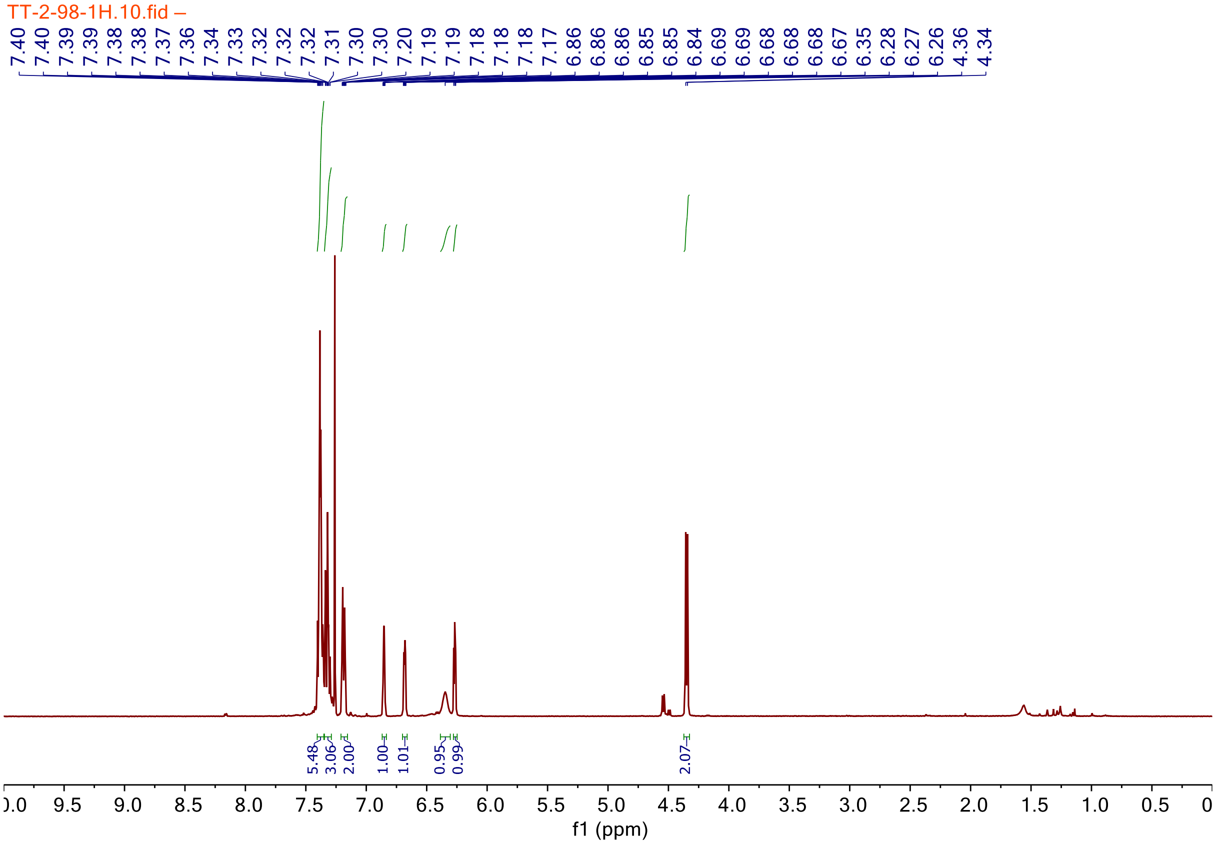

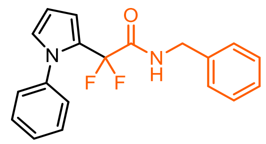

***N*-benzyl-2,2-difluoro-2-(1-phenyl-1*H*-pyrrol-2-yl)acetamide (21)**: $^{13}C\{^1H\}$ NMR (201 MHz, $CDCl_3$)

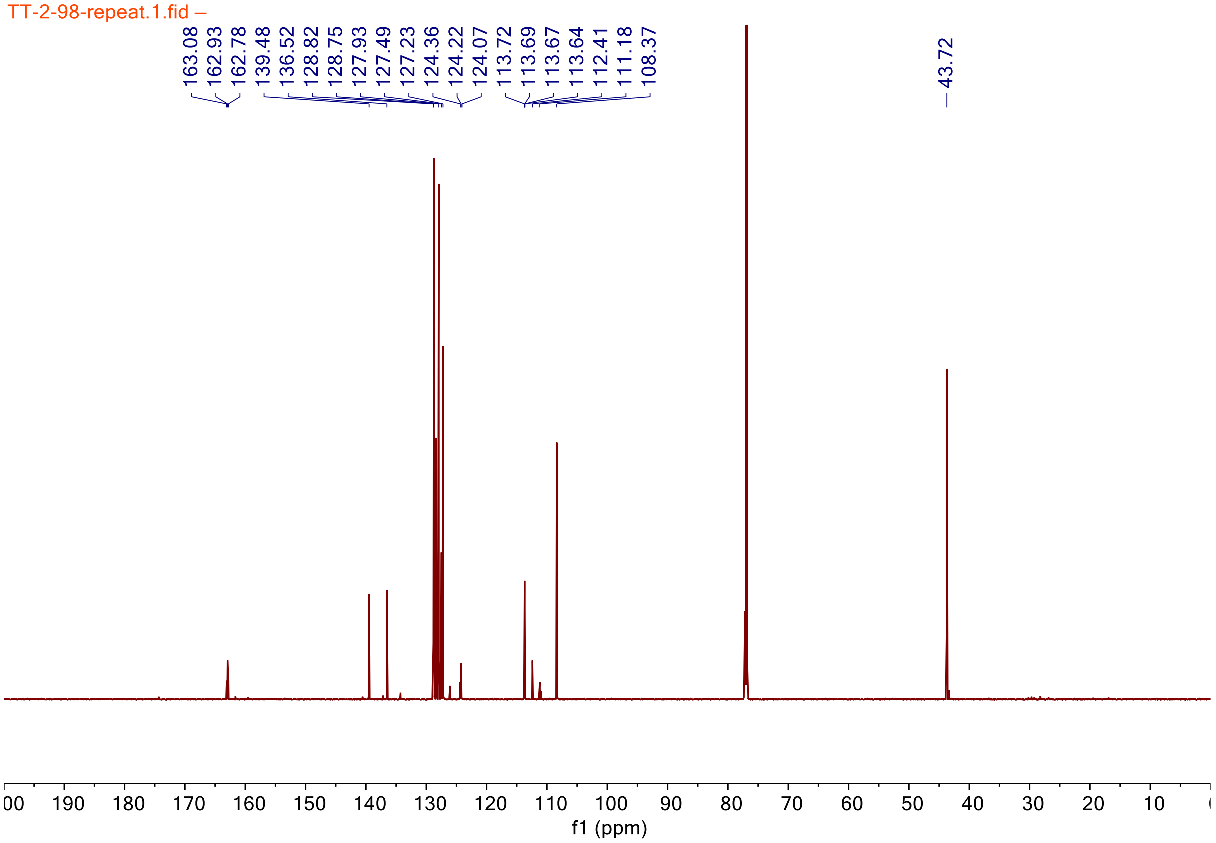

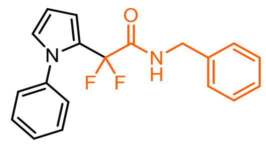

***N*-benzyl-2,2-difluoro-2-(1-phenyl-1*H*-pyrrol-2-yl)acetamide (21)**: ^{19}F NMR (376 MHz, $CDCl_3$)

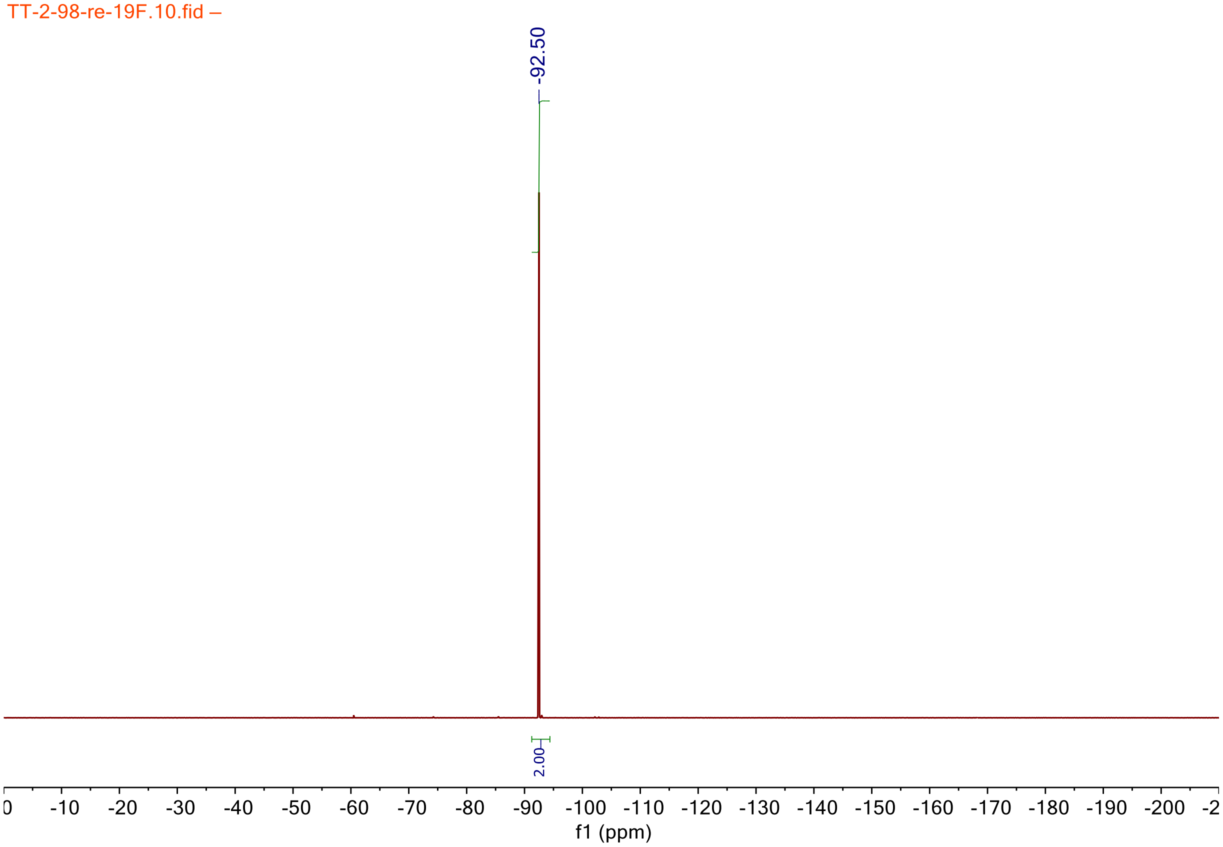

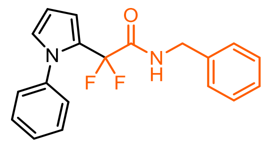

***N*-benzyl-2,2-difluoro-2-(1-phenyl-1*H*-pyrrol-2-yl)acetamide (21)**: ^{19}F NMR (376 MHz, $CDCl_3$) for control reaction without Bu_4NI

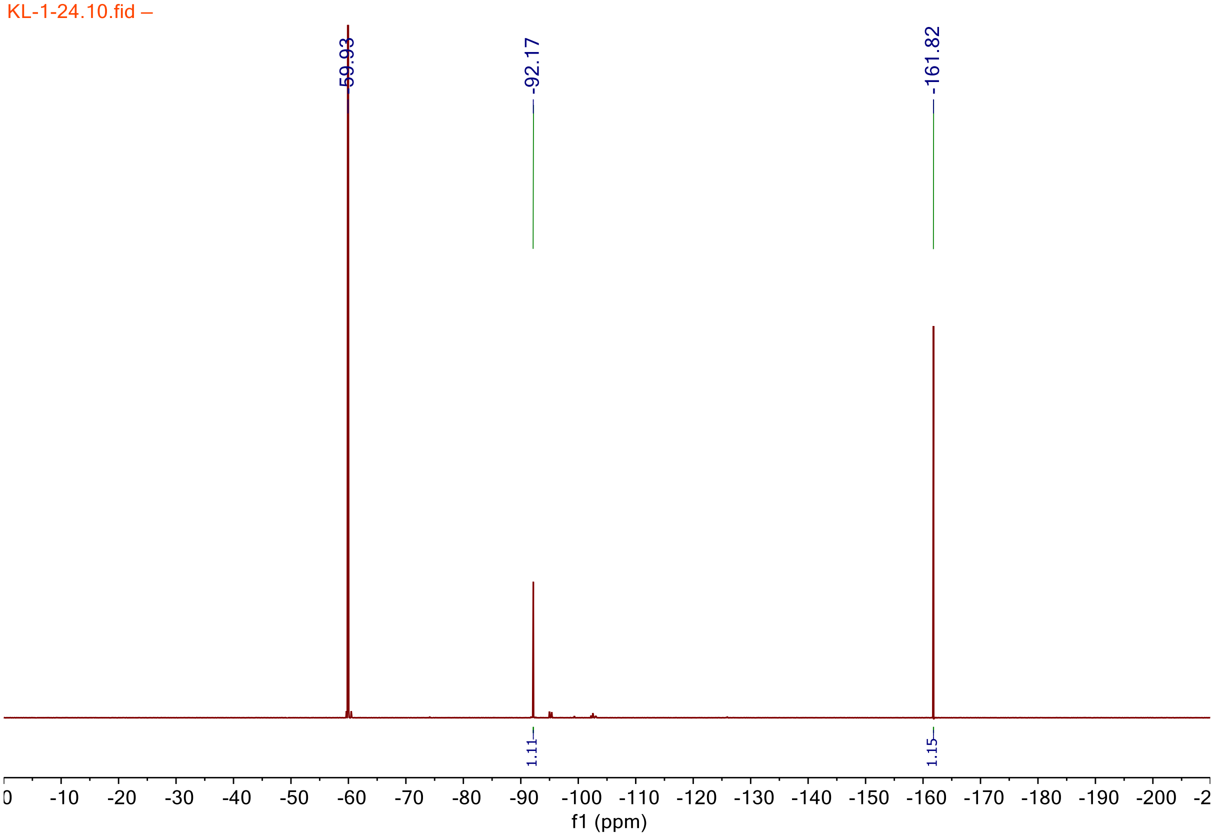

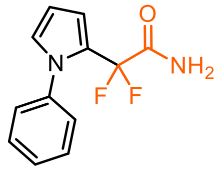

2,2-Difluoro-2-(1-phenyl-1*H*-pyrrol-2-yl)acetamide (22): ^{1}H NMR (400 MHz, $CDCl_3$)

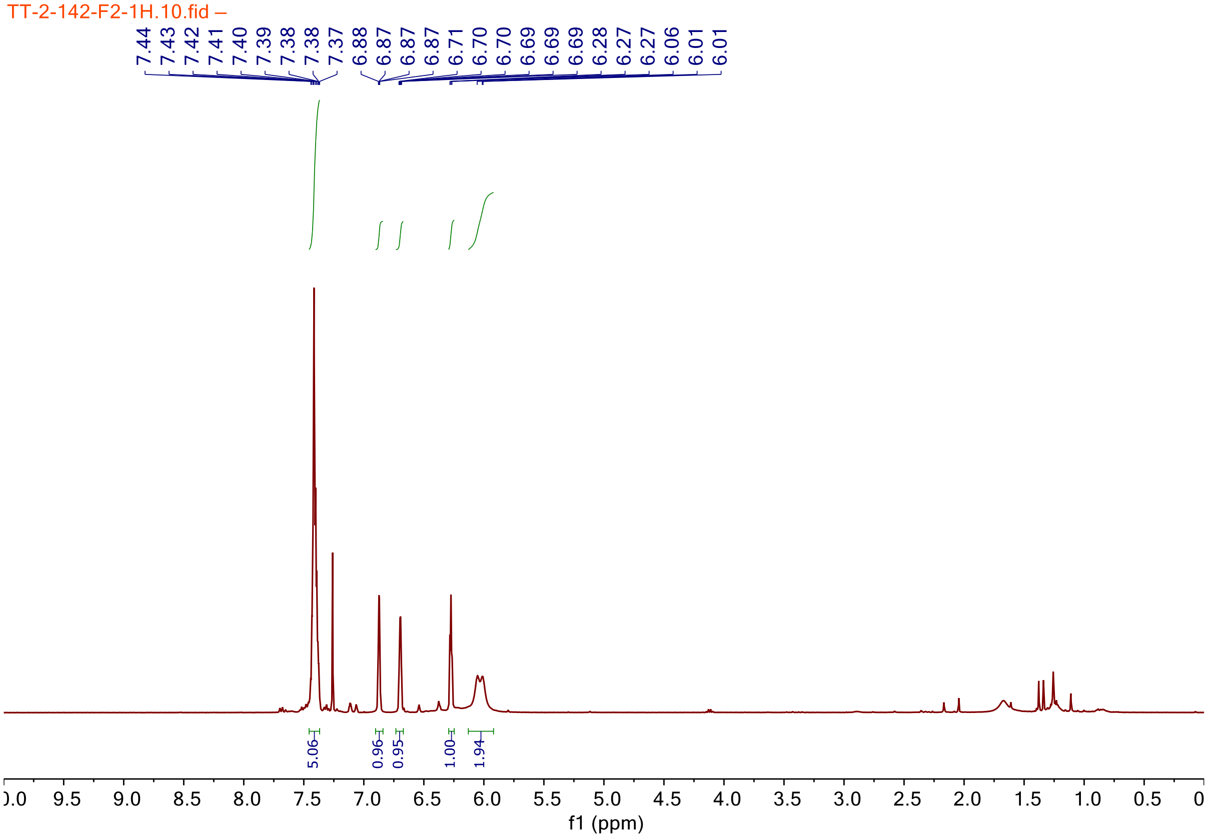

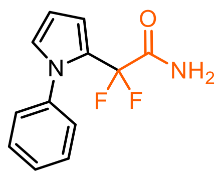

2,2-Difluoro-2-(1-phenyl-1*H*-pyrrol-2-yl)acetamide (22): $^{13}C\{^1H\}$ NMR (201 MHz, $CDCl_3$)

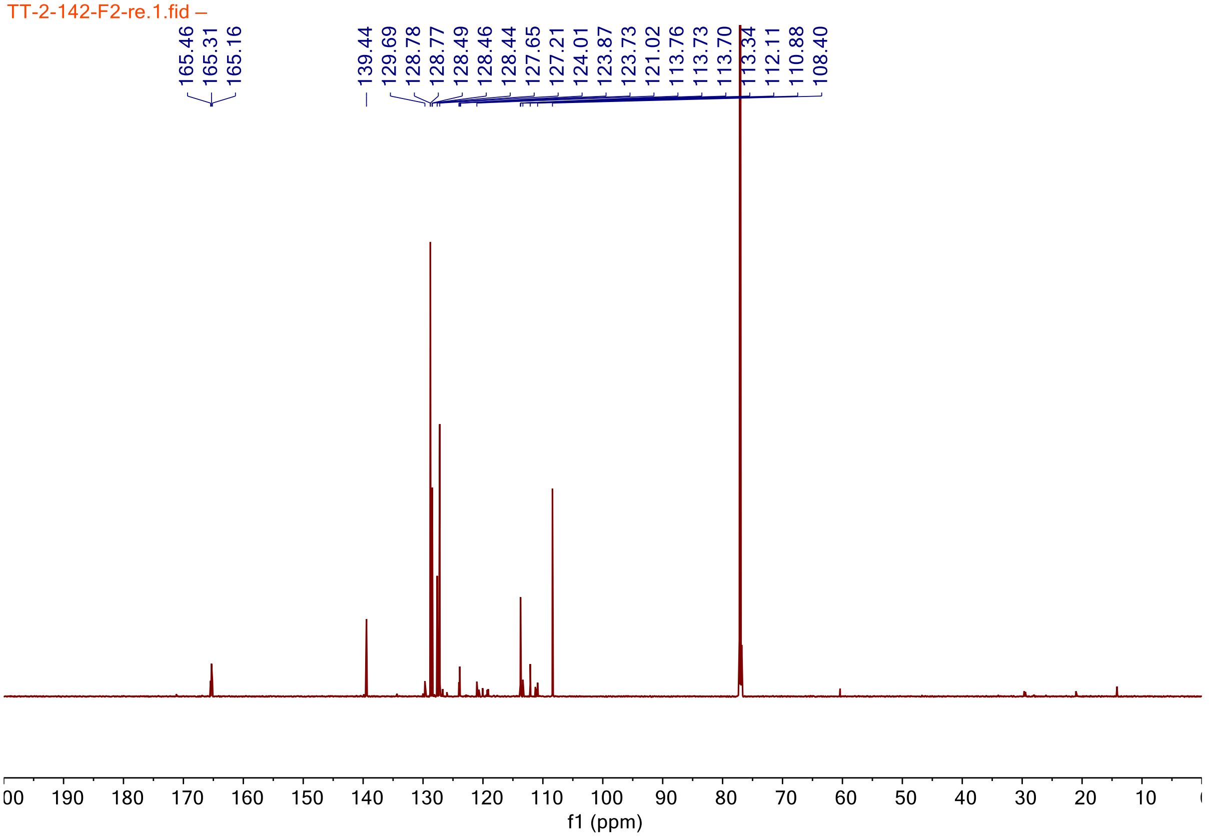

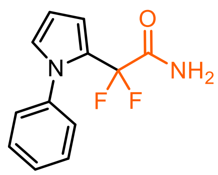

2,2-Difluoro-2-(1-phenyl-1*H*-pyrrol-2-yl)acetamide (22): ^{19}F NMR (376 MHz, $CDCl_3$)

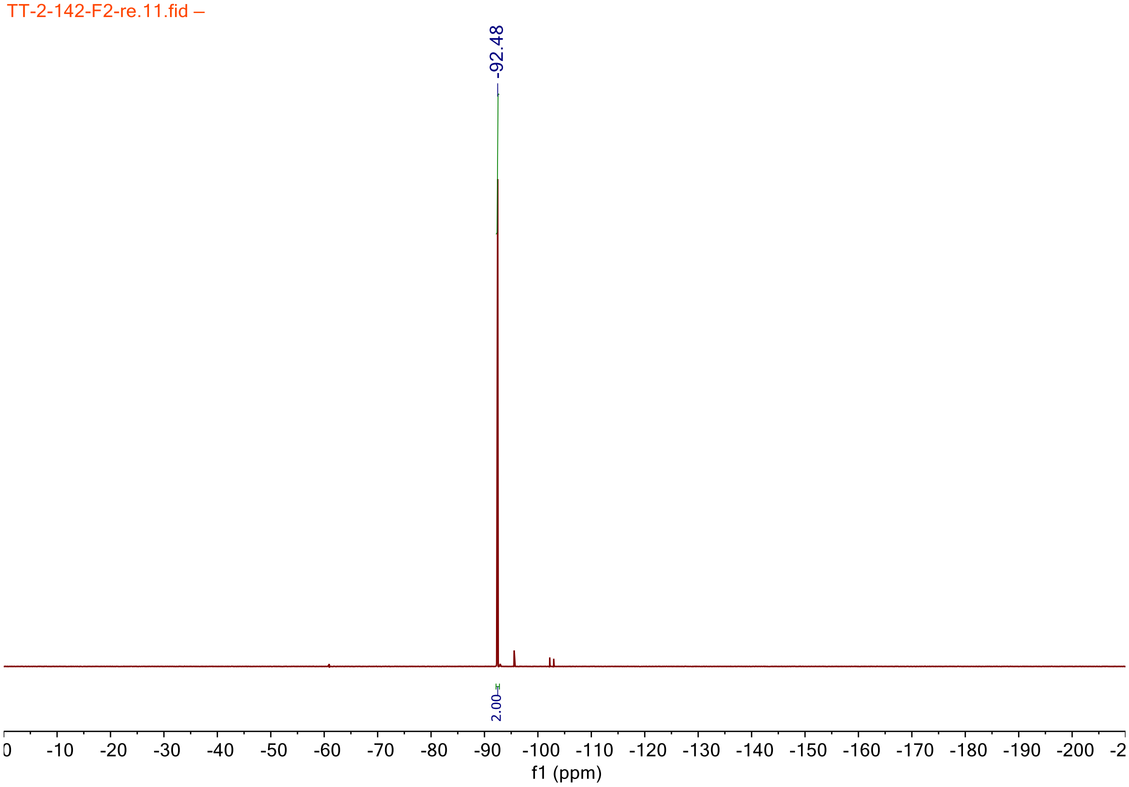

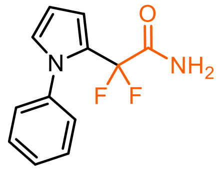

2,2-Difluoro-2-(1-phenyl-1*H*-pyrrol-2-yl)acetamide (22): ^{19}F NMR (376 MHz, $CDCl_3$) for control reaction without Bu_4NI

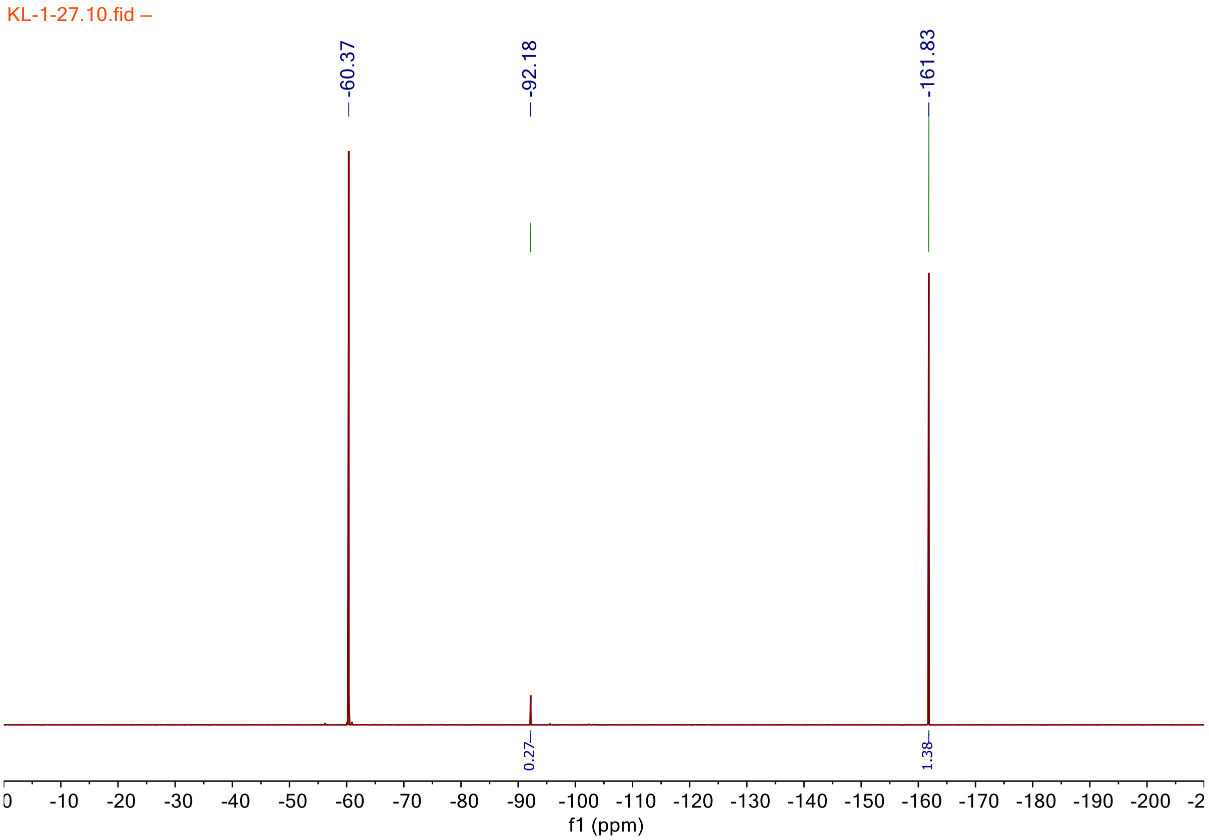

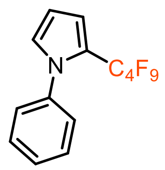

2-(Perfluorobutyl)-1-phenyl-1*H*-pyrrole (23): ^{1}H NMR (400 MHz, $CDCl_3$)

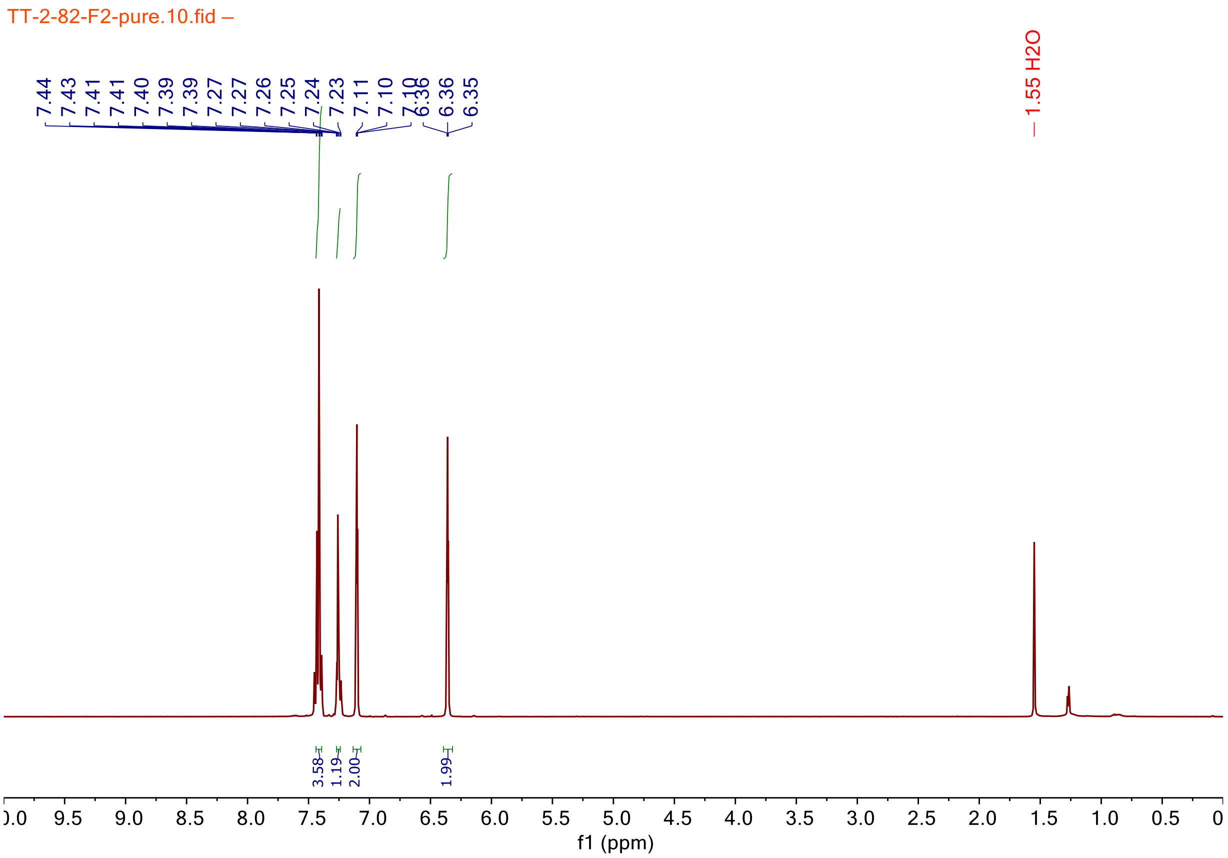

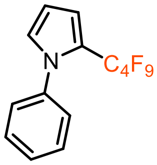

2-(Perfluorobutyl)-1-phenyl-1*H*-pyrrole (23): $^{13}C\{^{1}H\}$ NMR (201 MHz, $CDCl_3$)

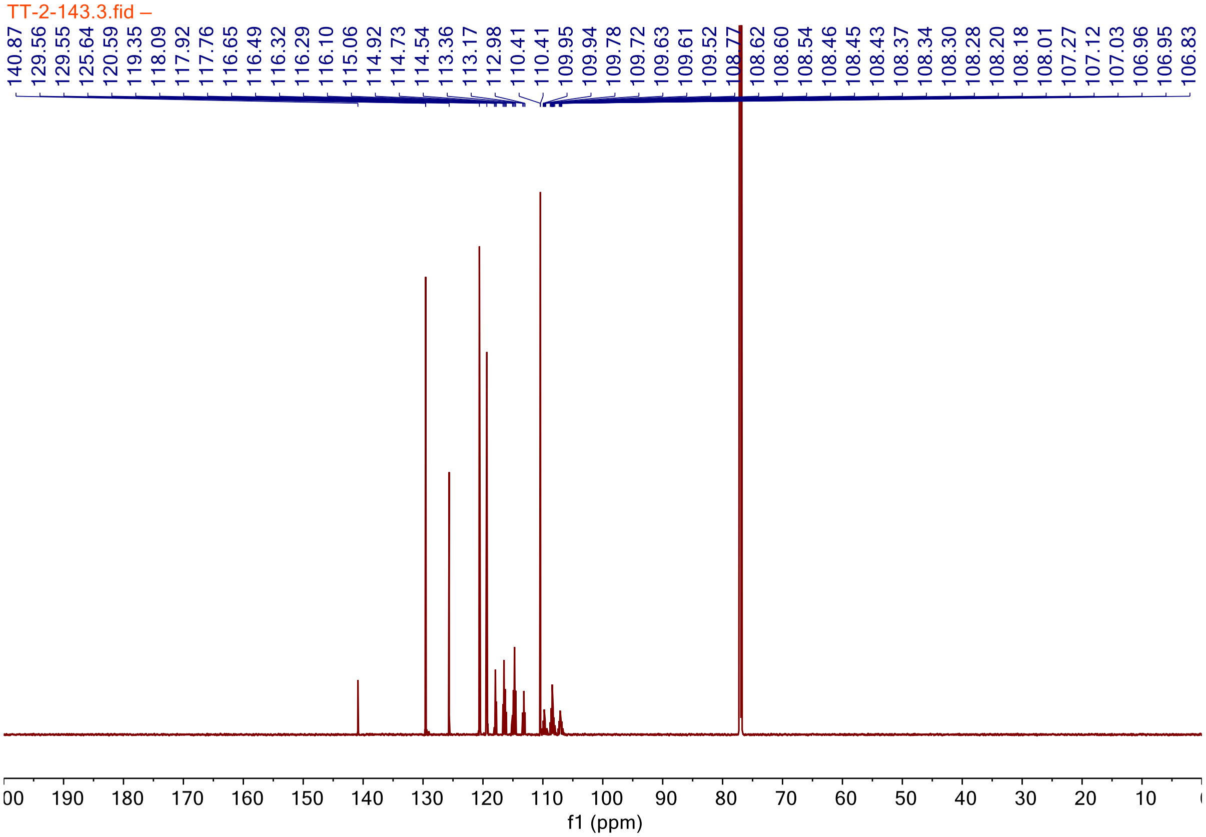

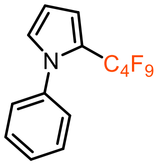

2-(Perfluorobutyl)-1-phenyl-1*H*-pyrrole (23) ^{19}F NMR (376 MHz, $CDCl_3$)

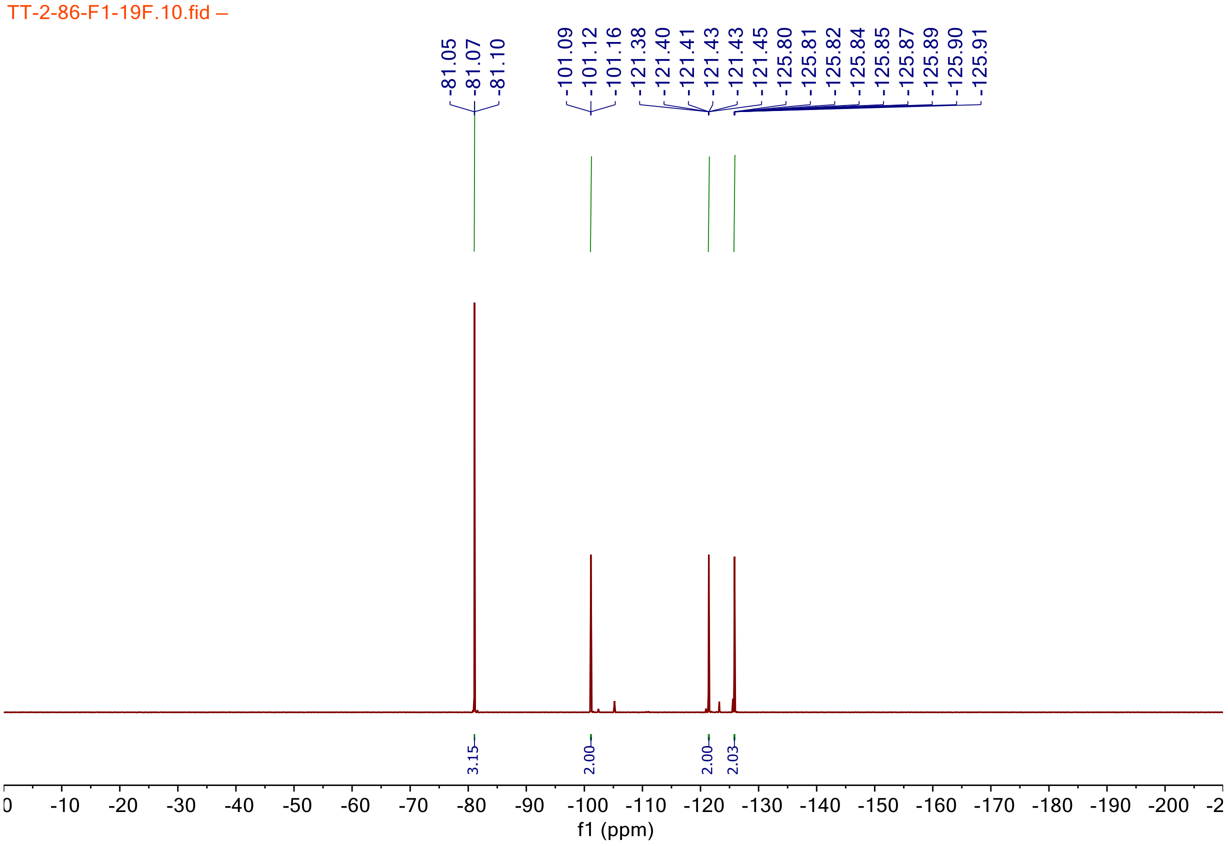

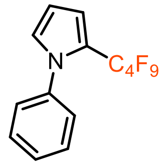

2-(Perfluorobutyl)-1-phenyl-1*H*-pyrrole (23): ^{19}F NMR (376 MHz, $CDCl_3$) for control reaction without Bu_4NI

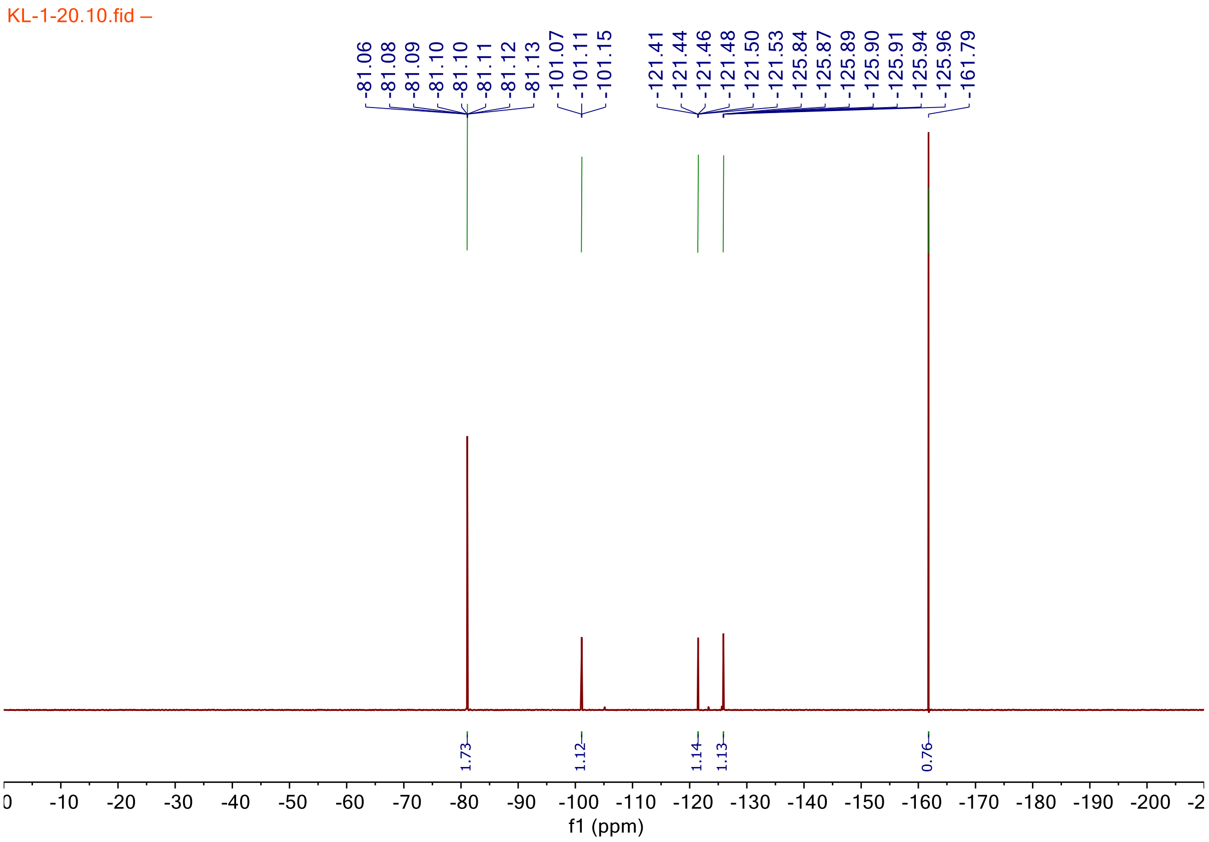

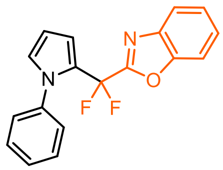

2-(Difluoro(1-phenyl-1*H*-pyrrol-2-yl)methyl)benzo[*d*]oxazole (24): ^{1}H NMR (400 MHz, $CDCl_3$)

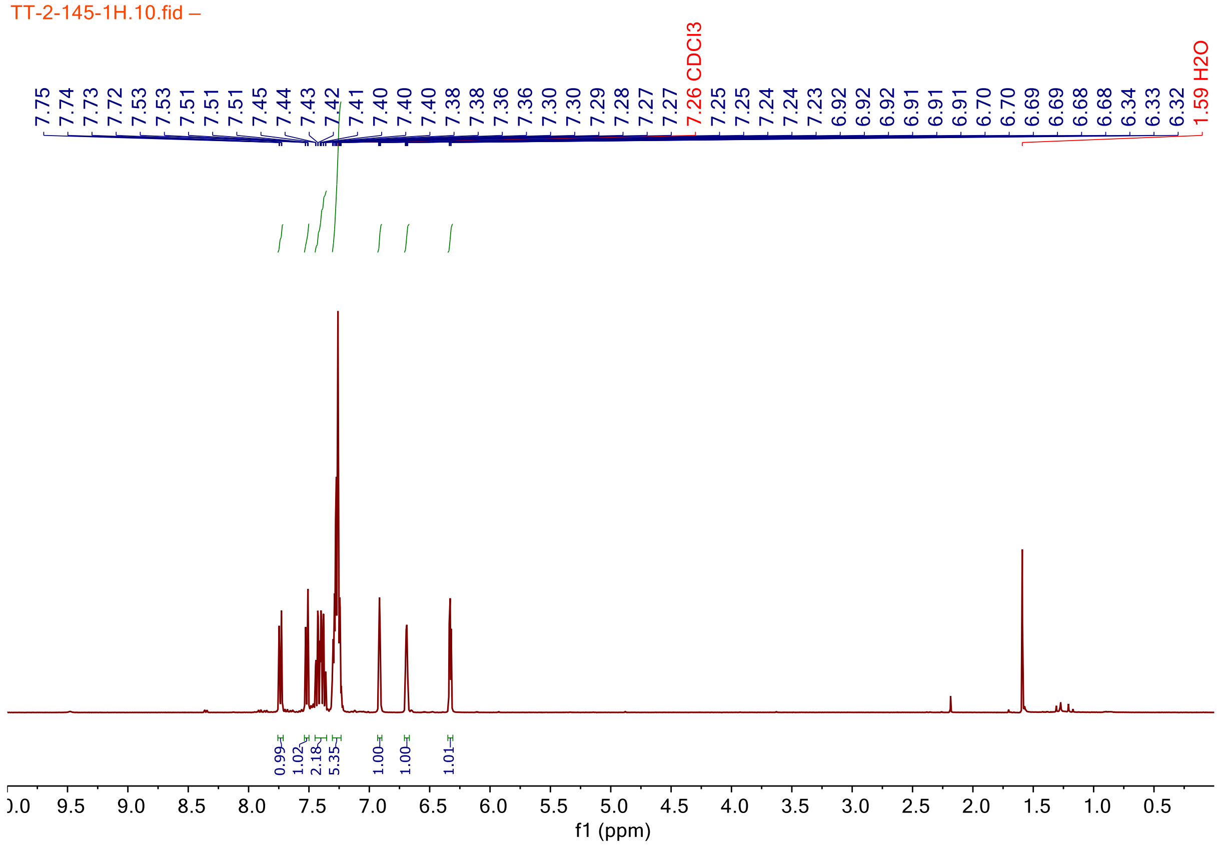

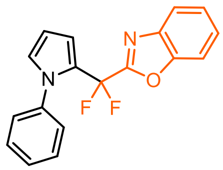

2-(Difluoro(1-phenyl-1*H*-pyrrol-2-yl)methyl)benzo[*d*]oxazole (24): $^{13}C\{^{1}H\}$ NMR (201 MHz, $CDCl_3$)

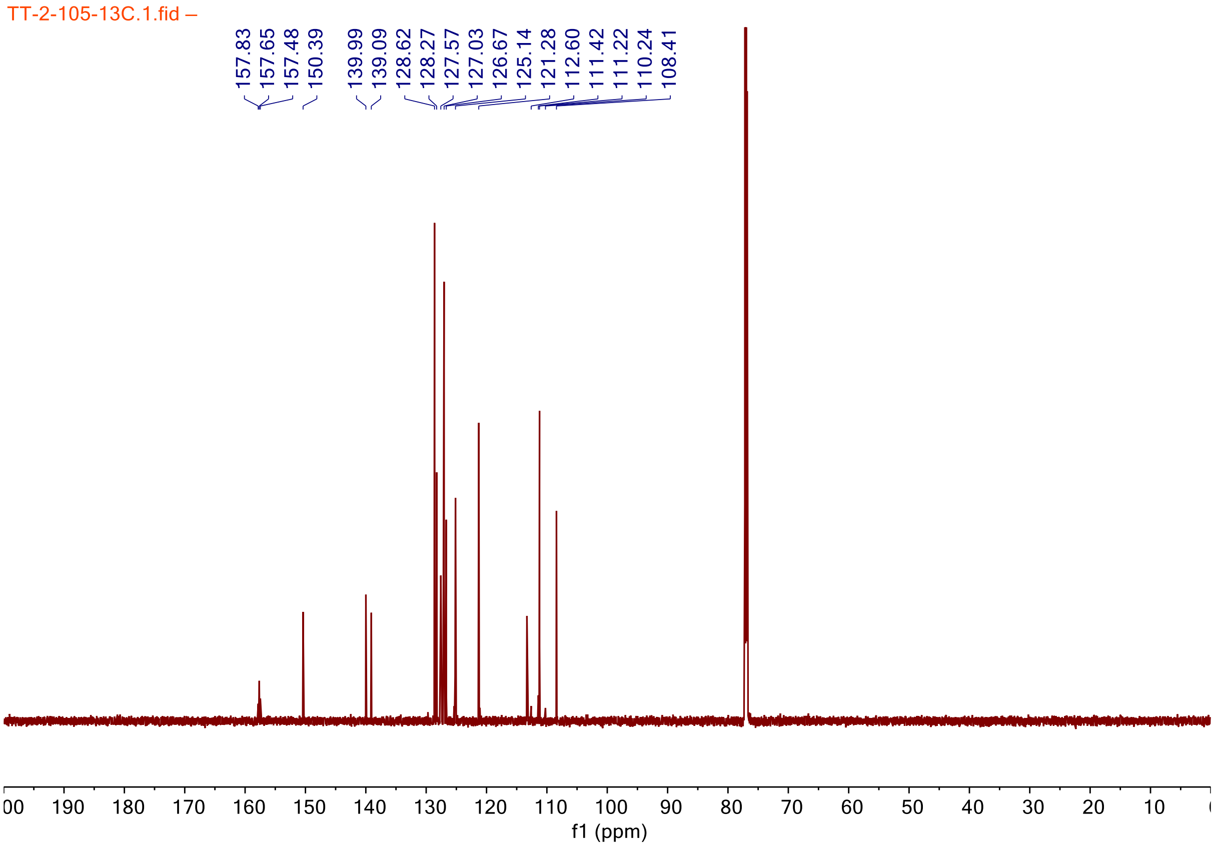

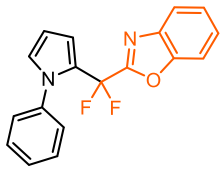

2-(Difluoro(1-phenyl-1*H*-pyrrol-2-yl)methyl)benzo[*d*]oxazole (24): ^{19}F NMR (376 MHz, $CDCl_3$)

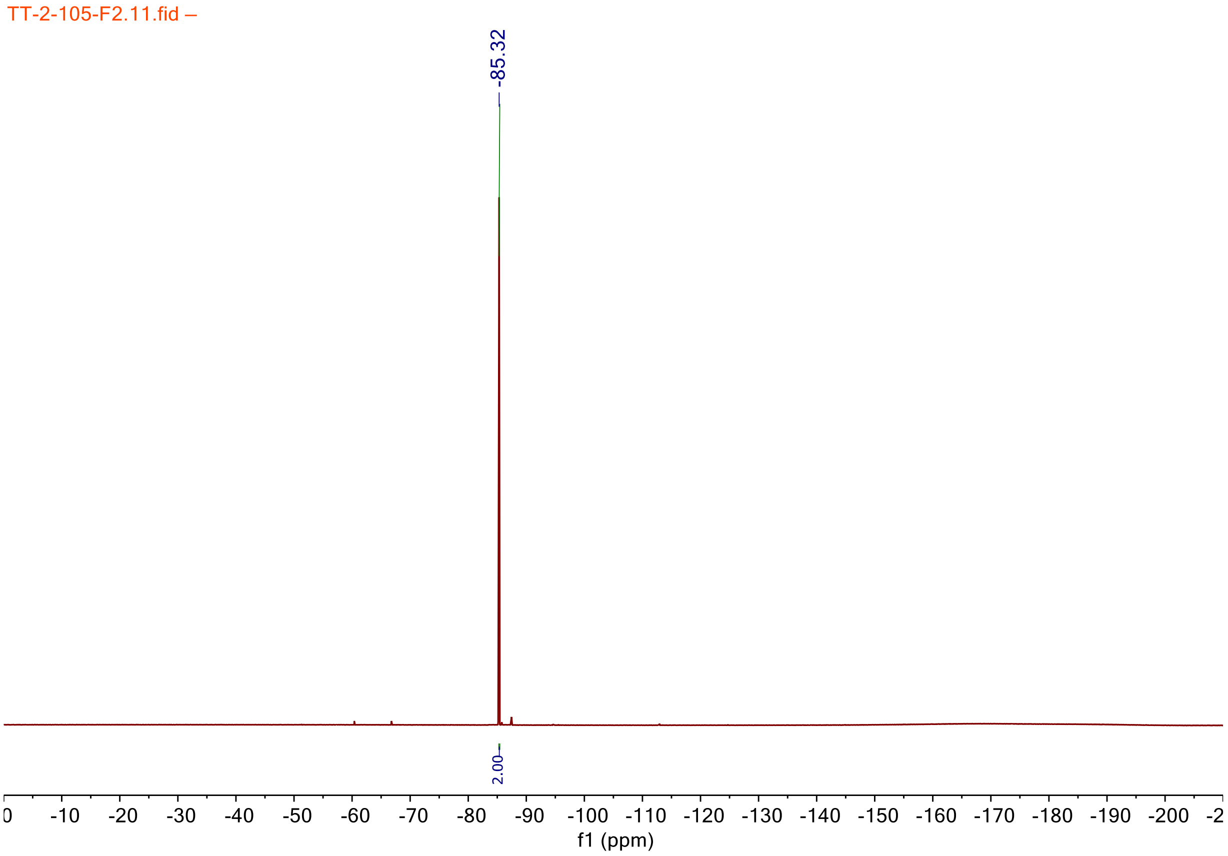

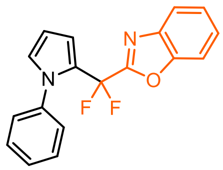

2-(Difluoro(1-phenyl-1*H*-pyrrol-2-yl)methyl)benzo[*d*]oxazole (24): ^{19}F NMR (376 MHz, $CDCl_3$) for control reaction without Bu_4NI

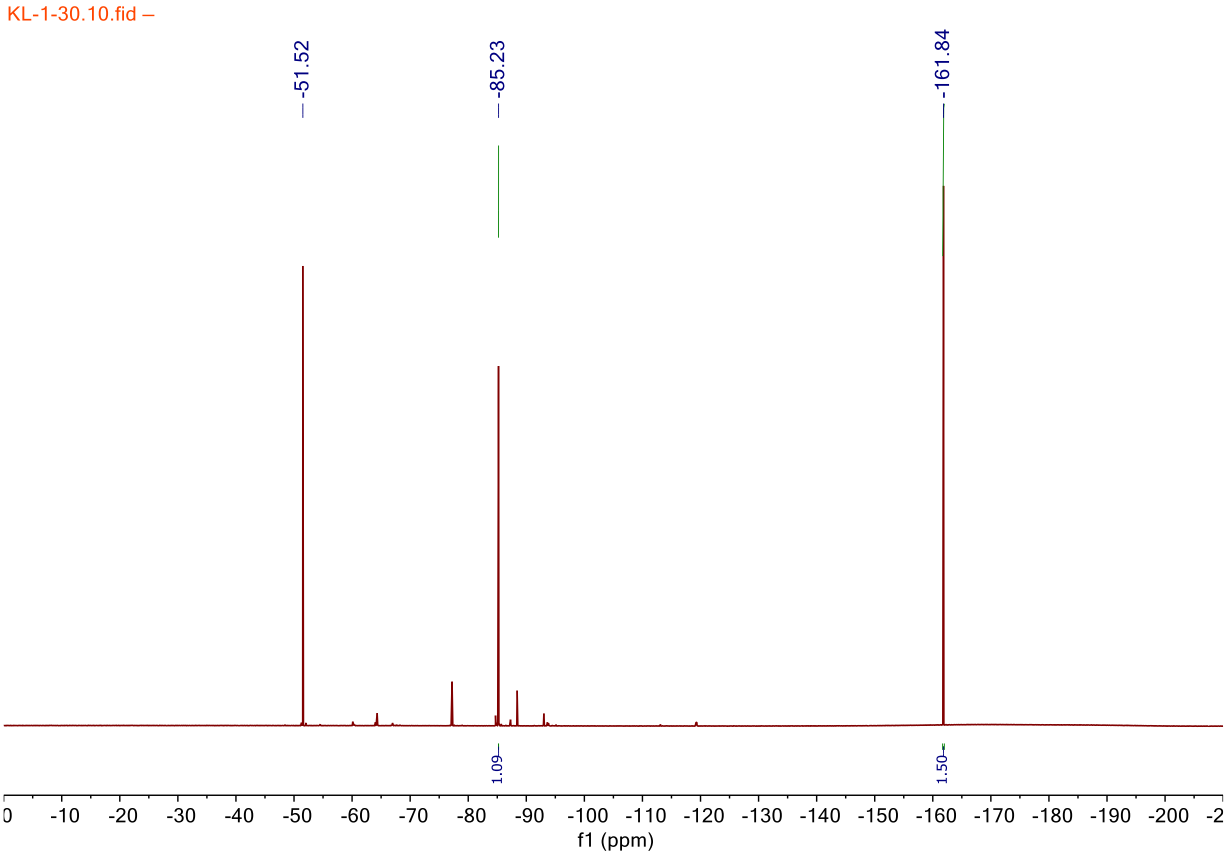

Supplement: Supplementary file 1 — jo4c02413_si_001.pdf [file jo4c02413_si_001.pdf]
